# Mapping the regulatory landscape for artificial intelligence in health within the European Union

**DOI:** 10.1038/s41746-024-01221-6

**Published:** 2024-08-27

**Authors:** Jelena Schmidt, Nienke M. Schutte, Stefan Buttigieg, David Novillo-Ortiz, Eric Sutherland, Michael Anderson, Bart de Witte, Michael Peolsson, Brigid Unim, Milena Pavlova, Ariel Dora Stern, Elias Mossialos, Robin van Kessel

**Affiliations:** 1 Department of International Health, Care and Public Health Research Institute (CAPHRI), Faculty of Health, Medicine and Life Sciences, Maastricht University, Maastricht, Netherlands; 2 Innovation in Health Information Systems Unit, SD Data Governance, Sciensano, Brussels, Belgium; 3 Ministry for Health and Active Ageing, Valletta, Malta; 4 Faculty of Health Sciences, University of Malta, Msida, Malta; 5 Division of Country Health Policies and Systems, World Health Organization Regional Office for Europe, Copenhagen, Denmark; 6 Independent Researcher, Paris, France; 7 LSE Health, Department of Health Policy, London School of Economics and Political Science, London, United Kingdom; 8 Hippo AI Foundation, Berlin, Germany; 9 Swedish eHealth Agency, Stockholm, Sweden; 10 Department of Cardiovascular, Endocrine-Metabolic Diseases and Aging, National Institute of Health, Rome, Italy; 11 Department of Health Services Research, Care and Public Health Research Institute (CAPHRI), Faculty of Health, Medicine and Life Sciences, Maastricht University, Maastricht, Netherlands; 12 Harvard Business School Technology and Operations Management, Boston, MS USA; 13 Harvard-MIT Center for Regulatory Science, Boston, MS, USA; 14 Digital Health Cluster, Hasso-Plattner Institute, University of Potsdam, Potsdam, Germany; 15 Institute of Global Health Innovation, Imperial College London, London, United Kingdom; 16 Department of Psychiatry, University of Cambridge, Cambridge, United Kingdom

**Keywords:** Health policy, Public health, Information technology, Law

## Abstract

Regulatory frameworks for artificial intelligence (AI) are needed to mitigate risks while ensuring the ethical, secure, and effective implementation of AI technology in healthcare and population health. In this article, we present a synthesis of 141 binding policies applicable to AI in healthcare and population health in the EU and 10 European countries. The EU AI Act sets the overall regulatory framework for AI, while other legislations set social, health, and human rights standards, address the safety of technologies and the implementation of innovation, and ensure the protection and safe use of data. Regulation specifically pertaining to AI is still nascent and scarce, though a combination of data, technology, innovation, and health and human rights policy has already formed a baseline regulatory framework for AI in health. Future work should explore specific regulatory challenges, especially with respect to AI medical devices, data protection, and data enablement.

## Introduction

Artificial Intelligence (AI) uses algorithms or models to perform tasks and exhibit behaviours such as learning, taking decisions, and making predictions^1^. In recent years, AI technologies have increasingly penetrated society, resulting in an array of potential health benefits and risks^2^. AI technologies can unlock considerable benefits for patients, clinicians, and healthcare services (e.g., identifying new medicines, predicting hospital readmissions or recognising pathology in medical images)^3^. In population health, applying AI technologies to real-world data may bring new insights into the determinants of health of populations^4–7^, assist in identifying disease patterns, or support drug discovery and development^1,6,8^. However, AI technologies may equally propagate behaviours harmful to health by incentivising addictive behaviours, distorting the ability of people to make free and informed decisions, and generating inaccurate health information^1,8,9^. Furthermore, AI technologies may only be accessible to a subset of a population or be affected by health data poverty (i.e., the diminished ability to benefit from health innovations due to under-representation in health datasets)^10,11^, thereby risking the exacerbation of existing digital and health inequalities^12–14^.

To address these challenges while allowing the benefits of AI to be reaped, a robust governance framework is necessary to facilitate their ethical, secure, and effective implementation in healthcare and population health^3^. The European Union (EU) introduced an ambitious risk framework for AI with the release of the EU AI Act in 2024. This framework is designed to guide the development and usage of AI according to European values and seeks to regulate AI systems across applications and contexts^15–17^. It pertains to AI systems across the health spectrum, aiming to mitigate the potentially harmful effects of AI on the health and well-being of populations^8,12,18,19^, while also allowing health-specific AI technologies to enhance individual and population health outcomes^3,6,20^.

Within the legal framework of the EU, the EU AI Act does not operate in isolation and has to function symbiotically with existing legislation, especially since AI is not a standalone concept within the health domain but embedded in other digital technologies, products, and services. Previous research has partially assessed the existing environment of AI policy and strategy in Europe, though this has exclusively focused on non-binding policies such as strategies and roadmaps^20–22^. An analysis of what EU legislation is applicable to AI in the context of healthcare and population health remains absent, even though such an analysis is urgent in light of the recent legislative developments in the EU, as well as the call of the UN AI Advisory Board for recurring scientific assessments of international and national AI policies to monitor their evolution^2^. In this article, we present the first comprehensive synthesis of binding policies applicable to AI in both healthcare and population health currently in force within the EU, nine member states, and the United Kingdom (UK). Subsequently, we identify potential best practices and highlight potential interactions between the mapped policies and the novel EU AI Act in relation to population health.

## Results

**Fig. 1:**
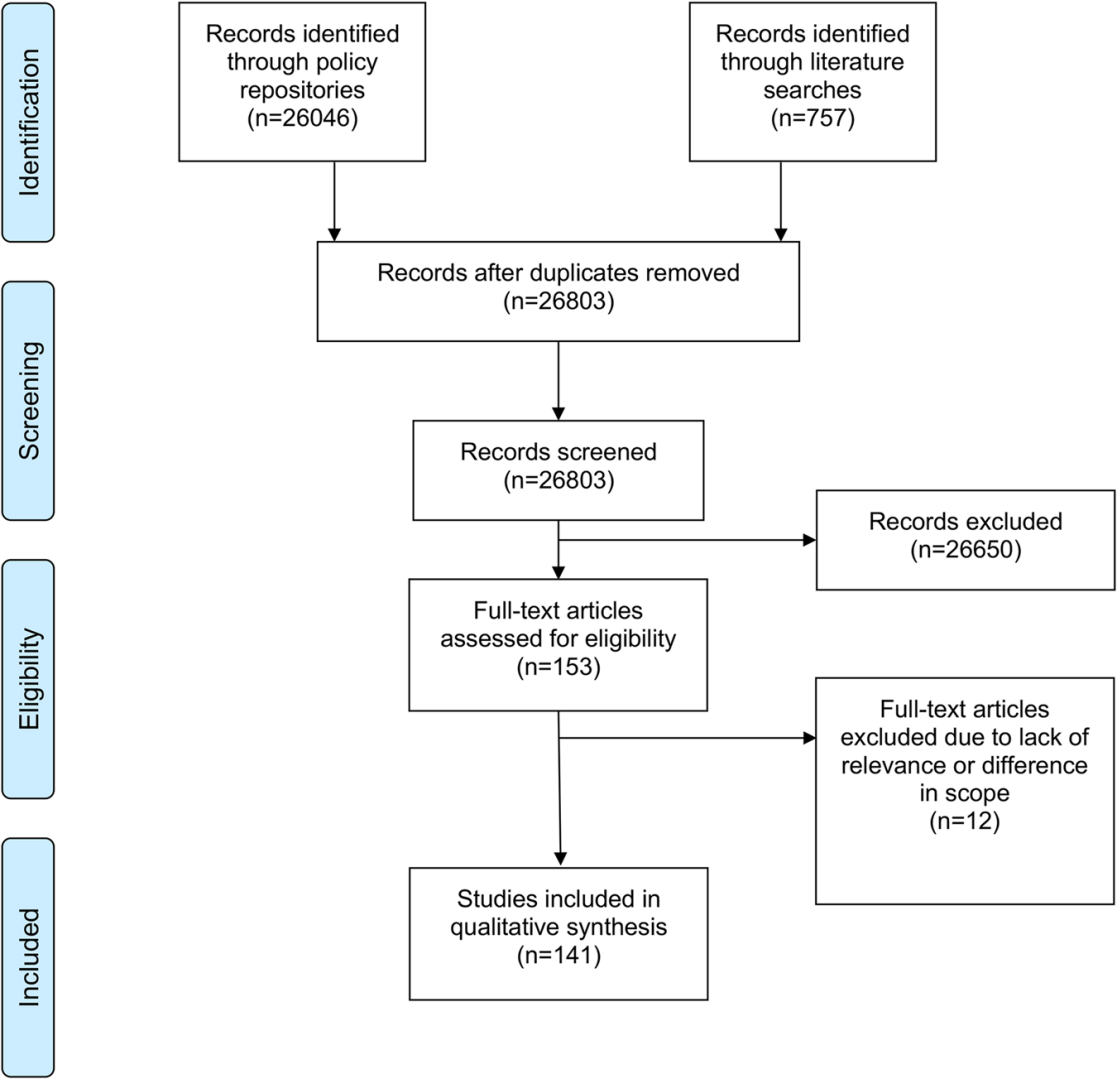
A PRISMA flowchart outlining the data collection process. The provided figure shows a PRISMA flowchart depicting a systematic overview of the identification, screening, eligibility, and inclusion processes used to determine the final set of studies included in the qualitative synthesis. Source: flowchart is adapted from Moher et al.^70^, which is an open-access article distributed under the terms of the Creative Commons Attribution License (CC-BY), which permits unrestricted use, distribution, and reproduction in any medium, provided the original author and source are credited^70^.

**Fig. 2:**
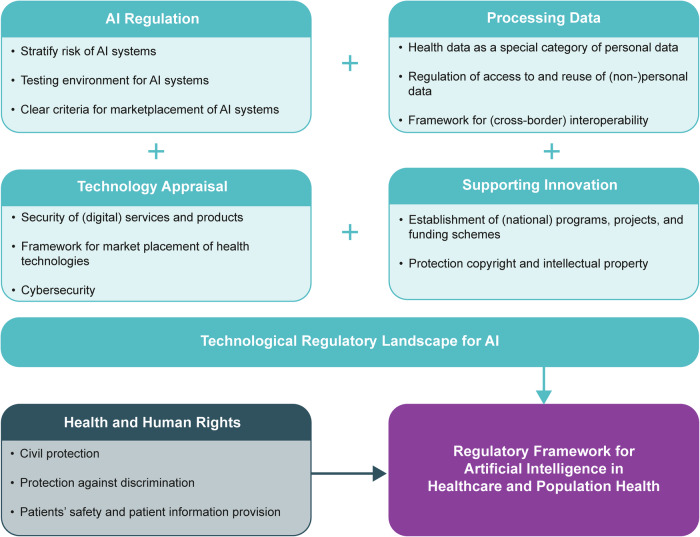
Regulatory framework for artificial intelligence in healthcare and population health. The regulatory framework for artificial intelligence in healthcare and population health consists two parts: the technological part and the health and human rights part. The key components of each of the four dimensions in the the technological regulatory landscape for artificial intelligence (AI) in healthcare and population health are outlined as AI Regulation, Processing Data, Technology Appraisal, and Supporting Innovation. The health and human rights part complements the technological regulatory landscape to ensure that the total regulatory framework encompasses the specific needs, norms, and values of the health domain. Source: authors’ own creation.

We identified 26,046 policy records (2976 for EU, 3161 for Belgium, 368 for Estonia, 2947 for France, 1084 for Germany, 3466 for Italy, 205 for Malta, 418 for Poland, 9421 for Portugal, 1364 for Sweden, and 636 for the UK). Additionally, 757 academic records were identified through scientific and grey literature searches (457 through PubMed; 300 through Google Scholar). The final number of sources included in the qualitative synthesis was 141. The PRISMA flowchart (see Fig. 1) shows the details of the search strategy for this policy mapping. Figure 2 shows the high-level details of how the AI regulatory framework is currently composed based on the final clustering of five categories: 1) AI regulation, 2) processing data, 3) technology appraisal, 4) supporting innovation and 5) health & human rights. An overview of country-specific details are included in Supplementary Tables 3 and 4. It is important to keep in mind that the EU legislation applies to all studied countries, except for the UK for legislation published after its departure from the EU. Thus, when there is no national law in place, European law is directly applicable without supplementary national law: regulations apply directly across the EU upon ratification, whereas Directives still need to be transposed into national law.

### AI regulation

Any AI system being placed on the EU market falls under the scope of the novel EU AI Act. It aims to ensure the ethical development of AI in Europe and beyond its borders while protecting health, safety, and fundamental rights. In the AI Act, specific requirements and obligations for high-risk AI systems are listed, such as a risk management system, draw-up of technical documentation, post-market monitoring, and the need to be developed in such a way that its operations are sufficiently transparent. The design and development of high-risk AI systems should be in such a way that natural persons can oversee their functioning. For the development of future AI systems, the EU AI Act provides the possibility to establish AI regulatory sandboxes at national level as well as the testing in real-world setting prior to market placement. The testing of high-risk AI systems in real-world conditions is also allowed for certain types of AI systems. High-risk AI systems finally need to undergo third-party conformity assessment to verify their performance and safety before market placement. No national binding policies were identified.

### Data processing

AI relies on data for its development and use, making rules concerning data protection, access, and (re)use fundamental. The European legal framework for personal data protection is embedded in the General Data Protection Regulation (GDPR). The purpose of the GDPR is to “protect fundamental rights and freedoms of natural persons and in particular their right to the protection of personal data” while enabling the free movement of such data. This duality means that for personal data to move freely, different legal requirements must be in place. The GDPR binds all EU member states and governs data transfer and handling in non-EU countries. Every country that receives or processes data from the EU needs to demonstrate compliance with the standards of the GDPR. As this also applies to countries such as the United States of America, China, and Australia, the GDPR can be viewed as quite influential in the promotion of personal data collection and processing at a global scale^23^.

At the national level, additional data protection policies were identified in eight of the ten included countries. To protect the processing of personal data, Belgium, Estonia, Germany, Malta, Poland, and the UK adopted the GDPR or modified their current legal base to complement the GDPR (France). The GDPR enables the member states to implement specifications on certain rules set out in the GDPR, resulting in some minor differences between the data protection laws. Relevant differences for this paper are the requirements of processing for public interest (Article 6 GDPR), of processing in the context of employment (Article 88 GDPR), of the age for when a child can give consent themselves (Article 8 GDPR), as well as processing of special categories of personal data (including health) (Article 9 GDPR). For example, Estonia, France, Germany, Malta, and Poland give specific rules, which need to be satisfied in order to process personal data on a public interest basis. Furthermore, France, Germany, and Malta passed additional data policies for health data specifically. In Germany, the context of this additional protection is the processing of patient data for use within the healthcare sector; in Malta, it involves the processing for insurance reasons as well as the secondary processing of personal data in the health sector; and in France, it is the processing of personal data within the Health Data Platform. France specifically states that the data within this platform can be used for the implementation and evaluation of health and social protection policies, for analysing health insurance expenditure, for health surveillance, monitoring, and security, and for research, studies, evaluation and innovation in health and social care.

Additional regulations apply to the protection of non-personal data. The Open Data Directive (Directive (EU) 2019/1024) intends to “promote the use of open data and stimulate innovation in products and services” by setting rules for the re-use of public sector information. The national transposition of the Open Data Directive was identified for Germany, Malta, Sweden, and the UK. Building on the Open Data Directive is the Data Governance Act, which increases the availability of data and facilitates data sharing by specifying conditions for the re-use of certain protected data held by public sector bodies (including data protected because of intellectual property rights) and providing processes and structures that should facilitate voluntary data sharing. The Data Act, passed in 2023 and applicable from September 2025, fosters fair access to and use of data that has been generated using products or services. It specifies specific requirements for the accessibility and transfer of the generated data. Both of these new Acts are regulations and therefore applicable to all member states.

While the above-mentioned policies specify the protection of data while processing, interoperability standards are setting the framework for making processing possible. The Interoperable Europe Act specifies the framework for cross-border interoperability of public services. In its form as a regulation, it will be applicable to all member states. Furthermore, as health systems can be regarded in most member states as public services, the requirements for interoperability standards will be applicable to all public health systems in the EU member states.

### Technology appraisal

AI systems are often embedded in other technologies, meaning general technology policies could be applicable. Technology policies are not a direct competence of the EU (Article 4 TFEU). However, the competence of the EU includes the proper functioning of the single market, meaning it can set standards for the safety of technological products being placed on the EU market. The General Product Safety Regulation (GPSR), applicable from December 2024, offers a broad-based framework for the safety of products being placed on the EU market, which do not fall within sector-specific safety regulations (e.g. medical devices, food). It defines a product as an item, which is intended for or used by consumers, either as a stand-alone product or interconnected to other products, hence potentially covering AI technology embedded in other products.

**Fig. 3:**
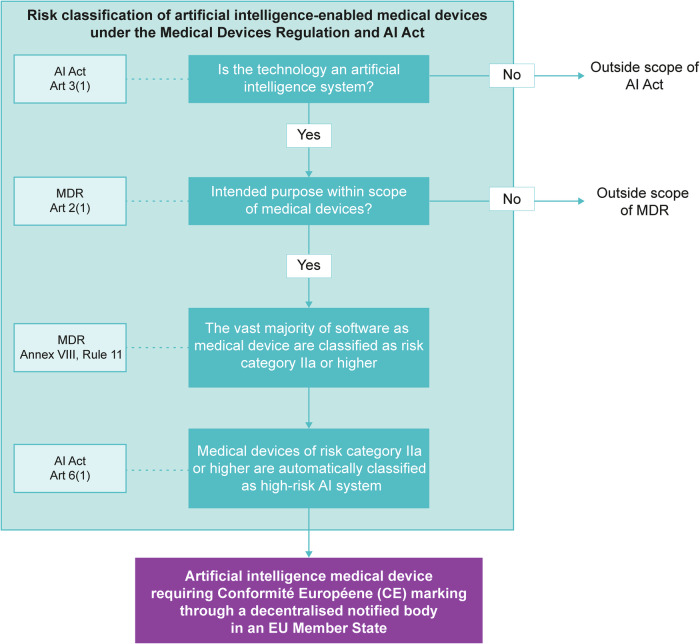
Risk classification process following the EU AI Act and EU Medical Devices Regulation. The figure shows a flowchart for the risk classification of artificial intelligence (AI)-enabled medical devices under the Medical Devices Regulation (MDR) and the AI Act. It begins with the question: “Is the technology an artificial intelligence system?” If the answer is “No,” it falls outside the scope of the AI Act. If “Yes,” it proceeds to check if the intended purpose is within the scope of medical devices. A “No” here means it is outside the scope of MDR. If both answers are “Yes,” the flowchart notes that the vast majority of software as a medical device is classified as risk category IIa or higher. Medical devices with risk class IIa or higher are high-risk artificial intelligence systems. This means that these AI-enabled medical device require Conformité Européene (CE) marking through a decentralized notified body. The boxes positioned on the left side indicate the specific section of the MDR or AI Act that informs that specific step. Source: authors’ own creation.

Products used for health and medical purposes are specifically regulated at the EU level with the Medical Devices Regulation (EU) 2017/745 (MDR) and the In Vitro Diagnostic Medical Devices Regulation (EU) 2017/746 (IVDR). Their purpose is to ensure that each medical device performs consistently with its intended purpose and complies with the general safety and performance requirements. Medical devices can be split into 4 risk classes (I, IIa, IIb, III) with AI medical devices being considered class IIa or higher due to the provision on software as medical devices, which automatically corresponds to them being considered high-risk AI systems under the AI Act (see Fig. 3). Before market placement, medical devices of class IIa and higher need to undergo third-party conformity assessment to provide a sufficient body of clinical evidence regarding the safety and performance of the medical device. Furthermore, Regulation (EU) 2021/2282 seeks to improve the availability of innovative medical technologies by setting an overall framework for the joint clinical assessment of health technologies. All medical devices and in vitro medical devices are also covered within this Regulation. The three regulations are directly applicable in all ten countries. From two of these ten countries (Malta and Sweden) updated national regulations were identified.

With the growing social and economic importance of the internet, policies for the digital sector have been enacted. One area is the protection of the Union against cyber-attacks by passing cybersecurity policies: Directive (EU) 2022/2555, which member states need to incorporate in their national law until October 2024, and the Cybersecurity Act Regulation (EU) 2019/881, which establishes the ENISA and a framework for voluntary European cybersecurity certification schemes. From five of the ten countries (France, Germany, Sweden, Portugal, and the UK) national law on cybersecurity was identified.

The two most recent EU policies for the digital sector are the Digital Markets Act (DMA) and the Digital Services Act (DSA). The DMA focuses on ensuring fair and open digital markets by setting rules for large and impactful online platforms and services (e.g. online search engines, online social networking services, web browsers, and virtual assistants). The DSA addresses online intermediaries (e.g. online marketplaces, social networks) and directs them, amongst others, to include information on measures and tools used for content moderation, including algorithm decision-making. Both regulations are binding for all EU member states.

### Supporting innovation

The establishment of innovation-friendly environments is embedded in the TFEU, stating that the EU should “have the objective of strengthening its scientific and technological bases”. Several binding decisions established different funding programs, such as Horizon Europe, which have AI as one of their priority areas. Complementary to the EU programs, there are national projects or programs, which aim to foster (health) innovation. In Germany, health insurance companies may promote the development of digital innovations “to improve the quality and cost-effectiveness of care.” The focus of Italy’s projects (the highly specialised competence centres and the ITS Academy) lies on training and capability building and funding. In Malta, important legislative steps were taken with the establishment of the Digital Innovation Authority. In Portugal, Decree-Law No. °67/2021 of July 30 sets the conditions for creating technological free zones (TFZs). TFZs are test sites which “intends to test new policy concepts, forms of governance, financing systems and social innovations”. Though not specifically geared towards AI, such settings allow for the testing of a broad range of potentially disruptive innovations. Notably hereby is that “The tests must not call into question the safety of people, animals and property, and must properly safeguard health and environmental risks in compliance with applicable legislation;”.

The EU provides regulations for the legal protection of copyrights and related rights in the information society and the digital market for enhancing the development of innovation. In particular, the 2019 amendment of Directive 2001/29/EC on the harmonisation of certain aspects of copyright and related rights in the information society stipulates in Article 1 how computer programs can fall under the protection of intellectual property legislation, while Article 2 highlights that computer programs are not among the eligible parties whose outputs can receive protection under intellectual property legislation. This is particularly relevant in the age of generative AI, where the AI program itself might fall under the copyright umbrella, but the outputs of that generative AI might not.

### Health & human rights

Compared to the other policy domains, health and human rights policies influence AI by establishing standards that should benefit society as a whole and contribute to the well-being of individuals. An important standard on the EU level is the European Convention on Human Rights (last amended in 2021). The convention states, amongst others, the importance of the right to private life and correspondence, which can only be restricted “in the interest of national security, public safety or the economic well-being of the country (…) for the protection of health or morals”. All ten countries in our study signed this Convention, and are therefore bound to it.

In 2021, Portugal passed its “Portuguese Chapter of Human Rights in the Digital Age” law, which states that “The use of artificial intelligence should be guided by respect for fundamental rights, ensuring a fair balance between the principles of explainability, security, transparency, and responsibility, which meets the circumstances of each specific case and establishes processes aimed at avoiding any prejudice and forms of discrimination”. In Sweden, both the “Patient Data Law” and the “Discrimination Act” are central parts of Swedish legislation, but neither explicitly mention AI. Furthermore, the Swedish “Patient Law” specifies that the patient needs to receive information about the proposed treatment. It remains unclear to what extent this is applicable when using AI-enabled medical devices for treatment support. Moreover, to what extent the patient should be informed on and how much information can be provided about the AI technologies embedded in medical systems or devices remains ambiguous. France, Italy, and Malta have specific bodies to uphold health and human rights standards in line with their national legislation. However, the scope of their responsibilities is broad, leaving it undefined whether it captures AI.

## Discussion

At the time of writing, this study represents the first comprehensive mapping of the regulatory environment of AI in healthcare and population health in Europe. While the EU AI Act plays an important role in the overall regulatory framework for AI, it does not regulate AI by itself. Other relevant AI legislations identified in this study set normative, ethical, and human rights standards, address the safety of technologies, the implementation of innovation, and the protection and use of data. Taken together, they have already formed a baseline regulatory framework for AI in healthcare and population health. These findings align with previous research highlighting how AI is regulated by more than AI-specific legislation^2^.

Health is established as a basic human right within the International Covenant on Economic, Social, and Cultural Rights^24^. This recognition of the importance of protecting human rights when applying AI technologies is being developed at European scale by the Council of Europe through the Convention on Artificial Intelligence, Human Rights, Democracy and the Rule of Law. This Convention aims to ensure that the design, development and application of AI systems is fully consistent with respect for human rights and may become the first legally binding international treaty (to all its signatories) specifically geared towards AI^25^. Portuguese policy also underscores the importance of protecting fundamental rights when using AI. Some countries have operationalised this by setting up dedicated bodies to uphold health and human rights standards. Moving forward, these bodies could oversee the implementation of AI in health legislation by regularly assessing the safety, efficacy, and fairness of AI technologies and updating regulations accordingly. Furthermore, to ensure accountability and foster trust among stakeholders, specific mechanisms for auditing and validating AI systems used in healthcare and public health could be established. An example of a certification scheme in practice is in Malta which assesses innovations on qualities, features, attributes, and behaviours.

Consumer vulnerability is increasing in the digital age^9,12,26^, in part due to a rise of personalised dark commercial patterns^9^. These refer to various practices used in online interfaces that lead users to make choices that are not always in their best interest^8,9^. The European regulations pertaining to digital platforms can play a crucial role in the protection of people’s health and well-being. The DSA prohibits the design, organisation or operation of online interfaces in a way that could deceive or manipulate the user. In addition, the DMAs states that online platforms and services should not use any behavioural techniques or interface designs. The current definition of high-risk AI applications within the EU AI Act does not cover AI systems used in e-commerce or search engines, meaning that machine learning used in large online search engines or platforms is not subject to strict rules of the AI Act^27^. The DMA may complement the EU AI Act, as it seeks to ensure fair competition between digital platforms and creates rules for platform actors. Online platforms and services are not allowed to process personal data of end-users for providing online advertising services, combine or cross-use personal data from different services/platforms without end-user consent. This could limit the use of consumer data (e.g. for building algorithms) and diminish their dominating role on the market. For the DMA and DSA to operate on a national level, the member states should proceed with their transposition. The current regulatory framework focuses strongly on the development of AI, while provisions on navigating liability in relation to AI systems causing harm remain absent so far. The proposed AI Liability Directive and Product Liability Directive aim to introduce new fault- and no fault-based liability provisions for AI systems and products containing AI systems^28,29^. Under these proposed legislations, the manufacturer of the AI system as well as the providers of digital products and devices containing AI systems would be able to be held accountable for damage done by defective AI systems or products containing those systems.

Data preparation and collection for the training of AI systems is being regulated through different legislation on data governance and processing at both the EU and national levels: the GDPR for the use of personal data, the “Open Data Act” for the use of non-personal data, and intellectual property and copyright laws. Health data is classified as a special category under the GDPR and processing of these special categories can be done without consent of the data subject for reasons of public interest in the areas of public health, theoretically allowing AI technologies to be used for this purpose. However, a clear definition of ‘public interest’ is direly needed. The “Open Data Directive” establishes the possibility to use public documents as well as research data from publicly funded projects. Intellectual property laws could hinder access to training data and AI technologies, posing challenges for collaborative innovation ecosystems in developing and speeding up the introduction of novel AI-enabled health technologies. This is particularly problematic considering the significant potential of AI in healthcare and population health^3,6^, which necessitates open collaboration to fully realise its benefits, avoiding future inequalities in health and improving access to healthcare. Simultaneously, existing copyright laws prohibit the use of any protected work for commercial purposes unless approved by the rightsholder, though there is the exclusion for text and data mining of works for scientific research purposes, which could include AI technologies. Moreover, different Creative Commons licences grant permissions, including for commercial use, as long as credit is given to the creator, which could pose challenges for (non-)commercial use of AI in the health domain due to the limited traceability of content in AI output. Finally, the Regulation for a European Health Data Space should lead to more health data becoming findable and accessible for large-scale research^30^, including the application of privacy-preserving methodologies such as federated learning^31,32^. In the proposed regulation, Article 34 states that health data can be processed for ‘training, testing and evaluating of algorithms, including in medical devices, AI systems and digital health applications’. Taken together, a regulatory framework providing usable data for the lawful development of AI technologies hinges on the clarity of its purpose definition.

With respect to the outputs of generative AI technologies, the question arises as to whether these can be covered under extant innovation policies, especially intellectual property laws. The copyright laws mapped in this study acknowledge only outputs created by natural persons or legal entities can be protected under copyright law, meaning outputs created by a non-human entity are not protected. Furthermore, the work needs to be the author’s own intellectual creation to qualify for copyright protection in the EU. Whereas copyright licenses protects works by giving the owner exclusive rights, open-source licenses (e.g., BSD licenses, GNU General Public Licenses, or copyleft licences) promote the use and modification of works^33^. Licensing health datasets and AI models with open-source licenses in the context of the AI innovation landscape would facilitate and improve access, though using it as AI training data raises the question if it could lead to AI-generated outputs that are considered derivative works, which can affect the conditions under which the derivate can or has to be distributed^33^.

Innovation policy can provide a strong breeding ground for AI technologies in health^34^. In the EU AI Act, regulatory sandboxes are proposed that are intended to provide a controlled environment for the development, testing, and validation of innovative AI systems^15,16^, including AI technologies in medical devices. An operational example of such regulatory sandboxes already exists in Portugal with the TFZs. Though not specifically geared towards AI, such settings allow for the testing of a broad range of potentially disruptive innovations. That said, whether the testing in these regulatory sandboxes is sufficient to acquire sufficient evidence for health-related AI technologies needs to be further investigated.

Furthermore, this updated regulatory framework starkly contrasts with the regulatory framework of the USA, which is streamlined at the national level by the Food and Drugs Administration. In the USA, no specific clearance pathway for AI-based medical devices exists but they are legally assimilated to Software as Medical Devices^35^. The FDA offers three centralized market access pathways for medical devices based on the risks of the devices: approval through the premarket approval pathway (most stringent review for high-risk devices), authorisation via the de novo pre-market review (for low and moderate-risk devices), and clearance via the 510(k) pathway (for devices substantially equivalent to one or more legally marketed devices)^36,37^. Clinical evaluations are not a requirement under the 510(k) pathway, resulting in the ability for AI medical devices to enter the USA healthcare market without clinical testing as long as a similar device has previously been granted market access^38^. That said, when clinical evaluations are required as part of the most stringent pre-market approval process, these can be performed without having to disentangle a complex regulatory framework. While this framework is potentially easier to navigate due to its streamlined nature, it requires medical devices to be reauthorised after each update that changes the underlying performance of a device, its safety characteristics, or the intended population for whom the device is intended to be used. For AI systems capable of continuous learning, this would mean they would be subject to continual reauthorisations every time their performance changes in response to new data^39^, risking commercial unviability.

Some limitations need to be considered. The selection of the countries is based on convenience sampling, meaning the findings need to be cautiously applied to countries outside the scope of this study. Nonetheless, country selection included a variety of Western and Eastern European countries, as well as small and large-sized markets, health systems, and political economies of AI in health. Given our focus on binding policies, we did not capture how the use of AI is integrated into clinical guidelines or recommendations, which should be explored in future research. The quality of the included records was not assessed. However, as the aim of this study was not to validate methodological rigour to ascertain confidence in the data synthesis but rather to collect information about regulatory frameworks in different countries, the absence of a quality assessment does not compromise the validity of this study. The possibility of errors in translation or misinterpretation cannot be dismissed, though country experts were involved to assess the completeness and correctness of the findings and assist with interpreting the mapped legislation for the studied countries. Finally, we acknowledge the possibility that a policy published prior to 2014 was overlooked in case it had not been amended in the last ten years, though the involvement of country experts to validate our policy data minimised this possibility.

Ultimately, the field of AI is rapidly developing and subject to change, while regulations specifically pertaining to AI technologies in healthcare and population health are still nascent and scarce. A combination of data, technology, innovation, and health and human rights policy has already formed a baseline regulatory framework for AI in health. Future work should more extensively explore potential interactions and missed aspects between existing legislation and regulation specifically pertaining to AI (e.g., with respect to medical devices, data protection, and data enablement), as well as monitor the development of the AI regulatory landscape over time. The development of a strong regulatory environment for AI is a prerequisite to extracting the benefits while curtailing the risks to individual and population health.

## Methods

This article followed a validated policy mapping framework methodology^40^, which is based on the foundational framework of a scoping review that is adapted to allow for the systematic screening of policy repositories rather than academic databases^41,42^. It has been previously applied to map policies and strategies in a number of disciplines, such as education^43,44^, employment^45^, digital health^46^, and substance abuse^47^. The findings were reported using the PRISMA-ScR (Preferred Reporting Items for Systematic Reviews and Meta-Analyses extension for Scoping Reviews; Supplementary Table 1) framework and analysed with the methodology of a qualitative document analysis^48,49^.

### Eligibility criteria

The ten countries included provide an overview of a diverse set of AI-related policy initiatives across Europe, namely Belgium, Estonia, France, Germany, Italy, Malta, Poland, Portugal, Sweden and the UK. In addition, EU legislation was included due to its direct influence on the national policies of EU Member States. The selection of countries was based on convenience sampling^50^, though they represent a mix of different political and health systems, as well as country sizes and levels of digital maturity^51,52^.

Eligible records had to be drafted by government institutions and be binding in nature, meaning they have a legally binding force for the actors covered by them. Rules and laws included in a binding framework must be followed, and non-compliance can result in legal penalties. Opinions, strategies, and guidance documents were not considered for inclusion, as these constitute non-binding policies, are solely indicative, and have been assessed previously^21,22,53^. In countries with federated governance structures (e.g., Belgium and Germany), subnational policies were not included, despite federal states having the legal mandate to enact policies with an indirect link to AI (education sector, labor law). In these countries, national law tends to set the overall framework that is of primary interest for this article^54^.

Eligible policies had to be published in the last 10 years (Jan 2014–March 2024). We considered this period appropriate because the current exponential growth of AI began in the last decade in conjunction with the availability of data and new computing power^55^. With the launch of the European Initiative of AI in 2018^56^, 2014 represents a viable starting point to capture already existing policies before the launch of the EU initiative. If a relevant policy was originally published prior to 2014 but had received amendments within our eligible time frame, the entire consolidated policy was considered for inclusion. As this article focused on understanding the legislative framework currently in effect, it was sufficient to include the latest version of a policy that is in effect rather than each previous iteration. Finally, we recognise that AI is not a standalone concept within the health domain but is embedded in other digital technologies, products, and services^57^. Nearly all such technologies rely on large volumes of data, which form the basis for training, testing, and validation of AI technologies^3^. Therefore, we also included legislation that captured the broader policy domains relating to AI, namely data and innovation policy.

### Data collection

Following the methods used in other policy mapping relating to public health^43,58^, the data collection consisted of five steps. As a first step, national and European policy repositories were searched as our primary source for data collection (Supplementary Table 2). For developing search strings for policy repositories, key terms were identified and based on these search terms. In this paper, the key terms for AI and its broader framework were grouped under “AI”, “innovation” and “data”. For healthcare and population health, we reviewed the OECD AI principles and determined “health” and “human factors” as practical key terms to also cover ethical considerations of the use of AI technology. Further keywords were identified through high-level policy documents and previous research pertaining to AI, data, innovation, and health and human rights policy^24,59–66^. The full overview of keywords is presented in Table 1. Prior to the search, all keywords were translated into the different local languages. In case the combination of the search terms yielded insufficient or no results, the key terms were used separately. Data collection was performed by four authors (JS, NMS, SB, and RVK). Countries were distributed among the authors based on language proficiency and knowledge of the policy environment. To ensure consistency among the authors, extracted policy data were reviewed in group discussions during weekly meetings. As a second step, to identify overarching political reforms or trends, supplementary searches were performed in PubMed and Google Scholar, the latter only screening the first 300 hits as per previous methodological guidance^67^. The build-up of the search string for the supplemental scientific literature search is shown in Supplementary Table 2. Due to its supplementary nature, it only includes the terms for AI and the countries, both adopted to fit the scientific database. The search was performed by one author (RVK) and findings were discussed with the others. As a third step, policy and academic publications were merged and checked if they conform with the eligibility criteria. The fourth step involved checking reference lists for additional policies that may have not been identified within the search. Lastly, all the policies identified were unified into one single table and the country, the policy name, the year of enactment, if available the last year of modification, and relevant paragraphs of the included policies were collected. The data was collected between 16 January and 6 February 2024. An updated search was conducted on 14 March 2024, after the official adoption of the EU AI Act. Records after this timeframe were added as a result of consulting country experts.

**Table 1:** Keywords used for searches in the policy repositories

AI	Data	Innovation	AND	Health	Human factors
Artificial Intelligence	Data governance	Digital*		Health	Social determinants
Machine learning	Data science	Intellectual property		Well-being	Safety
Deep learning	Data space	Innovation		Public Interest	Privacy
Algorithm	Information governance	Emerging technolog*		Patient	Trust
		Disruptive Technolog*			

The wildcard symbol ‘*’ was used to broaden the search results and allow for variations of keywords to be included.

### Data analysis

Included policies were analysed through a qualitative document analysis in order to extract passages relevant to the regulation of AI^49^. To identify and map recurring themes in the policies, a deductive content analysis was conducted^68,69^. Salient themes were extracted by one author (JS) and post hoc clustered in the five domains. The clustering was reviewed and verified by two authors (NMS and RVK). Finally, individual country information was tabulated per category, and cross-countries differences were narratively synthesised.

### Supplementary information

Supplementary Information

## Data Availability

All policy documents used in this study are publicly available from the respective national policy repositories. No new datasets were generated during this study. Due to all data being publicly available and already in force in the respective studied countries, there was no situation in which it was necessary to request consent.

## References

[1] World Health Organization. *Regulatory Considerations on Artificial Intelligence,* https://iris.who.int/bitstream/handle/10665/373421/9789240078871-eng.pdf?sequence=1&isAllowed=y (2023).

[2] UN Secretary-General’s AI Advisory Body. *Interim Report: Governing AI for Humanity,* https://www.un.org/sites/un2.un.org/files/ai_advisory_body_interim_report.pdf (2023).

[3] Morley, J., Murphy, L., Mishra, A., Joshi, I. & Karpathakis, K. Governing Data and Artificial Intelligence for Health Care: Developing an International Understanding. *JMIR Form. Res.* **6**, e31623 (2022). 35099403 10.2196/31623 PMC8844981

[4] Bauer, G. R. & Lizotte, D. J. Artificial Intelligence, Intersectionality, and the Future of Public Health. *Am. J. Public Health* **111**, 98–100 (2021). 33326280 10.2105/AJPH.2020.306006 PMC7750598

[5] Bompelli, A. et al. Social and Behavioral Determinants of Health in the Era of Artificial Intelligence with Electronic Health Records: A Scoping Review. *Health Data Sci.* **2021**, 9759016 (2021). 38487504 10.34133/2021/9759016 PMC10880156

[6] Lavigne, M., Mussa, F., Creatore, M. I., Hoffman, S. J. & Buckeridge, D. L. A population health perspective on artificial intelligence. *Health. Manag. Forum* **32**, 173–177 (2019). 10.1177/0840470419848428 PMC7323781 31106580

[7] Guevara, M. et al. Large language models to identify social determinants of health in electronic health records. *npj Digit. Med.* **7**, 1–14 (2024). 38200151 10.1038/s41746-023-00970-0 PMC10781957

[8] Holly, L. et al. Optimising adolescent wellbeing in a digital age. *BMJ* **380**, e068279 (2023). 36940933 10.1136/bmj-2021-068279 PMC10019455

[9] OECD. *Dark Commercial Patterns*. https://www.oecd.org/digital/dark-commercial-patterns-44f5e846-en.htm (2022).

[10] Ibrahim, H., Liu, X., Zariffa, N., Morris, A. D. & Denniston, A. K. Health data poverty: an assailable barrier to equitable digital health care. *Lancet Digital Health* **3**, e260–e265 (2021). 33678589 10.1016/S2589-7500(20)30317-4

[11] The Lancet Global Health. Advancing equity in medical device performance. *Lancet Glob Health* **12**, e712 (2024). 38614618 10.1016/S2214-109X(24)00141-4

[12] OECD. *AI in Health: Huge Potential, Huge Risk*, https://www.oecd.org/health/AI-in-health-huge-potential-huge-risks.pdf (2024).

[13] Vyas, D. A., Eisenstein, L. G. & Jones, D. S. Hidden in Plain Sight — Reconsidering the Use of Race Correction in Clinical Algorithms. *N. Engl. J. Med.* **383**, 874–882 (2020). 32853499 10.1056/NEJMms2004740

[14] van Kessel, R. et al. Digital Health Paradox: International Policy Perspectives to Address Increased Health Inequalities for People Living With Disabilities. *J. Med Internet Res.* **24**, e33819 (2022). 35191848 10.2196/33819 PMC8905475

[15] Gasser, U. & An, E. U. landmark for AI governance. *Science* **380**, 1203–1203 (2023). 37319234 10.1126/science.adj1627

[16] Prainsack, B. & Forgó, N. New AI regulation in the EU seeks to reduce risk without assessing public benefit. *Nat. Med.* 1–3, 10.1038/s41591-024-02874-2 (2024). 10.1038/s41591-024-02874-2 38499661

[17] Busch, F. et al. Navigating the European Union Artificial Intelligence Act for Healthcare. *npj Digit. Med.* **7**, 210 (2024). 39134637 10.1038/s41746-024-01213-6 PMC11319791

[18] Gilmore, A. B. et al. Defining and conceptualising the commercial determinants of health. *Lancet* **401**, 1194–1213 (2023). 36966782 10.1016/S0140-6736(23)00013-2

[19] Horton, R. Offline A mirage of progress for health and democracy. *Lancet* **403**, 1432 (2024). 38614474 10.1016/S0140-6736(24)00739-6

[20] Cohen, I. G., Evgeniou, T., Gerke, S. & Minssen, T. The European artificial intelligence strategy: implications and challenges for digital health. *Lancet Digital Health* **2**, e376–e379 (2020). 33328096 10.1016/S2589-7500(20)30112-6

[21] Roche, C., Wall, P. J. & Lewis, D. Ethics and diversity in artificial intelligence policies, strategies and initiatives. *AI Ethics* 1–21, 10.1007/s43681-022-00218-9 (2022). 10.1007/s43681-022-00218-9 PMC9540088 36246014

[22] van Berkel, N., Papachristos, E., Giachanou, A., Hosio, S. & Skov, M. B. A Systematic Assessment of National Artificial Intelligence Policies: Perspectives from the Nordics and Beyond. In *Proceedings of the 11th Nordic Conference on Human-Computer Interaction: Shaping Experiences, Shaping Society* 1–12 (Association for Computing Machinery, 2020). 10.1145/3419249.3420106.

[23] Lalova-Spinks, T. et al. EU-US data transfers: an enduring challenge for health research collaborations. *npj Digit. Med.* **7**, 215 (2024). 39152232 10.1038/s41746-024-01205-6 PMC11329736

[24] United Nations General Assembly. *International Covenant on Economic, Social and Cultural Rights*. (United Nations General Assembly, 1966).

[25] van Kolfschooten, H. & Shachar, C. The Council of Europe’s AI Convention (2023-2024): Promises and pitfalls for health protection. *Health Policy* **138**, 104935 (2023). 37925880 10.1016/j.healthpol.2023.104935

[26] OECD. *Consumer Vulnerability in the Digital Age*, https://www.oecd.org/publications/consumer-vulnerability-in-the-digital-age-4d013cc5-en.htm (2023).

[27] Hacker, P., Cordes, J. & Rochon, J. Regulating Gatekeeper Artificial Intelligence and Data: Transparency, Access and Fairness under the Digital Markets Act, the General Data Protection Regulation and Beyond. *Eur. J. Risk Regulation* 1–38, 10.1017/err.2023.81 (2023).

[28] European Commission. *Proposal for a Directive of the European Parliament and of the Council on Liability for Defective Products*. https://eur-lex.europa.eu/legal-content/EN/TXT/?uri=CELEX%3A52022PC0495 (2022).

[29] European Commission. *Proposal for an AI Liability Directive,* https://eur-lex.europa.eu/legal-content/EN/TXT/?uri=CELEX%3A52022PC0496 (2022).

[30] European Commission. *Proposal for a Regulation of the European Parliament and of the Council on the European Health Data Space*. https://eur-lex.europa.eu/legal-content/EN/TXT/?uri=celex%3A52022PC0197 (2022).

[31] Rieke, N. et al. The future of digital health with federated learning. *npj Digit. Med.* **3**, 1–7 (2020). 33015372 10.1038/s41746-020-00323-1 PMC7490367

[32] Sinaci, A. A. et al. Privacy-preserving federated machine learning on FAIR health data: A real-world application. *Comput. Struct. Biotechnol. J.* **24**, 136–145 (2024). 38434250 10.1016/j.csbj.2024.02.014 PMC10904920

[33] Schmit, C. D., Doerr, M. J. & Wagner, J. K. Leveraging IP for AI governance. *Science* **379**, 646–648 (2023). 36795826 10.1126/science.add2202

[34] van Kessel, R. et al. Mapping Factors That Affect the Uptake of Digital Therapeutics Within Health Systems: Scoping Review. *J. Med. Internet Res.* **25**, e48000 (2023). 37490322 10.2196/48000 PMC10410406

[35] Coder, M., McBride, L. & McClenahan, S. Core elements of national policy for digital health technology evidence and access. *npj Digit. Med.* **7**, 212 (2024). 39138261 10.1038/s41746-024-01209-2 PMC11322556

[36] Muehlematter, U. J., Daniore, P. & Vokinger, K. N. Approval of artificial intelligence and machine learning-based medical devices in the USA and Europe (2015–20): a comparative analysis. *Lancet Digital Health* **3**, e195–e203 (2021). 33478929 10.1016/S2589-7500(20)30292-2

[37] Muehlematter, U. J., Bluethgen, C. & Vokinger, K. N. FDA-cleared artificial intelligence and machine learning-based medical devices and their 510(k) predicate networks. *Lancet Digital Health* **5**, e618–e626 (2023). 37625896 10.1016/S2589-7500(23)00126-7

[38] Hwang, T. J., Kesselheim, A. S. & Vokinger, K. N. Lifecycle Regulation of Artificial Intelligence– and Machine Learning–Based Software Devices in Medicine. *JAMA* **322**, 2285–2286 (2019). 31755907 10.1001/jama.2019.16842

[39] Allison, K. & Gilbert, S. *Regulating Artificial Intelligence: Lessons from Medical Devices,* https://fsi9-prod.s3.us-west-1.amazonaws.com/s3fs-public/2023-11/2023-11-01_-_allison_gilbert-_regulating_ai_lessons_from_medical_devices.pdf (2023).

[40] van Kessel, R., Czabanowska, K. & Roman-Urrestarazu, A. Systematically mapping and analysing multi-level policy developments: a methodological toolkit. Preprint at 10.21203/rs.3.rs-3788502/v1 (2023).

[41] Arksey, H. & O’Malley, L. Scoping studies: towards a methodological framework. *Int. J. Soc. Res. Methodol.* **8**, 19–32 (2005). 10.1080/1364557032000119616

[42] Levac, D., Colquhoun, H. & O’Brien, K. K. Scoping studies: advancing the methodology. *Implement. Sci.* **5**, 69 (2010). 20854677 10.1186/1748-5908-5-69 PMC2954944

[43] van Kessel, R. et al. Autism and education—international policy in small EU states: policy mapping in Malta, Cyprus, Luxembourg and Slovenia. *Eur. J. Public Health* **30**, 1078–1083 (2020). 32879964 10.1093/eurpub/ckaa146 PMC7733051

[44] van Kessel, R. et al. Education, Special Needs, and Autism in the Baltic States: Policy Mapping in Estonia, Latvia, and Lithuania. *Front. Educ.* **5**, 161 (2020). 10.3389/feduc.2020.00161

[45] Bunt, D. et al. Quotas, and Anti-discrimination Policies Relating to Autism in the EU: Scoping Review and Policy Mapping in Germany, France, Netherlands, United Kingdom, Slovakia, Poland, and Romania. *Autism Res.* **13**, 1397–1417 (2020). 32441457 10.1002/aur.2315 PMC7496597

[46] van Kessel, R. et al. Digital Health Reimbursement Strategies of 8 European Countries and Israel: Scoping Review and Policy Mapping. *JMIR mHealth uHealth* **11**, e49003 (2023). 37773610 10.2196/49003 PMC10576236

[47] Neicun, J. et al. Mapping novel psychoactive substances policy in the EU: The case of Portugal, the Netherlands, Czech Republic, Poland, the United Kingdom and Sweden. *PLOS One* **14**, e0218011 (2019). 31242225 10.1371/journal.pone.0218011 PMC6594604

[48] Tricco, A. C. et al. PRISMA Extension for Scoping Reviews (PRISMA-ScR): Checklist and Explanation. *Ann. Intern. Med.* **169**, 467–473 (2018). 30178033 10.7326/M18-0850

[49] Dalglish, S. L., Khalid, H. & McMahon, S. A. Document analysis in health policy research: the READ approach. *Health Policy Plan.* **35**, 1424–1431 (2020). 10.1093/heapol/czaa064 PMC7886435 33175972

[50] Marshall, M. N. Sampling for qualitative research. *Fam. Pract.* **13**, 522–525 (1996). 9023528 10.1093/fampra/13.6.522

[51] Eurostat. Population on 1 January. https://ec.europa.eu/eurostat/databrowser/view/tps00001/default/table?lang=en&category=t_demo.t_demo_pop (2023).

[52] van Kessel, R., Wong, B. L. H., Rubinić, I., O’Nuallain, E. & Czabanowska, K. Is Europe prepared to go digital? making the case for developing digital capacity: An exploratory analysis of Eurostat survey data. *PLOS Digital Health* **1**, e0000013 (2022). 36812527 10.1371/journal.pdig.0000013 PMC9931321

[53] Foffano, F., Scantamburlo, T. & Cortés, A. Investing in AI for social good: an analysis of European national strategies. *AI Soc.* **38**, 479–500 (2023). 35528248 10.1007/s00146-022-01445-8 PMC9068863

[54] Liebig, L., Güttel, L., Jobin, A. & Katzenbach, C. Subnational AI policy: shaping AI in a multi-level governance system. *AI & Soc.*10.1007/s00146-022-01561-5 (2022).

[55] Council of Europe. History of Artificial Intelligence, https://www.coe.int/en/web/artificial-intelligence/history-of-ai (2024).

[56] European Commission. *Communication from the Commission on Artificial Intelligence for Europe*, https://eur-lex.europa.eu/legal-content/EN/TXT/?uri=COM%3A2018%3A237%3AFIN (2018).

[57] Panch, T., Pearson-Stuttard, J., Greaves, F. & Atun, R. Artificial intelligence: opportunities and risks for public health. *Lancet Digital Health* **1**, e13–e14 (2019). 33323236 10.1016/S2589-7500(19)30002-0

[58] van Kessel, R. et al. Autism, Austerity, and the Right to Education in the EU: Policy Mapping and Scoping Review of Ireland, Portugal, Italy, and Greece. *Eur. Policy Anal.* **00**, 1–13 (2021).

[59] World Health Organization. *Ethics and Governance of Artificial Intelligence for Health: Guidance on Large Multi-Modal Models* (WHO, 2023).

[60] European Commission. *Proposal For A Regulation Of The European Parliament And Of The Council Laying Down Harmonised Rules On Artificial Intelligence (Artificial Intelligence Act) And Amending Certain Union Legislative Acts*. https://eur-lex.europa.eu/legal-content/EN/TXT/?uri=celex%3A52021PC0206 (2021).

[61] United Nations General Assembly. *International Covenant on Civil and Political Rights* (United Nations General Assembly, 1966).

[62] European Commission. *Regulation (EU) 2022/868 of the European Parliament and of the Council of 30 May 2022 on European Data Governance and Amending Regulation (EU) 2018/1724 (Data Governance Act)* (European Commission, 2022).

[63] European Commission. *Regulation (EU) 2023/2854 of the European Parliament and of the Council of 13 December 2023 on Harmonised Rules on Fair Access to and Use of Data and Amending Regulation (EU) 2017/2394 and Directive (EU) 2020/1828 (Data Act)* (European Commission 2023).

[64] European Parliament. Innovation policy fact sheet, https://www.europarl.europa.eu/factsheets/en/sheet/67/innovation-policy (2023).

[65] European Commission. *Charter of Fundamental Rights of the European Union*. https://www.europarl.europa.eu/charter/pdf/text_en.pdf (2000).

[66] van Kessel, R., Ranganathan, S., Anderson, M., McMillan, B. & Mossialos, E. Exploring potential drivers of patient engagement with their health data through digital platforms: A scoping review. *Int. J. Med. Informatics* 105513, 10.1016/j.ijmedinf.2024.105513 (2024). 10.1016/j.ijmedinf.2024.105513 38851132

[67] Haddaway, N. R., Collins, A. M., Coughlin, D. & Kirk, S. The Role of Google Scholar in Evidence Reviews and Its Applicability to Grey Literature Searching. *PLOS One* **10**, e0138237 (2015). 26379270 10.1371/journal.pone.0138237 PMC4574933

[68] Braun, V. & Clarke, V. Using thematic analysis in psychology. *Qualitative Res. Psychol.* **3**, 77–101 (2006). 10.1191/1478088706qp063oa

[69] Kyngäs, H. Inductive Content Analysis. In *The Application of Content Analysis in Nursing Science Research* (eds. Kyngäs, H., Mikkonen, K. & Kääriäinen, M.) 13–21 (Springer International Publishing, Cham, 2020). 10.1007/978-3-030-30199-6_2.

[70] Moher, D., Liberati, A., Tetzlaff, J., Altman, D. G. & Group, T. P. Preferred Reporting Items for Systematic Reviews and Meta-Analyses: The PRISMA Statement. *PLOS Med.* **6**, e1000097 (2009). 21603045 10.1371/journal.pmed.1000097 PMC3090117

