# Supplementary material for: Mapping the regulatory landscape for artificial intelligence in health within the European Union
Source: NPJ Digit Med. 2024 Aug 27;7:229. doi: 10.1038/s41746-024-01221-6 (PMC11350181; doi:10.1038/s41746-024-01221-6)
Supplement: Supplementary file 1 — Supplementary Information [file 41746_2024_1221_MOESM1_ESM.pdf]

Supplementary Information

Schmidt, J., Schutte, N.M., Buttigieg, S., Novillo-Ortiz, D., Sutherland, E., Anderson, M., de Witte, B., Peolsson, M., Unim, B., Pavlova, M., Stern, A.D., Mossialos, E., van Kessel, R. Mapping the Regulatory Landscape for Artificial Intelligence in Health within the European Union

Table of contents

Supplementary Table 1. PRISMA-ScR checklist. 3

Supplementary Table 2. The policy repositories used per country........ 5

Supplementary Table 3. Build-up of the search string for PubMed and Google Scholar. 6

Supplementary Table 4. Country-specific details on the regulation of artificial intelligence. ... 7

Supplementary Table 5. Policy-specific details on the regulation of artificial intelligence. 14

Supplementary Table 1. PRISMA-ScR checklist.

SECTION	ITEM	PRISMA-ScR CHECKLIST ITEM	REPORTED ON PAGE #
TITLE			
Title	1	Identify the report as a scoping review.	n/a
ABSTRACT			
Structured summary	2	Provide a structured summary that includes (as applicable): background, objectives, eligibility criteria, sources of evidence, charting methods, results, and conclusions that relate to the review questions and objectives.	2
INTRODUCTION			
Rationale	3	Describe the rationale for the review in the context of what is already known. Explain why the review questions/objectives lend themselves to a scoping review approach.	3
Objectives	4	Provide an explicit statement of the questions and objectives being addressed with reference to their key elements (e.g., population or participants, concepts, and context) or other relevant key elements used to conceptualize the review questions and/or objectives.	3
METHODS			
Protocol and registration	5	Indicate whether a review protocol exists; state if and where it can be accessed (e.g., a Web address); and if available, provide registration information, including the registration number.	n/a
Eligibility criteria	6	Specify characteristics of the sources of evidence used as eligibility criteria (e.g., years considered, language, and publication status), and provide a rationale.	11-12
Information sources*	7	Describe all information sources in the search (e.g., databases with dates of coverage and contact with authors to identify additional sources), as well as the date the most recent search was executed.	12 & Supplementary Table 2
Search	8	Present the full electronic search strategy for at least 1 database, including any limits used, such that it could be repeated.	12 & Supplementary Table 3
Selection of sources of evidence†	9	State the process for selecting sources of evidence (i.e., screening and eligibility) included in the scoping review.	12
Data charting process‡	10	Describe the methods of charting data from the included sources of evidence (e.g., calibrated forms or forms that have been tested by the team before their use, and whether data charting was done independently or in duplicate) and any processes for obtaining and confirming data from investigators.	12
Data items	11	List and define all variables for which data were sought and any assumptions and simplifications made.	n/a

Critical appraisal of individual sources of evidence§	12	If done, provide a rationale for conducting a critical appraisal of included sources of evidence; describe the methods used and how this information was used in any data synthesis (if appropriate).	n/a
Synthesis of results	13	Describe the methods of handling and summarizing the data that were charted.	12
RESULTS			
Selection of sources of evidence	14	Give numbers of sources of evidence screened, assessed for eligibility, and included in the review, with reasons for exclusions at each stage, ideally using a flow diagram.	3-4
Characteristics of sources of evidence	15	For each source of evidence, present characteristics for which data were charted and provide the citations.	Supplementary Tables 4 and 5
Critical appraisal within sources of evidence	16	If done, present data on critical appraisal of included sources of evidence (see item 12).	n/a
Results of individual sources of evidence	17	For each included source of evidence, present the relevant data that were charted that relate to the review questions and objectives.	Supplementary Tables 4 and 5
Synthesis of results	18	Summarize and/or present the charting results as they relate to the review questions and objectives.	3-7
DISCUSSION			
Summary of evidence	19	Summarize the main results (including an overview of concepts, themes, and types of evidence available), link to the review questions and objectives, and consider the relevance to key groups.	7-10
Limitations	20	Discuss the limitations of the scoping review process.	10
Conclusions	21	Provide a general interpretation of the results with respect to the review questions and objectives, as well as potential implications and/or next steps.	10-11
FUNDING			
Funding	22	Describe sources of funding for the included sources of evidence, as well as sources of funding for the scoping review. Describe the role of the funders of the scoping review.	13

Supplementary Table 2. The policy repositories used per country.

Belgium	http://www.ejustice.just.fgov.be/cgi_wet/wet.pl (in dutch)
EU-general	https://eur-lex.europa.eu/homepage.html<br>https://www.coe.int/en/web/portal
Estonia	https://www.riigiteataja.ee/index.html
France	http://www.legifrance.gouv.fr/
Germany	https://www.gesetze-im-internet.de/titelsuche.html
Italy	https://www.normattiva.it/
Malta	https://legislation.mt
Poland	http://isap.sejm.gov.pl/<br>http://dziennikustaw.gov.pl/
Portugal	https://diariodarepublica.pt/dr/home
Sweden	https://beta.lagrummet.se/
United Kingdom	http://www.legislation.gov.uk/

Supplementary Table 3. Build-up of the search string for PubMed and Google Scholar.

Search	Query
#1	"Artificial Intelligence"[MeSH] OR Algorithm[MeSH] OR "Machine Learning"[MeSH] OR "Deep Learning"[MeSH] OR "Expert Systems"[MeSH] OR "Natural Language Processing"[MeSH] OR AI[Title/Abstract] OR "artificial intelligence"[Title/Abstract] OR "Machine Learning"[Title/Abstract] OR "Deep learning"[Title/Abstract] OR "neural network*"[Title/Abstract] OR "supervised learning"[Title/Abstract] OR "unsupervised learning"[Title/Abstract]
#2	Policy[Title/Abstract] OR law[Title/Abstract] OR legislation[Title/Abstract] OR decree[Title/Abstract]
#3	Europe[Title/Abstract] OR "European Union"[Title/Abstract] OR EU[Title/Abstract] OR UK[Title/Abstract] OR "United Kingdom"[Title/Abstract] OR "Engl*"[Title/Abstract] OR "Wales"[Title/Abstract] OR "Scot*"[Title/Abstract] OR "Northern Ir*"[Title/Abstract] OR France[Title/Abstract] OR French[Title/Abstract] OR German*[Title/Abstract] OR Belgi*[Title/Abstract] OR "Malta"[Title/Abstract] OR "Maltese"[Title/Abstract] OR Estonia*[Title/Abstract] OR Swed*[Title/Abstract] OR Portug*[Title/Abstract] OR Poland[Title/Abstract] OR Polish[Title/Abstract] OR Ital*[Title/Abstract]
Final	("Artificial Intelligence"[MeSH] OR Algorithm[MeSH] OR "Machine Learning"[MeSH] OR "Deep Learning"[MeSH] OR "Expert Systems"[MeSH] OR "Natural Language Processing"[MeSH] OR AI[Title/Abstract] OR "artificial intelligence"[Title/Abstract] OR "Machine Learning"[Title/Abstract] OR "Deep learning"[Title/Abstract] OR "neural network*"[Title/Abstract] OR "supervised learning"[Title/Abstract] OR "unsupervised learning"[Title/Abstract]) AND (Policy[Title/Abstract] OR law[Title/Abstract] OR legislation[Title/Abstract] OR decree[Title/Abstract]) AND (Europe[Title/Abstract] OR "European Union"[Title/Abstract] OR EU[Title/Abstract] OR UK[Title/Abstract] OR "United Kingdom"[Title/Abstract] OR "Engl*"[Title/Abstract] OR "Wales"[Title/Abstract] OR "Scot*"[Title/Abstract] OR "Northern Ir*"[Title/Abstract] OR France[Title/Abstract] OR French[Title/Abstract] OR German*[Title/Abstract] OR Belgi*[Title/Abstract] OR "Malta"[Title/Abstract] OR "Maltese"[Title/Abstract] OR Estonia*[Title/Abstract] OR Swed*[Title/Abstract] OR Portug*[Title/Abstract] OR Poland[Title/Abstract] OR Polish[Title/Abstract] OR Ital*[Title/Abstract])

Supplementary Table 4. Country-specific details on the regulation of artificial intelligence.

Country	AI regulations	Processing Data	Technology Appraisal	Supporting Innovation	Health & Human rights
Europe	AI systems are regulated by the EU AI Act, adopted in March 2024. The EU AI Act introduced the most ambitious framework to date designed to guide the development and usage of AI that seeks to regulate AI systems across applications and contexts. It delineates between minimal, limited, high, and unacceptable risks. A European Artificial Intelligence Board (a scientific panel of independent experts) and an EU database for high-risk AI systems will be established.	The data policies in the EU cover the protection and free flow of personal data (the GDPR, Regulation (EU) 2018/1725), the reuse and free flow of non-personal data (Directive (EU) 2019/1024; Regulation (EU) 2018/1807), and rules for use of and access to data (Data Governance Act; Data Act).<br>Further, rules for the protection of databases are mentioned (Directive 96/9/EC).<br>To enhance data protection within the EU an external advisory group on ethical dimensions of data protection exists, which has been established by the “European Data Portection Supervisor Decision of 3 December 2015”<br>.<br><br>The novel Interoperable Europe Act seeks to improve the cross-border interoperability of networks and information systems in public services by establishing common rules.	The EU adopted different policies in the field of technology to ensure proper functioning of the internal market. While the General Product Safety Regulation (GPSR) offers a broad-based framework for the safety of products being placed on the EU market, there are additional sector-specific regulations setting standards. These standards seek to establish harmonised rules for the electronic communications networks and services (Directive (EU) 2018/1972), for health technologies (Regulation (EU) 2021/2282, Regulation (EU) 2017/745, Regulation (EU) 2017/746).<br>Different acts exist for the protection and security of cyberspace and digital markets, on which member states should build upon (Directive (EU) 2022/2555, Council Decision(CFSP) 2019/797, Regulation (EU) 2019/1150, Regulation (EU) 2022/2065, Regulation (EU) 2022/1925).	As part of its innovative friendly framework, the EU established regulations for the legal protection of copyrights and related rights in the information society and the digital market (Directive 2018/790).<br>Furthermore, different regulations and decisions/conclusions from the Council, the Commission, or other EU level bodies established different programs or projects with the overall aim of enhancing Europe's competitiveness in facilitating innovation and strengthening the scientific and technological base: “Digital Europe Programme”, “Digital Decade Policy Programme 2030”, ‘Promoting Responsible Innovation in Artificial Intelligence for Peace and Security’, the European High Performance Computing Joint Undertaking, “EU4Health Programme”, the European Institute of Innovation and Technology (EIT), investEU programme,and Horizon Europe. Furthermore, the	The important standard in the EU for safeguarding human rights is the “Convention on Human Rights and freedoms”.<br>Further, the “council decision 2022/2349 of 21 November 2022” authorised the negotiation for a Council of Europe convention on AI, human rights, democracy, and the rule of law.<br>Already established is a special committee on artificial intelligence in a digital age, to analyse the future impact of AI on different sectors of the EU economy.<br>The newly established “Citizens, Equality, Rights and Values Programme” seeks to support citizens' initiatives to protect and promote EU rights.

			For protecting the cyberspace across the EU, the European Union Agency for Cybersecurity (ENISA) and the European Cybersecurity certification framework (Regulation (EU). 2019/881) exist.<br>Market surveillance and compliance of products in general are specified in Regulation (EU) 2019/1020.	eHealth Network and a “special committee on artificial intelligence in a digital age” exist.<br>All these initiatives are not solely focused on artificial intelligence but include specific objectives for AI.	
Belgium	No binding national policy identified	The data policies in Belgium cover the protection of personal data by the GDPR and the national adaptation of the aforementioned with the Belgian Data Protection Act.<br>To facilitate the availability of health (care) data, a health (care) data agency was established: the Health Data Agency.	No binding national policy identified	Belgian law outlines the rights and limitations of a person or entity (usufructuary) that are granted the temporary use and benefit of intellectual property and describes the responsibilities and rights of the actual owner of the intellectual property.	No binding national policy identified
Estonia	No binding national policy identified	The data policy in Estonia covers the protection of personal data with the Estonian Data Protection Act (RT I, 13.03.2019, 2), which elaborates and supplements the GDPR.<br>The Act of the Government of the Republic (RT I, 30.06.2023, 11) specifies in which case individuals’ rights, as specified in the Data Protection Act, may	No binding national policy identified	No binding national policy identified	No binding national policy identified

		be limited (for ensuring national security)<br>Furthermore, the “Law on the Organization of Health Care Services” (RT I 2001, 50, 284) guides the processing of personal data necessary for the provision of health care services.<br>The “Statute for maintaining a database of identity documents” (RT I, 22.12.2015, 45) regulates the database of identity documents and the “Data exchange layer of information systems” (RT I, 27.09.2016, 4) sets requirements for information systems' data exchange layer.<br>One specific regulation lays down the requirements and procedures for managing the Estonian information gateway eesti.ee (Requirements and procedures for managing, making information available, developing and using the Estonian information portal eesti.ee (RT I, 25.03.2021, 5)).			
France	No binding national policy identified	The protection of personal data is based primarily on Law No. 78-17. It was enacted in 1978 and has been modified with Law no. 2018-493 to align it to the GDPR.<br>Decree No. 2021-848 addresses the processing and	There is a strong focus on the protection of consumers and the defence of digital sovereignty in French policy concerning technological appraisal. To achieve this, there are rules relating to safety and confidentiality in electronic	France established the Defence Innovation Agency, which has the aim to to capture innovation and accelerate its deployment for the benefit of the ministry of defense.<br>French intellectual property law covers work that has been	The Scientific Council on radicalization processes aims to promote prevention and the fight against the processes of radicalization.

		use of health data (so called national health data system) for multiple purposes, e.g. the comparison of care practices and the evaluation of health and social protection policies. The implementation and development of the national health data system is being supported by a strategic data committee of health.<br>In France, the use and purpose of automated processing of personal data within different databases is regulated. For example, “SI Honorability” intends “to enable the authorized persons […] to check of the good repute of persons subject”in the sports field to counter cases of sexual violence. “Chamollion” provides data for the work, employment, and integration administrators. It has been implemted in accordance with the GDPR, as this processing is implemented for the execution of a mission of public interest.	communication services (Postal and Electronic Communications Code), to combat misinformation on online platforms (Law no. 2018-1202), and to regulate commercial influence of influencers (Law no. 2023-451).<br>Different agencies /institutions exist to uphold/control the rules/requirements specified within the above described policies. For example exists the Anti-cybercrime office (Law no. 2023-451) as well as the Joint Programs and Cyber Protection Command (CPIC)(Instruction No. 1221/DEF/EMA/CPI) in the area of cyberspace safety. The digital agency of security civil represents the ministry of civil security in respect to affected good and intellectual property of current productions and good (Decree No. 2018-856).	“created, independently of any public disclosure, by the sole fact of the realization, even if unfinished, of the author's conception”. This may include works like software, it does not give any guidance on the characteristics of the author. Furthermore, protection of intellectual property by online content-sharing service providers is regulated by transposing EU Directive 2019/790	

Germany	No binding national policy identified	The data protection policies in Germany cover the protection of personal data (adaptation of the GDPR), the reuse of public sector data and the use of electronic health records data. The Law to accelerate the digitalization of the healthcare system and the Health Data Usage Act seek to increase the availability and usability of health records data for research purposes by focusing on opt-out option for health data processing instead of opt-in.	The existing policies covers the privacy in telecommunications and media (Telecommunications Telemedia Data Protection Act), the security of information technology systems and of digital services by transposing Directive 2016/1148.<br>The responsible body for information security is the Federal Office for Information Security(Trust Services Act).	As part of transforming its healthcare sector, the development of digital innovations and the digitalization of healthcare are mentioned in the Digital Supply Act. Another policy established the establishment of a specific agency (SPRIND) to support leap innovations.<br>The intellectual property law requires the work to be created personally intellectually (Copyrights and Related Rights Act). Further, Germany transposed Directive 2019/790 regulating copyright in the digital internal market .	No binding national policy identified
Italy	No binding national policy identified	No binding national policy identified	No binding national policy identified	The Decree 12 September 2017, n. 214:establishes highly specialized competence centers, which focus on the assessment and training of business and the ”implementation of innovation, industrial research and experimental development projects proposed by companies”. Further, the ITS Academies aim to train highly specialised technicians who	The National Civil Protection Service is amongst others responsible for the protection of life, goods, and physical integrity from natural damage or danger and from damaging results of human activity.

				can quickly enter a market that is increasingly in need of technical and technological skills (Law15 July 2022, n. 99). The Fund for technological innovation should support the country’s technological innovation (Law 30 December 2018, n.145).	
Malta	No binding national policy identified	The data protection policies in Malta cover the re-use of public sector data, the protection of personal data (adaptation of the GDPR) and the processing of health data for insurance purposes. Reuse of personal data in the health sector remains possible for a number of purposes, including the functioning of the health system, protecting public health, and research activities.	The Gaming Act regulates the protection of persons in regards to gaming.<br>With the “Medical Devices and In-Vitro diagnostic Medical Devices Provision on the Maltese Market Regulations, 2020” Malta transposed the related EU medical devices Regulations.	The development of innovative technologies is being encouraged with the Malta Digital Innovation Authority Act. Further, the Innovative Technology Arrangements and Services Act gives the Agency the possibility to certify innovative technologies arrangements.	The Public Administration Act specifies which department and which person is responsible to ensure the protection of the public and to safeguard public health.
Poland	No binding national policy identified	The personal data is being protected in Poland with the adopted GDPR.	No binding national policy identified	No binding national policy identified	No binding national policy identified
Portugal	No binding national policy identified	No binding national policy identified	Portugal’s policy regulates the security of cyberspace.	For promoting innovations, the creation of technological free zones are stipulated within Decree-Law No. º 67/2021, of July 3. Furthermore, innovation and incubation centres are created, which focus on “promoting the development of entrepreneurship and other economic activities”.	The Portuguese Chapter of Human Rights in the Digital Age states the need to respect fundamental rights when using AI or algorithms.

Sweden	No binding national policy identified	The Swedish Data protection policies regulate the processing of personal data for the Swedish Medicine Agency (2020:421), the processing of personal data in health care ((2008:355), and the re-use of public sector information (2022:818).<br>Further, it is possible to perform automated-decision making in the case of administrative cases by the courts (2017:900) or on municipality level (2017:725).	Sweden has different laws to secure the safety of a product or good. These laws encompass general products, the use of medical devices (2021: 600; SOSF 2008:1), electronic communications networks/services (2022: 482), network and information systems (2018:1174), and digital services (2018:1174).	No binding national policy identified	Sweden has different policies (SOSFS 2011:9; 2022:1250; 2010:659) stating the requirements and importance of good and high quality healthcare. Further the “Patient Law” specifies that the patient needs to receive information about the proposed treatment. The “Discrimination Act” aims to counteract discrimination and promote equal rights and opportunities for everyone.
United Kingdom	No binding national policy identified	The legal base for personal data protection in the UK consists of two acts: Its Data Protection Act and its adopted GDPR. With a specific data protection act for the United States (US), personal data transfers to the US are being regulated in line with the above-mentioned acts.<br>Law “2015 No. 1415” covers the reuse of public sector information.	The “Online Safety Act 2023” and “The Network and Information Systems Regulations” establishes standards for the security of internet services as well as network and information systems.	No binding national policy identified	No binding national policy identified

Supplementary Table 5. Policy-specific details on the regulation of artificial intelligence.

Country	Year	Date of Latest Legislative Update	Policy name (original version)	Policy name (translated to english)	Content (translated to english)
Belgium	2023		2023031414/N- Wet houdende oprichting en organisatie van het Gezondheids(zorg)data-agentschap	Act establishing and organising the Health (Care) Data Agency	Art. 4. The Health (Care) Data Agency aims at the following goals:<br>1° Facilitating the availability of health (care) data and health (care) related data;<br>2° Developing and implementing a policy strategy on health (care) data and health (care) related data;<br>3° Stimulating <innovation>, scientific research and policy-supporting research.<br>The objectives of the Health (Care) Data Agency as referred to in the first paragraph concern the reuse of health (care) data and health (care) related data.<br>The Health (Care) Data Agency is not a supervisory authority within the meaning of Article 51 of Regulation (EU) 2016/679 of the European Parliament and of the Council of 27 April 2016 on the protection of individuals with regard to the processing of personal data and on the free movement of such data and repealing Directive 95/46/EC. The objectives to be pursued by the Health (Care) Data Agency and the missions assigned to it as referred to in Article 5 are without prejudice to the missions and powers of the Data Protection Authority, as stipulated in the Act of 3 December 2017 establishing the Data Protection Authority.
Belgium	2018		2018073046/N Wet betreffende de bescherming van natuurlijke personen met betrekking tot de verwerking van persoonsgegevens	Act on the Protection of Natural Persons with regard to the Processing of Personal Data	Art. 99. Notwithstanding Title 4, the consultation for historical, scientific or statistical purposes, by a further processing controller, of personal data of the intelligence and security services and of their personnel shall be authorised by the intelligence and security service concerned if it does not prejudice its missions, its obligations referred to in Articles 13(3) and 13/4(2) of the Act of 30 November 1998, an ongoing investigation or judicial enquiry, or Belgium's relations with foreign states or international organisations and in accordance with the Act of 11 December 1998. Any request to the State Archives for further processing of personal data of the intelligence and security services and of their personnel for purposes other than those referred to in the first paragraph shall be refused insofar as the purpose is legitimate and the intelligence and security service concerned considers that the processing cannot prejudice the interests referred to in the first paragraph.<br><br>Art. 101. The personal data referred to in Article 99 shall be anonymised prior to their consultation. If further processing of anonymised data does not allow the achievement of historical, scientific or statistical purposes, the intelligence and security service may allow the consultation of pseudonymised data.<br>If the anonymisation or pseudonymisation does not make the identification of the data impossible, the intelligence and security service shall refuse the consultation if it causes a disproportionate interference with private life.<br>If further processing of pseudonymised data does not allow to achieve the historical, scientific or statistical purposes, the intelligence and security service may allow the consultation of non-pseudonymised data if it does not cause a disproportionate interference with private life.

					Art. 102. Notwithstanding Title 4, communication or publication of non-anonymised or non-pseudonymised personal data referred to in Article 99, consulted by the further processing controller, shall only be possible with the agreement of the intelligence and security service concerned and under the conditions it establishes.
Belgium	2018		2018090501/N - Wet tot oprichting van het informatieveiligheidscomité en tot wijziging van diverse wetten betreffende de uitvoering van verordening (EU) 2016/679 van 27 april 2016 van het Europees Parlement en de Raad betreffende de bescherming van natuurlijke personen in verband met de verwerking van persoonsgegevens en betreffende het vrije verkeer van die gegevens en tot intrekking van richtlijn 95/46/EG	2018090501/N - Law establishing the Information Security Committee and amending various laws on the implementation of Regulation (EU) 2016/679 of 27 April 2016 of the European Parliament and of the Council on the protection of individuals with regard to the processing of personal data and on the free movement of such data and repealing Directive 95/46/EC	Art. 12. An article 5bis is inserted in the same Act, reading:<br>"Art. 5bis. Without prejudice to the processing of personal data for archiving in the public interest, scientific or historical research or statistical purposes referred to in Article 89 of Regulation (EU) 2016/679 of 27 April 2016 of the European Parliament and of the Council on the protection of individuals with regard to the processing of personal data and on the free movement of such data and repealing Directive 95/46/EC (General Data Protection Regulation), the social security institutions referred to in Art, 2°, the social inspection services and the Directorate of Administrative Fines of the Department of Legal Studies, Documentation and Disputes of the Federal Public Service Employment, Labour and Social Dialogue, either individually or jointly, with a view to the prevention, establishment, prosecution and punishment of violations of social regulations falling within their respective competences, and with a view to the collection and recovery of amounts falling within their respective competences, where appropriate, after deliberation by the competent chamber of the Information Security Committee, collect, process and compile all data necessary for the application of labour law and social security legislation into a data warehouse that enables them to carry out data mining and data matching processes, including profiling within the meaning of Article 4(4) of the General Data Protection Regulation.<br><br>For the purposes of this provision, the following definitions shall apply:<br>1° "data warehouse": a data system containing a large amount of <digital> data that lends itself to analysis;<br>2° "data mining": the sophisticated search for information in large data sets;<br>3° "data matching": comparing multiple sets of collected data with each other.<br>The controller for the data processing referred to in the first paragraph is the institution or service referred to in the first paragraph that is responsible for the relevant processing in the data warehouse. Where two or more controllers jointly determine the purposes and means of processing, they shall be joint controllers.<br><br>Without prejudice to storage necessary for processing for archiving in the public interest, scientific or historical research or statistical purposes referred to in Article 89 of Regulation (EU) 2016/679 of 27 April 2016 of the European Parliament and of the Council on the protection of individuals with regard to the processing of personal data and on the free movement of such data and repealing Directive 95/46/EC (General Data Protection Regulation) personal data resulting from the processing operations in the data warehouse shall be kept for no longer than necessary for the purposes for which they are processed including the requirements on the application of recurrence and revocation of a granted extension with a maximum retention period not exceeding one year after the statute of limitations of all claims falling within the competence of the controller and, where applicable, the full payment of all related amounts.

					The controller shall prepare a list of the categories of persons who may access the personal data in the data warehouse, describing their capacity in relation to the processing of the targeted data. This list shall be kept at the disposal of the Data Protection Authority.<br>The controller shall ensure that the designated persons are bound by a legal or statutory obligation, or by an equivalent contractual provision, to respect the confidentiality of the data concerned.<br>Where personal data are communicated to the Crossroads Bank or to a social security institution, the deliberation must stipulate, where appropriate, that these data may be processed in the context of the purposes of processing in the data warehouse referred to in the first paragraph."
Belgium	2020		2020020416/N - Wet houdende boek 3 "Goederen" van het Burgerlijk Wetboek	2020020416/N - Law containing Book 3 'Goods' of the Civil Code	Art. 3.166. Usufruct on intellectual rights<br>The usufruct relating to an intellectual property right grants the usufructuary the right to normal exploitation thereof. In this context, the usufructuary can only conclude contracts insofar as the payment of the remuneration is spread over the total duration of the contract. Otherwise, the consent of the bare owner is required.<br>At the end of the usufruct, the contracts concluded exclusively by the usufructuary continue to have effect, without prejudice to the bare owner's right to terminate them subject to three years' notice.<br>Unless the moral rights belong to a third party, they must be exercised by agreement between the usufructuary and the bare owner; if no agreement can be reached, the most diligent party shall take the matter to court.
Council of Europe	1981			Convention for the protection of individuals with regard to Automatic Processing of Personal data	Article 1 – Object and purpose<br>The purpose of this Convention is to secure in the territory of each Party for every individual, whatever his nationality or residence, respect for his rights and fundamental freedoms, and in particular his right to privacy, with regard to automatic processing of personal data relating to him ("data protection").<br><br>Article 3 - Scope<br>The Parties undertake to apply this Convention to automated personal data files and automatic processing of personal data in the public and private sectors. Any State may, at the time of signature or when depositing its instrument of ratification, acceptance, approval or accession, or at any later time, give notice by a declaration addressed to the Secretary General of the Council of Europe:<br>a. that it will not apply this Convention to certain categories of automated personal data files, a list of which will be deposited. In this list it shall not include, however, categories of automated data files subject under its domestic law to data protection provisions. Consequently, it shall amend this list by a new declaration whenever additional categories of automated personal data files are subjected to data protection provisions under its domestic law;<br>b. that it will also apply this Convention to information relating to groups of persons, associations, foundations, companies, corporations and any other bodies consisting directly or indirectly of individuals, whether or not such bodies possess legal personality;<br>c. that it will also apply this Convention to personal data files which are not processed automatically.<br><br>Article 5 – Quality of data<br>Personal data undergoing automatic processing shall be:

					a. obtained and processed fairly and lawfully;<br>b. stored for specified and legitimate purposes and not used in a way incompatible with those purposes;<br>c. adequate, relevant and not excessive in relation to the purposes for which they are stored;<br>d. accurate and, where necessary, kept up to date;<br>e. preserved in a form which permits identification of the data subjects for no longer than is required for the purpose for which those data are stored.<br><br>Article 6 – Special categories of data<br>Personal data revealing racial origin, political opinions or religious or other beliefs, as well as personal data concerning health or sexual life, may not be processed automatically unless domestic law provides appropriate safeguards. The same shall apply to personal data relating to criminal convictions.<br><br>Article 7 – Data security<br>Appropriate security measures shall be taken for the protection of personal data stored in automated data files against accidental or unauthorised destruction or accidental loss as well as against unauthorised access, alteration or dissemination.<br><br>Article 8 – Additional safeguards for the data subject<br>Any person shall be enabled:<br>a. to establish the existence of an automated personal data file, its main purposes, as well as the identity and habitual residence or principal place of business of the controller of the file;<br>b. to obtain at reasonable intervals and without excessive delay or expense confirmation of whether personal data relating to him are stored in the automated data file as well as communication to him of such data in an intelligible form;<br>c. to obtain, as the case may be, rectification or erasure of such data if these have been processed contrary to the provisions of domestic law giving effect to the basic principles set out in Articles 5 and 6 of this Convention;<br>d. to have a remedy if a request for confirmation or, as the case may be, communication, rectification or erasure as referred to in paragraphs b and c of this article is not complied with.<br><br>Article 12 – Transborder flows of personal data and domestic law<br>The following provisions shall apply to the transfer across national borders, by whatever medium, of personal data undergoing automatic processing or collected with a view to their being automatically processed.<br>A Party shall not, for the sole purpose of the protection of privacy, prohibit or subject to special authorisation transborder flows of personal data going to the territory of another Party.<br>Nevertheless, each Party shall be entitled to derogate from the provisions of paragraph 2:<br>a. insofar as its legislation includes specific regulations for certain categories of personal data or of automated personal data files, because of the nature of those data or those files, except where the regulations of the other Party provide an equivalent protection;

					b. when the transfer is made from its territory to the territory of a non-Contracting State through the intermediary of the territory of another Party, in order to avoid such transfers resulting in circumvention of the legislation of the Party referred to at the beginning of this paragraph.
Council of Europe	2001			Additional Protocol to the Convention for the Protection of Individuals with regard to Automatic Processing of Personal Data regarding supervisory authorities and transborder data flows	Article 2 Transborder flows of personal data to a recipient which is not subject to the jurisdiction of a Party to the Convention<br>Each Party shall provide for the transfer of personal data to a recipient that is subject to the jurisdiction of a State or organisation that is not Party to the Convention only if that State or organisation ensures an adequate level of protection for the intended data transfer.<br>By way of derogation from paragraph 1 of Article 2 of this Protocol, each Party may allow for the transfer of personal data :<br>a. if domestic law provides for it because of :– specific interests of the data subject, or– legitimate prevailing interests, especially important public interests, or<br>b. if safeguards, which can in particular result from contractual clauses, are provided by the controller responsible for the transfer and are found adequate by the competent authorities according to domestic law.

Council of Europe	2018			Convention for the Protection of Individuals with regard to Automatic Processing of Personal Data as it will be amended by its Protocol CETS No. 223	Article 1 – Object and purpose<br>The purpose of this Convention is to protect every individual, whatever his or her nationality or residence, with regard to the processing of their personal data, thereby contributing to respect for his or her human rights and fundamental freedoms, and in particular the right to privacy.<br><br>Article 2 – Definitions (1)<br>For the purposes of this Convention:<br>a “personal data” means any information relating to an identified or identifiable individual (“data subject”);<br>b “data processing” means any operation or set of operations performed on personal data, such as the collection, storage, preservation, alteration, retrieval, disclosure, making available, erasure, or destruction of, or the carrying out of logical and/or arithmetical operations on such data;<br>c where automated processing is not used, “data processing” means an operation or set of operations performed upon personal data within a structured set of such data which are accessible or retrievable according to specific criteria;<br>d “controller” means the natural or legal person, public authority, service, agency or any other body which, alone or jointly with others, has decision-making power with respect to data processing;<br>e “recipient” means a natural or legal person, public authority, service, agency or any other body to whom data are disclosed or made available;<br>f “processor” means a natural or legal person, public authority, service, agency or any other body which processes personal data on behalf of the controller.<br>Article 3 – Scope (1)<br>1 Each Party undertakes to apply this Convention to data processing subject to its jurisdiction in the public and private sectors, thereby securing every individual’s right to protection of his or her personal data.<br>2 This Convention shall not apply to data processing carried out by an individual in the course of purely personal or household activities.<br><br>Article 5 – Legitimacy of data processing and quality of data (1)<br>1 Data processing shall be proportionate in relation to the legitimate purpose pursued and reflect at all stages of the processing a fair balance between all interests concerned, whether public or private, and the rights and freedoms at stake.<br>2 Each Party shall provide that data processing can be carried out on the basis of the free, specific, informed and unambiguous consent of the data subject or of some other legitimate basis laid down by law.<br>3 Personal data undergoing processing shall be processed lawfully.<br>4 Personal data undergoing processing shall be: a processed fairly and in a transparent manner;<br>b collected for explicit, specified and legitimate purposes and not processed in a way incompatible with those purposes; further processing for archiving purposes in the public interest, scientific or historical research purposes or statistical purposes is, subject to appropriate safeguards, compatible with those purposes;<br>c adequate, relevant and not excessive in relation to the purposes for which they are processed;<br>d accurate and, where necessary, kept up to date;<br>e preserved in a form which permits identification of data subjects for no longer than is necessary for the purposes for which those data are processed.

					Article 6 – Special categories of data (1)<br>1 The processing of:<br>– genetic data;<br>– personal data relating to offences, criminal proceedings and convictions, and related security measures;<br>– biometric data uniquely identifying a person<br>- personal data for the information they reveal relating to racial or ethnic origin, political opinions, trade-union membership, religious or other beliefs, health or sexual life, shall only be allowed where appropriate safeguards are enshrined in law, complementing those of this Convention.<br>2 Such safeguards shall guard against the risks that the processing of sensitive data may present for the interests, rights and fundamental freedoms of the data subject, notably a risk of discrimination.<br>Article 7 – Data security (1)<br>1 Each Party shall provide that the controller, and, where applicable the processor, takes appropriate security measures against risks such as accidental or unauthorised access to, destruction, loss, use, modification or disclosure of personal data.<br>2 Each Party shall provide that the controller notifies, without delay, at least the competent supervisory authority within the meaning of Article 15 of this Convention, of those data breaches which may seriously interfere with the rights and fundamental freedoms of data<br>subjects.<br>Article 8 – Transparency of processing (2)<br>1 Each Party shall provide that the controller informs the data subjects of:<br>a his or her identity and habitual residence or establishment;<br>b the legal basis and the purposes of the intended processing;<br>c the categories of personal data processed;<br>d the recipients or categories of recipients of the personal data, if any; and<br>e the means of exercising the rights set out in Article 9, as well as any necessary additional information in order to ensure fair and transparent processing of the personal data.<br>2 Paragraph 1 shall not apply where the data subject already has the relevant information.<br>3 Where the personal data are not collected from the data subjects, the controller shall not be required to provide such information where the processing is expressly prescribed by law or this proves to be impossible or involves disproportionate efforts.<br><br>Article 9 – Rights of the data subject (1)<br>1 Every individual shall have a right:<br>a not to be subject to a decision significantly affecting him or her based solely on an automated processing of data without having his or her views taken into consideration;<br>b to obtain, on request, at reasonable intervals and without excessive delay or expense, confirmation of the processing of personal data relating to him or her, the communication in an intelligible form of the data processed, all available information on their origin, on the preservation period as well as any other information that the controller is required to provide in order to ensure the transparency of processing in accordance with Article 8,<br>paragraph 1;

					c to obtain, on request, knowledge of the reasoning underlying data processing where the results of such processing are applied to him or her;<br>d to object at any time, on grounds relating to his or her situation, to the processing of personal data concerning him or her unless the controller demonstrates legitimate grounds for the processing which override his or her interests or rights and fundamental<br>freedoms;<br>e to obtain, on request, free of charge and without excessive delay, rectification or erasure, as the case may be, of such data if these are being, or have been, processed contrary to the provisions of this Convention;<br>f to have a remedy under Article 12 where his or her rights under this Convention have been violated;<br>g to benefit, whatever his or her nationality or residence, from the assistance of a supervisory authority within the meaning of Article 15, in exercising his or her rights under this Convention.<br>2 Paragraph 1.a shall not apply if the decision is authorised by a law to which the controller is subject and which also lays down suitable measures to safeguard the data subject's rights, freedoms and legitimate interests.<br><br>Article 14 – Transborder flows of personal data (1)<br>1 A Party shall not, for the sole purpose of the protection of personal data, prohibit or subject to special authorisation the transfer of such data to a recipient who is subject to the jurisdiction of another Party to the Convention. Such a Party may, however, do so if there is a real and serious risk that the transfer to another Party, or from that other Party to a non-Party, would lead to circumventing the provisions of the Convention. A Party may also do so, if bound by harmonised rules of protection shared by States belonging to a regional international<br>organisation.<br>2 When the recipient is subject to the jurisdiction of a State or international organisation which is not Party to this Convention, the transfer of personal data may only take place where an appropriate level of protection based on the provisions of this Convention is secured.<br>3 An appropriate level of protection can be secured by:<br>a the law of that State or international organisation, including the applicable international treaties or agreements; or<br>b ad hoc or approved standardised safeguards provided by legally-binding and enforceable instruments adopted and implemented by the persons involved in the transfer and further processing.<br>4 Notwithstanding the provisions of the previous paragraphs, each Party may provide that the transfer of personal data may take place if:<br>a the data subject has given explicit, specific and free consent, after being informed of risks arising in the absence of appropriate safeguards; or<br>b the specific interests of the data subject require it in the particular case; or<br>c prevailing legitimate interests, in particular important public interests, are provided for by law and such transfer constitutes a necessary and proportionate measure in a democratic society; or it constitutes a necessary and proportionate measure in a democratic society for freedom<br>of expression.

					5 Each Party shall provide that the competent supervisory authority within the meaning of Article 15 of this Convention is provided with all relevant information concerning the transfers of data referred to in paragraph 3, littera b and, upon request, paragraph 4, litterae b and c.<br>6 Each Party shall also provide that the supervisory authority is entitled to request that the person who transfers data demonstrates the effectiveness of the safeguards or the existence of prevailing legitimate interests and that the supervisory authority may, in order to protect the rights and fundamental freedoms of data subjects, prohibit such transfers, suspend them or subject them to condition

Council of Europe	1950	2021		convention for the protection of human rights and fundamental freedoms as amended by protocol no. 15	Article 8 - Right to respect for private and family life<br>Everyone has the right to respect for his private and family life, his home and his correspondence. There shall be no interference by a public authority with the exercise of this right except such as is in accordance with the law and is necessary in a democratic society in the interests of national security, public safety or the economic well-being of the country, for the prevention of disorder or crime, for the protection of health or morals, or for the protection of the rights and freedoms of others.<br><br>Article 10 – Freedom of expression<br>Everyone has the right to freedom of expression. This right shall include freedom to hold opinions and to receive and impart information and ideas without interference by public authority and regardless of frontiers. This article shall not prevent States from requiring the licensing of broadcasting, television or cinema enterprises.<br>The exercise of these freedoms, since it carries with it duties and responsibilities, may be subject to such formalities, conditions, restrictions or penalties as are prescribed by law and are necessary in a democratic society, in the interests of national security, territorial integrity or public safety, for the prevention of disorder or crime, for the protection of health or morals, for the protection of the reputation or rights of others, for preventing the disclosure of information received in confidence, or for maintaining the authority and impartiality of the<br>judiciary.<br><br>Article 14 – Prohibition of discrimination<br>The enjoyment of the rights and freedoms set forth in this Convention shall be secured without discrimination on any ground such as sex, race, colour, language, religion, political or other opinion, national or social origin, association with a national minority, property, birth or other status.
Council of Europe	2022			convention on cybercrime - protocol on xenophobia and racism: second protocol on enhanced co-operation and disclosure of elctronic evidence	Article 1 - Definitions<br>a. "computer system" means any device or a group of interconnected or related devices, one or more of which, pursuant to a program, performs automatic processing of data;<br><br>Article 2 - illegal access<br><br>Article 3 - illegal interception<br><br>Article 4 - data interference<br><br>Article 5 - system interference<br><br>Article 6 - misuse of devices<br><br>Article 10 - Offences related to infringements of copyright and related rights

					1. Each Party shall adopt such legislative and other measures as may be necessary to establish as criminal offences under ist domestic law the infringement of copyright, as defined under the law of that Party, pursuant to the obligations it has undertaken under the Paris Act of 24 July 1971 revising the Bern Convention for the Protection of Literacy and Artistic Works, the Agreement on Trade-Related Aspects of Intellectual Property Rights and the WIPO Copyright Tretay, with the exception of any moral rights conferred by such conventions, where such acts are committed wilfully, on a commercial scale and by means of a computer system.

Estonia	2019		Isikuandmete kaitse seaduse rakendamise seadus	The Act on the Implementation of the Personal Data Protection Act (RT I, 13.03.2019, 2)	„ 4 1 . chapter HEALTHCARE DATABASE<br><br>§ 46 1 . Health Insurance Fund databas<br>The Health Insurance Fund's database is kept for the purpose of fulfilling the public tasks of the Health Insurance Fund arising from the laws, which are providing health insurance benefits, paying for health services and performing other tasks related to the organization of health care services in accordance with the Health Insurance Act, the Act on the Organization of Health Care Services and other legal acts and the expenses stipulated in the Health Insurance Fund's budget.<br><br>§ 46 2 . Responsible processor of the Health Insurance Fund database<br>The responsible processor of the Health Insurance Fund's database is the Health Insurance Fund.<br><br>§ 46 3 . Data to be entered into the Health Insurance Fund's database<br>(1) The following data shall be entered into the Health Insurance Fund's database:<br>1) general data of the person – personal identification number and date of birth, first and last name, place of residence, current account and contact information;<br>2) data underlying the origination, termination and suspension of insurance coverage;<br>3) data underlying payment for non-monetary health insurance benefits;<br>4) data underlying the payment of financial health insurance benefits;<br>5) health care provider and other data related to health care;<br>6) other data necessary for the health insurance fund to perform its tasks arising from the Health Insurance Act, the Health Services Organization Act and other legislation.<br>(2) The data of the Health Insurance Fund's database shall be stored for 75 years from the date of entry into the database or 30 years after the person's death. Logs and basic data are stored in accordance with the regulations of the database.<br>(3) The bailiff has the right to process the temporary incapacity benefit data in the Health Insurance Fund's database in order to seize and release the insured person's compensation.<br>(4) A fact indicated in an entry in the Health Insurance Fund's database acquires legal meaning from the entry, unless a different due date is provided by law.<br>(5) The entry in the HIF database is made within five calendar days after the arrival of the duly completed documents on which the entry is based at the HIF.<br><br>§ 46 4 . The right to collect data<br>(1) The health insurance fund has the right to receive data from a person in the cases provided for in legislation, including special types of personal data, if this data is necessary for the performance of the tasks assigned to the health insurance fund by law.<br>(2) The health insurance fund has the right to demand personal data and other data from persons who have entered into a contract with it, as well as from other persons and state and local government unit institutions in the cases provided for in the legislation, if this data is necessary for the performance of the tasks assigned to the health insurance fund by law.<br>(3) The persons specified in subsections 1 and 2 of this section may not charge a fee for issuing data to the health insurance fund.

					(4) The person or institution obliged to release the data must fulfill their obligation without delay, but no later than within the deadline notified by the health insurance fund upon request for data, or justify the impossibility of properly fulfilling the obligation to the health insurance fund in writing.<br><br>§ 46 5 . Statute of the Health Insurance Fund database<br>(1) The Health Insurance Fund database is established and its statutes are established by a regulation of the minister responsible for the field.<br>(2) The basic regulations of the Health Insurance Fund's database stipulate:<br>1) the structure of the database and the detailed composition of the data;<br>2) list of basic documents necessary for entering data into the database;<br>3) the procedure for keeping records of receiving and issuing data;<br>4) the list of data providers, the procedure for accessing and issuing data;<br>5) the procedure for correcting incorrect data and informing about it;<br>6) conditions and procedure for closing access to data;<br>7) more precise data storage procedure;<br>8) other conditions necessary for maintaining the database.";<br>6) the law is supplemented by § 48 4 in the following wording:<br>§ 48 4 . Rights and obligations related to the Health Insurance Fund's database<br>The rights and obligations related to the health insurance database established on the basis of the Health Insurance Act shall be transferred to the health insurance database established on the basis of this Act.".<br>§ 25. Amendment of the Identity Documents Act<br>The following changes will be made to the Identity Documents Act:<br>1) the title of section 9 2 is changed and worded as follows:<br>§ 9 2 . Processing of personal data ";<br>2) section 9 2 is supplemented by paragraphs 7–9 in the following wording:<br>"(7) In the case of the procedures provided for in this Act, the administrative body has the right to process personal data, including special types of personal data.<br>(8) The administrative body may transmit personal data to third parties in order to find out and check matters of importance in the procedures for issuing and revoking an identity document and in the procedures for issuing, suspending and revoking the e-resident's digital identity card. Third parties may process the personal data provided to them to the extent necessary to find out the circumstances of importance in the proceedings.<br>(9) When carrying out the procedures for issuing and revoking an identity document and when carrying out the procedure for issuing, suspending and revoking the digital identity card of an e-resident, the administrative body has the right to collect data on circumstances that may be important in the proceedings from databases, other institutions and persons performing public tasks and from private persons. The mentioned persons and institutions have the obligation to transfer this data to the administrative body, and the administrative body has the right to process this data.";<br>3) the text of section 15 2 is amended and worded as follows:<br>"(1) The database of identity documents (hereinafter the database) is a database established by the Government of the Republic, the statutes of which are established by a regulation of the minister responsible for the field.

					(2) The purpose of maintaining the database is to ensure public order and national security through the processing of data related to identification and revocation of identity documents provided for in subsection 15 (4) of § 15 of this Act and the data of persons who requested these documents.<br>(3) In order to fulfill the purpose of maintaining the database, data related to the identification of the person, the issuance and invalidation of identity documents provided for in § 15 subsection 4 of this Act and the data of the persons who requested these documents are processed in the course of performing the tasks set forth in the legislation of the European Union, foreign agreements, laws and regulations, and during the corresponding procedures data of the given administrative acts and performed actions.<br>(4) The responsible processor of the data collection is the Police and Border Guard Board.<br>(5) The composition of the data to be entered into the database and the term of their storage shall be determined in the statute of the database.<br>(6) In private-law and public-law relations, data from the database on administrative acts issued and actions performed during the procedures specified in subsection 3 of this section may be used as data on identification of a person and the issuance and invalidation of identity documents provided for in § 15, subsection 4 of this Act.";<br>§ 30. Amendment of the Defense Service Act<br>§ 14 1 . Information processing and restrictions on the right to receive information and personal data<br>(1)The following information, among others, may be processed in order to fulfill or ensure the fulfillment of military duty, military service and substitute service:<br>1) personal data; 2) special types of personal data; 3) anonymized data; 4) personal data addressed to the general public and available from public sources.<br>(2) Information is processed directly by the Ministry of Defence, the Defense Resources Board, the Defense Forces, the Defense Resources Board's medical commission, the Defense Forces medical commission or the Defense Ministry's medical appeals commission (hereinafter referred to in this section together with the organization managing the defense forces).<br>(3) The institution organizing the defense force duty may limit the following rights of the data subject when processing information, including:<br>1) to find out about the automated or non-automated processing of his personal data, including which personal data is processed, as well as the purpose, legal basis, scope and reason of the processing;<br>2) find out the recipients of his personal data and the categories of personal data to be published, as well as information on whether his personal data will be transferred to a third country or an international organization;<br>3) find out about the technical and organizational protection measures and access restrictions for the processing of his personal data;<br>4) get acquainted with collected and processed personal data;<br>5) demand restriction of the processing of his personal data;<br>6) demand the transfer of his personal data;<br>7) learn about a breach of personal data.

Estonia	2016	2019	Infosüsteemide andmevahetuskiht	Data exchange layer of information systems (RT I, 27.09.2016, 4)	Chapter 1 General settings<br>§ 1. Scope<br>(1) The regulation establishes requirements for the data exchange layer of information systems, its use and management.<br>(2) The Regulation does not apply to an information system containing state secrets or classified foreign information.<br><br>§ 2. Terms In the Regulation, the terms are used in the following sense:<br>1) the data exchange layer of information systems (hereinafter X-tee) is the technical infrastructure and environment between the members of X-tee, which enables secure and evidential internet-based data exchange; 2) X-tee member is an institution or person who has joined X-tee; 3) the center is the State Information System Agency, which is responsible for managing and developing the X-tee; 4) data service is an X-tee member service through which internet-based data exchange takes place; 5) the data service provider is an X-tee member who provides data service to other members; 6) the user of the data service is a member of X-tee who uses the data service; 7) a data service intermediary is a member of X-tee who allows a physical or legal person external to his organization to access the data service through his information system; 8) the end user of the data service is a natural person who uses the data service through the X-tee member's information system; 9) a message is a set of formatted data that is exchanged between the data service provider and the user via the X-way; 10) the subsystem is a technologically and organizationally defined part of the X-tee member's information system for the provision or use of data services; 11) the access right is enabling the use of the data service in the X-tee software; 12) X-tee base protocol set is a set of rules that ensures safe data exchange operation via a computer network; 13) the security server is a software solution that follows the X-tee base protocol; 14) The X-tee message protocol is a part of the X-tee base protocol suite, which enables X-tee members to process messages; 15) e-stamp is a collection of electronic data that complies with Regulation (EU) No. 910/2014 of the European Parliament and of the Council on trust services required for e-identification and e-transactions in the internal market and which repeals Directive 1999/93/EC (OJ L 257 , 28.08.2014, pages 73–114) (hereinafter Regulation (EU) No. 910/2014 of the European Parliament and of the Council) to the requirements of the advanced or qualified e-stamp; 16) the request log is a part of the security server based on the X-way base protocol, where messages exchanged on X-way, confirmed with an e-stamp, are stored.<br><br>Chapter 2X-way management<br>§ 3. Principles of X-way management<br>The following principles are followed when managing X-tee:<br>1) independence from the platform and architecture – X-tee allows X-tee members on the software platform to communicate with the data service provider on the software platform via the information system;<br>2) multilateralism – the X-tee member's ability to request access to all data services provided via X-tee;<br>3) openness and standardization – international standards and protocols are used whenever possible in the management and development of the X-tee;

					4) security – when exchanging data via X-tee, the integrity, usability and confidentiality of the data do not change.<br>§ 4. Tasks of the center<br><br>(1) The center:<br>1) manages the information in both production and test environments of X-tee members, X-tee registered security servers and subsystems connected to X-tee, which ensure the availability of information necessary to create X-tee's secure data exchange channel and use data services X-tee to the member's security server;<br>2) organizes the processing of requests regarding membership, subsystem and security server;<br>3) develops the conditions for joining and using the X-tee and publishes them on the center's website;<br>4) ensures the possibility of using the X road;<br>5) monitors the use of X-tee and collects usage statistics;<br>6) handles security incidents;<br>7) restricts the rights of the X-tee member in the cases provided for in this regulation;<br>8) advises the X-tee member on issues related to the X-tee;<br>9) informs the X-tee member about changes in the management or use of the X-tee and all known circumstances preventing the use of the X-tee or maintenance work, by sending an e-mail to the contacts of the X-tee member specified in the state information system management system (hereafter RIHA);<br>10) manages and organizes the connection of the Estonian X-tee environment with other data exchange environments;<br>11) ensures the free availability of standardized security server software to X-tee members;<br>12) ensures compliance of the standard solution of the subsystem intended for the end user of the personal data service with the X-tee message protocol and the free availability of the software to the X-tee member;<br>13) prepares and implements X-tee infrastructure development projects and ensures the architectural integrity of X-tee;<br>14) suspends the availability of the information necessary for using the data service to the security server of the X-tee member in the case specified in subsection 3 of § 14;<br>15) manages and develops the necessary solutions for the registration of members and the trust service, as well as for monitoring the functioning of the X-tee platform.<br>(2) The center is obliged to comply with the following notification deadlines when fulfilling the obligation to notify provided in paragraph 1, point 9:<br>1) One month in advance of any change in the management or use of the X-tee or planned maintenance work;<br>2) When notifying about an extraordinary change in the management and use of the X-tee and unplanned maintenance work, the center has the right to follow a shorter notice period than the one specified in point 1;<br>3) changes in the X-tee base protocol or the X-tee message protocol that lead to changes in the X-tee member's subsystem or data service shall be notified 18 months in advance.<br>Chapter 3Using the X path

					§ 5. Joining X-tee and its membership<br>(1) Joining the X-tee is requested through RIHA.<br>(2) When joining the X-tee, the applicant signs a joining agreement with the center. The rights, obligations and responsibilities of the parties are fixed in the membership agreement.<br>(3) An X-tee member has the right to use the X-tee in the manner prescribed by this regulation and the membership agreement.<br>(4) An X-tee member is obliged to:<br>1) ensure the continuity, management, development and safe and uninterrupted operation of his information system upon joining X-tee;<br>2) adopt the elements of ensuring secure and standardized data exchange provided for in § 7 and adapt their information system to work in the X-tee environment;<br>3) to implement measures that ensure the integrity, confidentiality and availability of data to mitigate security-related risks, and to ensure independent auditing of the implemented measures at least every four years;<br>4) to fulfill the orders sent by the center;<br>5) keep the data about yourself in RIHA up-to-date;<br>6) immediately inform the center about a problem related to the use of X-tee and a circumstance that may affect the fulfillment of the obligations of the center or an X-tee member;<br>7) immediately notify the incident handling department of the center about the security incident and its immediate threat;<br>8) transmit the requests related to the X-way and the information specified in point 6 to the center via RIHA or, if this is impossible, by e-mail;<br>9) at the request of the center, provide the information, security rules and a description of the implementation of the implemented measures necessary to assess the security of the security server.<br>(5) When maintaining the database of the state and local government unit, the member of X-tee, when implementing the measures provided for in clause 4, point 3 of this section, and ensuring independent auditing of the implemented measures, is based on the distinctions provided for in subsection 9, subsection 3 of § 43 of the Public Information Act.<br><br>§ 6. Refusal to join X-tee<br>The center has the right to reject the application for joining the X-tee if:<br>1) the applicant does not have a unique identifier for which it is possible to issue an e-stamp certificate that meets the requirements published on the center's website;<br>2) at the request of the center, the applicant has not submitted the necessary documents to establish the right of representation, or the applicant does not have the right of representation to submit the application;<br>3) the data provided by the applicant when joining is not registered in RIHA or the data is not up-to-date;<br>4) the applicant or his information system does not meet the other requirements set out in this regulation or the operating principles of the X-tee.<br><br>§ 7. Elements of ensuring secure and standardized data exchange<br>Secure and standardized data exchange on the X-way is guaranteed if all the following conditions are met:<br>1) through the creation of a secure data exchange channel in accordance with § 8;

					2) by ensuring the integrity of data exchange with an e-stamp in accordance with § 9;<br>3) by defining the subsystem in accordance with § 10;<br>4) through harmonized data service provision requirements in accordance with § 11;<br>5) when determining the user of the data service through the agreement on the use of the data service and the granting of access rights in accordance with § 12.<br><br>§ 8. Creation of a secure data exchange channel<br>(1) In order to enable the creation of a secure X-tee data exchange channel, the X-tee member must install the security server software information system and register the security server authentication certificate in the center, which must meet the requirements published on the center's website.<br>(2) X-tee is allowed to use only security server software that complies with the X-tee base protocol set approved by the center.<br>(3) When using the security server, the X-tee member is obliged to:<br>1) ensure the existence of a request log of messages exchanged on X-tee certified with an e-stamp and, in case of archiving the request log, to develop a procedure for archiving the request log, which includes the frequency of archiving and the list of information to be archived;<br>2) determine the persons who, and under what conditions, can access the archived request log of the security server if the request log is archived;<br>3) when archiving the request log, ensure the same confidentiality requirements for the processing of archived messages as are required for using the data service;<br>4) host a security server in the territory under the jurisdiction of the Republic of Estonia.<br>(4) When using the security server offered by the center, the X-tee member, in addition to fulfilling the obligations specified in paragraph 3, is obliged to:<br>1) use the security server software in accordance with the instructions published on the center's website;<br>2) update the security server software no later than two months after the center makes software updates available.<br>(5) The security server may be hosted outside the territory under the jurisdiction of the Republic of Estonia only with the permission of the center, if the X-tee member:<br>1) ensures the fulfillment of the obligations stipulated in § 5 subsection 4;<br>2) implements measures to ensure the integrity, confidentiality and availability of data to mitigate security-related risks, and ensures independent auditing of the implemented measures at least every two years.<br>(6) When sharing its security server with another X-way member, an X-way member must use an encrypted connection and two-way authentication to connect the security server and the subsystem.<br><br>§ 9. Ensuring the integrity of data exchange with an e-stamp<br>(1) The integrity of the data exchange and the identification of the connection between the message exchanged on the X-way and the X-way member is ensured by an e-stamp, for the creation of which the X-way member is obliged to use the following trust services in the security server that meet the requirements of Regulation (EU) No. 910/2014 of the European Parliament and of the Council:<br>1) certification service through which a qualified e-stamp certificate is issued;<br>2) certificate validity confirmation service;<br>3) time stamp service.

					(2) The e-stamp formed on X-way is valid if the time difference between the validity confirmation of the used certificate and the time stamp is not more than eight hours.<br>(3) An X-tee member is prohibited from processing data exchanged on X-tee that cannot be confirmed with the e-stamp specified in subsection 1.<br><br>§ 10. X-path interfaced subsystem<br>(1) It is possible to use and provide service on the X-way only on such a subsystem that is registered in the center.<br>(2) In order to register the subsystem on the X-way, the X-way member submits an application to the center through RIHA.<br>(3) It is possible to register only such a subsystem on X-way:<br>1) which is registered in RIHA;<br>2) to which the natural person responsible for the operation of the subsystem and the contact details of the administrator of the security server serving the subsystem are assigned;<br>3) in respect of which measures are applied to ensure the integrity, confidentiality and availability of data to mitigate security-related risks, and independent auditing of the implemented measures is ensured at least every four years, based on the distinctions provided in subsection 5 of § 5.<br>(4) After registering the subsystem, the X-tee member is obliged to:<br>1) assign jobs and positions that have the right to use the subsystem and thus the data services provided to the subsystem, and allow access within the organization only to authorized persons;<br>2) ensure safe and trouble-free operation of the subsystem interfaced with X-tee and compliance with the data service usage agreement between X-tee members.<br>(5) The center has the right to reject the request for subsystem registration or delete the registered subsystem from the register if any of the requirements set forth in subsections 3 and 4 are not met.<br><br>§ 11. Requirements for data service<br>The data service must:<br>1) comply with the X-tee message protocol established by the center;<br>2) be registered in RIHA with a description of the data service that meets the center's requirements and is timely and relevant, and contain information about the security measures necessary for using the data service, taking into account the composition of the data contained in the data service and the nature of the data service;<br>3) be usable also in the X-tee test environment.<br><br>§ 12. Provision and use of data service<br>(1) The data service is provided and used in accordance with the data service usage agreement between X-tee members. The agreement for the use of the data service determines:<br>1) the information security measures necessary for the use of the data service and the organizational, physical and IT security measures required from the subsystem of the user of the data service, taking into account the composition of the data to be processed and the requirements prescribed by legislation;<br>2) permission to mediate the data service to a third party in accordance with § 13;<br>3) service level conditions.

					(2) The data service provider is obliged to:<br>1) register the data service together with the technical description of the data service in the security server and keep the description of the data service relevant and up-to-date both in the security server and in RIHA;<br>2) before entering into an agreement with the user of the data service, make sure whether the user of the data service implements sufficient measures to ensure the integrity, confidentiality and availability of data to mitigate security-related risks;<br>3) ensure that the right of access to the X-tee system is consistent with the data service usage agreement between X-tee members.<br>(3) The use of the data service is possible in the subsystem of the X-tee member, which has been granted access rights to use the specific data service.<br>(4) The user and the provider of the data service are obliged to:<br>1) comply with the agreement on the use of the data service;<br>2) link messages received to the security server with a time stamp.<br>(5) The X-tee member ensures authentication and authorization of the end user participating in the provision or use of data services through its information system.<br><br>§ 13. Mediation of data service<br>(1) An X-tee member may grant access to the subsystem to a physical or legal person outside the organization only if:<br>1) an X-tee member has drawn up and disclosed the data service mediation procedure in accordance with paragraph 2;<br>2) X-tee member has registered as a data service intermediary with X-tee;<br>3) permission to mediate the data service is fixed in the data service usage agreement between X-tee members.<br>(2) The data service mediation procedure must include:<br>1) the basis for the data service mediation;<br>2) procedure for mediated authentication and authorization of the subsystem using the data service;<br>3) the procedure for archiving the mediated authentication and authorization log of the subsystem using the data service and the term for keeping the log;<br>4) The procedure for archiving the X-tee request log and access to the archive and the retention period.<br>(3) As a data service intermediary, the X-tee member is obliged to:<br>1) follow the data service mediation procedure established by himself;<br>2) inform the center and the data service provider, whose data service the mediator has access to, about the change in the data service mediation procedure;<br>3) be based on the rights and obligations between the parties specified in the agreements specified in paragraph 1, point 3, and verify the permissibility of mediating the data service;<br>4) disclose to the data service provider the data of the parties mediated by the subsystem in accordance with the X-tee base protocol.<br><br>§ 14. Termination of X-tee membership

					(1) An X-tee member has the right to cancel membership at any time by submitting a written statement to the center.<br>(2) If the deadline for X-tee membership termination is not specified in the application specified in subsection 1, the membership ends on the working day following the receipt of the aforementioned application.<br>(3) The center has the right to immediately terminate the membership or limit the rights arising from the membership or to give a deadline for remedying the deficiency, if:<br>1) the X-tee member violates the conditions set forth in this regulation, the membership agreement or the data service mediation procedure;<br>2) The X-tee member has submitted false or incomplete data.<br>(4) The center has the right to terminate the membership by notifying the X-tee member by e-mail 30 calendar days in advance.<br><br>Chapter 4Application settings<br>§ 15. Peculiarities of ensuring the integrity of data exchange with an e-stamp<br>(1) The data service provider is obliged to ensure the integrity of the data exchange specified in subsection 1 of § 9 with an e-stamp from January 2, 2017.<br>(2) The user of the data service is obliged to ensure the integrity of the data exchange specified in subsection 1 of § 9 with an e-stamp from June 2, 2017.<br><br>§ 16. Distribution of X-tee software The X-tee software is distributed according to the MIT License for free use of the software .<br><br>§ 17. Revocation of the regulation Regulation No. 78 of the Government of the Republic of April 24, 2008 "Data exchange layer of information systems" is declared invalid.<br><br>§ 18. Entry into force of subsections 2 and 3 of section 9 Paragraphs 2 and 3 of Section 9 enter into force on June 2, 2017.

Estonia	2002	2024	Tervishoiuteenuste korraldamise seadus	The Law on the Organization of Health Care Services (RT I 2001, 50, 284)	§ 4 1 . Processing of personal data<br>(1) A health care service provider who has a statutory duty of confidentiality has the right to process personal data necessary for the provision of health care services, including special types of personal data, without the consent of the data subject.<br>[RT I, 13.03.2019, 2 - enters into force. 15.03.2019]<br><br>(1 1) A health care service provider who has a legal duty of confidentiality has the right to process personal data, including special types of personal data:<br>1) for planning the provision of health care services based on the purpose specified in § 2 subsection 1 of this Act;<br>2) to the extent and for the purpose provided for in Clause 7 of Clause 1 of § 56 of this Act.<br>[RT I, 13.03.2019, 2 - enters into force. 15.03.2019]<br><br>(1 2) The right to process the data specified in point 1 of subsection 1 1 of this section for the purpose of planning the provision of health care services extends to the provision of general medical care and school health care services. The right to data processing for the purpose of planning the provision of health care services extends to the provider of specialized medical care and independent physiotherapy, speech therapy, psychological treatment, nursing care and midwifery care only if there is a previously concluded health care service contract and preventive activities have been implemented within a reasonable time after the contract ends.<br>[RT I, 10.10.2022, 1 - enters into force. 01.10.2023]<br><br>(1 3) The reason and purpose of the processing of personal data, including special types of personal data provided for in subsection 1 1 of this section, must be documented.<br>[RT I, 13.03.2019, 2 - enters into force. 15.03.2019]<br><br>(2) The transfer of data reflecting the health status of a data subject staying in hospital or access to them is allowed to his relatives, unless<br>1) the data subject has prohibited access to the data or their transfer;<br>2) the body conducting the investigation has prohibited access to the data or their transmission in the interest of preventing a crime, catching a criminal or finding out the truth in criminal proceedings.<br>[RT I 2007, 24, 127 - entry into force. 01.01.2008]<br><br>(3) In the provision of health care services, the processing of personal data of the deceased is permitted for the protection of the life and health of the ascendant or descendent related to him or her, as well as the sister or brother.<br>[RT I, 13.03.2019, 2 - enters into force. 15.03.2019]<br>§ 4 2 . Documentation and storage of healthcare service provision and health data<br>[RT I, 13.03.2019, 2 - entered into force. 15.03.2019]

					(1) When documenting the provision of health care services, it is mandatory to comply with the documentation requirements, to use the classifications, lists, address data and health information system standards prescribed in the state information system.<br><br>(2) Documents certifying the provision of healthcare services may be created and stored digitally. Paper documents that have been digitized may be destroyed ahead of time, provided that their integrity and authenticity are preserved within the prescribed retention period.<br><br>(2 1) The healthcare service provider ensures the traceability of personal data processing in the service provider's information system.<br>[RT I, 20.06.2022, 63 - enters into force. 27.06.2022]<br><br>(3) The minister responsible for the field shall establish by regulation the conditions and procedures for the documentation of healthcare services, in which the following shall be submitted:<br>1) data of the healthcare service provider;<br>2) general personal data of the patient;<br>3) patient's health data;<br>4) other data related to service provision.<br><br>(4) From the data collected on the basis of subsection 3 of this section, the data proving the provision of outpatient and inpatient health care services shall be stored for 30 years from the confirmation of the data of the service provided to the patient.<br><br>(5) In contrast to the term specified in subsection 4 of this section, the following data proving the provision of health care services shall be stored as follows:<br>1) the data of the student's health card for five years after graduation or leaving school, as well as the data of the emergency card and the referral letter and the response to the referral letter for five years after the confirmation of the data;<br>2) data of death notification and cause of death notification for ten years from the confirmation of the data;<br>3) a tissue sample containing health data taken to perform a pathomorphological examination of the breath of life is stored based on the need to provide health care services, but not longer than 30 years from the confirmation of the data;<br>4) autopsy report data 30 years after data confirmation;<br>5) blood card, transfusion protocol and post-transfusion reaction protocol data for 30 years after the person's death.<br><br>(6) When processing digitally stored data, the term for storing logs is determined in the ministerial regulation provided on the basis of subsection 3 of this section .<br>[RT I, 13.03.2019, 2 - enters into force. 15.03.2019, the deadlines set out in paragraphs 4-6 shall be applied to documents prepared from 15 March 2019.]

Estonia	2013	2021	Eesti teabevärava eesti.ee haldamise, teabe kättesaadavaks tegemise, arendamise ning kasutamise nõuded ja kord	Requirements and procedures for managing, making information available, developing and using the Estonian information portal eesti.ee (RT I, 25.03.2021, 5)	Chapter 1General settings<br>§ 1. Scope of regulation and single point of contact<br>(1) The regulation establishes the requirements and procedure for managing the Estonian information gateway eesti.ee (hereinafter the information gateway), making information and electronically publicly available services available in the information gateway, and developing and using the information gateway.<br>(2) Information is made public through the information portal and access to the service is offered.<br>(3) The information gateway performs the task of a single point of contact. The basis for the operation of the single contact point is based on the law of the general part of the Code of Economic Activities.<br>[RT I, 12.11.2015, 3 - enters into force. 15.11.2015]<br>(4) The regulation does not regulate the interface of the database with the information gateway.<br>§ 2. Terms<br>In the regulation, the terms are used in the following sense:<br>1) end user of the information gateway (hereinafter end user) – a natural or legal person who uses information disclosed through the information gateway or an electronic publicly available service;<br>2) information disclosed in the information portal – timely and relevant information directed to the end user about the rights of the end user, which helps him to fulfill his obligations arising from the law;<br>3) electronic public service (hereinafter referred to as service) – a service offered by a service provider mediated by an information gateway, which has no access restrictions and can be used on a uniform basis;<br>4) holder of information – the institution and person specified in § 5 subsection 1 of the Public Information Act;<br>5) service provider – the responsible processor of the database providing the service or the information holder or the authorized processor of the database;<br>6) service level agreement - an agreement concluded between the manager of the information gateway and the service provider to enable access to the service through the information gateway and which stipulates the requirements for the quality of the service provided by the service provider in the information gateway and its changes, the service-related requirements for the service provider and the manager of the information gateway, and the rights and obligations of the parties;<br>7) official e-mail address – a unique e-mail address necessary to make information available to the end user through the official e-mail information system, which is formed based on the personal identification code for the end user for a natural person and based on the registry code for the end user for a legal entity.<br>[RT I, 25.03.2021, 2 - enters into force. 28.03.2021]<br>§ 3. Rights and obligations of the administrator of the information gateway<br>(1) The National Information System Agency is the operator of the information gateway (hereinafter the operator) within the meaning of § 32 1 subsection 2 of the Public Information Act.<br>(2) The administrator's rights are as follows:<br>1) to limit the access of the end user of the information gateway to the information gateway or its part, if allowing access would violate the rights of the person or damage the security of the state's information systems;<br>2) close the service in the information gateway if there is no legal basis for providing the service in the information gateway or if the service does not meet the conditions of the service level agreement concluded with the service provider;<br>3) remove outdated or inappropriate information related to the service provider's field of activity from the information gateway without prior consent;<br>4) process non-personalized data about the end user's activity in the information gateway for statistical purposes and to determine the need for information gateway development;<br>5) monitor the activity of the personalized end user and the service provider in the information gateway based on the end user's application;<br>6) to store the data entered into the technical environment of the information gateway and the activities of the end user in the log and session database, taking into account the provisions of § 21;<br>[illegible]

Estonia	2016	2022	Isikut tõendavate dokumentide andmekogu pidamise põhimäärus	Statute for maintaining a database of identity documents (RT I, 22.12.2015, 45)	Chapter 1<br>General settings<br>§ 1. Name of the database<br>The name of the database is the database of identity documents (hereinafter the database).<br><br>§ 2. Responsible processor of the database<br>The responsible processor of the data collection is the Police and Border Guard Board.<br><br>§ 2 1 . Authorized processor of the database<br>The authorized processor of the data collection is the Information Technology and Development Center of the Ministry of the Interior.<br>[RT I, 26.08.2022, 1 - enters into force. 29.08.2022]<br><br>§ 3. Purpose of keeping the database<br>The purpose of keeping a database is to keep records of the issuance and invalidation of identity documents specified in § 15 subsection 4 of the Identification of Persons and Identity Documents Act, as well as the persons who requested said documents, in order to ensure the internal security of the state.<br><br>Chapter 2Structure of the database and requirements for maintaining the database<br>§ 4. Method and composition of the database<br><br>(1) The database is considered a one-level information technology database.<br><br>(2) Digital data collection cards (hereinafter data collection card) are part of the data collection.<br>§ 5. Protection of data contained in the database<br><br>(1) Appropriate organizational, physical and information technology security measures are implemented to ensure the availability, integrity and confidentiality of the data contained in the database.<br><br>(2) The security class of the data contained in the data set is K3T3S2. The security level of the database is high (H).<br>Chapter 3Data to be entered into the database<br>§ 6. Data of identity documents entered into the database<br><br>Data on requests for the following identity documents and identity documents are entered into the database:<br>1) Estonian citizen's passport;<br>2) identity card;<br>3) digital identity card;<br>4) residence permit card;<br>5) passport of a foreigner;<br>6) seafaring certificate;

					7) seaman's service book;<br>8) temporary travel document;<br>9) travel document of the refugee.<br>§ 7. Data related to personal identification or identity verification<br><br>The following data are entered into the database regarding the identification or verification of identity and the person to be identified:<br>1) personal code;<br>2) first name(s);<br>3) surname (surnames);<br>4) date of birth;<br>5) gender;<br>6) place of birth;<br>7) photo or facial image;<br>8) fingerprint images;<br>9) data on the impossibility of taking fingerprints from the person;<br>10) procedure registration number;<br>11) the date of initiation of the identification procedure;<br>12) the method of identifying the person and the reason for the identification procedure;<br>13) Name and number of the document issued by the Police and Border Guard Board;<br>14) name and number of other document issued to the person;<br>15) other information collected during the procedure for identification of the person and important in the procedure;<br>16) first and last name of the official who made the entries in the database.<br>§ 8. Data related to the applicant of the identity document<br><br>(1) The following information about the applicant for an Estonian citizen's passport, identity card, residence permit card, digital identity card, foreigner's passport, seaman's certificate, seafarer's service book, temporary travel document and refugee travel document shall be entered in the database:<br>1) first name(s);<br>2) surname (surnames);<br>3) personal identification number;<br>4) date of birth;<br>5) place of birth;<br>6) gender;<br>7) citizenship;<br>8) contact details (street, house, apartment, city or village, municipality, county, country, postal code, e-mail, telephone);<br>9) photo or facial image;<br>10) fingerprint images;<br>11) data on the impossibility of taking fingerprints from a person;<br>12) signature or signature image;

					13) data on identification of inability to sign;<br>14) justification of its necessity when applying for an additional passport;<br>15) nationality;<br>[RT I, 14.01.2017, 1 - enters into force. 18.01.2017]<br>16) mother tongue;<br>[RT I, 14.01.2017, 1 - enters into force. 18.01.2017]<br>17) education.<br>[RT I, 14.01.2017, 1 - enters into force. 18.01.2017]

Estonia	1996	2023	Vabariigi Valitsuse seadus	Act of the Government of the Republic (RT I, 30.06.2023, 11)	§ 77 1 . Differences in the processing of personal data in the performance of tasks related to national defense and ensuring national security<br><br>(1) The State Chancellery may receive and process, among other things, personal data, special types of personal data, anonymized data and personal data addressed to the general public or available from public sources when processing information necessary for the performance of tasks related to national defense and ensuring national security.<br><br>(2) If the State Chancellery processes personal data in the performance of tasks related to ensuring national security or national defense, the provisions of §§ 14, 17, 19, 20–27, 29–38, 43 and 45 of the Personal Data Protection Act apply to the processing of personal data with the exceptions provided in this section.<br><br>(3) The State Chancellery may limit the rights of the data subject provided for in §§ 23–25 of the Personal Data Protection Act only for the purposes of national defense or ensuring national security, as well as for the protection of the rights or freedoms of another person or data subject, taking into account the protective measures provided for in §§ 24 and 25 of the Personal Data Protection Act<br><br>(4) The special committee for supervision of security institutions of the Riigikogu supervises the fulfillment of the requirements for the processing of personal data in the performance of tasks related to national defense and ensuring national security of the State Chancellery.<br>[RT I, 09.08.2022, 3 - enters into force. 19.08.2022]
Europe	2019		DIRECTIVE 96/9/EC OF THE EUROPEAN PARLIAMENT AND<br>OF THE COUNCIL<br>of 11 March 1996<br>on the legal protection of databases	DIRECTIVE 96/9/EC OF THE EUROPEAN PARLIAMENT AND<br>OF THE COUNCIL<br>of 11 March 1996<br>on the legal protection of databases	Article 13 Continued application of other legal provisions<br>This Directive shall be without prejudice to provisions concerning in particular copyright, rights related to copyright or any other rights or obligations subsisting in the data, works or other materials incorporated into a database, patent rights, trade marks, design rights, the protection of national treasures, laws on restrictive practices and unfair competition, trade secrets, security, confidentiality, data protection and privacy, access to public documents, and the law of contract.
Europe	2019		DIRECTIVE 96/9/EC OF THE EUROPEAN PARLIAMENT AND<br>OF THE COUNCIL<br>of 11 March 1996<br>on the legal protection of databases	DIRECTIVE 96/9/EC OF THE EUROPEAN PARLIAMENT AND<br>OF THE COUNCIL<br>of 11 March 1996<br>on the legal protection of databases	CHAPTER I SCOPE<br>Article 1 Scope<br>1. This Directive concerns the legal protection of databases in any form.<br>2. For the purposes of this Directive, ‘database’ shall mean a collection of independent works, data or other materials arranged in a systematic or methodical way and individually accessible by electronic or other means.<br>3. Protection under this Directive shall not apply to computer programs used in the making or operation of databases accessible by electronic means.

Europe	2019		DIRECTIVE 96/9/EC OF THE EUROPEAN PARLIAMENT AND<br>OF THE COUNCIL<br>of 11 March 1996<br>on the legal protection of databases	DIRECTIVE 96/9/EC OF THE EUROPEAN PARLIAMENT AND<br>OF THE COUNCIL<br>of 11 March 1996<br>on the legal protection of databases	Article 2 Limitations on the scope<br>This Directive shall apply without prejudice to Community provisions relating to:<br>(a) the legal protection of computer programs;<br>(b) rental right, lending right and certain rights related to copyright in the field of intellectual property,<br>(c) the term of protection of copyright and certain related rights.
Europe	2021		REGULATION (EU) 2021/1232 OF THE EUROPEAN PARLIAMENT AND OF THE COUNCIL of 14 July 2021<br>on a temporary derogation from certain provisions of Directive 2002/58/EC as regards the use of technologies by providers of number-independent interpersonal communications services for the processing of personal and other data for the purpose of combating online child sexual abuse	REGULATION (EU) 2021/1232 OF THE EUROPEAN PARLIAMENT AND OF THE COUNCIL of 14 July 2021<br>on a temporary derogation from certain provisions of Directive 2002/58/EC as regards the use of technologies by providers of number-independent interpersonal communications services for the processing of personal and other data for the purpose of combating online child sexual abuse	Article 1 Subject matter and scope<br>1. This Regulation lays down temporary and strictly limited rules derogating from certain obligations laid down in Directive 2002/58/EC, with the sole objective of enabling providers of certain number-independent interpersonal communications services (‘providers’) to use, without prejudice to Regulation (EU) 2016/679, specific technologies for the processing of personal and other data to the extent strictly necessary to detect online child sexual abuse on their services and report it and to remove online child sexual abuse material from their services.<br>2. This Regulation does not apply to the scanning of audio communications<br><br>Article 3 Scope of the derogation<br>1. Articles 5(1) and 6(1) of Directive 2002/58/EC shall not apply to the confidentiality of communications involving the<br>processing by providers of personal and other data in connection with the provision of number-independent interpersonal<br>communications services provided that:<br>(a) the processing is:<br>(i) strictly necessary for the use of specific technology for the sole purpose of detecting and removing online child<br>sexual abuse material and reporting it to law enforcement authorities and to organisations acting in the public<br>interest against child sexual abuse and of detecting solicitation of children and reporting it to law enforcement<br>authorities or organisations acting in the public interest against child sexual abuse;<br>(ii) proportionate and limited to technologies used by providers for the purpose set out in point -----<br><br>Article 4 European Data Protection Board guidelines<br>By 3 September 2021, and pursuant to Article 70 of Regulation (EU) 2016/679, the Commission shall request the<br>European Data Protection Board to issue guidelines for the purpose of assisting the supervisory authorities in assessing<br>whether processing falling within the scope of this Regulation, for existing and new technologies used for the purpose set<br>out in Article 3(1), point (a)(i), of this Regulation, complies with Regulation (EU) 2016/679.

Europe	2018		Regulation (EU) 2018/1807 of the European Parliament and of the Council of 14 November 2018 on a framework for the free flow of non-personal data in the European Union (Text with EEA relevance.)	Regulation (EU) 2018/1807 of the European Parliament and of the Council of 14 November 2018 on a framework for the free flow of non-personal data in the European Union (Text with EEA relevance.)	Article 1 Subject matter<br>This Regulation aims to ensure the free flow of data other than personal data within the Union by laying down rules relating to data localisation requirements, the availability of data to competent authorities and the porting of data for professional users.<br><br>Article 2 Scope<br>1. (a) This Regulation applies to the processing of electronic data other than personal data in the Union, which is:<br>provided as a service to users residing or having an establishment in the Union, regardless of whether the service<br>provider is established or not in the Union; or<br>(b) carried out by a natural or legal person residing or having an establishment in the Union for its own needs.<br>2. In the case of a data set composed of both personal and non-personal data, this Regulation applies to the<br>non-personal data part of the data set. Where personal and non-personal data in a data set are inextricably linked, this<br>Regulation shall not prejudice the application of Regulation (EU) 2016/679.
Europe	2021		Regulation (EU) 2022/868 of the European Parliament and of the Council of 30 May 2022 on European data governance and amending Regulation (EU) 2018/1724 (Data Governance Act) (Text with EEA relevance)	Regulation (EU) 2022/868 of the European Parliament and of the Council of 30 May 2022 on European data governance and amending Regulation (EU) 2018/1724 (Data Governance Act) (Text with EEA relevance)	Article 1 Subject matter and scope<br><br>1. This Regulation lays down:<br>(a) conditions for the re-use, within the Union, of certain categories of data held by public sector bodies;<br>(b) a notification and supervisory framework for the provision of data intermediation services;<br>(c) a framework for voluntary registration of entities which collect and process data made available for altruistic purposes; and<br>(d) a framework for the establishment of a European Data Innovation Board.<br><br>2. This Regulation does not create any obligation on public sector bodies to allow the re-use of data, nor does it release public sector bodies from their confidentiality obligations under Union or national law.<br><br>This Regulation is without prejudice to:<br><br>(a) specific provisions in Union or national law regarding the access to or re-use of certain categories of data, in particular with regard to the granting of access to and disclosure of official documents; and<br><br>(b) the obligations of public sector bodies under Union or national law to allow the re-use of data or to requirements related to processing of non-personal data.<br><br>Where sector-specific Union or national law requires public sector bodies, data intermediation services providers or recognised data altruism organisations to comply with specific additional technical, administrative or organisational requirements, including through an authorisation or certification regime,

					those provisions of that sector-specific Union or national law shall also apply. Any such specific additional requirements shall be non-discriminatory, proportionate and objectively justified.<br>3. Union and national law on the protection of personal data shall apply to any personal data processed in connection with this Regulation. In particular, this Regulation is without prejudice to Regulations (EU) 2016/679 and (EU) 2018/1725 and Directives 2002/58/EC and (EU) 2016/680, including with regard to the powers and competences of supervisory authorities. In the event of a conflict between this Regulation and Union law on the protection of personal data or national law adopted in accordance with such Union law, the relevant Union or national law on the protection of personal data shall prevail. This Regulation does not create a legal basis for the processing of personal data, nor does it affect any of the rights and obligations set out in Regulations (EU) 2016/679 or (EU) 2018/1725 or Directives 2002/58/EC or (EU) 2016/680.<br>4. This Regulation is without prejudice to the application of competition law.<br>5. This Regulation is without prejudice to the competences of the Member States with regard to their activities concerning public security, defence and national security.<br><br>Article 3 Categories of data<br><br>Article 5 Conditions for re-use
Europe	2021		Regulation (EU) 2022/868 of the European Parliament and of the Council of 30 May 2022 on European data governance and amending Regulation (EU) 2018/1724 (Data Governance Act) (Text with EEA relevance)	Regulation (EU) 2022/868 of the European Parliament and of the Council of 30 May 2022 on European data governance and amending Regulation (EU) 2018/1724 (Data Governance Act) (Text with EEA relevance)	Article 2 Definitions<br>(20) ‘secure processing environment’ means the physical or virtual environment and organisational means to ensure compliance with Union law, such as Regulation (EU) 2016/679, in particular with regard to data subjects’ rights, intellectual property rights, and commercial and statistical confidentiality, integrity and accessibility, as well as with applicable national law, and to allow the entity providing the secure processing environment to determine and supervise all data processing actions, including the display, storage, download and export of data and the calculation of derivative data through computational algorithms;
Europe	2021		Regulation (EU) 2022/868 of the European Parliament and of the Council of 30 May 2022 on European data governance and amending Regulation (EU) 2018/1724 (Data Governance Act) (Text with EEA relevance)	Regulation (EU) 2022/868 of the European Parliament and of the Council of 30 May 2022 on European data governance and amending Regulation (EU) 2018/1724 (Data Governance Act) (Text with EEA relevance)	Article 3 Categories of data<br><br>1. This Chapter applies to data held by public sector bodies which are protected on grounds of:<br>(a) commercial confidentiality, including business, professional and company secrets;<br>(b) statistical confidentiality;<br>(c) the protection of intellectual property rights of third parties; or<br>(d)the protection of personal data, insofar as such data fall outside the scope of Directive (EU) 2019/1024

Europe	2021		Regulation (EU) 2022/868 of the European Parliament and of the Council of 30 May 2022 on European data governance and amending Regulation (EU) 2018/1724 (Data Governance Act) (Text with EEA relevance)	Regulation (EU) 2022/868 of the European Parliament and of the Council of 30 May 2022 on European data governance and amending Regulation (EU) 2018/1724 (Data Governance Act) (Text with EEA relevance)	Article 5 Conditions for re-use<br>1. Public sector bodies which are competent under national law to grant or refuse access for the re-use of one or more of the categories of data referred to in Article 3(1) shall make publicly available the conditions for allowing such re-use and the procedure to request the re-use via the single information point referred to in Article 8. Where they grant or refuse access for re-use, they may be assisted by the competent bodies referred to in Article 7(1).<br>Member States shall ensure that public sector bodies are equipped with the necessary resources to comply with this Article.<br>2. Conditions for re-use shall be non-discriminatory, transparent, proportionate and objectively justified with regard to the categories of data and the purposes of re-use and the nature of the data for which re-use is allowed. Those conditions shall not be used to restrict competition.<br>3. Public sector bodies shall, in accordance with Union and national law, ensure that the protected nature of data is preserved. They may provide for the following requirements:<br>(a) to grant access for the re-use of data only where the public sector body or the competent body, following the request for re-use, has ensured that data has been:<br>(i) anonymised, in the case of personal data; and<br>(ii) modified, aggregated or treated by any other method of disclosure control, in the case of commercially confidential information, including trade secrets or content protected by intellectual property rights;<br>(b) to access and re-use the data remotely within a secure processing environment that is provided or controlled by the public sector body;<br>(c) to access and re-use the data within the physical premises in which the secure processing environment is located in accordance with high security standards, provided that remote access cannot be allowed without jeopardising the rights and interests of third parties.<br>4. In the case of re-use allowed in accordance with paragraph 3, points (b) and (c), the public sector bodies shall impose conditions that preserve the integrity of the functioning of the technical systems of the secure processing environment used. The public sector body shall reserve the right to verify the process, the means and any results of processing of data undertaken by the re-user to preserve the integrity of the protection of the data and reserve the right to prohibit the use of results that contain information jeopardising the rights and interests of third parties. The decision to prohibit the use of the results shall be comprehensible and transparent to the re-user.<br>12. Where justified because of the substantial number of requests across the Union concerning the re-use of non-personal data in specific third countries, the Commission may adopt implementing acts declaring that the legal, supervisory and enforcement arrangements of a third country:<br>(a) ensure protection of intellectual property and trade secrets in a way that is essentially equivalent to the protection ensured under Union law;<br>(b) are being effectively applied and enforced; and<br>(c) provide effective judicial redress.<br>Those implementing acts shall be adopted in accordance with the examination procedure referred to in Article 33(3).<br><br>Article 7 Competent bodies

					1. For the purpose of carrying out the tasks referred to in this Article, each Member State shall designate one or more competent bodies, which may be competent for particular sectors, to assist the public sector bodies which grant or refuse access for the re-use of the categories of data referred to in Article 3(1). Member States may either establish one or more new competent bodies or rely on existing public sector bodies or on internal services of public sector bodies that fulfil the conditions laid down in this Regulation.<br>2. The competent bodies may be empowered to grant access for the re-use of the categories of data referred to in Article 3(1) pursuant to Union or national law which provides for such access to be granted. Where they grant or refuse access for the re-use, Articles 4, 5, 6 and 9 shall apply to those competent bodies.<br>3. The competent bodies shall have adequate legal, financial, technical and human resources to carry out the tasks assigned to them, including the necessary technical knowledge to be able to comply with relevant Union or national law concerning the access regimes for the categories of data referred to in Article 3(1).<br>4. The assistance provided for in paragraph 1 shall include, where necessary:<br>(a) providing technical support by making available a secure processing environment for providing access for the re-use of data;<br>(b) providing guidance and technical support on how to best structure and store data to make that data easily accessible;<br>(c) providing technical support for pseudonymisation and ensuring data processing in a manner that effectively preserves the privacy, confidentiality, integrity and accessibility of the information contained in the data for which re-use is allowed, including techniques for the anonymisation, generalisation, suppression and randomisation of personal data or other state-of-the-art privacy-preserving methods, and the deletion of commercially confidential information, including trade secrets or content protected by intellectual property rights;<br>(d) assisting the public sector bodies, where relevant, to provide support to re-users in requesting consent for re-use from data subjects or permission from data holders in line with their specific decisions, including on the jurisdiction in which the data processing is intended to take place and assisting the public sector bodies in establishing technical mechanisms that allow the transmission of requests for consent or permission from re-users, where practically feasible;<br>(e) providing public sector bodies with assistance in assessing the adequacy of contractual commitments made by a re-user pursuant to Article 5(10).<br>5. Each Member State shall notify the Commission of the identity of the competent bodies designated pursuant to paragraph 1 by 24 September 2023. Each Member State shall also notify the Commission of any subsequent change to the identity of those competent bodies.<br>Article 8 Single information points<br>1. Member States shall ensure that all relevant information concerning the application of Articles 5 and 6 is available and easily accessible through a single information point. Member States shall establish a new body or designate an existing body or structure as the single information point. The single information point may be linked to sectoral, regional or local information points. The functions of the single information point may be automated provided that the public sector body ensures adequate support.<br>2. The single information point shall be competent to receive enquiries or requests for the re-use of the categories of data referred to in Article 3(1) and shall transmit them, where possible and appropriate by automated means, to the competent public sector bodies, or the competent bodies referred to in Article

					7(1), where relevant. The single information point shall make available by electronic means a searchable asset list containing an overview of all available data resources including, where relevant, those data resources that are available at sectoral, regional or local information points, with relevant information describing the available data, including at least the data format and size and the conditions for their re-use.<br>3. The single information point may establish a separate, simplified and well-documented information channel for SMEs and start-ups, addressing their needs and capabilities in requesting the re-use of the categories of data referred to in Article 3(1).<br>4. The Commission shall establish a European single access point offering a searchable electronic register of data available in the national single information points and further information on how to request data via those national single information points.

Europe	2021		Regulation (EU) 2022/868 of the European Parliament and of the Council of 30 May 2022 on European data governance and amending Regulation (EU) 2018/1724 (Data Governance Act) (Text with EEA relevance)	Regulation (EU) 2022/868 of the European Parliament and of the Council of 30 May 2022 on European data governance and amending Regulation (EU) 2018/1724 (Data Governance Act) (Text with EEA relevance)	Article 29 European Data Innovation Board<br><br>1. The Commission shall establish a European Data Innovation Board in the form of an expert group, consisting of representatives of the competent authorities for data intermediation services and the competent authorities for the registration of data altruism organisations of all Member States, the European Data Protection Board, the European Data Protection Supervisor, ENISA, the Commission, the EU SME Envoy or a representative appointed by the network of SME envoys, and other representatives of relevant bodies in specific sectors as well as bodies with specific expertise. In its appointments of individual experts, the Commission shall aim to achieve gender and geographical balance among the members of the expert group.<br><br>Article 30 Tasks of the European Data Innovation Board<br><br>The European Data Innovation Board shall have the following tasks:<br>(a) to advise and assist the Commission with regard to developing a consistent practice of public sector bodies and competent bodies referred to in Article 7(1) in handling requests for the re-use of the categories of data referred to in Article 3(1);<br><br>(b) to advise and assist the Commission with regard to developing a consistent practice for data altruism across the Union;<br><br>(c) to advise and assist the Commission with regard to developing a consistent practice of the competent authorities for data intermediation services and the competent authorities for the registration of data altruism organisations in the application of requirements applicable to data intermediation services providers and recognised data altruism organisations;<br><br>(d) to advise and assist the Commission with regard to developing consistent guidelines on how to best protect, in the context of this Regulation, commercially sensitive non-personal data, in particular trade secrets, but also non-personal data representing content protected by intellectual property rights from unlawful access that risks intellectual property theft or industrial espionage;<br><br>(e) to advise and assist the Commission with regard to developing consistent guidelines for cybersecurity requirements for the exchange and storage of data;<br><br>(f) to advise the Commission, in particular taking into account the input from standardisation organisations, on the prioritisation of cross-sector standards to be used and developed for data use and cross-sector data sharing between emerging common European data spaces, cross-sectoral comparison and exchange of best practices with regard to sectoral requirements for security and access procedures, taking into account sector-specific standardisation activities, in particular clarifying and distinguishing which standards and practices are cross-sectoral and which are sectoral;

					(g) to assist the Commission, in particular taking into account the input from standardisation organisations, in addressing fragmentation of the internal market and the data economy in the internal market by enhancing cross-border, cross-sector interoperability of data as well as data sharing services between different sectors and domains, building on existing European, international or national standards, inter alia with the aim of encouraging the creation of common European data spaces;<br><br>(h) to propose guidelines for common European data spaces, namely purpose- or sector-specific or cross-sectoral interoperable frameworks of common standards and practices to share or jointly process data for, inter alia, the development of new products and services, scientific research or civil society initiatives, such common standards and practices taking into account existing standards, complying with the competition rules and ensuring non-discriminatory access to all participants, for the purpose of facilitating data sharing in the Union and reaping the potential of existing and future data spaces, addressing, inter alia:<br><br>(i) cross-sectoral standards to be used and developed for data use and cross-sector data sharing, cross-sectoral comparison and exchange of best practices with regard to sectoral requirements for security and access procedures, taking into account sector-specific standardisation activities, in particular clarifying and distinguishing which standards and practices are cross-sectoral and which are sectoral;<br><br>(ii) requirements to counter barriers to market entry and to avoid lock-in effects, for the purpose of ensuring fair competition and interoperability;<br><br>(iii) adequate protection for lawful data transfers to third countries, including safeguards against any transfers prohibited by Union law;<br><br>(iv) adequate and non-discriminatory representation of relevant stakeholders in the governance of common European data spaces;<br><br>(v) adherence to cybersecurity requirements in accordance with Union law;<br><br>(i) to facilitate cooperation between Member States with regard to setting harmonised conditions allowing for the re-use of the categories of data referred to in Article 3(1) held by public sector bodies across the internal market;<br><br>(j) to facilitate cooperation between competent authorities for data intermediation services and competent authorities for the registration of data altruism organisations through capacity-building and the exchange of information, in particular by establishing methods for the efficient exchange of information relating to the notification procedure for data intermediation services providers and the registration and monitoring of recognised data altruism organisations, including coordination with regard to the setting of fees or penalties, as well as facilitate cooperation between competent authorities for data intermediation services and competent authorities for the registration of data altruism organisations with regard to international access and transfer of data;

					(k) to advise and assist the Commission with regard to evaluating whether the implementing acts referred to in Article 5(11) and (12) are to be adopted;<br><br>(l) to advise and assist the Commission with regard to developing the European data altruism consent form in accordance with Article 25(1);<br><br>(m) to advise the Commission on improving the international regulatory environment for non-personal data, including standardisation.

Europe	2023		Regulation (EU) 2023/2854 of the European Parliament and of the Council of 13 December 2023 on harmonised rules on fair access to and use of data and amending Regulation (EU) 2017/2394 and Directive (EU) 2020/1828 (Data Act)	Regulation (EU) 2023/2854 of the European Parliament and of the Council of 13 December 2023 on harmonised rules on fair access to and use of data and amending Regulation (EU) 2017/2394 and Directive (EU) 2020/1828 (Data Act)	Article 1 Subject matter and scope<br>This Regulation lays down harmonised rules, inter alia, on:<br>(a) the making available of product data and related service data to the user of the connected product or related service;<br>(b) the making available of data by data holders to data recipients;<br>(c) the making available of data by data holders to public sector bodies, the Commission, the European Central Bank and<br>Union bodies, where there is an exceptional need for those data for the performance of a specific task carried out in<br>the public interest;<br>(d) facilitating switching between data processing services;<br>(e) introducing safeguards against unlawful third-party access to non-personal data; and<br>(f) the development of interoperability standards for data to be accessed, transferred and used.<br>2. This Regulation covers personal and non-personal data, including the following types of data, in the following<br>contexts:<br>(a) Chapter II applies to data, with the exception of content, concerning the performance, use and environment of<br>connected products and related services;<br>(b) Chapter III applies to any private sector data that is subject to statutory data sharing obligations;<br>(c) Chapter IV applies to any private sector data accessed and used on the basis of contract between enterprises;<br>(d) Chapter V applies to any private sector data with a focus on non-personal data;<br>(e) Chapter VI applies to any data and services processed by providers of data processing services;<br>(f) Chapter VII applies to any non-personal data held in the Union by providers of data processing services.<br>3. This Regulation applies to:<br>(a) manufacturers of connected products placed on the market in the Union and providers of related services, irrespective<br>of the place of establishment of those manufacturers and providers;<br>(b) users in the Union of connected products or related services as referred to in point (a);<br>(c) data holders, irrespective of their place of establishment, that make data available to data recipients in the Union;<br>(d) data recipients in the Union to whom data are made available<br>(e) public sector bodies, the Commission, the European Central Bank and Union bodies that request data holders to make<br>data available where there is an exceptional need for those data for the performance of a specific task carried out in the<br>public interest and to the data holders that provide those data in response to such request;<br>(f) providers of data processing services, irrespective of their place of establishment, providing such services to customers<br>in the Union;

					(g) participants in data spaces and vendors of applications using smart contracts and persons whose trade, business or<br>profession involves the deployment of smart contracts for others in the context of executing an agreement<br><br>CHAPTER II BUSINESS TO CONSUMER AND BUSINESS TO BUSINESS DATA SHARING<br>Article 3 Obligation to make product data and related service data accessible to the user<br>1.Connected products shall be designed and manufactured, and related services shall be designed and provided, in such<br>a manner that product data and related service data, including the relevant metadata necessary to interpret and use those<br>data, are, by default, easily, securely, free of charge, in a comprehensive, structured, commonly used and machine-readable<br>format, and, where relevant and technically feasible, directly accessible to the user.<br><br>Article 4 The rights and obligations of users and data holders with regard to access, use and making available product data<br>and related service data<br>1. Where data cannot be directly accessed by the user from the connected product or related service, data holders shall<br>make readily available data, as well as the relevant metadata necessary to interpret and use those data, accessible to the user<br>without undue delay, of the same quality as is available to the data holder, easily, securely, free of charge, in a<br>comprehensive, structured, commonly used and machine-readable format and, where relevant and technically feasible,<br>continuously and in real-time. This shall be done on the basis of a simple request through electronic means where<br>technically feasible.<br>2. Users and data holders may contractually restrict or prohibit accessing, using or further sharing data, if such<br>processing could undermine security requirements of the connected product, as laid down by Union or national law,<br>resulting in a serious adverse effect on the health, safety or security of natural persons. Sectoral authorities may provide<br>users and data holders with technical expertise in that context. Where the data holder refuses to share data pursuant to this<br><br>Article 5 Right of the user to share data with third parties<br>1.Upon request by a user, or by a party acting on behalf of a user, the data holder shall make available readily available<br>data, as well as the relevant metadata necessary to interpret and use those data, to a third party without undue delay, of the

					same quality as is available to the data holder, easily, securely, free of charge to the user, in a comprehensive, structured,<br>commonly used and machine-readable format and, where relevant and technically feasible, continuously and in real-time.<br>The data shall be made available by the data holder to the third party in accordance with Articles 8 and 9<br>Article, it shall notify the competent authority designated pursuant to Article 37<br><br>Article 6 Obligations of third parties receiving data at the request of the user<br>1. A third party shall process the data made available to it pursuant to Article 5 only for the purposes and under the<br>conditions agreed with the user and subject to Union and national law on the protection of personal data including the<br>rights of the data subject insofar as personal data are concerned. The third party shall erase the data when they are no<br>longer necessary for the agreed purpose, unless otherwise agreed with the user in relation to non-personal data.<br><br>CHAPTER VI SWITCHING BETWEEN DATA PROCESSING SERVICES<br>Article 26 Information obligation of providers of data processing services<br>The provider of data processing services shall provide the customer with:<br>(a) information on available procedures for switching and porting to the data processing service, including information on<br>available switching and porting methods and formats as well as restrictions and technical limitations which are known<br>to the provider of data processing services;<br>(b) a reference to an up-to-date online register hosted by the provider of data processing services, with details of all the data<br>structures and data formats as well as the relevant standards and open interoperability specifications, in which the<br>exportable data referred to in Article 25(2), point (e), are available.

Europe	2016		REGULATION (EU) 2016/679 OF THE EUROPEAN PARLIAMENT AND OF THE COUNCIL<br>of 27 April 2016 on the protection of natural persons with regard to the processing of personal data and on the free movement of such data, and<br>repealing Directive 95/46/EC (General Data Protection Regulation)	REGULATION (EU) 2016/679 OF THE EUROPEAN PARLIAMENT AND OF THE COUNCIL<br>of 27 April 2016 on the protection of natural persons with regard to the processing of personal data and on the free movement of such data, and<br>repealing Directive 95/46/EC (General Data Protection Regulation)	Article 1 Subject-matter and objectives<br>1. This Regulation lays down rules relating to the protection of natural persons with regard to the processing of personal data and rules relating to the free movement of personal data.<br>2. This Regulation protects fundamental rights and freedoms of natural persons and in particular their right to the protection of personal data.<br>3. The free movement of personal data within the Union shall be neither restricted nor prohibited for reasons connected with the protection of natural persons with regard to the processing of personal data.
Europe	2016		REGULATION (EU) 2016/679 OF THE EUROPEAN PARLIAMENT AND OF THE COUNCIL of 27 April 2016<br>on the protection of natural persons with regard to the processing of personal data and on the free movement of such data, and<br>repealing Directive 95/46/EC (General Data Protection Regulation)	REGULATION (EU) 2016/679 OF THE EUROPEAN PARLIAMENT AND OF THE COUNCIL of 27 April 2016<br>on the protection of natural persons with regard to the processing of personal data and on the free movement of such data, and<br>repealing Directive 95/46/EC (General Data Protection Regulation)	Article 2 Material scope<br>1. This Regulation applies to the processing of personal data wholly or partly by automated means and to the processing other than by automated means of personal data which form part of a filing system or are intended to form part of a filing system.<br>2. This Regulation does not apply to the processing of personal data:<br>(a) in the course of an activity which falls outside the scope of Union law;<br>(b) by the Member States when carrying out activities which fall within the scope of Chapter 2 of Title V of the TEU;<br>(c) by a natural person in the course of a purely personal or household activity;<br>(d) by competent authorities for the purposes of the prevention, investigation, detection or prosecution of criminal offences or the execution of criminal penalties, including the safeguarding against and the prevention of threats to public security.<br>3. For the processing of personal data by the Union institutions, bodies, offices and agencies, Regulation (EC) No 45/2001 applies. Regulation (EC) No 45/2001 and other Union legal acts applicable to such processing of personal data shall be adapted to the principles and rules of this Regulation in accordance with Article 98.<br>4. This Regulation shall be without prejudice to the application of Directive 2000/31/EC, in particular of the liability rules of intermediary<br>service providers in Articles 12 to 15 of that Directive.
Europe	2016		REGULATION (EU) 2016/679 OF THE EUROPEAN PARLIAMENT AND OF THE COUNCIL<br>of 27 April 2016<br>on the protection of natural persons with regard to the processing	REGULATION (EU) 2016/679 OF THE EUROPEAN PARLIAMENT AND OF THE COUNCIL<br>of 27 April 2016<br>on the protection of natural persons with regard to the processing	Article 4 - Definitions<br>(2) ‘processing’ means any operation or set of operations which is performed on personal data or on sets of personal data, whether or not by automated means, such as collection, recording, organ sation, structuring, storage, adaptation or alteration, retrieval, consultation, use, disclosure by transmission, dissemination or otherwise making available, alignment or combination, restriction, erasure or destruction;

			of personal data and on the free movement of such data, and<br>repealing Directive 95/46/EC (General Data Protection Regulation)	of personal data and on the free movement of such data, and<br>repealing Directive 95/46/EC (General Data Protection Regulation)	
Europe	2016		REGULATION (EU) 2016/679 OF THE EUROPEAN PARLIAMENT AND OF THE COUNCIL<br>of 27 April 2016<br>on the protection of natural persons with regard to the processing<br>of personal data and on the free movement of such data, and<br>repealing Directive 95/46/EC (General Data Protection Regulation)	REGULATION (EU) 2016/679 OF THE EUROPEAN PARLIAMENT AND OF THE COUNCIL<br>of 27 April 2016<br>on the protection of natural persons with regard to the processing<br>of personal data and on the free movement of such data, and<br>repealing Directive 95/46/EC (General Data Protection Regulation)	Article 6 - Lawfulness of processing<br>1. Processing shall be lawful only if and to the extent that at least one of the following applies:<br>(a) the data subject has given consent to the processing of his or her personal data for one or more specific purposes;<br>(b) processing is necessary for the performance of a contract to which the data subject is party or in order to take steps at the request of the data subject prior to entering into a contract;<br>(c) processing is necessary for compliance with a legal obligation to which the controller is subject;<br>(d) processing is necessary in order to protect the vital interests of the data subject or of another natural person;<br>(e) processing is necessary for the performance of a task carried out in the public interest or in the exercise of official authority vested in the controller;<br>(f) processing is necessary for the purposes of the legitimate interests pursued by the controller or by a third party, except where such interests are overridden by the interests or fundamental rights and freedoms of the data subject which require protection of personal data, in particular where the data subject is a child.<br>Point (f) of the first subparagraph shall not apply to processing carried out by public authorities in the performance of their tasks.

Europe	2016		REGULATION (EU) 2016/679 OF THE EUROPEAN PARLIAMENT AND OF THE COUNCIL of 27 April 2016 on the protection of natural persons with regard to the processing of personal data and on the free movement of such data, and repealing Directive 95/46/EC (General Data Protection Regulation)	REGULATION (EU) 2016/679 OF THE EUROPEAN PARLIAMENT AND OF THE COUNCIL of 27 April 2016 on the protection of natural persons with regard to the processing of personal data and on the free movement of such data, and repealing Directive 95/46/EC (General Data Protection Regulation)	Chapter I - Scope and Definitions<br>Article 1: subject matter and scope<br>1. This Regulation lays down rules concerning the placing on the market, making available on the market or putting into service of medical devices for human use and accessories for such devices in the Union. This Regulation also applies to clinical investigations concerning such medical devices and accessories conducted in the Union.<br>CHAPTER II MAKING AVAILABLE ON THE MARKET AND PUTTING INTO SERVICE OF DEVICES, OBLIGATIONS OF ECONOMIC OPERATORS, REPROCESSING, CE MARKING, FREE MOVEMENT<br>Article 5 Placing on the market and putting into service<br>1. A device may be placed on the market or put into service only if it complies with this Regulation when duly supplied and properly installed, maintained and used in accordance with its intended purpose.<br>2. A device shall meet the general safety and performance requirements set out in Annex I which apply to it, taking into account its intended purpose.<br>Article 109 Confidentiality<br>1. Unless otherwise provided for in this Regulation and without prejudice to existing national provisions and practices in the Member States on confidentiality, all parties involved in the application of this Regulation shall respect the confidentiality of information and data obtained in carrying out their tasks in order to protect the following:<br>(a) personal data, in accordance with Article 110;<br>Article 110 Data protection<br>1. Member States shall apply Directive 95/46/EC to the processing of personal data carried out in the Member States pursuant to this Regulation.<br>2. Regulation (EC) No 45/2001 shall apply to the processing of personal data carried out by the Commission pursuant to this Regulation.
Europe	2016		REGULATION (EU) 2016/679 OF THE EUROPEAN PARLIAMENT AND OF THE COUNCIL of 27 April 2016 on the protection of natural persons with regard to the processing of personal data and on the free movement of such data, and repealing Directive 95/46/EC (General Data Protection Regulation)	REGULATION (EU) 2016/679 OF THE EUROPEAN PARLIAMENT AND OF THE COUNCIL of 27 April 2016 on the protection of natural persons with regard to the processing of personal data and on the free movement of such data, and repealing Directive 95/46/EC (General Data Protection Regulation)	Article 10 Processing of personal data relating to criminal convictions and offences<br>Processing of personal data relating to criminal convictions and offences or related security measures based on Article 6(1) shall be carried out<br>only under the control of official authority or when the processing is authorised by Union or Member State law providing for appropriate safeguards for the rights and freedoms of data subjects. Any comprehensive register of criminal convictions shall be kept only under the control of official authority.

Europe	2016		REGULATION (EU) 2016/679 OF THE EUROPEAN PARLIAMENT AND OF THE COUNCIL of 27 April 2016 on the protection of natural persons with regard to the processing of personal data and on the free movement of such data, and repealing Directive 95/46/EC (General Data Protection Regulation)	REGULATION (EU) 2016/679 OF THE EUROPEAN PARLIAMENT AND OF THE COUNCIL of 27 April 2016 on the protection of natural persons with regard to the processing of personal data and on the free movement of such data, and repealing Directive 95/46/EC (General Data Protection Regulation)	Article 15 - right of access by the data subject<br>1. The data subject shall have the right to obtain from the controller confirmation as to whether or not personal data concerning him or her<br>are being processed, and, where that is the case, access to the personal data and the following information:<br>(a) the purposes of the processing;<br>(b) the categories of personal data concerned;<br>(c) the recipients or categories of recipient to whom the personal data have been or will be disclosed, in particular recipients in third countries or international organisations<br>(d) where possible, the envisaged period for which the personal data will be stored, or, if not possible, the criteria used to determine that period;<br>(e) the existence of the right to request from the controller rectification or erasure of personal data or restriction of processing of personal data concerning the data subject or to object to such processing;<br>(f) the right to lodge a complaint with a supervisory authority;<br>(g) where the personal data are not collected from the data subject, any available information as to their source;<br>(h) the existence of automated decision-making, including profiling, referred to in Article 22(1) and (4) and, at least in those cases, meaningful information about the logic involved, as well as the significance and the envisaged consequences of such processing for the data subject.
Europe	2016		REGULATION (EU) 2016/679 OF THE EUROPEAN PARLIAMENT AND OF THE COUNCIL of 27 April 2016 on the protection of natural persons with regard to the processing of personal data and on the free movement of such data, and repealing Directive 95/46/EC (General Data Protection Regulation)	REGULATION (EU) 2016/679 OF THE EUROPEAN PARLIAMENT AND OF THE COUNCIL of 27 April 2016 on the protection of natural persons with regard to the processing of personal data and on the free movement of such data, and repealing Directive 95/46/EC (General Data Protection Regulation)	Article 17 Right to erasure (‘right to be forgotten’)<br>1. The data subject shall have the right to obtain from the controller the erasure of personal data concerning him or her without undue delay<br>and the controller shall have the obligation to erase personal data without undue delay where one of the following grounds applies:<br>(a) the personal data are no longer necessary in relation to the purposes for which they were collected or otherwise processed;<br>(b) the data subject withdraws consent on which the processing is based according to point (a) of Article 6(1), or point (a) of Article 9(2),<br>and where there is no other legal ground for the processing;<br>(c) the data subject objects to the processing pursuant to Article 21(1) and there are no overriding legitimate grounds for the processing, or<br>the data subject objects to the processing pursuant to Article 21(2);<br>(d) the personal data have been unlawfully processed;<br>(e) the personal data have to be erased for compliance with a legal obligation in Union or Member State law to which the controller is subject;<br>(f) the personal data have been collected in relation to the offer of information society services referred to in Article 8(1).

Europe	2016		REGULATION (EU) 2016/679 OF THE EUROPEAN PARLIAMENT AND OF THE COUNCIL<br>of 27 April 2016<br>on the protection of natural persons with regard to the processing<br>of personal data and on the free movement of such data, and<br>repealing Directive 95/46/EC (General Data Protection Regulation)	REGULATION (EU) 2016/679 OF THE EUROPEAN PARLIAMENT AND OF THE COUNCIL<br>of 27 April 2016<br>on the protection of natural persons with regard to the processing<br>of personal data and on the free movement of such data, and<br>repealing Directive 95/46/EC (General Data Protection Regulation)	Article 18 Right to restriction of processing<br>1. The data subject shall have the right to obtain from the controller restriction of processing where one of the following applies:<br>(a) the accuracy of the personal data is contested by the data subject, for a period enabling the controller to verify the accuracy of the<br>personal data;<br>(b) the processing is unlawful and the data subject opposes the erasure of the personal data and requests the restriction of their use instead;<br>(c) the controller no longer needs the personal data for the purposes of the processing, but they are required by the data subject for the establishment, exercise or defence of legal claims;<br>(d) the data subject has objected to processing pursuant to Article 21(1) pending the verification whether the legitimate grounds of the controller override those of the data subject.
Europe	2016		REGULATION (EU) 2016/679 OF THE EUROPEAN PARLIAMENT AND OF THE COUNCIL<br>of 27 April 2016<br>on the protection of natural persons with regard to the processing<br>of personal data and on the free movement of such data, and<br>repealing Directive 95/46/EC (General Data Protection Regulation)	REGULATION (EU) 2016/679 OF THE EUROPEAN PARLIAMENT AND OF THE COUNCIL<br>of 27 April 2016<br>on the protection of natural persons with regard to the processing<br>of personal data and on the free movement of such data, and<br>repealing Directive 95/46/EC (General Data Protection Regulation)	Article 25 Data protection by design and by default<br>1. Taking into account the state of the art, the cost of implementation and the nature, scope, context and purposes of processing as well as the<br>risks of varying likelihood and severity for rights and freedoms of natural persons posed by the processing, the controller shall, both at the time of the determination of the means for processing and at the time of the processing itself, implement appropriate technical and organisational measures, such as pseudonymisation, which are designed to implement data-protection principles, such as data minimisation, in an effective manner and to integrate the necessary safeguards into the processing in order to meet the requirements of this Regulation<br>and protect the rights of data subjects.<br>2. The controller shall implement appropriate technical and organisational measures for ensuring that, by default, only personal data which are necessary for each specific purpose of the processing are processed. That obligation applies to the amount of personal data collected, the extent of their processing, the period of their storage and their accessibility. In particular, such measures shall ensure that by default personal data are not made accessible without the individual's intervention to an indefinite number of natural persons.<br>3. An approved certification mechanism pursuant to Article 42 may be used as an element to demonstrate compliance with the requirements<br>set out in paragraphs 1 and 2 of this Article.
Europe	2016		REGULATION (EU) 2016/679 OF THE EUROPEAN PARLIAMENT AND OF THE COUNCIL<br>of 27 April 2016<br>on the protection of natural persons with regard to the processing	REGULATION (EU) 2016/679 OF THE EUROPEAN PARLIAMENT AND OF THE COUNCIL<br>of 27 April 2016<br>on the protection of natural persons with regard to the processing	Article 27 Representatives of controllers or processors not established in the Union<br>1. Where Article 3(2) applies, the controller or the processor shall designate in writing a representative in the Union.

			of personal data and on the free movement of such data, and<br>repealing Directive 95/46/EC (General Data Protection Regulation)	of personal data and on the free movement of such data, and<br>repealing Directive 95/46/EC (General Data Protection Regulation)	
Europe	2016		REGULATION (EU) 2016/679 OF THE EUROPEAN PARLIAMENT AND OF THE COUNCIL<br>of 27 April 2016<br>on the protection of natural persons with regard to the processing<br>of personal data and on the free movement of such data, and<br>repealing Directive 95/46/EC (General Data Protection Regulation)	REGULATION (EU) 2016/679 OF THE EUROPEAN PARLIAMENT AND OF THE COUNCIL<br>of 27 April 2016<br>on the protection of natural persons with regard to the processing<br>of personal data and on the free movement of such data, and<br>repealing Directive 95/46/EC (General Data Protection Regulation)	Section 2 - security of personal data<br><br>Article 32 - security of processing<br>1. Taking into account the state of the art, the costs of implementation and the nature, scope, context and purposes of processing as well<br>as the risk of varying likelihood and severity for the rights and freedoms of natural persons, the controller and the processor shall implement<br>appropriate technical and organisational measures to ensure a level of security appropriate to the risk, including inter alia as appropriate:<br>(a) the pseudonymisation and encryption of personal data;<br>(b) the ability to ensure the ongoing confidentiality, integrity, availability and resilience of processing systems and services;<br>(c) the ability to restore the availability and access to personal data in a timely manner in the event of a physical or technical incident;<br>(d) a process for regularly testing, assessing and evaluating the effectiveness of technical and organisational measures for ensuring the security of the processing.
Europe	2016		REGULATION (EU) 2016/679 OF THE EUROPEAN PARLIAMENT AND OF THE COUNCIL<br>of 27 April 2016<br>on the protection of natural persons with regard to the processing<br>of personal data and on the free movement of such data, and<br>repealing Directive 95/46/EC (General Data Protection Regulation)	REGULATION (EU) 2016/679 OF THE EUROPEAN PARLIAMENT AND OF THE COUNCIL<br>of 27 April 2016<br>on the protection of natural persons with regard to the processing<br>of personal data and on the free movement of such data, and<br>repealing Directive 95/46/EC (General Data Protection Regulation)	Section 3 - data protection impact assessment and prior consultation<br><br>Article 35 - Data protection impact assessment<br>1. Where a type of processing in particular using new technologies, and taking into account the nature, scope, context and purposes of the processing, is likely to result in a high risk to the rights and freedoms of natural persons, the controller shall, prior to the processing, carry out an assessment of the impact of the envisaged processing operations on the protection of personal data. A single assessment may address a set of similar processing operations that present similar high risks.<br>2. The controller shall seek the advice of the data protection officer, where designated, when carrying out a data protection impact assessment.<br>----- has more paragraphs<br>7. The assessment shall contain at least:<br>(c) an assessment of the risks to the rights and freedoms of data subjects referred to in paragraph 1; and

Europe	2016		REGULATION (EU) 2016/679 OF THE EUROPEAN PARLIAMENT AND OF THE COUNCIL<br>of 27 April 2016<br>on the protection of natural persons with regard to the processing<br>of personal data and on the free movement of such data, and<br>repealing Directive 95/46/EC (General Data Protection Regulation)	REGULATION (EU) 2016/679 OF THE EUROPEAN PARLIAMENT AND OF THE COUNCIL<br>of 27 April 2016<br>on the protection of natural persons with regard to the processing<br>of personal data and on the free movement of such data, and<br>repealing Directive 95/46/EC (General Data Protection Regulation)	CHAPTER V<br>Transfers of personal data to third countries or international organisations<br>Article 44 General principle for transfers<br>Any transfer of personal data which are undergoing processing or are intended for processing after transfer to a third country or to an international organisation shall take place only if, subject to the other provisions of this Regulation, the conditions laid down in this Chapter are complied with by the controller and processor, including for onward transfers of personal data from the third country or an international organisation to another third country or to another international organisation. All provisions in this Chapter shall be applied in order to ensure that the level of protection of natural persons guaranteed by this Regulation is not undermined.
Europe	2016		REGULATION (EU) 2016/679 OF THE EUROPEAN PARLIAMENT AND OF THE COUNCIL<br>of 27 April 2016<br>on the protection of natural persons with regard to the processing<br>of personal data and on the free movement of such data, and<br>repealing Directive 95/46/EC (General Data Protection Regulation)	REGULATION (EU) 2016/679 OF THE EUROPEAN PARLIAMENT AND OF THE COUNCIL<br>of 27 April 2016<br>on the protection of natural persons with regard to the processing<br>of personal data and on the free movement of such data, and<br>repealing Directive 95/46/EC (General Data Protection Regulation)	Section 3 - European data protection board<br>Article 68 European Data Protection Board<br>1. The European Data Protection Board (the 'Board') is hereby established as a body of the Union and shall have legal personality.

Europe	2016		REGULATION (EU) 2016/679<br>OF THE EUROPEAN<br>PARLIAMENT AND OF THE<br>COUNCIL<br>of 27 April 2016<br>on the protection of natural<br>persons with regard to the<br>processing<br>of personal data and on the<br>free movement of such data,<br>and<br>repealing Directive 95/46/EC<br>(General Data Protection<br>Regulation)	REGULATION (EU) 2016/679<br>OF THE EUROPEAN<br>PARLIAMENT AND OF THE<br>COUNCIL<br>of 27 April 2016<br>on the protection of natural<br>persons with regard to the<br>processing<br>of personal data and on the<br>free movement of such data,<br>and<br>repealing Directive 95/46/EC<br>(General Data Protection<br>Regulation)	CHAPTER IX Provisions relating to specific processing situations<br>Article 85<br>Processing and freedom of expression and information<br>1. Member States shall by law reconcile the right to the protection of personal data pursuant to this Regulation with the right to freedom of expression and information, including processing for journalistic purposes and the purposes of academic, artistic or literary expression.<br>2. For processing carried out for journalistic purposes or the purpose of academic artistic or literary expression, Member States shall provide<br>for exemptions or derogations from Chapter II (principles), Chapter III (rights of the data subject), Chapter IV (controller and processor),<br>Chapter V (transfer of personal data to third countries or international organisations), Chapter VI (independent supervisory authorities),<br>Chapter VII (cooperation and consistency) and Chapter IX (specific data processing situations) if they are necessary to reconcile the right<br>to the protection of personal data with the freedom of expression and information.<br><br>Article 88 Processing in the context of employment<br>1. Member States may, by law or by collective agreements, provide for more specific rules to ensure the protection of the rights and<br>freedoms in respect of the processing of employees' personal data in the employment context, in particular for the purposes of the<br>recruitment, the performance of the contract of employment, including discharge of obligations laid down by law or by collective agreements,<br>management, planning and organisation of work, equality and diversity in the workplace, health and safety at work, protection of employer's or<br>customer's property and for the purposes of the exercise and enjoyment, on an individual or collective basis, of rights and benefits related to<br>employment, and for the purpose of the termination of the employment relationship.<br>2. Those rules shall include suitable and specific measures to safeguard the data subject's human dignity, legitimate interests and<br>fundamental rights, with particular regard to the transparency of processing, the transfer of personal data within a group of undertakings,<br>or a group of enterprises engaged in a joint economic activity and monitoring systems at the work place.<br><br>Article 89 Safeguards and derogations relating to processing for archiving purposes in the public interest, scientific or historical research<br>purposes or statistical purposes<br>1. Processing for archiving purposes in the public interest, scientific or historical research purposes or statistical purposes, shall be subject to<br>appropriate safeguards, in accordance with this Regulation, for the rights and freedoms of the data subject. Those safeguards shall ensure that

					technical and organisational measures are in place in particular in order to ensure respect for the principle of data minimisation. Those measures<br>may include pseudonymisation provided that those purposes can be fulfilled in that manner. Where those purposes can be fulfilled by<br>further processing which does not permit or no longer permits the identification of data subjects, those purposes shall be fulfilled in that<br>manner.<br>2. Where personal data are processed for scientific or historical research purposes or statistical purposes, Union or Member State law may provide for derogations from the rights referred to in Articles 15, 16, 18 and 21 subject to the conditions and safeguards referred to in paragraph 1 of this Article in so far as such rights are likely to render impossible or seriously impair the achievement of the specific purposes, and such derogations are necessary for the fulfilment of those purposes.

Europe	2018		REGULATION (EU) 2018/1725 OF THE EUROPEAN PARLIAMENT AND OF THE COUNCIL of 23 October 2018 on the protection of natural persons with regard to the processing of personal data by the Union institutions, bodies, offices and agencies and on the free movement of such data, and repealing Regulation (EC) No 45/2001 and Decision No 1247/2002/EC	REGULATION (EU) 2018/1725 OF THE EUROPEAN PARLIAMENT AND OF THE COUNCIL of 23 October 2018 on the protection of natural persons with regard to the processing of personal data by the Union institutions, bodies, offices and agencies and on the free movement of such data, and repealing Regulation (EC) No 45/2001 and Decision No 1247/2002/EC	CHAPTER I GENERAL PROVISIONS<br>Article 1 Subject matter and objectives<br>1. This Regulation lays down rules relating to the protection of natural persons with regard to the processing of personal<br>data by the Union institutions and bodies and rules relating to the free movement of personal data between them or to other recipients established in the Union.<br>2. This Regulation protects fundamental rights and freedoms of natural persons and in particular their right to the protection of personal data.<br>3. The European Data Protection Supervisor shall monitor the application of the provisions of this Regulation to all processing operations carried out by a Union institution or body.<br><br>CHAPTER II GENERAL PRINCIPLES<br>Article 4 Principles relating to processing of personal data<br>1. Personal data shall be:<br>(a) processed lawfully, fairly and in a transparent manner in relation to the data subject (‘lawfulness, fairness and transparency’);<br>(b) collected for specified, explicit and legitimate purposes and not further processed in a manner that is incompatible with those purposes; further processing for archiving purposes in the public interest, scientific or historical research purposes or statistical purposes shall, in accordance with Article 13, not be considered to be incompatible with the initial purposes (‘purpose limitation’);<br>(c) adequate, relevant and limited to what is necessary in relation to the purposes for which they are processed (‘data minimisation’);<br>(d) accurate and, where necessary, kept up to date; every reasonable step must be taken to ensure that personal data that are inaccurate, having regard to the purposes for which they are processed, are erased or rectified without delay (‘accuracy’);<br>(e) kept in a form which permits identification of data subjects for no longer than is necessary for the purposes for which the personal data are processed; personal data may be stored for longer periods insofar as the personal data will be processed solely for archiving purposes in the public interest, scientific or historical research purposes or statistical purposes in accordance with Article 13 subject to implementation of the appropriate technical and organisational measures required by this Regulation in order to safeguard the rights and freedoms of the data subject (‘storage limitation’);<br>(f) processed in a manner that ensures appropriate security of the personal data, including protection against unauthorised or unlawful processing and against accidental loss, destruction or damage, using appropriate technical or organisational measures (‘integrity and confidentiality’).<br>2. The controller shall be responsible for, and be able to demonstrate compliance with, paragraph 1 (‘accountability’).

Europe	2018		REGULATION (EU) 2018/1725 OF THE EUROPEAN PARLIAMENT AND OF THE COUNCIL of 23 October 2018 on the protection of natural persons with regard to the processing of personal data by the Union institutions, bodies, offices and agencies and on the free movement of such data, and repealing Regulation (EC) No 45/2001 and Decision No 1247/2002/EC	REGULATION (EU) 2018/1725 OF THE EUROPEAN PARLIAMENT AND OF THE COUNCIL of 23 October 2018 on the protection of natural persons with regard to the processing of personal data by the Union institutions, bodies, offices and agencies and on the free movement of such data, and repealing Regulation (EC) No 45/2001 and Decision No 1247/2002/EC	Article 5 Lawfulness of processing<br>Processing shall be lawful only if and to the extent that at least one of the following applies:<br>(a) processing is necessary for the performance of a task carried out in the public interest or in the exercise of official authority vested in the Union institution or body; (b) processing is necessary for compliance with a legal obligation to which the controller is subject; (c) processing is necessary for the performance of a contract to which the data subject is party or in order to take steps at the request of the data subject prior to entering into a contract; (d) the data subject has given consent to the processing of his or her personal data for one or more specific purposes; (e) processing is necessary in order to protect the vital interests of the data subject or of another natural person.<br>2.The basis for the processing referred to in points (a) and (b) of paragraph 1 shall be laid down in Union law.<br>Article 6 Processing for another compatible purpose<br>Where the processing for a purpose other than that for which the personal data have been collected is not based on the data subject’s consent or on Union law which constitutes a necessary and proportionate measure in a democratic society to safeguard the objectives referred to in Article 25(1), the controller shall, in order to ascertain whether processing for another purpose is compatible with the purpose for which the personal data are initially collected, take into account, inter alia:<br>(a) any link between the purposes for which the personal data have been collected and the purposes of the intended further processing;<br>(b) the context in which the personal data have been collected, in particular regarding the relationship between data subjects and the controller;<br>(c) the nature of the personal data, in particular whether special categories of personal data are processed, pursuant to Article 10, or whether personal data related to criminal convictions and offences are processed, pursuant to Article 11;<br>(d) the possible consequences of the intended further processing for data subjects;<br>(e) the existence of appropriate safeguards, which may include encryption or pseudonymisation<br><br>Article 10 Processing of special categories of personal data<br>1. Processing of personal data revealing racial or ethnic origin, political opinions, religious or philosophical beliefs, or<br>trade union membership, and the processing of genetic data, biometric data for the purpose of uniquely identifying a<br>natural person, data concerning health or data concerning a natural person’s sex life or sexual orientation shall be<br>prohibited.<br>2.Paragraph 1 shall not apply if one of the following applies: (see regulation for excemptions)<br><br>Article 13 Safeguards relating to processing for archiving purposes in the public interest, scientific or historical research purposes or statistical purposes<br>Processing for archiving purposes in the public interest, scientific or historical research purposes or statistical purposes, shall be subject to appropriate safeguards, in accordance with this Regulation, for the rights and freedoms of the data subject. Those safeguards shall ensure that technical and organisational

					measures are in place in particular in order to ensure respect for the principle of data minimisation. Those measures may include pseudonymisation provided that those purposes can be fulfilled in that manner. Where those purposes can be fulfilled by further processing which does not permit or no longer permits the identification of data subjects, those purposes shall be fulfilled in that manner.
Europe	2019		Directive (EU) 2019/1024 of the European Parliament and of the Council of 20 June 2019 on open data and the re-use of public sector information (recast)	Directive (EU) 2019/1024 of the European Parliament and of the Council of 20 June 2019 on open data and the re-use of public sector information (recast)	CHAPTER I GENERAL PROVISIONS<br>Article 1 Subject matter and scope<br>1. In order to promote the use of open data and stimulate innovation in products and services, this Directive establishes a set of minimum rules governing the re-use and the practical arrangements for facilitating the re-use of:<br>(a)existing documents held by public sector bodies of the Member States;<br>(b)existing documents held by public undertakings that are:<br>(i)active in the areas defined in Directive 2014/25/EU;<br>(ii)acting as public service operators pursuant to Article 2 of Regulation (EC) No 1370/2007;<br>(iii)acting as air carriers fulfilling public service obligations pursuant to Article 16 of Regulation (EC) No 1008/2008; or<br>(iv)acting as Community shipowners fulfilling public service obligations pursuant to Article 4 of Regulation (EEC) No 3577/92;<br>(c)research data pursuant to the conditions set out in Article 10.<br><br>2. This Directive does not apply to:<br>(a)documents the supply of which is an activity falling outside the scope of the public task of the public sector bodies concerned as defined by law or by other binding rules in the Member State, or, in the

					absence of such rules, as defined in accordance with common administrative practice in the Member State in question, provided that the scope of the public tasks is transparent and subject to review;<br>(b)documents held by public undertakings:<br>(i)produced outside the scope of the provision of services in the general interest as defined by law or other binding rules in the Member State;<br>(ii)related to activities directly exposed to competition and therefore, pursuant to Article 34 of Directive 2014/25/EU, not subject to procurement rules;<br>(c)documents for which third parties hold intellectual property rights;<br>(d)documents, such as sensitive data, which are excluded from access by virtue of the access regimes in the Member state, including on grounds of:<br>(i)the protection of national security (namely, State security), defence, or public security;<br>(ii)statistical confidentiality;<br>(iii)commercial confidentiality (including business, professional or company secrets);<br>(e)documents access to which is excluded or restricted on grounds of sensitive critical infrastructure protection related information as defined in point (d) of Article 2 of Directive 2008/114/EC;<br>(f)documents access to which is restricted by virtue of the access regimes in the Member States, including cases whereby citizens or legal entities have to prove a particular interest to obtain access to documents;<br>(g)logos, crests and insignia;<br>(h)documents, access to which is excluded or restricted by virtue of the access regimes on grounds of protection of personal data, and parts of documents accessible by virtue of those regimes which contain personal data the re-use of which has been defined by law as being incompatible with the law concerning the protection of individuals with regard to the processing of personal data or as undermining the protection of privacy and the integrity of the individual, in particular in accordance with Union or national law regarding the protection of personal data;<br>(i)documents held by public service broadcasters and their subsidiaries, and by other bodies or their subsidiaries for the fulfilment of a public service broadcasting remit;<br>(j)documents held by cultural establishments other than libraries, including university libraries, museums and archives;<br>(k)documents held by educational establishments of secondary level and below, and, in the case of all other educational establishments, documents other than those referred to in point (c) of paragraph 1;<br>(l)documents other than those referred to in point (c) of paragraph 1 held by research performing organisations and research funding organisations, including organisations established for the transfer of research results<br>CHAPTER II REQUESTS FOR RE-USE<br>Article 4 Processing of requests for re-use<br>1. Public sector bodies shall, through electronic means where possible and appropriate, process requests for re-use and shall make the document available for re-use to the applicant or, if a licence is needed, finalise the licence offer to the applicant within a reasonable time that is consistent with the time frames laid down for the processing of requests for access to documents.<br>Chapter 3 - conditions for re-use<br>Article 10 Research data

					1.Member States shall support the availability of research data by adopting national policies and relevant actions aiming at making publicly funded research data openly available (‘open access policies’), following the principle of ‘open by default’ and compatible with the FAIR principles. In that context, concerns relating to intellectual property rights, personal data protection and confidentiality, security and legitimate commercial interests, shall be taken into account in accordance with the principle of ‘as open as possible, as closed as necessary’. Those open access policies shall be addressed to research performing organisations and research funding organisations.<br>2.Without prejudice to point (c) of Article 1(2), research data shall be re-usable for commercial or non-commercial purposes in accordance with Chapters III and IV, insofar as they are publicly funded and researchers, research performing organisations or research funding organisations have already made them publicly available through an institutional or subject-based repository. In that context, legitimate commercial interests, knowledge transfer activities and pre-existing intellectual property rights shall be taken into account.
Europe	2001	2019	DIRECTIVE 2001/29/EC OF THE EUROPEAN PARLIAMENT AND OF THE COUNCIL<br>of 22 May 2001 on the harmonisation of certain aspects of copyright and related rights in the information society	DIRECTIVE 2001/29/EC OF THE EUROPEAN PARLIAMENT AND OF THE COUNCIL<br>of 22 May 2001 on the harmonisation of certain aspects of copyright and related rights in the information society	CHAPTER I<br>OBJECTIVE AND SCOPE<br>Article 1 Scope<br>1. This Directive concerns the legal protection of copyright and related rights in the framework of the internal market, with particular<br>emphasis on the information society.<br>2. Except in the cases referred to in Article 11, this Directive shall leave intact and shall in no way affect existing Community provisions relating to:<br>(a) the legal protection of computer programs;<br>(b) rental right, lending right and certain rights related to copyright in the field of intellectual property;<br>(c) copyright and related rights applicable to broadcasting of programmes by satellite and cable retransmission;<br>(d) the term of protection of copyright and certain related rights;<br>(e) the legal protection of databases.<br><br>Article 2 Reproduction right<br>Member States shall provide for the exclusive right to authorise or prohibit direct or indirect, temporary or permanent reproduction by any means and in any form, in whole or in part:<br>(a) for authors, of their works;<br>(b) for performers, of fixations of their performances;<br>(c) for phonogram producers, of their phonograms;<br>(d) for the producers of the first fixations of films, in respect of the original and copies of their films;<br>(e) for broadcasting organisations, of fixations of their broadcasts, whether those broadcasts are transmitted by wire or over the air, including by cable or satellite.

Europe	2019		DIRECTIVE (EU) 2019/790 OF THE EUROPEAN PARLIAMENT AND OF THE COUNCIL of 17 April 2019 on copyright and related rights in the Digital Single Market and amending Directives 96/9/EC and 2001/29/EC	DIRECTIVE (EU) 2019/790 OF THE EUROPEAN PARLIAMENT AND OF THE COUNCIL of 17 April 2019 on copyright and related rights in the Digital Single Market and amending Directives 96/9/EC and 2001/29/EC	TITLE I GENERAL PROVISIONS<br>Article 1 Subject matter and scope<br>This Directive lays down rules which aim to harmonise further Union law applicable to copyright and related rights in the framework of the internal market, taking into account, in particular, digital and cross-border uses of protected content. It also lays down rules on exceptions and limitations to copyright and related rights, on the facilitation of licences, as well as rules which aim to ensure a well-functioning marketplace for the exploitation of works and other subject matter.
Europe	2020		DIRECTIVE (EU) 2019/790 OF THE EUROPEAN PARLIAMENT AND OF THE COUNCIL of 17 April 2019 on copyright and related rights in the Digital Single Market and amending Directives 96/9/EC and 2001/29/EC	DIRECTIVE (EU) 2019/790 OF THE EUROPEAN PARLIAMENT AND OF THE COUNCIL of 17 April 2019 on copyright and related rights in the Digital Single Market and amending Directives 96/9/EC and 2001/29/EC	TITLE II MEASURES TO ADAPT EXCEPTIONS AND LIMITATIONS TO THE DIGITAL AND CROSS-BORDER<br>ENVIRONMENT<br>Article 3 Text and data mining for the purposes of scientific research<br>1. Member States shall provide for an exception to the rights provided for in Article 5(a) and Article 7(1) of Directive<br>96/9/EC, Article 2 of Directive 2001/29/EC, and Article 15(1) of this Directive for reproductions and extractions made<br>by research organisations and cultural heritage institutions in order to carry out, for the purposes of scientific research,<br>text and data mining of works or other subject matter to which they have lawful access.<br>2. Copies of works or other subject matter made in compliance with paragraph 1 shall be stored with an appropriate<br>level of security and may be retained for the purposes of scientific research, including for the verification of research<br>results.<br>3. Rightholders shall be allowed to apply measures to ensure the security and integrity of the networks and databases<br>where the works or other subject matter are hosted. Such measures shall not go beyond what is necessary to achieve<br>that objective.<br>4. Member States shall encourage rightholders, research organisations and cultural heritage institutions to define<br>commonly agreed best practices concerning the application of the obligation and of the measures referred to in<br>paragraphs 2 and 3 respectively.<br><br>Article 5 Use of works and other subject matter in digital and cross-border teaching activities<br>Member States shall provide for an exception or limitation to the rights provided for in Article 5(a), (b), (d) and (e)<br>and Article 7(1) of Directive 96/9/EC, Articles 2 and 3 of Directive 2001/29/EC, Article 4(1) of Directive 2009/24/EC

					and Article 15(1) of this Directive in order to allow the digital use of works and other subject matter for the sole<br>purpose of illustration for teaching, to the extent justified by the non-commercial purpose to be achieved, on condition<br>that such use:<br>(a) takes place under the responsibility of an educational establishment, on its premises or at other venues, or through<br>a secure electronic environment accessible only by the educational establishment's pupils or students and teaching<br>staff; and<br>(b) is accompanied by the indication of the source, including the author's name, unless this turns out to be impossible.<br>2.Notwithstanding Article 7(1), Member States may provide that the exception or limitation adopted pursuant to<br>paragraph 1 does not apply or does not apply as regards specific uses or types of works or other subject matter, such as<br>material that is primarily intended for the educational market or sheet music, to the extent that suitable licences<br>authorising the acts referred to in paragraph 1 of this Article and covering the needs and specificities of educational<br>establishments are easily available on the market.<br>Member States that decide to avail of the first subparagraph of this paragraph shall take the necessary measures to<br>ensure that the licences authorising the acts referred to in paragraph 1 of this Article are available and visible in an<br>appropriate manner for educational establishments.<br>3. The use of works and other subject matter for the sole purpose of illustration for teaching through secure electronic environments undertaken in compliance with the provisions of national law adopted pursuant to this Article<br>shall be deemed to occur solely in the Member State where the educational establishment is established.<br>4. Member States may provide for fair compensation for rightholders for the use of their works or other subject matter pursuant to paragraph 1.
Europe	2021		DIRECTIVE (EU) 2019/790 OF THE EUROPEAN PARLIAMENT AND OF THE COUNCIL of 17 April 2019 on copyright and related rights in the Digital Single Market and amending Directives 96/9/EC and 2001/29/EC	DIRECTIVE (EU) 2019/790 OF THE EUROPEAN PARLIAMENT AND OF THE COUNCIL of 17 April 2019 on copyright and related rights in the Digital Single Market and amending Directives 96/9/EC and 2001/29/EC	CHAPTER 2 Certain uses of protected content by online services<br>Article 17 Use of protected content by online content-sharing service providers<br>1.Member States shall provide that an online content-sharing service provider performs an act of communication to the public or an act of making available to the public for the purposes of this Directive when it gives the public access to copyright-protected works or other protected subject matter uploaded by its users.

Europe	2021		Regulation (EU) 2021/694 of the European Parliament and of the Council of 29 April 2021 establishing the Digital Europe Programme and repealing Decision (EU) 2015/2240 (Text with EEA relevance)	Regulation (EU) 2021/694 of the European Parliament and of the Council of 29 April 2021 establishing the Digital Europe Programme and repealing Decision (EU) 2015/2240 (Text with EEA relevance)	Article 1 Subject matter<br>This Regulation establishes the Digital Europe Programme (the 'Programme') for the duration of the MFF 2021-2027. This Regulation lays down the objectives of the Programme, its budget for the period 2021 to 2027, the forms of Union funding and the rules for providing such funding.<br>Article 3 Programme objectives<br>1. The general objectives of the Programme shall be to support and accelerate the digital transformation of the European economy, industry and society, to bring its benefits to citizens, public administrations and businesses across the Union, and to improve the competitiveness of Europe in the global digital economy while contributing to bridging the digital divide across the Union and reinforcing the Union's strategic autonomy, through holistic, cross-sectoral and cross-border support and a stronger Union contribution.<br>The Programme shall be implemented in close coordination with other Union programmes as applicable, and shall aim:<br>(a) to strengthen and promote Europe's capacities in key digital technology areas through large-scale deployment;<br>(b) in the private sector and in areas of public interest, to widen the diffusion and uptake of Europe's key digital technologies, promoting the digital transformation and access to digital technologies.<br>2. The Programme shall have five interrelated specific objectives:<br>(a) Specific Objective 1 – High Performance Computing<br>(b) Specific Objective 2 – Artificial Intelligence<br>(c) Specific Objective 3 – Cybersecurity and Trust<br>(d) Specific Objective 4 – Advanced Digital Skills<br>(e) Specific Objective 5 Deployment and Best Use of Digital Capacity and Interoperability.<br>Article 5 Specific Objective 2 – Artificial Intelligence<br>1. The financial contribution from the Union under Specific Objective 2 – Artificial Intelligence shall pursue the following operational objectives:<br>(a) build up and strengthen core AI capacities and knowledge in the Union, including building up and strengthening quality data resources and corresponding exchange mechanisms, and libraries of algorithms, while guaranteeing a human-centric and inclusive approach that respects Union values;<br>(b) make the capacities referred to in point (a) accessible to businesses, especially SMEs and start-ups, as well as civil society, not-for-profit organisations, research institutions, universities and public administrations, in order to maximise their benefit to the European society and economy;<br>(c) reinforce and network AI testing and experimentation facilities in Member States;<br>(d) develop and reinforce commercial application and production systems in order to facilitate the integration of technologies in value chains and the development of innovative business models and to shorten the time required to pass from innovation to commercial exploitation and foster the uptake of AI-based solutions in areas of public interest and in society.<br>AI-based solutions and data made available shall respect the principle of privacy and security by design and shall fully comply with data protection legislation.<br><br>2. The Commission, in accordance with Union and international law, including the Charter, and taking into account, inter alia, the recommendations of the High-Level Expert Group on Artificial Intelligence, shall set

					out ethical requirements in the work programmes under Specific Objective 2. Calls for proposals, calls for tenders and grant agreements shall include the relevant requirements set out in those work programmes. Where appropriate, the Commission shall carry out checks to ensure compliance with those ethical requirements. Funding for actions which do not comply with the ethical requirements may be suspended, terminated or reduced at any time in accordance with the Financial Regulation.<br><br>3. The actions under Specific Objective 2 shall be implemented primarily through direct management. The ethical and legal requirements referred to in this Article shall apply to all actions of Specific Objective 2, regardless of the method of implementation.<br><br>Annex 1 - specific actions<br>Specific Objective 2 – Artificial Intelligence<br>The Programme shall build up and strengthen core AI capacities in Europe, including data resources and repositories of algorithms, and make them accessible to all public administrations and businesses, and shall reinforce and network existing and newly established AI testing and experimentation facilities in Member States.<br>Initial and, where appropriate, subsequent actions under this objective shall include:<br><br>1. The creation of common European data spaces that make accessible data across Europe, including information gathered from the re-use of public sector information, and become a data input source for AI solutions. The spaces should be open to the public and private sectors. For increased usage, data within a space are to be made interoperable, in particular through data formats that are open, machine readable, standardised and documented, both in the interactions between the public and private sectors, within sectors and across sectors (semantic interoperability).<br><br>2. The development of common European libraries or interfaces to libraries of algorithms that make them easily accessible to all potential European users on the basis of fair, reasonable and non-discriminatory terms. Businesses and the public sector are to be able to identify and acquire whichever solution would work best for their needs.<br><br>3. Co-investment with Member States in world class reference facilities for testing and experimentation in real setting focusing on the applications of AI in essential sectors such as health, earth or environment monitoring, transport and mobility, security, manufacturing and finance, as well as in other areas of public interest. Those facilities are to be open to all actors across Europe and connected to the network of European Digital Innovation Hubs. Those facilities are to be equipped with or connected to large computing and data handling facilities, as well as latest AI technologies, including emerging areas such as neuromorphic computing, deep learning and robotics.<br>Annex II measurable indicators to monitor the implementation and to report on the progress of the programme towards the achivement of its specific objectives<br>Specific Objective 2 – Artificial Intelligence<br>2.1.The total amount co-invested in testing and experimentation facilities

					2.2. The usage of common European libraries or interfaces to libraries of algorithms, usage of common European data spaces and usage of testing and experimentation facilities related to actions under this Regulation<br>2.3. The number of cases for which organisations decide to integrate AI in their products, processes or services, as a result of the Programme
Europe	2020		Regulation (EU) 2021/522 of the European Parliament and of the Council of 24 March 2021 establishing a Programme for the Union’s action in the field of health (‘EU4Health Programme’) for the period 2021-2027, and repealing Regulation (EU) No 282/2014 (Text with EEA relevance)	Regulation (EU) 2021/522 of the European Parliament and of the Council of 24 March 2021 establishing a Programme for the Union’s action in the field of health (‘EU4Health Programme’) for the period 2021-2027, and repealing Regulation (EU) No 282/2014 (Text with EEA relevance)	Article 1 Subject matter<br>This Regulation establishes the EU4Health Programme (the ‘Programme’) for the period of the multiannual financial framework 2021 to 2027. The duration of the Programme is aligned with the duration of the multiannual financial framework.<br>This Regulation also lays down the objectives of the Programme, the budget for the period from 2021 to 2027, the forms of Union funding and the rules for providing such funding.<br>Annex<br>6. Actions meeting the objective laid down in point (f) of Article 4<br>(a)Supporting a Union framework and the respective interoperable digital tools for cooperation among Member States and cooperation in networks, including those needed for HTA cooperation;<br>(b)Supporting the deployment, operation and maintenance of mature, secure and interoperable digital service infrastructure and data quality assurance processes for the exchange of, access to, and use and reuse of, data; supporting cross-border networking, including through the use and interoperability of electronic health records, registries and other databases; developing appropriate governance structures and interoperable health information systems;<br>(c)Supporting the digital transformation of healthcare and health systems, including through benchmarking and capacity building, for the uptake of innovative tools and technologies such as artificial intelligence, and supporting the digital upskilling of healthcare professionals;

					(d)Supporting the optimal use of telemedicine and telehealth, including through satellite communication for remote areas, fostering digitally-driven organisational innovation in healthcare facilities and promoting digital tools to support citizen empowerment and patient-centred care;<br>(e)Supporting the development, operation and maintenance of databases and digital tools and their interoperability, including already established projects, where appropriate, with other sensing technologies, such as space-based technologies and artificial intelligence;
Europe	2019		COMMISSION IMPLEMENTING DECISION 2019/1765 of 22 October 2019 providing the rules for the establishment, the management and the functioning of the network of national authorities responsible for eHealth, and repealing Implementing Decision 2011/890/EU	COMMISSION IMPLEMENTING DECISION 2019/1765 of 22 October 2019<br>providing the rules for the establishment, the management and the functioning of the network of national authorities responsible for eHealth, and repealing Implementing Decision 2011/890/EU	Article 1 Subject matter<br>This Decision provides the necessary rules for the establishment, the management and the functioning of the eHealth Network of national authorities responsible for eHealth, as provided for by Article 14 of Directive 2011/24/EU.<br>Article 4 - activities of the eHealth Network<br>1. In pursuing the objective referred to in Article 14(2)(a) of Directive 2011/24/EU the eHealth Network may, in particular:<br>c) provide guidance to Member States and facilitate the exchange of good practices concerning the development of different digital health services, such as telemedicine, m-health, or new technologies in the area of big data and artificial intelligence, taking into consideration ongoing actions at EU level<br>Article 7 Protection of personal data processed through the eHealth Digital Service Infrastructure<br>1. The Member States, represented by the relevant National Authorities or other designated bodies shall be regarded as controllers of personal data they process through the eHealth Digital Service Infrastructure for Cross-Border eHealth Information Services and shall<br>clearly and transparently allocate the responsibilities between controllers.<br>2. The Commission shall be regarded as data processor for patients’ personal data processed through the eHealth Digital Service Infrastructure for Cross-Border eHealth Information Services. In its capacity as processor, the Commission shall manage the core services<br>of the eHealth Digital Service Infrastructure for Cross-Border eHealth Information Services and shall comply with the obligations of a processor laid down in the Annex I to this Decision. The Commission shall not have access to patients’ personal data processed through the eHealth Digital Service Infrastructure for Cross-Border eHealth Information Services.<br>3. The Commission shall be regarded as controller of the processing of personal data necessary to grant and manage access rights to the core services of eHealth Digital Service Infrastructure for Cross-Border eHealth Information Services. Such data are contact details of users,<br>including name, surname and email address and their affiliation.<br><br>Article 7a Cross-border exchange of data between national contact tracing and warning mobile applications through the federation gateway<br>1. Where personal data is exchanged through the federation gateway, the processing shall be limited to the purposes of facilitating the interoperability of national contact tracing and warning mobile applications within the federation gateway and the continuity of contact tracing in a cross-border context.<br>2. The personal data referred to in paragraph 3 shall be transmitted to the federation gateway in a pseudonymised format.

Europe	2018		REGULATION (EU) 2018/1726 OF THE EUROPEAN PARLIAMENT AND OF THE COUNCIL of 14 November 2018 on the European Union Agency for the Operational Management of Large-Scale IT Systems in the Area of Freedom, Security and Justice (eu-LISA), and amending Regulation (EC) No 1987/2006 and Council Decision 2007/533/JHA and repealing Regulation (EU) No 1077/2011	REGULATION (EU) 2018/1726 OF THE EUROPEAN PARLIAMENT AND OF THE COUNCIL of 14 November 2018 on the European Union Agency for the Operational Management of Large-Scale IT Systems in the Area of Freedom, Security and Justice (eu-LISA), and amending Regulation (EC) No 1987/2006 and Council Decision 2007/533/JHA and repealing Regulation (EU) No 1077/2011	CHAPTER I SUBJECT MATTER AND OBJECTIVES<br>Article 1 Subject matter 1.<br>A European Union Agency for the Operational Management of Large-Scale IT Systems in the Area of Freedom, Security and Justice (the Agency) is hereby established.<br>2. The Agency, as established by this Regulation, shall replace and succeed the European Agency for the operational management of large-scale IT systems in the area of freedom, security and justice, as established by Regulation (EU) No 1077/2011.<br><br>3. The Agency shall be responsible for the operational management of the Schengen Information System (SIS II), the Visa Information<br>System (VIS) and Eurodac.<br>4. The Agency shall be responsible for the preparation, development or operational management of the Entry/Exit System (EES), DubliNet,<br>the European Travel Information and Authorisation System (ETIAS), ECRIS-TCN and the ECRIS reference implementation.<br>4a. The Agency shall be responsible for the development and ope ational management, including technical evolutions, of the computerised<br>system for the cross-border electronic exchange of data in the area of judicial cooperation in civil and criminal matters (the ‘e-CODEX system’).<br>4b. The Agency shall be responsible for the development and operational management, including technical evolutions, of the joint investigation teams collaboration platform (‘JITs collaboration platform’).<br>5. The Agency may be made responsible for the preparation, development or operational management of large-scale IT systems in the area<br>of freedom, security and justice other than those referred to in paragraphs 3, 4 and 4a of this Article, including existing systems,<br>only if so provided by relevant Union legal acts governing those systems, based on Articles 67 to 89 TFEU, taking into account,<br>where appropriate, the developments in research referred to in Article 14 of this Regulation and the results of pilot projects and proofs of concept referred to in Article 15 of this Regulation.<br><br>Article 12 Data quality<br>1. Without prejudice to Member States' responsibilities with regard to the data entered into the systems under the Agency's operational responsibility, the Agency, closely involving its Advisory Groups, shall establish for all systems under the Agency's operational responsibility<br>automated data quality control mechanisms and procedures, common data quality indicators and the minimum quality standards to store<br>data, in accordance with the relevant provisions of the legal instruments governing those information systems and of Article 37 of Regulations<br>2019/817 (1) (EU) European Parliament and of the Council. and 2. (EU) 2019/818 (2) of the European Parliament.<br>2. The Agency shall establish a central repository containing only anonymised data for reporting and statistics in accordance with

					Article 39 of Regulations (EU) 2019/817 and (EU) 2019/818, subject to specific provisions in the legal instruments governing the devel<br>opment, establishment, operation and use of large-scale IT systems managed by the Agency.<br><br>Article 14 - Monitoring of research<br>1. The Agency shall monitor developments in research relevant for the operational management of SIS II, VIS, Eurodac, the EES, ETIAS,<br>DubliNet, ECRIS-TCN, the e-CODEX system, the JITs collaboration platform and other large-scale IT systems as referred to in Article 1(5).<br>2. The Agency may contribute to the implementation of the parts of the European Union Framework Programme for Research and Innovation that relate to large-scale IT systems in the area of freedom, security and justice. For that purpose, and where the Commission has delegated the relevant powers to it, the Agency shall have the following tasks:<br>(a) managing some stages of programme implementation and some phases in the lifetime of specific projects on the basis of the relevant work programmes adopted by the Commission;<br>(b) adopting the instruments of budget execution and for revenue and expenditure and carrying out all the operations necessary for the management of the programme; and<br>(c) providing support in programme implementation.<br><br>Article 35 Data protection<br>1. The processing of personal data by the Agency shall be subject to Regulation (EU) 2018/1725.<br>2. The Management Board shall adopt measures for the application of Regulation (EU) 2018/1725 by the Agency, including measures<br>concerning the data protection officer. Those measures shall be adopted after consulting the European Data Protection Supervisor.<br><br>Article 36 Purposes of processing personal data<br>1. The Agency may process personal data only for the following purposes:<br>(a) where necessary for the performance of its tasks related to the operational management of large-scale IT systems entrusted to it under Union law;<br>(b) where necessary for its administrative tasks.<br>2. Where the Agency processes personal data for the purpose referred to in point (a) of paragraph 1 of this Article, Regulation (EU) 2018/1725 shall apply without prejudice to the specific provisions concerning data protection and data security of the Union legal acts governing the development, establishment, operation and use of the systems.

Europe	2022		Directive (EU) 2022/2555 of the European Parliament and of the Council of 14 December 2022 on measures for a high common level of cybersecurity across the Union, amending Regulation (EU) No 910/2014 and Directive (EU) 2018/1972, and repealing Directive (EU) 2016/1148 (NIS 2 Directive) (Text with EEA relevance)	Directive (EU) 2022/2555 of the European Parliament and of the Council of 14 December 2022 on measures for a high common level of cybersecurity across the Union, amending Regulation (EU) No 910/2014 and Directive (EU) 2018/1972, and repealing Directive (EU) 2016/1148 (NIS 2 Directive) (Text with EEA relevance)	Article 1 Subject matter<br>1. This Directive lays down measures that aim to achieve a high common level of cybersecurity across the Union, with a view to improving the functioning of the internal market.<br>2. To that end, this Directive lays down:<br>(a) obligations that require Member States to adopt national cybersecurity strategies and to designate or establish competent authorities, cyber crisis management authorities, single points of contact on cybersecurity (single points of contact) and computer security incident response teams (CSIRTs);<br>(b) cybersecurity risk-management measures and reporting obligations for entities of a type referred to in Annex I or II as well as for entities identified as critical entities under Directive (EU) 2022/2557;<br>(c) rules and obligations on cybersecurity information sharing;<br>(d) supervisory and enforcement obligations on Member States.<br><br>Article 7 National cybersecurity strategy<br>1. Each Member State shall adopt a national cybersecurity strategy that provides for the strategic objectives, the resources required to achieve those objectives, and appropriate policy and regulatory measures, with a view to achieving and maintaining a high level of cybersecurity....<br><br>Article 21 Cybersecurity risk-management measures<br>1. Member States shall ensure that essential and important entities take appropriate and proportionate technical, operational and organisational measures to manage the risks posed to the security of network and information systems which those entities use for their operations or for the provision of their services, and to prevent or minimise the impact of incidents on recipients of their services and on other services.<br>Taking into account the state-of-the-art and, where applicable, relevant European and international standards, as well as the cost of implementation, the measures referred to in the first subparagraph shall ensure a level of security of network and information systems appropriate to the risks posed. When assessing the proportionality of those measures, due account shall be taken of the degree of the entity's exposure to risks, the entity's size and the likelihood of occurrence of incidents and their severity, including their societal and economic impact.<br><br>2. The measures referred to in paragraph 1 shall be based on an all-hazards approach that aims to protect network and information systems and the physical environment of those systems from incidents, and shall include at least the following:<br>(a)policies on risk analysis and information system security;<br>(b)incident handling;<br>(c)business continuity, such as backup management and disaster recovery, and crisis management;<br>(d)supply chain security, including security-related aspects concerning the relationships between each entity and its direct suppliers or service providers;<br>(e)security in network and information systems acquisition, development and maintenance, including vulnerability handling and disclosure;<br>(f)policies and procedures to assess the effectiveness of cybersecurity risk-management measures;<br>(g)basic cyber hygiene practices and cybersecurity training;<br>(h)policies and procedures regarding the use of cryptography and, where appropriate, encryption;

					(i)human resources security, access control policies and asset management;<br>(j)the use of multi-factor authentication or continuous authentication solutions, secured voice, video and text communications and secured emergency communication systems within the entity, where appropriate.<br><br>5. By 17 October 2024, the Commission shall adopt implementing acts laying down the technical and the methodological requirements of the measures referred to in paragraph 2 with regard to DNS service providers, TLD name registries, cloud computing service providers, data centre service providers, content delivery network providers, managed service providers, managed security service providers, providers of online market places, of online search engines and of social networking services platforms, and trust service providers.

Europe	2018		Regulation (EU) 2019/881 of the European Parliament and of the Council of 17 April 2019 on ENISA (the European Union Agency for Cybersecurity) and on information and communications technology cybersecurity certification and repealing Regulation (EU) No 526/2013 (Cybersecurity Act) (Text with EEA relevance)	Regulation (EU) 2019/881 of the European Parliament and of the Council of 17 April 2019 on ENISA (the European Union Agency for Cybersecurity) and on information and communications technology cybersecurity certification and repealing Regulation (EU) No 526/2013 (Cybersecurity Act) (Text with EEA relevance)	Article 1 Subject matter and scope<br><br>1. With a view to ensuring the proper functioning of the internal market while aiming to achieve a high level of cybersecurity, cyber resilience and trust within the Union, this Regulation lays down:<br>(a) objectives, tasks and organisational matters relating to ENISA (the European Union Agency for Cybersecurity); and<br>(b) a framework for the establishment of European cybersecurity certification schemes for the purpose of ensuring an adequate level of cybersecurity for ICT products, ICT services and ICT processes in the Union, as well as for the purpose of avoiding the fragmentation of the internal market with regard to cybersecurity certification schemes in the Union.<br><br><br>Article 9 Knowledge and information<br>ENISA shall:<br>(a)perform analyses of emerging technologies and provide topic-specific assessments on the expected societal, legal, economic and regulatory impact of technological innovations on cybersecurity;<br>(b)perform long-term strategic analyses of cyber threats and incidents in order to identify emerging trends and help prevent incidents;<br>(c)in cooperation with experts from Member States authorities and relevant stakeholders, provide advice, guidance and best practices for the security of network and information systems, in particular for the security of the infrastructures supporting the sectors listed in Annex II to Directive (EU) 2016/1148 and those used by the providers of the digital services listed in Annex III to that Directive;<br>(d)through a dedicated portal, pool, organise and make available to the public information on cybersecurity provided by the Union institutions, bodies, offices and agencies and information on cybersecurity provided on a voluntary basis by Member States and private and public stakeholders;<br>(e) collect and analyse publicly available information regarding significant incidents and compile reports with a view to providing guidance to citizens, organisations and businesses across the Union.<br><br><br>Article 46 European cybersecurity certification framework<br>1. The European cybersecurity certification framework shall be established in order to improve the conditions for the functioning of the internal market by increasing the level of cybersecurity within the Union and enabling a harmonised approach at Union level to European cybersecurity certification schemes, with a view to creating a digital single market for ICT products, ICT services and ICT processes.<br>2. The European cybersecurity certification framework shall provide for a mechanism to establish European cybersecurity certification schemes and to attest that the ICT products, ICT services and ICT processes that have been evaluated in accordance with such schemes comply with specified security requirements for the purpose of protecting the availability, authenticity, integrity or confidentiality of stored or transmitted or processed data or the functions or services offered by, or accessible via, those products, services and processes throughout their life cycle.<br><br>Article 56 Cybersecurity certification

					1. ICT products, ICT services and ICT processes that have been certified under a European cybersecurity certification scheme adopted pursuant to Article 49 shall be presumed to comply with the requirements of such scheme.<br>2. The cybersecurity certification shall be voluntary, unless otherwise specified by Union law or Member State law

Europe	2019		COUNCIL DECISION (CFSP) 2019/797<br>of 17 May 2019<br>concerning restrictive measures against cyber-attacks threatening<br>the Union or its Member States	COUNCIL DECISION (CFSP) 2019/797 of 17 May 2019 concerning restrictive measures against cyber-attacks threatening the Union or its Member States	Article 1<br>1. This Decision applies to cyber-attacks with a significant effect,<br>including attempted cyber-attacks with a potentially significant effect,<br>which constitute an external threat to the Union or its Member States.<br>2. Cyber-attacks constituting an external threat include those which:<br>(a) originate, or are carried out, from outside the Union;<br>(b) use infrastructure outside the Union;<br>(c) are carried out by any natural or legal person, entity or body established or operating outside the Union; or<br>(d) are carried out with the support, at the direction or under the control of any natural or legal person, entity or body operating outside the<br>Union.<br>3. For this purpose, cyber-attacks are actions involving any of the following:<br>(a) access to information systems;<br>(b) information system interference;<br>(c) data interference; or<br>(d) data interception, where such actions are not duly authorised by the owner or by another right holder of the system or data or part of it, or are not permitted under the law of the Union or of the Member State concerned.<br><br>Article 4<br>1. Member States shall take the measures necessary to prevent the entry into, or transit through, their territories of:<br>(a) natural persons who are responsible for cyber-attacks or attempted cyber-attacks;<br>(b) natural persons who provide financial, technical or material support for or are otherwise involved in cyber-attacks or attempted cyber-<br>attacks, including by planning, preparing, participating in, directing, assisting or encouraging such attacks, or facilitating them whether<br>by action or omission;<br>(c) natural persons associated with the persons covered by points (a) and (b), as listed in the Annex.

Europe	2019		REGULATION (EU) 2019/881 OF THE EUROPEAN PARLIAMENT AND OF THE COUNCIL of 17 April 2019 on ENISA (the European Union Agency for Cybersecurity) and on information and communications technology cybersecurity certification and repealing Regulation (EU) No 526/2013 (Cybersecurity Act)	REGULATION (EU) 2019/881 OF THE EUROPEAN PARLIAMENT AND OF THE COUNCIL of 17 April 2019 on ENISA (the European Union Agency for Cybersecurity) and on information and communications technology cybersecurity certification and repealing Regulation (EU) No 526/2013 (Cybersecurity Act)	TITLE I GENERAL PROVISIONS<br>Article 1 Subject matter and scope<br>1. With a view to ensuring the proper functioning of the internal market while aiming to achieve a high level of cybersecurity, cyber resilience and trust within the Union, this Regulation lays down: (a) objectives, tasks and organisational matters relating to ENISA (the European Union Agency for Cybersecurity); and (b) a framework for the establishment of European cybersecurity certification schemes for the purpose of ensuring an adequate level of cybersecurity for ICT products, ICT services and ICT processes in the Union, as well as for the purpose of avoiding the fragmentation of the internal market with regard to cybersecurity certification schemes in the Union. The framework referred to in point (b) of the first subparagraph applies without prejudice to specific provisions in other Union legal acts regarding voluntary or mandatory certification.<br>2. This Regulation is without prejudice to the competences of the Member States regarding activities concerning public security, defence, national security and the activities of the State in areas of criminal law.<br><br>TITLE II ENISA (THE EUROPEAN UNION AGENCY FOR CYBERSECURITY)<br>CHAPTER I Mandate and objectives<br>Article 3 Mandate<br>1. ENISA shall carry out the tasks assigned to it under this Regulation for the purpose of achieving a high common<br>level of cybersecurity across the Union, including by actively supporting Member States, Union institutions, bodies, offices<br>and agencies in improving cybersecurity. ENISA shall act as a reference point for advice and expertise on cybersecurity for<br>Union institutions, bodies, offices and agencies as well as for other relevant Union stakeholders.<br>ENISA shall contribute to reducing the fragmentation of the internal market by carrying out the tasks assigned to it under<br>this Regulation.
Europe	2019		REGULATION (EU) 2019/881 OF THE EUROPEAN PARLIAMENT AND OF THE COUNCIL of 17 April 2019 on ENISA (the European Union Agency for Cybersecurity) and on information and communications technology cybersecurity certification and repealing Regulation (EU) No 526/2013 (Cybersecurity Act)	REGULATION (EU) 2019/881 OF THE EUROPEAN PARLIAMENT AND OF THE COUNCIL of 17 April 2019 on ENISA (the European Union Agency for Cybersecurity) and on information and communications technology cybersecurity certification and repealing Regulation (EU) No 526/2013 (Cybersecurity Act)	Chapter VI - General provisions concerning ENISA<br>Article 41 Protection of personal data<br>1. The processing of personal data by ENISA shall be subject to Regulation (EU) 2018/1725.

Europe	2019		REGULATION (EU) 2019/881 OF THE EUROPEAN PARLIAMENT AND OF THE COUNCIL of 17 April 2019 on ENISA (the European Union Agency for Cybersecurity) and on information and communications technology cybersecurity certification and repealing Regulation (EU) No 526/2013 (Cybersecurity Act)	REGULATION (EU) 2019/881 OF THE EUROPEAN PARLIAMENT AND OF THE COUNCIL of 17 April 2019 on ENISA (the European Union Agency for Cybersecurity) and on information and communications technology cybersecurity certification and repealing Regulation (EU) No 526/2013 (Cybersecurity Act)	TITLE III CYBERSECURITY CERTIFICATION FRAMEWORK<br>Article 46 European cybersecurity certification framework<br>1. The European cybersecurity certification framework shall be established in order to improve the conditions for the functioning of the internal market by increasing the level of cybersecurity within the Union and enabling a harmonised approach at Union level to European cybersecurity certification schemes, with a view to creating a digital single market for ICT products, ICT services and ICT processes<br><br>Article 51 Security objectives of European cybersecurity certification schemes<br>A European cybersecurity certification scheme shall be designed to achieve, as applicable, at least the following security<br>objectives:<br>(a) to protect stored, transmitted or otherwise processed data against accidental or unauthorised storage, processing, access or disclosure during the entire life cycle of the ICT product, ICT service or ICT process;<br>(b) to protect stored, transmitted or otherwise processed data against accidental or unauthorised destruction, loss or alteration or lack of availability during the entire life cycle of the ICT product, ICT service or ICT process;<br>(c) that authorised persons, programs or machines are able only to access the data, services or functions to which their access rights refer;<br>(d) to identify and document known dependencies and vulnerabilities<br>(e) to record which data, services or functions have been accessed, used or otherwise processed, at what times and by whom;<br>(f) to make it possible to check which data, services or functions have been accessed, used or otherwise processed, at what times and by whom;<br>(g) to verify that ICT products, ICT services and ICT processes do not contain known vulnerabilities;<br>(h) to restore the availability and access to data, services and functions in a timely manner in the event of a physical or technical incident;<br>(i) that ICT products, ICT services and ICT processes are secure by default and by design;<br>(j) that ICT products, ICT services and ICT processes are provided with up-to-date software and hardware that do not contain publicly known vulnerabilities, and are provided with mechanisms for secure updates.

Europe	2022		Regulation (EU) 2022/2065 of the European Parliament and of the Council of 19 October 2022 on a Single Market For Digital Services and amending Directive 2000/31/EC (Digital Services Act) (Text with EEA relevance)	Regulation (EU) 2022/2065 of the European Parliament and of the Council of 19 October 2022 on a Single Market For Digital Services and amending Directive 2000/31/EC (Digital Services Act) (Text with EEA relevance)	CHAPTER I GENERAL PROVISIONS<br><br>Article 1 Subject matter<br>1. The aim of this Regulation is to contribute to the proper functioning of the internal market for intermediary services by setting out harmonised rules for a safe, predictable and trusted online environment that facilitates innovation and in which fundamental rights enshrined in the Charter, including the principle of consumer protection, are effectively protected.<br><br>2. This Regulation lays down harmonised rules on the provision of intermediary services in the internal market. In particular, it establishes:<br><br>(a) a framework for the conditional exemption from liability of providers of intermediary services;<br>(b) rules on specific due diligence obligations tailored to certain specific categories of providers of intermediary services;<br>(c) rules on the implementation and enforcement of this Regulation, including as regards the cooperation of and coordination between the competent authorities<br><br>CHAPTER III DUE DILIGENCE OBLIGATIONS FOR A TRANSPARENT AND SAFE ONLINE ENVIRONMENT<br><br>SECTION 1 Provisions applicable to all providers of intermediary services<br><br>Article 13 Legal representatives<br>1. Providers of intermediary services which do not have an establishment in the Union but which offer services in the Union shall designate, in writing, a legal or natural person to act as their legal representative in one of the Member States where the provider offers its services.<br><br><br><br>Article 14 Terms and conditions<br>1. Providers of intermediary services shall include information on any restrictions that they impose in relation to the use of their service in respect of information provided by the recipients of the service, in their terms and conditions. That information shall include information on any policies, procedures, measures and tools used for the purpose of content moderation, including algorithmic decision-making and human review, as well as the rules of procedure of their internal complaint handling system. It shall be set out in clear, plain, intelligible, user-friendly and unambiguous language, and shall be publicly available in an easily accessible and machine-readable format.<br>2. Providers of intermediary services shall inform the recipients of the service of any significant change to the terms and conditions.<br>3. Where an intermediary service is primarily directed at minors or is predominantly used by them, the provider of that intermediary service shall explain the conditions for, and any restrictions on, the use of the service in a way that minors can understand.

					4. Providers of intermediary services shall act in a diligent, objective and proportionate manner in applying and enforcing the restrictions referred to in paragraph 1, with due regard to the rights and legitimate interests of all parties involved, including the fundamental rights of the recipients of the service, such as the freedom of expression, freedom and pluralism of the media, and other fundamental rights and freedoms as enshrined in the Charter.<br>5. Providers of very large online platforms and of very large online search engines shall provide recipients of services with a concise, easily-accessible and machine-readable summary of the terms and conditions, including the available remedies and redress mechanisms, in clear and unambiguous language.<br>6. Very large online platforms and very large online search engines within the meaning of Article 33 shall publish their terms and conditions in the official languages of all the Member States in which they offer their services.<br><br>Article 15 Transparency reporting obligations for providers of intermediary services<br><br>1. Providers of intermediary services shall make publicly available, in a machine-readable format and in an easily accessible manner, at least once a year, clear, easily comprehensible reports on any content moderation that they engaged in during the relevant period. Those reports shall include, in particular, information on the following, as applicable:<br><br>(a)for providers of intermediary services, the number of orders received from Member States' authorities including orders issued in accordance with Articles 9 and 10, categorised by the type of illegal content concerned, the Member State issuing the order, and the median time needed to inform the authority issuing the order, or any other authority specified in the order, of its receipt, and to give effect to the order;<br><br>(b)for providers of hosting services, the number of notices submitted in accordance with Article 16, categorised by the type of alleged illegal content concerned, the number of notices submitted by trusted flaggers, any action taken pursuant to the notices by differentiating whether the action was taken on the basis of the law or the terms and conditions of the provider, the number of notices processed by using automated means and the median time needed for taking the action;<br><br>(c)for providers of intermediary services, meaningful and comprehensible information about the content moderation engaged in at the providers' own initiative, including the use of automated tools, the measures taken to provide training and assistance to persons in charge of content moderation, the number and type of measures taken that affect the availability, visibility and accessibility of information provided by the recipients of the service and the recipients' ability to provide information through the service, and other related restrictions of the service; the information reported shall be categorised by the type of illegal content or violation of the terms and conditions of the service provider, by the detection method and by the type of restriction applied;<br><br>(d) for providers of intermediary services, the number of complaints received through the internal complaint-handling systems in accordance with the provider's terms and conditions and additionally, for providers of online platforms, in accordance with Article 20, the basis for those complaints, decisions taken

					in respect of those complaints, the median time needed for taking those decisions and the number of instances where those decisions were reversed;<br><br>(e) any use made of automated means for the purpose of content moderation, including a qualitative description, a specification of the precise purposes, indicators of the accuracy and the possible rate of error of the automated means used in fulfilling those purposes, and any safeguards applied.<br><br>Section 5 Additional obligations for providers of very large online platforms and of very large online search engines to manage systemic risks<br><br>Article 34 Risk assessment<br>1. Providers of very large online platforms and of very large online search engines shall diligently identify, analyse and assess any systemic risks in the Union stemming from the design or functioning of their service and its related systems, including algorithmic systems, or from the use made of their services. They shall carry out the risk assessments by the date of application referred to in Article 33(6), second subparagraph, and at least once every year thereafter, and in any event prior to deploying functionalities that are likely to have a critical impact on the risks identified pursuant to this Article. This risk assessment shall be specific to their services and proportionate to the systemic risks, taking into consideration their severity and probability, and shall include the following systemic risks:<br>(a)the dissemination of illegal content through their services;<br>(b)any actual or foreseeable negative effects for the exercise of fundamental rights, in particular the fundamental rights to human dignity enshrined in Article 1 of the Charter, to respect for private and family life enshrined in Article 7 of the Charter, to the protection of personal data enshrined in Article 8 of the Charter, to freedom of expression and information, including the freedom and pluralism of the media, enshrined in Article 11 of the Charter, to non-discrimination enshrined in Article 21 of the Charter, to respect for the rights of the child enshrined in Article 24 of the Charter and to a high-level of consumer protection enshrined in Article 38 of the Charter;<br>(c)any actual or foreseeable negative effects on civic discourse and electoral processes, and public security;<br>(d)any actual or foreseeable negative effects in relation to gender-based violence, the protection of public health and minors and serious negative consequences to the person’s physical and mental well-being.<br>2. When conducting risk assessments, providers of very large online platforms and of very large online search engines shall take into account, in particular, whether and how the following factors influence any of the systemic risks referred to in paragraph 1:<br><br>(a)the design of their recommender systems and any other relevant algorithmic system;<br>(b)their content moderation systems;<br>(c)the applicable terms and conditions and their enforcement;<br>(d) systems for selecting and presenting advertisements;<br>(e) data related practices of the provider.

					The assessments shall also analyse whether and how the risks pursuant to paragraph 1 are influenced by intentional manipulation of their service, including by inauthentic use or automated exploitation of the service, as well as the amplification and potentially rapid and wide dissemination of illegal content and of information that is incompatible with their terms and conditions.<br><br>The assessment shall take into account specific regional or linguistic aspects, including when specific to a Member State.<br><br>Article 40 Data access and scrutiny<br>1. Providers of very large online platforms or of very large online search engines shall provide the Digital Services Coordinator of establishment or the Commission, at their reasoned request and within a reasonable period specified in that request, access to data that are necessary to monitor and assess compliance with this Regulation.<br>3. For the purposes of paragraph 1, providers of very large online platforms or of very large online search engines shall, at the request of either the Digital Service Coordinator of establishment or of the Commission, explain the design, the logic, the functioning and the testing of their algorithmic systems, including their recommender systems.

Europe	2019		Regulation (EU) 2019/1150 of the European Parliament and of the Council of 20 June 2019 on promoting fairness and transparency for business users of online intermediation services (Text with EEA relevance)	Regulation (EU) 2019/1150 of the European Parliament and of the Council of 20 June 2019 on promoting fairness and transparency for business users of online intermediation services (Text with EEA relevance)	Article 1 Subject matter and scope<br><br>1. The purpose of this Regulation is to contribute to the proper functioning of the internal market by laying down rules to ensure that business users of online intermediation services and corporate website users in relation to online search engines are granted appropriate transparency, fairness and effective redress possibilities.<br><br>Article 9 Access to data<br>1. Providers of online intermediation services shall include in their terms and conditions a description of the technical and contractual access, or absence thereof, of business users to any personal data or other data, or both, which business users or consumers provide for the use of the online intermediation services concerned or which are generated through the provision of those services.<br>2. Through the description referred to in paragraph 1, providers of online intermediation services shall adequately inform business users in particular of the following:<br><br>(a)whether the provider of online intermediation services has access to personal data or other data, or both, which business users or consumers provide for the use of those services or which are generated through the provision of those services, and if so, to which categories of such data and under what conditions;<br><br>(b)whether a business user has access to personal data or other data, or both, provided by that business user in connection to the business user’s use of the online intermediation services concerned or generated through the provision of those services to that business user and the consumers of the business user’s goods or services, and if so, to which categories of such data and under what conditions;<br><br>(c)in addition to point (b), whether a business user has access to personal data or other data, or both, including in aggregated form, provided by or generated through the provision of the online intermediation services to all of the business users and consumers thereof, and if so, to which categories of such data and under what conditions; and<br><br>(d)whether any data under point (a) is provided to third parties, along with, where the provision of such data to third parties is not necessary for the proper functioning of the online intermediation services, information specifying the purpose of such data sharing, as well as possibilities for business users to opt out from that data sharing.

Europe	2022		Regulation (EU) 2022/1925 of the European Parliament and of the Council of 14 September 2022 on contestable and fair markets in the digital sector and amending Directives (EU) 2019/1937 and (EU) 2020/1828 (Digital Markets Act) (Text with EEA relevance)	Regulation (EU) 2022/1925 of the European Parliament and of the Council of 14 September 2022 on contestable and fair markets in the digital sector and amending Directives (EU) 2019/1937 and (EU) 2020/1828 (Digital Markets Act) (Text with EEA relevance)	Article 1 Subject matter and scope<br><br>1. The purpose of this Regulation is to contribute to the proper functioning of the internal market by laying down harmonised rules ensuring for all businesses, contestable and fair markets in the digital sector across the Union where gatekeepers are present, to the benefit of business users and end users.<br><br>Article 5 Obligations for gatekeepers<br><br>1. The gatekeeper shall comply with all obligations set out in this Article with respect to each of its core platform services listed in the designation decision pursuant to Article 3(9).<br>2. The gatekeeper shall not do any of the following:<br>(a)process, for the purpose of providing online advertising services, personal data of end users using services of third parties that make use of core platform services of the gatekeeper;<br><br>(b)combine personal data from the relevant core platform service with personal data from any further core platform services or from any other services provided by the gatekeeper or with personal data from third-party services;<br><br>(c)cross-use personal data from the relevant core platform service in other services provided separately by the gatekeeper, including other core platform services, and vice versa; and<br><br>(d) sign in end users to other services of the gatekeeper in order to combine personal data,<br><br>unless the end user has been presented with the specific choice and has given consent within the meaning of Article 4, point (11), and Article 7 of Regulation (EU) 2016/679.
Europe	2018		Directive (EU) 2018/1972 of the European Parliament and of the Council of 11 December 2018 establishing the European Electronic Communications Code (Recast)Text with EEA relevance.	Directive (EU) 2018/1972 of the European Parliament and of the Council of 11 December 2018 establishing the European Electronic Communications Code (Recast)Text with EEA relevance.	chapeter 1 subject matter, aim and definition<br>Article 1 Subject matter, scope and aims<br>1. This Directive establishes a harmonised framework for the regulation of electronic communications networks, electronic communications services, associated facilities and associated services, and certain aspects of terminal equipment. It lays down tasks of national regulatory authorities and, where applicable, of other competent authorities, and establishes a set of procedures to ensure the harmonised application of the regulatory framework throughout the Union.<br><br>2. The aims of this Directive are to:<br>(a)implement an internal market in electronic communications networks and services that results in the deployment and take-up of very high capacity networks, sustainable competition, interoperability of electronic communications services, accessibility, security of networks and services and end-user benefits; and<br><br>(b)ensure the provision throughout the Union of good quality, affordable, publicly available services through effective competition and choice, to deal with circumstances in which the needs of end-users, including

					those with disabilities in order to access the services on an equal basis with others, are not satisfactorily met by the market and to lay down the necessary end-user rights.<br><br>3. This Directive is without prejudice to:<br>(a) obligations imposed by national law in accordance with Union law or by Union law in respect of services provided using electronic communications networks and services;<br>(b) measures taken at Union or national level, in accordance with Union law, to pursue general interest objectives, in particular relating to the protection of personal data and privacy, content regulation and audiovisual policy;<br>(c) actions taken by Member States for public order and public security purposes and for defence;<br>(d) Regulations (EU) No 531/2012 and (EU) 2015/2120 and Directive 2014/53/EU.<br><br>4. The Commission, the Body of European Regulators for Electronic Communications ('BEREC') and the authorities concerned shall ensure compliance of their processing of personal data with Union data protection rules.<br><br><br>Title V security<br>Article 40 Security of networks and services<br><br>1. Member States shall ensure that providers of public electronic communications networks or of publicly available electronic communications services take appropriate and proportionate technical and organisational measures to appropriately manage the risks posed to the security of networks and services. Having regard to the state of the art, those measures shall ensure a level of security appropriate to the risk presented. In particular, measures, including encryption where appropriate, shall be taken to prevent and minimise the impact of security incidents on users and on other networks and services.<br>The European Union Agency for Network and Information Security ('ENISA') shall facilitate, in accordance with Regulation (EU) No 526/2013 of the European Parliament and of the Council (45), the coordination of Member States to avoid diverging national requirements that may create security risks and barriers to the internal market.<br><br>2. Member States shall ensure that providers of public electronic communications networks or of publicly available electronic communications services notify without undue delay the competent authority of a security incident that has had a significant impact on the operation of networks or services.<br>In order to determine the significance of the impact of a security incident, where available the following parameters shall, in particular, be taken into account:<br>(a)the number of users affected by the security incident;<br>(b)the duration of the security incident;<br>(c)the geographical spread of the area affected by the security incident;<br>(d)the extent to which the functioning of the network or service is affected.<br>(e)the extent of impact on economic and societal activities.

					Where appropriate, the competent authority concerned shall inform the competent authorities in other Member States and ENISA. The competent authority concerned may inform the public or require the providers to do so, where it determines that disclosure of the security incident is in the public interest.<br>Once a year, the competent authority concerned shall submit a summary report to the Commission and to ENISA on the notifications received and the action taken in accordance with this paragraph.<br>3. Member States shall ensure that in the case of a particular and significant threat of a security incident in public electronic communications networks or publicly available electronic communications services, providers of such networks or services shall inform their users potentially affected by such a threat of any possible protective measures or remedies which can be taken by the users. Where appropriate, providers shall also inform their users of the threat itself.<br>4. This Article is without prejudice to Regulation (EU) 2016/679 and Directive 2002/58/EC.<br>5. The Commission, taking utmost account of ENISA's opinion, may adopt implementing acts detailing the technical and organisational measures referred to in paragraph 1, as well as the circumstances, format and procedures applicable to notification requirements pursuant to paragraph 2. They shall be based on European and international standards to the greatest extent possible, and shall not prevent Member States from adopting additional requirements in order to pursue the objectives set out in paragraph 1.<br>Those implementing acts shall be adopted in accordance with the examination procedure referred to in Article 118(4).<br><br>CHAPTER II General authorisation<br>Section 1 General part<br>Article 12 General authorisation of electronic communications networks and services<br><br>1. Member States shall ensure the freedom to provide electronic communications networks and services, subject to the conditions set out in this Directive. To this end, Member States shall not prevent an undertaking from providing electronic communications networks or services, except where this is necessary for the reasons set out in Article 52(1) TFEU. Any such limitation to the freedom to provide electronic communications networks and services shall be duly reasoned and shall be notified to the Commission.<br><br>2. The provision of electronic communications networks or services, other than number-independent interpersonal communications services, may, without prejudice to the specific obligations referred to in Article 13(2) or rights of use referred to in Articles 46 and 94, be subject only to a general authorisation.<br><br>Tittle III End user rights<br>Article 99 Non-discrimination<br>Providers of electronic communications networks or services shall not apply any different requirements or general conditions of access to, or use of, networks or services to end-users, for reasons related to the end-user's nationality, place of residence or place of establishment, unless such different treatment is objectively justified.<br><br>Article 100 Fundamental rights safeguard

					1. National measures regarding end-users' access to, or use of, services and applications through electronic communications networks shall respect the Charter of Fundamental Rights of the Union (the 'Charter') and general principles of Union law.<br>Article 103 - Transparency, comparison of offers and publication of information<br>4. Member States may require that providers of internet access services or publicly available number-based interpersonal communications services, or both, distribute public interest information free of charge to existing and new end-users, where appropriate, by the means that they ordinarily use in their communications with end-users. In such a case, that public interest information shall be provided by the relevant public authorities in a standardised format and shall, inter alia, cover the following topics:<br>(a) the most common uses of internet access services and publicly available number-based interpersonal communications services to engage in unlawful activities or to disseminate harmful content, in particular where it may prejudice respect for the rights and freedoms of others, including infringements of data protection rights, copyright and related rights, and their legal consequences; and<br>(b) the means of protection against risks to personal security, privacy and personal data when using internet access services and publicly available number-based interpersonal communications services.
Europe	2021		REGULATION (EU) 2021/2282 OF THE EUROPEAN PARLIAMENT AND OF THE COUNCIL of 15 December 2021 on health technology assessment and amending Directive 2011/24/EU	REGULATION (EU) 2021/2282 OF THE EUROPEAN PARLIAMENT AND OF THE COUNCIL of 15 December 2021 on health technology assessment and amending Directive 2011/24/EU	Article 7 Health technologies subject to joint clinical assessments<br>1. The following health technologies shall be subject to joint clinical assessments:<br>(a) medicinal products as referred to in Article 3(1) and Article 3(2), point (a), of Regulation (EC) No 726/2004, for which the application for a marketing authorisation is submitted in accordance with that Regulation after the relevant dates set out in paragraph 2 of this Article, and for which that application is in compliance with Article 8(3) of Directive 2001/83/EC;<br>(b) medicinal products authorised in the Union for which a joint clinical assessment report has been published, in cases where an authorisation is granted pursuant to the second subparagraph of Article 6(1) of Directive 2001/83/EC for a variation to an existing marketing authorisation which corresponds to a new therapeutic indication;<br>(c) medical devices classified as class IIb or III pursuant to Article 51 of Regulation (EU) 2017/745 for which the relevant expert panels have provided a scientific opinion in the framework of the clinical evaluation consultation procedure pursuant to Article 54 of that Regulation, and subject to selection pursuant to paragraph 4 of this Article;<br>(d) in vitro diagnostic medical devices classified as class D pursuant to Article 47 of Regulation (EU) 2017/746 for which the relevant expert panels have provided their views in the framework of the procedure pursuant to Article 48(6) of that Regulation, and subject to selection pursuant to paragraph 4 of this Article.<br>2. The dates referred to in paragraph 1, point (a), shall be as follows:<br>(a) 12 January 2025, for medicinal products with new active substances for which the applicant declares in its application for authorisation submitted to the European Medicines Agency that it contains a new active substance for which the therapeutic indication is the treatment of cancer and medicinal products which are regulated as advanced therapy medicinal products pursuant to Regulation (EC) No 1394/2007 of the European Parliament and of the Council (15);<br>(b) 13 January 2028, for medicinal products which are designated as orphan medicinal products pursuant to Regulation (EC) No 141/2000 of the European Parliament and of the Council (16);

					(c) 13 January 2030, for medicinal products referred to in paragraph 1 other than those referred to in points (a) and (b) of this paragraph.<br>3. By way of derogation from paragraph 2 of this Article, the Commission, upon a recommendation from the Coordination Group, shall adopt a decision by means of an implementing act establishing that medicinal products referred to in that paragraph shall be subject to joint clinical assessment at a date earlier than the dates set out in that paragraph, provided that the medicinal product, in particular according to Article 22, has the potential to address an unmet medical need or a public health emergency or has a significant impact on healthcare systems.<br><br>4. After 12 January 2025, the Commission, after seeking a recommendation from the Coordination Group, shall adopt a decision, by means of an implementing act and at least every two years, selecting the medical devices and in vitro diagnostic medical devices referred to in paragraph 1, points (c) and (d), for joint clinical assessment based on one or more of the following criteria:<br>(a) unmet medical needs;<br>(b) first in class;<br>(c) potential impact on patients, public health or healthcare systems;<br>(d) incorporation of software using artificial intelligence, machine learning technologies or algorithms;<br>(e) significant cross-border dimension;<br>(f) major Union-wide added value.<br>5. The implementing acts referred to in paragraphs 3 and 4 of this Article shall be adopted in accordance with the examination procedure referred to in Article 33(2).

Europe	2017		Regulation (EU) 2017/746 of the European Parliament and of the Council of 5 April 2017 on in vitro diagnostic medical devices and repealing Directive 98/79/EC and Commission Decision 2010/227/EU (Text with EEA relevance.)	Regulation (EU) 2017/746 of the European Parliament and of the Council of 5 April 2017 on in vitro diagnostic medical devices and repealing Directive 98/79/EC and Commission Decision 2010/227/EU (Text with EEA relevance.)	CHAPTER I Scope and definitions<br>Article 1 Subject matter and scope<br>1. This Regulation lays down rules concerning the placing on the market, making available on the market or putting into service of in vitro diagnostic medical devices for human use and accessories for such devices in the Union. This Regulation also applies to performance studies concerning such in vitro diagnostic medical devices and accessories conducted in the Union.<br><br>Article 2 - Definitions<br>(2) ‘in vitro diagnostic medical device’ means any medical device which is a reagent, reagent product, calibrator, control material, kit, instrument,<br>apparatus, piece of equipment, software or system, whether used alone or in combination, intended by the manufacturer to be used in vitro<br>for the examination of specimens, including blood and tissue donations, derived from the human body, solely or principally for the purpose of providing information on one or more of the following:<br>(a) concerning a physiological or pathological process or state;<br>(b) concerning congenital physical or mental impairments;<br>(c) concerning the predisposition to a medical condition or a disease;<br>(d) to determine the safety and compatibility with potential recipients;<br>(e) to predict treatment response or reactions;<br>(f) to define or monitoring therapeutic measures.<br><br>Article 5 Placing on the market and putting into service<br>1. A device may be placed on the market or put into service only if it complies with this Regulation when duly supplied and properly installed, maintained and used in accordance with its intended purpose.<br>2. A device shall meet the general safety and performance requirements set out in Annex I which apply to it, taking into account its intended purpose.<br>3. Demonstration of conformity with the general safety and performance requirements shall include a performance evaluation in accordance with Article 56.<br>4. Devices that are manufactured and used within health institutions, with the exception of devices for performance studies, shall be considered as having been put into service.<br><br>Article 56 Performance evaluation and clinical evidence<br>1. Confirmation of conformity with relevant general safety and performance requirements set out in Annex I, in particular those concerning the performance characteristics referred to in Chapter I and Section 9 of Annex I, under the normal conditions of the intended use of the device, and the evaluation of the interference(s) and cross-reaction(s) and of the acceptability of the benefit-risk ratio referred to in Sections 1 and 8 of Annex I, shall be based on scientific validity, analytical and clinical performance data providing sufficient clinical evidence, including where applicable relevant data as referred to in Annex III.<br>The manufacturer shall specify and justify the level of the clinical evidence necessary to demonstrate conformity with the relevant general safety and performance requirements. That level of clinical evidence shall be appropriate in view of the characteristics of the device and its intended purpose.

Europe	2016		EUROPEAN DATA PROTECTION SUPERVISOR DECISION of 3 December 2015<br>establishing an external advisory group on the ethical dimensions of data protection ('the Ethics Advisory Group')	EUROPEAN DATA PROTECTION SUPERVISOR DECISION of 3 December 2015<br>establishing an external advisory group on the ethical dimensions of data protection ('the Ethics Advisory Group')	Article 1 Subject matter<br>The external advisory group on the ethical dimensions of data protection (the 'Ethics Advisory Group', further: 'the Advisory Group') is hereby established.<br><br>Article 2 Tasks<br>The Advisory Group shall:<br>a) analyse ethical dimensions of data protection;<br>b) submit recommendations to the EDPS upon request;<br>c) submit research suggestions, fostering interdisciplinary cooperation;<br>d) produce at least two public reports;<br>e) involve other experts in its work on a permanent or ad hoc basis, where appropriate, particularly where these other<br>experts may bring additional knowledge and experience not represented in the Advisory Group, including experience<br>in the areas of medicine, health, finance, energy, political governance, police or security;<br>f) present assumptions to a critical audience and measure the outcome of the reflections of the Advisory Group against

					the experience of other practitioners.<br>2. The deliveries of the Advisory Group shall be based on a sound and stable basis in knowledge, empiric evidence<br>and thorough scrutiny.<br>3.The EDPS shall present the results of the Advisory Group to a broad audience in the context of workshops or<br>conferences.
Europe	2021		COMMISSION DECISION (EU) 2021/156 of 9 February 2021<br>renewing the mandate of the European Group on Ethics in Science and New Technologies	COMMISSION DECISION (EU) 2021/156 of 9 February 2021<br>renewing the mandate of the European Group on Ethics in Science and New Technologies	Article 1 Subject matter<br>The European Group on Ethics in Science and New Technologies ('EGE') is set up.<br><br>Article 2 Task<br>The task of the EGE shall be to provide the Commission with independent advice on questions where ethical, societal and<br>fundamental rights dimensions intersect with the development of science and new technologies, either at the request of the<br>Commission or on its own initiative, expressed through its chairperson and agreed with the responsible Commission<br>department.<br>In particular, the EGE shall:<br>(a) identify, define and examine ethical questions raised by developments in science and technologies;<br>(b) provide guidance critical for the development, implementation and monitoring of Union policies or legislation in the<br>form of analyses and recommendations, presented in opinions and statements, that shall be oriented towards the<br>promotion of ethical Union policymaking, in accordance with the Charter of Fundamental Rights of the European<br>Union.

Europe	2022		DECISION (EU) 2022/2481 OF THE EUROPEAN PARLIAMENT AND OF THE COUNCIL of 14 December 2022 establishing the Digital Decade Policy Programme 2030	DECISION (EU) 2022/2481 OF THE EUROPEAN PARLIAMENT AND OF THE COUNCIL of 14 December 2022 establishing the Digital Decade Policy Programme 2030	Article 1 Subject matter<br><br>1. This Decision establishes the Digital Decade Policy Programme 2030 and sets out a monitoring and cooperation mechanism for that programme designated to:<br><br>(a)creating an environment favourable to innovation and investment by setting a clear direction for the digital transformation of the Union and for the delivery of digital targets at Union level by 2030, on the basis of measurable indicators;<br>(b)structuring and stimulating cooperation between the European Parliament, the Council, the Commission and the Member States;<br>(c)fostering the consistency, comparability, transparency and completeness of monitoring and reporting by the Union.<br><br>Article 3 General objectives of the Digital Decade Policy Programme 2030<br>1 The European Parliament, the Council, the Commission and the Member States shall cooperate to support and achieve the following general objectives at Union level (the ‘general objectives’):<br>(a) promoting a human-centred, fundamental-rights-based, inclusive, transparent and open digital environment where secure and interoperable digital technologies and services observe and enhance Union principles, rights and values and are accessible to all, everywhere in the Union;<br>(e) developing a comprehensive and sustainable ecosystem of interoperable digital infrastructures, where high performance, edge, cloud, quantum computing, artificial intelligence, data management and network connectivity work in convergence, to promote their uptake by businesses in the Union, and to create opportunities for growth and jobs through research, development and innovation, and ensuring that the Union has a competitive, secure and sustainable data cloud infrastructure in place, with high security and privacy standards and complying with the Union data protection rules.<br><br>2. In cooperating to achieve the general objectives set out in this Article, the Member States and the Commission shall take account of the digital principles and rights set out in the European Declaration on Digital Rights and Principles for the Digital Decade.<br><br>Article 4 Digital Targets<br>(3)the digital transformation of businesses, where:<br>(a)at least 75 % of Union enterprises have taken up one or more of the following, in line with their business operations:<br>(i)cloud computing services;<br>(ii)big data;<br>(iii)artificial intelligence;

Europe	2021		COUNCIL REGULATION (EU) 2021/1173 of 13 July 2021<br>on establishing the European High Performance Computing Joint Undertaking and repealing Regulation (EU) 2018/1488	COUNCIL REGULATION (EU) 2021/1173 of 13 July 2021<br>on establishing the European High Performance Computing Joint Undertaking and repealing Regulation (EU) 2018/1488	Article 1 Establishment<br>1. For the implementation of the initiative on European High Performance Computing, a Joint Undertaking within the meaning of Article 187 of the Treaty on the Functioning of the European Union (TFEU) (the 'European High Performance Computing Joint Under taking', the 'Joint Undertaking') is hereby established for a period until 31 December 2033.<br><br>Article 3 Mission and objectives<br>1. The mission of the Joint Undertaking shall be: to develop, deploy, extend and maintain in the Union a world-leading federated, secure and<br>hyper-connected supercomputing, quantum computing, service and data infrastructure ecosystem; to support the development and uptake of<br>demand-oriented and user-driven innovative and competitive supercom puting systems based on a supply chain that will ensure components,<br>technologies and knowledge limiting the risk of disruptions and the development of a wide range of applications optimised for these systems; and, to widen the use of that supercomputing infrastructure to a large number of public and private users, and support the twin transition and the development of key skills for European science and industry.<br><br>Article 4 Pillars of activity<br>1. The Joint Undertaking shall implement the mission referred to in Article 3 according to the following pillars of activities:<br>(d) technology pillar, addressing ambitious research and innovation activities for developing a world-class, competitive and innovative supercomputing ecosystem across Europe addressing hardware and software technologies, and their integration into computing systems, covering the whole scientific and industrial value chain, for contributing to the Union's strategic autonomy; it shall also focus on energy-efficient High Performance Computing technologies, contributing to environmental sustainability; those activities shall address inter alia:<br>(i) low-power micro-processing components, interconnection components, system architecture and related technologies such as novel algorithms, software codes, tools, and environments;<br>(ii) emerging computing paradigms and their integration into leading supercomputing systems through a co-design approach; these technologies shall be linked with the development, acquisition and deployment of high-end supercomputers, including quantum computers, and infrastructures;<br>(iii) technologies and systems for the interconnection and operation of classical supercomputing systems with other, often complementary computing technologies, such as quantum computing or other emerging computing technologies and ensure their effective operation;<br>(iv) new algorithms and software technologies that offer substantial performance increases;<br><br>Article 31 Processing of personal data<br>Where the implementation of this Regulation requires the processing of personal data, they shall be processed in accordance with Regulation<br>(EU) 2018/1725 of the European Parliament and of the Council (7).

Europe	2021		REGULATION (EU) 2021/819 OF THE EUROPEAN PARLIAMENT AND OF THE COUNCIL<br>of 20 May 2021<br>on the European Institute of Innovation and Technology	REGULATION (EU) 2021/819 OF THE EUROPEAN PARLIAMENT AND OF THE COUNCIL<br>of 20 May 2021<br>on the European Institute of Innovation and Technology	Article 1 Subject matter<br>This Regulation establishes the European Institute of Innovation and Technology (EIT)<br><br>Article 3 Mission and objectives<br>1. The EIT's mission is to contribute to sustainable Union economic growth and competitiveness by reinforcing the<br>innovation capacity of the Union and Member States in order to address major challenges faced by society. It shall do this<br>by promoting synergies, integration and cooperation among higher education, research and innovation of the highest<br>standards, including by fostering entrepreneurship, thereby strengthening the innovation ecosystems across the Union in<br>an open and transparent manner. The EIT shall also deliver on the Union strategic priorities and contribute to the<br>realisation of Union objectives and policies, including the European Green Deal, the European Recovery Plan, the<br>European strategy for data, the SME Strategy for a sustainable and digital Europe and the New Industrial Strategy for<br>Europe and those related to achieving the Union's strategic autonomy, while retaining an open economy. Furthermore, it<br>shall contribute to tackling global challenges, including the SDGs by following the principles of the 2030 Agenda and the<br>Paris Agreement, and to achieving a net-zero greenhouse gas economy by 2050 at the latest.
Europe	2021		Regulation (EU) 2021/695 of the European Parliament and of the Council of 28 April 2021 establishing Horizon Europe – the Framework Programme for Research and Innovation, laying down its rules for participation and dissemination, and repealing Regulations (EU) No 1290/2013 and (EU) No 1291/2013 (Text with EEA relevance)	Regulation (EU) 2021/695 of the European Parliament and of the Council of 28 April 2021 establishing Horizon Europe – the Framework Programme for Research and Innovation, laying down its rules for participation and dissemination, and repealing Regulations (EU) No 1290/2013 and (EU) No 1291/2013 (Text with EEA relevance)	Article 1 Subject matter<br>1. This Regulation establishes Horizon Europe - the Framework Programme for Research and Innovation (the 'Programme') for the duration of the MFF 2021-2027, sets out the rules for participation and dissemination concerning indirect actions under the Programme and determines the framework governing Union support for R&I activities for the same duration.<br><br>- artifical intelligence within cluster 1 (health) and cluster 4 (digital industry and space) mentioned
Europe	2021		Regulation (EU) 2021/695 of the European Parliament and of the Council of 28 April 2021 establishing Horizon Europe – the Framework Programme for	Regulation (EU) 2021/695 of the European Parliament and of the Council of 28 April 2021 establishing Horizon Europe – the Framework Programme	Article 3 Programme objectives<br><br>1. The general objective of the Programme is to deliver scientific, technological, economic and societal impact from the Union's investments in R&I so as to strengthen the scientific and technological bases of the Union and foster the competitiveness of the Union in all Member States including in its industry, to

			Research and Innovation, laying down its rules for participation and dissemination, and repealing Regulations (EU) No 1290/2013 and (EU) No 1291/2013 (Text with EEA relevance)	for Research and Innovation, laying down its rules for participation and dissemination, and repealing Regulations (EU) No 1290/2013 and (EU) No 1291/2013 (Text with EEA relevance)	deliver on the Union strategic priorities and to contribute to the realisation of Union objectives and policies, to tackle global challenges, including the SDGs by following the principles of the 2030 Agenda and the Paris Agreement, and to strengthen the ERA. The Programme shall thus maximise Union added value by focusing on objectives and activities that cannot be effectively realised by Member States acting alone, but in cooperation.
Europe	2021		Regulation (EU) 2021/695 of the European Parliament and of the Council of 28 April 2021 establishing Horizon Europe – the Framework Programme for Research and Innovation, laying down its rules for participation and dissemination, and repealing Regulations (EU) No 1290/2013 and (EU) No 1291/2013 (Text with EEA relevance)	Regulation (EU) 2021/695 of the European Parliament and of the Council of 28 April 2021 establishing Horizon Europe – the Framework Programme for Research and Innovation, laying down its rules for participation and dissemination, and repealing Regulations (EU) No 1290/2013 and (EU) No 1291/2013 (Text with EEA relevance)	Article 19 Ethics<br><br>1. Actions carried out under the Programme shall comply with ethical principles and relevant Union, national and international law, including the Charter and the European Convention for the Protection of Human Rights and Fundamental Freedoms and its Supplementary Protocols.<br>Particular attention shall be paid to the principle of proportionality, to the right to privacy, the right to the protection of personal data, the right to the physical and mental integrity of a person, the right to non-discrimination and to the need to ensure protection of the environment and high levels of human health protection.<br>2. Legal entities participating in an action shall provide:<br><br>(a) an ethics self-assessment identifying and detailing all the foreseeable ethics issues related to the objective, implementation and likely impact of the activities to be funded, including a confirmation of compliance with paragraph 1 and a description of how it will be ensured;<br><br>(b) a confirmation that the activities will comply with the European Code of Conduct for Research Integrity published by All European Academies and that no activities excluded from funding will be conducted;<br><br>(c) for activities carried out outside the Union, a confirmation that the same activities would have been allowed in a Member State; and<br><br>(d) for activities making use of human embryonic stem cells, as appropriate, details of licensing and control measures that shall be taken by the competent authorities of the Member States concerned as well as details of the ethics approvals that shall be obtained before the activities concerned start.<br><br>3. Proposals shall be systematically screened to identify actions which raise complex or serious ethics issues and submit them to an ethics assessment. The ethics assessment shall be carried out by the Commission unless it is delegated to the funding body. All actions involving the use of human embryonic stem cells or human embryos shall be subject to an ethics assessment. Ethics screenings and assessments shall be carried out with the support of ethics experts. The Commission and the funding bodies shall ensure the transparency of the ethics procedures without prejudice to the confidentiality of the content of those procedures.

					4. Legal entities participating in an action shall obtain all approvals or other mandatory documents from the relevant national, local ethics committees or other bodies, such as data protection authorities, before the start of the relevant activities. Those documents shall be kept on file and provided to the Commission or the relevant funding body upon request.<br><br>5. If appropriate, ethics checks shall be carried out by the Commission or the relevant funding body. For serious or complex ethics issues, ethics checks shall be carried out by the Commission unless the Commission delegates this task to the funding body.<br>Ethics checks shall be carried out with the support of ethics experts.<br><br>6. Actions which do not fulfil the ethics requirements referred to in paragraphs 1 to 4 and are therefore not ethically acceptable, shall be rejected or terminated once the ethical unacceptability has been established.
Europe	2021		Regulation (EU) 2021/695 of the European Parliament and of the Council of 28 April 2021 establishing Horizon Europe – the Framework Programme for Research and Innovation, laying down its rules for participation and dissemination, and repealing Regulations (EU) No 1290/2013 and (EU) No 1291/2013 (Text with EEA relevance)	Regulation (EU) 2021/695 of the European Parliament and of the Council of 28 April 2021 establishing Horizon Europe – the Framework Programme for Research and Innovation, laying down its rules for participation and dissemination, and repealing Regulations (EU) No 1290/2013 and (EU) No 1291/2013 (Text with EEA relevance)	Annex I Broad Lines of Activities<br>d) Cluster 'Digital, Industry and Space': reinforcing capacities and securing Europe's sovereignty in key enabling technologies for digitisation and production, and in space technology, all along the value chain; to build a competitive, digital, low-carbon and circular industry; ensure a sustainable supply of raw materials; develop advanced materials and provide the basis for advances and innovation in global societal challenges.<br><br>Areas of intervention: manufacturing technologies; key digital technologies, including quantum technologies; emerging enabling technologies; advanced materials; artificial intelligence and robotics; next generation internet; advanced computing and Big Data; circular industries; low carbon and clean industries; space, including earth observation.

Europe	2021		Regulation (EU) 2021/695 of the European Parliament and of the Council of 28 April 2021 establishing Horizon Europe – the Framework Programme for Research and Innovation, laying down its rules for participation and dissemination, and repealing Regulations (EU) No 1290/2013 and (EU) No 1291/2013 (Text with EEA relevance)	Regulation (EU) 2021/695 of the European Parliament and of the Council of 28 April 2021 establishing Horizon Europe – the Framework Programme for Research and Innovation, laying down its rules for participation and dissemination, and repealing Regulations (EU) No 1290/2013 and (EU) No 1291/2013 (Text with EEA relevance)	Annex IV Synergies with other union programmes<br>7. Synergies with the Digital Europe Programme (DEP) shall ensure that:<br><br>(a) whereas several thematic areas addressed by the Programme and DEP converge, the type of actions to be supported, their expected results and their intervention logic are different and complementary;<br><br>(b) the R&I needs related to digital aspects of the Programme are identified and established through its strategic planning; this includes, for example, R&I for high performance computing, artificial intelligence, cybersecurity, distributed ledger technologies, quantum technologies combining digital with other enabling technologies and non-technological innovations; support for the scale-up of companies introducing breakthrough innovations (many of which combine digital and physical technologies); and support to digital research infrastructures;<br><br>(c) DEP focuses on large-scale digital capacity and infrastructure building in, for example, high performance computing, artificial intelligence, cybersecurity, distributed ledger technologies, quantum technologies and advanced digital skills aiming at wide uptake and deployment across the Union of critical existing or tested innovative digital solutions within a Union framework in areas of public interest (such as health, public administration, justice and education) or market failure (such as the digitisation of businesses, in particular SMEs); DEP is mainly implemented through coordinated and strategic investments with Member States, in particular through joint public procurement, in digital capacities to be shared across the Union and in Union-wide actions that support interoperability and standardisation as part of developing the Digital Single Market;<br><br>(d) DEP capacities and infrastructures are made available to the R&I community, including for activities supported under the Programme including testing, experimentation and demonstration across all sectors and disciplines;<br><br>(e)novel digital technologies developed through the Programme are to be progressively taken up and deployed by DEP;<br><br>(f) the Programme's initiatives for the development of skills and competencies curricula, including those delivered at the relevant EIT KICs, are complemented by DEP supported capacity-building in advanced digital skills;<br><br>(g) strong coordination mechanisms for strategic programming, operating procedures and governance structures exist for both programmes.

Europe	2021		Regulation (EU) 2021/695 of the European Parliament and of the Council of 28 April 2021 establishing Horizon Europe – the Framework Programme for Research and Innovation, laying down its rules for participation and dissemination, and repealing Regulations (EU) No 1290/2013 and (EU) No 1291/2013 (Text with EEA relevance)	Regulation (EU) 2021/695 of the European Parliament and of the Council of 28 April 2021 establishing Horizon Europe – the Framework Programme for Research and Innovation, laying down its rules for participation and dissemination, and repealing Regulations (EU) No 1290/2013 and (EU) No 1291/2013 (Text with EEA relevance)	ANNEX VI AREAS FOR POSSIBLE MISSIONS AND AREAS FOR POSSIBLE INSTITUTIONALISED EUROPEAN PARTNERSHIPS TO BE ESTABLISHED UNDER ARTICLE 185 OR 187 TFEU<br><br>In accordance with Articles 8 and 12 of this Regulation, the areas for possible missions and possible European Partnerships to be established under Article 185 or 187 TFEU are set out in this Annex.<br><br>I. Areas for possible missions:<br><br>—Missions Area 1: Adaptation to Climate Change, including Societal Transformation.<br>—Mission Area 2: Cancer.<br>—Mission Area 3: Healthy Oceans, Seas, Coastal and Inland Waters.<br>—Mission Area 4: Climate-Neutral and Smart Cities.<br>—Mission Area 5: Soil Health and Food.<br><br>Each mission follows the principles set out in Article 8(4) of this Regulation.<br><br>II. Areas for possible Institutionalised European Partnerships on the basis of Article 185 or 187 TFEU:<br><br>—Partnership Area 1: Faster development and safer use of health innovations for European patients, and global health.<br>—Partnership Area 2: Advancing key digital and enabling technologies and their use, including but not limited to novel technologies such as artificial intelligence, photonics and quantum technologies.<br>—Partnership Area 3: European leadership in Metrology including an integrated Metrology system.<br>—Partnership Area 4: Accelerate competitiveness, safety and environmental performance of Union air traffic, aviation and rail.<br>—Partnership Area 5: Sustainable, inclusive and circular bio-based solutions.<br>—Partnership Area 6: Hydrogen and sustainable energy storage technologies with lower environmental footprint and less energy-intensive production.<br>—Partnership Area 7: Clean, connected, cooperative, autonomous and automated solutions for future mobility demands of people and goods.<br>—Partnership Area 8: Innovative and R&D intensive SMEs.
Europe	2021		REGULATION (EU) 2021/523 OF THE EUROPEAN PARLIAMENT AND OF THE COUNCIL of 24 March 2021 establishing the InvestEU Programme and amending Regulation (EU) 2015/1017	REGULATION (EU) 2021/523 OF THE EUROPEAN PARLIAMENT AND OF THE COUNCIL of 24 March 2021 establishing the InvestEU Programme and amending Regulation (EU) 2015/1017	Annex II - Areas eligible for finance and investment operations<br>(6) the development, deployment and scaling-up of digital technologies and services, especially digital technologies and<br>services, including media, online service platforms and secure digital communication, that contribute to the objectives of the Digital Europe Programme, in particular through:<br>(a) artificial intelligence;

Europe	2021		COUNCIL REGULATION (EU) 2021/2085 of 19 November 2021 establishing the Joint Undertakings under Horizon Europe and<br>repealing Regulations (EC) No 219/2007, (EU) No 557/2014, (EU) No 558/2014, (EU) No 559/2014, (EU) No 560/2014, (EU) No 561/2014 and (EU) No 642/2014	COUNCIL REGULATION (EU) 2021/2085 of 19 November 2021 establishing the Joint Undertakings under Horizon Europe and<br>repealing Regulations (EC) No 219/2007, (EU) No 557/2014, (EU) No 558/2014, (EU) No 559/2014, (EU) No 560/2014, (EU) No 561/2014 and (EU) No 642/2014	Article 1 Subject matter<br>This Regulation sets up nine joint undertakings within the meaning of Article 187 TFEU for the implementation of institutionalised European<br>partnerships referred to in Article 10(1), point (c), of the Horizon Europe Regulation. It determines their objectives and tasks, membership, organisation and other operating rules.<br><br>Article 3 Establishment<br>1. The following joint undertakings are set up as Union bodies for a period ending on 31 December 2031 and financed under the MFF 2021-<br>2027:<br>(f) the Innovative Health Initiative Joint Undertaking;<br><br>Article 4 - objectives and principles<br>2. The joint undertakings shall, through the involvement and<br>commitment of partners in designing and implementing a programme<br>of research and innovation activities with European added value, deliver<br>collectively on the following general objectives:<br>(a) strengthening and integrating the scientific, innovation and tech<br>nological capacities and facilitating collaborative links across the<br>Union to support the creation and diffusion of high-quality new<br>knowledge and skills, in particular with a view to delivering on<br>global challenges, securing and enhancing Union competitiveness,<br>European added value, resilience and sustainability and contributing<br>to a reinforced European Research Area (ERA);<br>(b) securing sustainability-driven global leadership and resilience of<br>Union value chains in key technologies and industries in line with<br>the industrial and SMEs strategies for Europe, the European Green<br>Deal, the European Recovery Plan and other relevant Union<br>policies;<br>(c) developing and accelerating the uptake of innovative solutions<br>throughout the Union addressing climate, environmental, health,<br>digital and other global challenges contributing to Union strategic<br>priorities, accelerating the economic growth of the Union and<br>fostering the innovation ecosystem, while reaching the United<br>Nations Sustainable Development Goals and achieving climate<br>neutrality in the Union at the latest by 2050, in line with the<br>Paris Agreement, thereby improving the quality of life of<br>European citizens.<br><br><br>Article 35 Processing of personal data

					Where the implementation of this Regulation requires the processing of personal data this shall be processed in accordance with Regulation (EU) 2018/1725 of the European Parliament and of the Council (9).<br><br>Article 115 - Additional objectives of the Innovative Health Joint undertaking<br>2. The Innovative Health Initiative Joint Undertaking shall also have the following specific objectives:<br>(a) contribute towards a better understanding of the determinants of health and priority disease areas;<br>(b) integrate fragmented health research and innovation efforts bringing together health industry sectors and other stakeholders, focusing on<br>unmet public health needs, to enable the development of tools, data, platforms, technologies and processes for improved prediction,<br>prevention, interception, diagnosis, treatment and management of diseases, meeting the needs of end-users;<br>(c) demonstrate the feasibility of people-centred integrated health care solutions;<br>(d) exploit the full potential of digitalisation and data exchange in health care;<br>(e) enable the development of new and improved methodologies and models for a comprehensive assessment of the added value of innovative and integrated health care solutions.

Europe	2021		DECISION (EU) 2021/820 OF THE EUROPEAN PARLIAMENT AND OF THE COUNCIL of 20 May 2021 on the Strategic Innovation Agenda of the European Institute of Innovation and Technology (EIT) 2021-2027: Boosting the Innovation Talent and Capacity of Europe and repealing Decision No 1312/2013/EU	DECISION (EU) 2021/820 OF THE EUROPEAN PARLIAMENT AND OF THE COUNCIL of 20 May 2021 on the Strategic Innovation Agenda of the European Institute of Innovation and Technology (EIT) 2021-2027: Boosting the Innovation Talent and Capacity of Europe and repealing Decision No 1312/2013/EU	Article 1<br>The Strategic Innovation Agenda of the European Institute of Innovation and Technology for the period from 2021 to 2027 (SIA 2021-2027) as set out in the Annex is hereby adopted.<br><br>Mentioned within the rational " The role of Europe as a global actor includes the need to enhance the dissemination of European cultural content. Europe needs to remain competitive in the global digital race for the creation of new technologies (such as Artificial<br>Intelligence, the Internet of Things and blockchain) for which CCSI are important generators of content, products and<br>services. Moreover, on a global scale, CCSI (such as design and architecture) contribute actively to the sustainable<br>development and drive green innovation, while cultural content (literature, film and the arts) can, in addition to its<br>intrinsic value, raise awareness of ecological problems and inform public opinion"
Europe	2022		COUNCIL DECISION (CFSP) 2022/2269<br>of 18 November 2022<br>on Union support for the implementation of a project 'Promoting Responsible Innovation in<br>Artificial Intelligence for Peace and Security'	COUNCIL DECISION (CFSP) 2022/2269<br>of 18 November 2022<br>on Union support for the implementation of a project 'Promoting Responsible Innovation in<br>Artificial Intelligence for Peace and Security'	Article 1 With a view to the implementation of the 'Global Strategy for the European Union's Foreign And Security Policy', and<br>taking into account the Union's Strategy against illicit firearms, small arms and light weapons and their ammunition<br>entitled 'Securing Arms, Protecting Citizens' as well as the Commission's Communication entitled 'Artificial Intelligence for<br>Europe', the Union shall support the implementation of a project, 'Promoting Responsible Innovation in Artificial<br>Intelligence for Peace and Security'.<br>2. The project activities to be supported by the Union shall have the specific objective to support greater engagement of<br>the civilian artificial intelligence (AI) community in mitigating the risks that the diversion and misuse of civilian AI research<br>and innovation by irresponsible actors may pose to international peace and security, by:<br>— generating greater understanding of how decisions in the development and diffusion of AI research and innovation can<br>impact the risks of diversion and misuse, and in turn generate risk or opportunities for peace and security;<br>— promoting responsible innovation processes, methods and tools which can help ensure the peaceful application of<br>civilian innovations and the responsible dissemination of AI knowledge. To that end, the project will support capacity-<br>building, research and engagement activities that: enhance the capacity within the global civilian AI community to<br>include and address the peace and security risks presented by the diversion and misuse of civilian AI by irresponsible<br>actors through responsible innovation processes; and strengthen the connection between risk mitigation efforts in

					responsible AI in the civilian sphere with those already ongoing in the disarmament, arms control and non-proliferation community at an intergovernmental level.<br>3.The project and the activities referred to in paragraphs 1 and 2 are not intended to establish any new standards,<br>principles or regulation, or otherwise enter into areas within the competence of Member States. Instead, the intention is to<br>develop civilian responsible innovation efforts to include peace and security risks presented by the diversion and misuse of<br>civilian AI by irresponsible actors, and provide education on existing relevant intergovernmental efforts
Europe	2022		COUNCIL DECISION (CFSP) 2022/2320<br>of 25 November 2022<br>on Union support for the implementation of a project 'Unlocking Innovation: Enabling Technologies<br>and International Security'	COUNCIL DECISION (CFSP) 2022/2320<br>of 25 November 2022<br>on Union support for the implementation of a project 'Unlocking Innovation: Enabling Technologies<br>and International Security'	
Europe	2021		Setting up a special committee on artificial intelligence in a digital age, and defining its responsibilities, numerical strength and term of office European Parliament decision of 18 June 2020 on setting up a special committee on artificial intelligence in<br>a digital age, and defining its responsibilities, numerical strength and term of office (2020/2684(RSO)	Setting up a special committee on artificial intelligence in a digital age, and defining its responsibilities, numerical strength and term of office European Parliament decision of 18 June 2020 on setting up a special committee on artificial intelligence in<br>a digital age, and defining its responsibilities, numerical strength and term of office (2020/2684(RSO)	1. Decides to set up a special committee on artificial intelligence in a digital age, vested with the following strictly defined responsibilities:<br>(a) to analyse the future impact of artificial intelligence in the digital age on the EU economy, in particular on skills, employment, fintech, education, health, transport, tourism, agriculture, environment, defence, industry, energy and e-government;<br>(b) to further investigate the challenge of deploying artificial intelligence and its contribution to business value and economic growth;<br>(c) to analyse the approach of third countries and their contribution to complementing EU actions;<br>(d) to submit to Parliament's responsible standing committees an evaluation defining common EU objectives in the medium- and long-term and include the major steps needed to reach them, using as a starting point the following Commission communications published on 19 February 2020:<br>– Shaping Europe's digital future (COM(2020)0067),<br>– A European Strategy for Data (COM(2020)0066),<br>– White Paper on Artificial Intelligence - a European approach to excellence and trust (COM(2020)0065),<br>– Report on the safety and liability implications of Artificial Intelligence, the Internet of Things and robotics (COM(2020)0064),

Europe	2021		REGULATION (EU) 2021/692 OF THE EUROPEAN PARLIAMENT AND OF THE COUNCIL of 28 April 2021 establishing the Citizens, Equality, Rights and Values Programme and repealing Regulation (EU) No 1381/2013 of the European Parliament and of the Council and Council Regulation (EU) No 390/2014	REGULATION (EU) 2021/692 OF THE EUROPEAN PARLIAMENT AND OF THE COUNCIL of 28 April 2021 establishing the Citizens, Equality, Rights and Values Programme and repealing Regulation (EU) No 1381/2013 of the European Parliament and of the Council and Council Regulation (EU) No 390/2014	Article 4 - equality, rights and gender equality strand<br>within the general objective set out in article 2(1) and within the specific objectives set out in point (b) of Article 2(2), the programme shall focus on<br>(3) protecting and promoting Union citizenship rights and the right to the protection of personal data.
Europe	2022		COUNCIL DECISION (EU) 2022/2349 of 21 November 2022<br>authorising the opening of negotiations on behalf of the European Union for a Council of Europe<br>convention on artificial intelligence, human rights, democracy and the rule of law	COUNCIL DECISION (EU) 2022/2349 of 21 November 2022<br>authorising the opening of negotiations on behalf of the European Union for a Council of Europe<br>convention on artificial intelligence, human rights, democracy and the rule of law	Article 1<br>1. The Commission is hereby authorised to open negotiations, on behalf of the Union, as regards matters falling within<br>the exclusive competence of the Union, for a Council of Europe convention on artificial intelligence, human rights,<br>democracy and the rule of law.<br>2.The negotiations shall be conducted on the basis of the negotiating directives of the Council set out in the addendum<br>to this Decision, which may be revised and further developed as appropriate depending on the evolution of the<br>negotiations.<br><br>Article 2<br>The negotiations referred to in Article 1 shall be conducted in consultation with the Working Party on Telecommunications<br>and Information Society, which is hereby designated as the special committee within the meaning of Article 218(4) TFEU.

Europe	2020		DECISION OF THE GOVERNING BOARD OF THE INNOVATIVE MEDICINES INITIATIVE 2 JOINT UNDERTAKING<br>of 11 August 2020 laying down internal rules concerning restrictions of certain rights of data subjects in relation to processing of personal data in the framework of the functioning of the IMI 2 JU	DECISION OF THE GOVERNING BOARD OF THE INNOVATIVE MEDICINES INITIATIVE 2 JOINT UNDERTAKING<br>of 11 August 2020 laying down internal rules concerning restrictions of certain rights of data subjects in relation to<br>processing of personal data in the framework of the functioning of the IMI 2 JU	Article 1 Subject matter and scope<br>1. This Decision lays down rules relating to the conditions under which the IMI 2 JU in the framework of its procedures<br>set out paragraph 2 may restrict the application of the rights enshrined in Articles 14 to 22, 35 and 36, as well as Article 4<br>thereof, following Article 25 of Regulation (EU) 2018/1725.<br>2. Within the framework of the administrative functioning of the IMI 2 JU, this Decision applies to the processing<br>operations on personal data by the Programme Office for the purposes of conducting administrative inquiries, disciplinary<br>proceedings, preliminary activities related to cases of potential irregularities reported to OLAF, processing whistleblowing<br>cases, (formal and informal) procedures of harassment, processing internal and external complaints, conducting internal<br>audits, investigations carried out by the Data Protection Officer in line with Article 45(2) of Regulation (EU) 2018/1725<br>and (IT) security investigations handled internally or with external involvement (e.g. CERT-EU).<br>3. The categories of data concerned are hard data ('objective' data such as identification data, contact data, professional<br>data, administrative details, data received from specific sources, electronic communications and traffic data) and soft data<br>('subjective' data related to the case such as reasoning, behavioural data, appraisals, performance and conduct data and data<br>related to or brought forward in connection with the subject matter of the procedure or activity).<br>4. Where the IMI 2 JU performs its duties with respect to data subject's rights under Regulation (EU) 2018/1725, it shall<br>consider whether any of the exemptions laid down in that Regulation apply.<br>5.Subject to the conditions set out in this Decision, the restrictions may apply to the following rights: provision of<br>information to data subjects, right of access, rectification, erasure, restriction of processing, communication of a personal<br>data breach to the data subject or confidentiality of communication.<br><br>Article 5 Restrictions to the rights of data subjects<br>1. In duly justified cases and under the conditions stipulated in this decision, the following rights may be restricted by<br>the controller in the context of the processing operations listed in paragraph 2 below where necessary and proportionate:<br>(a) the right to information;<br>(b) the right of access;<br>(c) the right of rectification, erasure and restriction of processing;<br>(d) the right to communication of a personal data breach to the data subject;

					(e) the right to confidentiality of electronic communications.<br>2. In accordance with Article 25(2)(a) of Regulation (EU) 2018/1725, in duly justified cases and under the conditions<br>stipulated in this decision, restrictions may be applied by the controller in the context of the following processing<br>operations:<br>(a) the performance of administrative inquiries and disciplinary proceedings;<br>(b) preliminary activities related to cases of potential irregularities reported to OLAF;<br>(c) whistleblowing procedures;<br>(d) formal and informal) procedures for cases of harassment (<br>(e) processing internal and external complaints;<br>(f) internal audits;<br>(g) the investigations carried out by the Data Protection Officer in line with Article 45(2) of Regulation (EU) 2018/1725;<br>(h) (IT) security investigations handled internally or with external involvement (e.g. CERT-EU);<br>(i) within the frame of the grant management or procurement procedure, after the closing date of the submission of the<br>calls for proposals or the application of tenders. The restriction shall continue to apply as long as the reasons justifying it remain applicable.
Europe	2017		REGULATION (EU) 2017/2394 OF THE EUROPEAN PARLIAMENT AND OF THE COUNCIL of 12 December 2017 on cooperation between national authorities responsible for the enforcement of consumer protection laws and repealing	REGULATION (EU) 2017/2394 OF THE EUROPEAN PARLIAMENT AND OF THE COUNCIL of 12 December 2017 on cooperation between national authorities responsible for the enforcement of consumer protection laws and repealing	Article 1<br>Subject matter<br>This Regulation lays down the conditions under which competent authorities, having been designated by their Member States as responsible for the enforcement of Union laws that protect consumers' interests, cooperate and coordinate actions with each other and with the Commission, in order to enforce compliance with those laws and to ensure the smooth functioning of the internal market, and in order to enhance the protection of consumers' economic interests.

			Regulation (EC) No 2006/2004	Regulation (EC) No 2006/2004	
Europe	2017		REGULATION (EU) 2017/745 OF THE EUROPEAN PARLIAMENT AND OF THE COUNCIL of 5 April 2017 on medical devices, amending Directive 2001/83/EC, Regulation (EC) No 178/2002 and Regulation (EC) No 1223/2009 and repealing Council Directives 90/385/EEC and 93/42/EEC	REGULATION (EU) 2017/745 OF THE EUROPEAN PARLIAMENT AND OF THE COUNCIL of 5 April 2017 on medical devices, amending Directive 2001/83/EC, Regulation (EC) No 178/2002 and Regulation (EC) No 1223/2009 and repealing Council Directives 90/385/EEC and 93/42/EEC	CHAPTER II<br>MAKING AVAILABLE ON THE MARKET AND PUTTING INTO SERVICE OF DEVICES, OBLIGATIONS OF ECONOMIC OPERATORS, REPROCESSING, CE MARKING, FREE MOVEMENT<br>Article 5 Placing on the market and putting into service<br>1. A device may be placed on the market or put into service only if it complies with this Regulation when duly supplied and properly installed, maintained and used in accordance with its intended purpose.<br>2. A device shall meet the general safety and performance requirements set out in Annex I which apply to it, taking into account its intended purpose.<br><br>Article 109 Confidentiality<br>1. Unless otherwise provided for in this Regulation and without prejudice to existing national provisions and practices in the Member States on confidentiality, all parties involved in the application of this Regulation shall respect the confidentiality of information and data obtained in carrying out their tasks in order to protect the following:<br>(a) personal data, in accordance with Article 110;<br><br>Article 110 Data protection<br>1. Member States shall apply Directive 95/46/EC to the processing of personal data carried out in the Member States pursuant to this Regulation.<br>2. Regulation (EC) No 45/2001 shall apply to the processing of personal data carried out by the Commission pursuant to this Regulation.
Europe	2022		DIRECTIVE (EU) 2022/2555 OF THE EUROPEAN PARLIAMENT AND OF THE COUNCIL of 14 December 2022 on measures for a high common level of cybersecurity across the Union, amending Regulation (EU) No 910/2014	DIRECTIVE (EU) 2022/2555 OF THE EUROPEAN PARLIAMENT AND OF THE COUNCIL of 14 December 2022 on measures for a high common level of cybersecurity across the Union, amending Regulation (EU) No 910/2014	Article 1 Subject matter<br>This Directive lays down measures that aim to achieve a high common level of cybersecurity across the Union, with a<br>view to improving the functioning of the internal market

			and Directive (EU) 2018/1972, and repealing Directive (EU) 2016/1148 (NIS 2 Directive)	and Directive (EU) 2018/1972, and repealing Directive (EU) 2016/1148 (NIS 2 Directive)	
Europe	2019		Regulation (EU) 2019/1020 of the European Parliament and of the Council of 20 June 2019 on market surveillance and compliance of products and amending Directive 2004/42/EC and Regulations (EC) No 765/2008 and (EU) No 305/2011 (Text with EEA relevance.)	Regulation (EU) 2019/1020 of the European Parliament and of the Council of 20 June 2019 on market surveillance and compliance of products and amending Directive 2004/42/EC and Regulations (EC) No 765/2008 and (EU) No 305/2011 (Text with EEA relevance.)	CHAPTER I GENERAL PROVISIONS<br><br>Article 1 Subject matter<br><br>1. The objective of this Regulation is to improve the functioning of the internal market by strengthening the market surveillance of products covered by the Union harmonisation legislation referred to in Article 2, with a view to ensuring that only compliant products that fulfil requirements providing a high level of protection of public interests, such as health and safety in general, health and safety in the workplace, the protection of consumers, the protection of the environment and public security and any other public interests protected by that legislation, are made available on the Union market
Europe	2015		REGULATION (EU) 2015/1017 OF THE EUROPEAN PARLIAMENT AND OF THE COUNCIL of 25 June 2015<br>on the European Fund for Strategic Investments, the European Investment Advisory Hub and the European Investment Project Portal and amending Regulations (EU) No 1291/2013 and (EU) No 1316/2013 — the European Fund for Strategic Investments	REGULATION (EU) 2015/1017 OF THE EUROPEAN PARLIAMENT AND OF THE COUNCIL of 25 June 2015<br>on the European Fund for Strategic Investments, the European Investment Advisory Hub and the European Investment Project Portal and amending Regulations (EU) No 1291/2013 and (EU) No 1316/2013 — the European Fund for Strategic Investments	Article 9 - requirements for the use of the EU guarantee<br>2. a) research, development and innovation, in particular through:<br>(i) projects that are in line with Horizon 2020;<br>(ii) research infrastructures;<br>(iii) demonstration projects and programmes as well as deployment<br>of related infrastructures, technologies and processes;<br>(iv) support to academia including collaboration with industry;<br>(v) knowledge and technology transfer;
Europe	2021		Decision (EU) 2021/820 of the European Parliament and of the Council of 20 May 2021 on the Strategic Innovation Agenda of the European Institute of Innovation and Technology (EIT) 2021-2027: Boosting the Innovation Talent and Capacity of Europe and	Decision (EU) 2021/820 of the European Parliament and of the Council of 20 May 2021 on the Strategic Innovation Agenda of the European Institute of Innovation and Technology (EIT) 2021-2027: Boosting the Innovation Talent and Capacity of	"The role of Europe as a global actor includes the need to enhance the dissemination of European cultural content. Europe needs to remain competitive in the global digital race for the creation of new technologies (such as Artificial Intelligence, the Internet of Things and blockchain) for which CCSI are important generators of content, products and services. Moreover, on a global scale, CCSI (such as design and architecture) contribute actively to the sustainable development and drive green innovation, while cultural content (literature, film and the arts) can, in addition to its intrinsic value, raise awareness of ecological problems and inform public opinion."

			repealing Decision No 1312/2013/EU (Text with EEA relevance)	Europe and repealing Decision No 1312/2013/EU (Text with EEA relevance)	"Digital Europe Programme, established by Regulation (EU) 2021/694 of the European Parliament and of the Council (23)<br>—The KICs (knowledge and innovation communities), in particular the CLCs, shall collaborate with the European Digital Innovation Hubs in accordance with Regulation (EU) 2021/694 to support the digital transformation of the industry and public sector organisations.<br><br>— Feasibilities shall be explored to use infrastructures and capacities developed under the Digital Europe Programme (such as data resources and libraries of artificial intelligence algorithms and high performance computing competence centres in Member States) by the KICs in education and training, as well as for testing and demonstration purposes in innovation projects."
Europe	2024			Artificial Intelligence Act	Article 1 Subject matter<br>1. The purpose of this Regulation is to improve the functioning of the internal market and promoting the uptake of human centric and trustworthy artificial intelligence, while ensuring a high level of protection of health, safety, fundamental rights enshrined in the Charter, including democracy, rule of law and environmental protection against harmful effects of artificial intelligence systems in the Union and supporting innovation<br>2. This Regulation lays down:<br>(a) harmonised rules for the placing on the market, the putting into service and the use of artificial intelligence systems (‘AI systems’) in the Union;<br>(b) prohibitions of certain artificial intelligence practices;<br>(c) specific requirements for high-risk AI systems and obligations for operators of such systems;<br>(d) harmonised transparency rules for certain AI systems;<br>(da) harmonised rules for the placing on the market of general-purpose AI models;<br>(e) rules on market monitoring, market surveillance governance and enforcement;<br>(ea) measures to support innovation, with a particular focus on SMEs, including start-ups

Europe	2024			Artificial Intelligence Act	Article 2 Scope<br>1. This Regulation applies to:<br>(a) providers placing on the market or putting into service AI systems or placing on the market general-purpose AI models in the Union, irrespective of whether those providers are established or who are located within the Union or in a third country;<br>(b) deployers of AI systems that have their place of establishment or who are located within the Union;<br>(c) providers and deployers of AI systems that have their place of establishment or who are located in a third country, where the output produced by the system is used in the Union;<br>(ca) importers and distributors of AI systems;<br>(cb) product manufacturers placing on the market or putting into service an AI system together with their product and under their own name or trademark;<br>(cc) authorised representatives of providers, which are not established in the Union.<br>(cc) affected persons that are located in the Union.<br>2. For AI systems classified as high-risk AI systems in accordance with Articles 6(1) and 6(2) related to products covered by Union harmonisation legislation listed in Annex II, section B only Article 84 of this Regulation shall apply. Article 53 shall apply only insofar as the requirements for high-risk AI systems under this Regulation have been integrated under that Union harmonisation legislation.<br>3. This Regulation shall not apply to areas outside the scope of EU law and in any event shall not affect the competences of the Member States concerning national security, regardless of the type of entity entrusted by the Member States to carry out the tasks in relation to those competences.<br>This Regulation shall not apply to AI systems if and insofar placed on the market, put into service, or used with or without modification of such systems exclusively for military, defence or national security purposes, regardless of the type of entity carrying out those activities.<br>This Regulation shall not apply to AI systems which are not placed on the market or put into service in the Union, where the output is used in the Union exclusively for military, defence or national security purposes, regardless of the type of entity carrying out those activities.<br>4. This Regulation shall not apply to public authorities in a third country nor to international organisations falling within the scope of this Regulation pursuant to paragraph 1, where those authorities or organisations use AI systems in the framework of international cooperation or agreements for law enforcement and judicial cooperation with the Union or with one or more Member States, under the condition that this third country or international organisations provide adequate safeguards with respect to the protection of fundamental rights and freedoms of individuals.<br>5. This Regulation shall not affect the application of the provisions on the liability of intermediary service providers set out in Chapter II, Section 4 of Directive 2000/31/EC of the European Parliament and of the Council29 [as to be replaced by the corresponding provisions of the Digital Services Act].<br>5a. This Regulation shall not apply to AI systems and models, including their output, specifically developed and put into service for the sole purpose of scientific research and development.<br>5a. Union law on the protection of personal data, privacy and the confidentiality of communications applies to personal data processed in connection with the rights and obligations laid down in this Regulation. This Regulation shall not affect Regulations (EU) 2016/679 and (EU) 2018/1725 and Directives 2002/58/EC and (EU) 2016/680, without prejudice to arrangements provided for in Article 10(5) and Article 54 of this Regulation.

					5b. This Regulation shall not apply to any research, testing and development activity regarding AI systems or models prior to being placed on the market or put into service; those activities shall be conducted respecting applicable Union law. The testing in real world conditions shall not be covered by this exemption.<br>5b. This Regulation is without prejudice to the rules laid down by other Union legal acts related to consumer protection and product safety.<br>5c. This Regulation shall not apply to obligations of deployers who are natural persons using AI systems in the course of a purely personal non-professional activity.<br>5e. This Regulation shall not preclude Member States or the Union from maintaining or introducing laws, regulations or administrative provisions which are more favourable to workers in terms of protecting their rights in respect of the use of AI systems by employers, or to encourage or allow the application of collective agreements which are more favourable to workers.<br>5g. The obligations laid down in this Regulation shall not apply to AI systems released under free and open source licences unless they are placed on the market or put into service as high-risk AI systems or an AI system that falls under Title II and IV.
Europe	2024			Artificial Intelligence Act	Article 3 Definitions<br>For the purpose of this Regulation, the following definitions apply:<br>(1)‘artificial intelligence system’ (AI system) means software that is developed with one or more of the techniques and approaches listed in Annex I and can, for a given set of human-defined objectives, generate outputs such as content, predictions, recommendations, or decisions influencing the environments they interact with; Article 3 definitions<br>(1) ‘AI system‘ is a machine-based system designed to operate with varying levels of autonomy and that may exhibit adaptiveness after deployment and that, for explicit or implicit objectives, infers, from the input it receives, how to generate outputs such as predictions, content, recommendations, or decisions that can influence physical or<br>virtual environments;<br>(bg) 'AI regulatory sandbox’ means a concrete and controlled framework set up by a competent authority which offers providers or prospective providers of AI systems the possibility to develop, train, validate and

					test, where appropriate in real world conditions, an innovative AI system, pursuant to a sandbox plan for a limited time under regulatory supervision;<br>(bi) ‘testing in real world conditions’ means the temporary testing of an AI system for its intended purpose in real world conditions outside of a laboratory or otherwise simulated environment with a view to gathering reliable and robust data and to<br>assessing and verifying the conformity of the AI system with the requirements of this Regulation; testing in real world conditions shall not be considered as placing the AI system on the market or putting it into service within the meaning of this Regulation,<br>provided that all conditions under Article 53 or Article 54a are fulfilled

Europe	2024			Artificial Intelligence Act	Article 5 Prohibited Artificial Intelligence Practices<br>1. The following artificial intelligence practices shall be prohibited:<br>(a) the placing on the market, putting into service or use of an AI system that deploys subliminal techniques beyond a person's consciousness or purposefully manipulative or deceptive techniques, with the objective to or the effect of materially distorting a person's or a group of persons' behaviour by appreciably impairing the person's ability to make an informed decision, thereby causing the person to take a decision that that person would not have otherwise taken in a manner that causes or is likely to cause that person, another person or group of persons significant harm;<br>(b) the placing on the market, putting into service or use of an AI system that exploits any of the vulnerabilities of a person or a specific group of persons due to their age, disability or a specific social or economic situation, with the objective to or the effect of materially distorting the behaviour of that person or a person pertaining to that<br>group in a manner that causes or is reasonably likely to cause that person or another person significant harm;<br>(ba) the placing on the market or putting into service for this specific purpose, or use of biometric categorisation systems that categorise individually natural persons based on their biometric data to deduce or infer their race, political opinions, trade union membership, religious or philosophical beliefs, sex life or sexual orientation.<br>This prohibition does not cover any labelling or filtering of lawfully acquired biometric datasets, such as images, based on biometric data or categorizing of biometric data in the area of law enforcement;<br>(c) the placing on the market, putting into service or use of AI systems for the evaluation or classification of natural persons or groups thereof over a certain period of time based on their social behaviour or known, inferred or predicted personal or personality characteristics, with the social score leading to either or both of the following:<br>(i) detrimental or unfavourable treatment of certain natural persons or whole groups thereof in social contexts that are unrelated to the contexts in which the data was originally generated or collected;<br>(ii) detrimental or unfavourable treatment of certain natural persons or groups thereof that is unjustified or disproportionate to their social behaviour or its gravity;<br>(d) the use of 'real-time' remote biometric identification systems in publicly accessible spaces for the purpose of law enforcement unless and in as far as such use is strictly necessary for one of the following objectives:<br>(i) the targeted search for specific victims of abduction, trafficking in human beings and sexual exploitation of human beings as well as search for missing persons;<br>(ii) the prevention of a specific, substantial and imminent threat to the life or physical safety of natural persons or a genuine and present or genuine and foreseeable threat of a terrorist attack;<br>(iii) the localisation or identification of a person suspected of having committed a criminal offence, for the purposes of conducting a criminal investigation, prosecution or executing a criminal penalty for offences, referred to in Annex<br>IIa and punishable in the Member State concerned by a custodial sentence or a detention order for a maximum period of at least four years. This paragraph is without prejudice to the provisions in Article 9 of the GDPR for the processing<br>of biometric data for purposes other than law enforcement.

					(da) the placing on the market, putting into service for this specific purpose, or use of an AI system for making risk assessments of natural persons in order to assess or predict the risk of a natural person to commit a criminal offence, based solely on the profiling of a natural person or on assessing their personality traits and characteristics. This prohibition shall not apply to AI systems used to support the human assessment of the involvement of a person in a criminal activity, which is already based on objective and verifiable facts directly linked to a criminal activity;<br>(db) the placing on the market, putting into service for this specific purpose, or use of AI systems that create or expand facial recognition databases through the untargeted scraping of facial images from the internet or CCTV footage;<br>(dc) the placing on the market, putting into service for this specific purpose, or use of AI systems to infer emotions of a natural person in the areas of workplace and education institutions except in cases where the use of the AI system is intended to be put in place or into the market for medical or safety reasons.<br><br>2. The use of ‘real-time’ remote biometric identification systems in publicly accessible spaces for the purpose of law enforcement for any of the objectives referred to in paragraph 1 point (d) shall only be deployed for the purposes under paragraph 1, point (d) to confirm the specifically targeted individual’s identity and it shall take into account the following<br>elements:<br>(a) the nature of the situation giving rise to the possible use, in particular the seriousness, probability and scale of the harm caused in the absence of the use of the system;<br>(b) the consequences of the use of the system for the rights and freedoms of all persons concerned, in particular the seriousness, probability and scale of those consequences.<br><br>6. The Commission shall publish annual reports on the use of ‘real-time’ remote biometric identification systems in publicly accessible spaces for law enforcement purposes based on aggregated data in Member States based on the annual reports referred to in paragraph 5, which shall not include sensitive operational data of the related law enforcement activities.
Europe	2024			Artificial Intelligence Act	Article 6 Classification rules for high-risk AI systems<br>1. Irrespective of whether an AI system is placed on the market or put into service independently from the products referred to in points (a) and (b), that AI system shall be considered high-risk where both of the following conditions are fulfilled:<br>(a) the AI system is intended to be used as a safety component of a product, or the AI system is itself a product, covered by the Union harmonisation legislation listed in Annex II;<br>(b) the product whose safety component pursuant to point (a) is the AI system, or the AI system itself as a product, is required to undergo a third-party conformity assessment, with a view to the placing on the

					market or putting into service of that product pursuant to the Union harmonisation legislation listed in Annex II.<br>2. In addition to the high-risk AI systems referred to in paragraph 1, AI systems referred to in Annex III shall also be considered high-risk
Europe	2024			Artificial Intelligence Act	Article 7 Amendments to Annex III<br>1. The Commission is empowered to adopt delegated acts in accordance with Article 73 to amend Annex III by adding or modifying use cases of high-risk AI systems where both of the following conditions are fulfilled:<br>(a) the AI systems are intended to be used in any of the areas listed in points 1 to 8 of Annex III;<br>(b) the AI systems pose a risk of harm to health and safety, or an adverse impact on fundamental rights, and that risk is equivalent to or greater than the risk of harm or of adverse impact posed by the high-risk AI systems already referred to in Annex III.
Europe	2024			Artificial Intelligence Act	Article 8 Compliance with requirements<br>1. High-risk AI systems shall comply with the requirements established in this Chapter, taking into account its intended purpose as well as the generally acknowledged state of the art on AI and AI related technologies. The risk management system referred to in Article 9 shall be taken into account when ensuring compliance with those requirements.
Europe	2024			Artificial Intelligence Act	Article 9 Risk management system<br>1. A risk management system shall be established, implemented, documented and maintained in relation to high-risk AI systems.<br>2. The risk management system shall be understood as a continuous iterative process planned and run throughout the entire lifecycle of a high-risk AI system, requiring regular systematic review and updating. It shall comprise the following steps:<br>(a) identification and analysis of the known and the reasonably foreseeable risks that the high-risk AI system can pose to the health, safety or fundamental rights when the high-risk AI system is used in accordance with its intended purpose;<br>(b) estimation and evaluation of the risks that may emerge when the high-risk AI system is used in accordance with its intended purpose and under conditions of reasonably foreseeable misuse;<br>(c) evaluation of other possibly arising risks based on the analysis of data gathered from the post-market monitoring system referred to in Article 61;<br>(d) adoption of appropriate and targeted risk management measures designed to address the risks identified pursuant to point a of this paragraph in accordance with the provisions of the following paragraphs.

Europe	2024			Artificial Intelligence Act	Article 10 Data and data governance<br>1. High-risk AI systems which make use of techniques involving the training of models with data shall be developed on the basis of training, validation and testing data sets that meet the quality criteria referred to in paragraphs 2 to 5 whenever such datasets are used.<br>2. Training, validation and testing data sets shall be subject to appropriate data governance and management practices appropriate for the intended purpose of the AI system. Those practices shall concern in particular:<br>(a) the relevant design choices;<br>(aa) data collection processes and origin of data, and in the case of personal data, the original purpose of data collection;<br>(c) relevant data preparation processing operations, such as annotation, labelling, cleaning, updating, enrichment and aggregation;<br>(d) the formulation of assumptions, notably with respect to the information that the data are supposed to measure and represent;<br>(e) an assessment of the availability, quantity and suitability of the data sets that are needed;<br>(f) examination in view of possible biases that are likely to affect the health and safety of persons, negatively impact fundamental rights or lead to discrimination prohibited under Union law, especially where data outputs influence inputs for future operations;<br>(fa) appropriate measures to detect, prevent and mitigate possible biases identified according to point (f);<br>(g) the identification of relevant data gaps or shortcomings that prevent compliance with this Regulation, and how those gaps and shortcomings can be addressed.<br>3. Training, validation and testing datasets shall be relevant, sufficiently representative, and to the best extent possible, free of errors and complete in view of the intended purpose. They shall have the appropriate statistical properties, including, where applicable, as regards the persons or groups of persons in relation to whom the high-risk AI system is intended to be used. These characteristics of the data sets may be met at the level of individual data sets or a combination thereof.<br>4. Datasets shall take into account, to the extent required by the intended purpose, the characteristics or elements that are particular to the specific geographical, contextual, behavioural or functional setting within which the high-risk AI system is intended to be used.<br>5. To the extent that it is strictly necessary for the purposes of ensuring bias detection and correction in relation to the high-risk AI systems in accordance with the second paragraph, point f and fa, the providers of such systems may exceptionally process special categories of personal data referred to in Article 9(1) of Regulation (EU) 2016/679, Article 10 of Directive (EU) 2016/680 and Article 10(1) of Regulation (EU) 2018/1725, subject to appropriate safeguards for the fundamental rights and freedoms of natural persons. In addition to provisions set out in the Regulation (EU) 2016/679, Directive (EU) 2016/680 and Regulation (EU) 2018/1725, all the following conditions shall apply in order for such processing to occur:<br>(a) the bias detection and correction cannot be effectively fulfilled by processing other data, including synthetic or anonymised data;<br>(b) the special categories of personal data processed for the purpose of this paragraph are subject to technical limitations on the re-use of the personal data and state of the art security and privacy-preserving measures, including pseudonymisation;

					(c) the special categories of personal data processed for the purpose of this paragraph are subject to measures to ensure that the personal data processed are secured, protected subject to suitable safeguards, including strict controls and documentation of the access, to avoid misuse and ensure only authorised persons have access to those personal data with appropriate confidentiality obligations;<br>(d) the special categories of personal data processed for the purpose of this paragraph are not to be transmitted, transferred or otherwise accessed by other parties;<br>(e) the special categories of personal data processed for the purpose of this paragraph are deleted once the bias has been corrected or the personal data has reached the end of its retention period, whatever comes first;<br>(f) the records of processing activities pursuant to Regulation (EU) 2016/679, Directive (EU) 2016/680 and Regulation (EU) 2018/1725 includes justification why the processing of special categories of personal data was strictly necessary to detect and<br>correct biases and this objective could not be achieved by processing other data.<br>6. For the development of high-risk AI systems not using techniques involving the training of models, paragraphs 2 to 5 shall apply only to the testing data sets.
Europe	2024			Artificial Intelligence Act	Article 11 Technical documentation<br>1. The technical documentation of a high-risk AI system shall be drawn up before that system is placed on the market or put into service and shall be kept up-to date.<br>The technical documentation shall be drawn up in such a way to demonstrate that the high-risk AI system complies with the requirements set out in this Chapter and provide national competent authorities and notified bodies with the necessary information in a clear and comprehensive form to assess the compliance of the AI system with those requirements<br><br>Article 12 Record-keeping<br>1. High-risk AI systems shall technically allow for the automatic recording of events (‘logs’) over the duration of the lifetime of the system.<br>2. In order to ensure a level of traceability of the AI system’s functioning that is appropriate to the intended purpose of the system, logging capabilities shall enable the recording of events relevant for:<br>(i) identification of situations that may result in the AI system presenting a risk within the meaning of Article 65(1) or in a substantial modification;

					(ii) facilitation of the post-market monitoring referred to in Article 61; and<br>(iii) monitoring of the operation of high-risk AI systems referred to in Article 29(4).<br><br>Article 13 Transparency and provision of information to deployers<br>1. High-risk AI systems shall be designed and developed in such a way to ensure that their operation is sufficiently transparent to enable deployers to interpret the system's output and use it appropriately. An appropriate type and degree of transparency shall be ensured with a view to achieving compliance with the relevant obligations of the provider and deployer set out in Chapter 3 of this Title.<br><br>Article 14 Human oversight<br>1. High-risk AI systems shall be designed and developed in such a way, including with appropriate human-machine interface tools, that they can be effectively overseen by natural persons during the period in which the AI system is in use.
Europe	2024			Artificial Intelligence Act	Article 40 Harmonised standards and standardisation deliverables<br>1. High-risk AI systems or general purpose AI models which are in conformity with harmonised standards or parts thereof the references of which have been published in the Official Journal of the European Union in accordance with Regulation (EU) 1025/2012 shall be presumed to be in conformity with the requirements set out in Chapter 2 of this Title or, as applicable, with the requirements set out in [Chapter on GPAI], to the extent those standards cover those requirements.
Europe	2024			Artificial Intelligence Act	Article 52 Transparency obligations for providers and users of certain AI systems and GPAI models<br>1. Providers shall ensure that AI systems intended to directly interact with natural persons are designed and developed in such a way that the concerned natural persons are informed that they are interacting with an AI system, unless this is obvious from the point of view of a natural person who is reasonably well-informed, observant and circumspect, taking into account the circumstances and the context of use. This obligation shall not apply to AI systems authorised by law to detect, prevent, investigate and prosecute criminal offences, subject to appropriate safeguards for the rights and freedoms of third parties unless those systems are available for the public to report a criminal offence
Europe	2024			Artificial Intelligence Act	Article 53 AI regulatory sandboxes<br>1. Member States shall ensure that their competent authorities establish at least one AI regulatory sandbox at national level, which shall be operational 24 months after entry into force. This sandbox may also be established jointly with one or several other Member States' competent authorities. The Commission may provide technical support, advice and<br>tools for the establishment and operation of AI regulatory sandboxes. The obligation established in previous paragraph can also be fulfilled by participation in an existing sandbox insofar as this participation provides equivalent level of national coverage for the participating Member States.<br>1g. The establishment of AI regulatory sandboxes shall aim to contribute to the following objectives:<br>(a) improve legal certainty to achieve regulatory compliance with this Regulation or, where relevant, other applicable Union and Member States legislation;

					(b) support the sharing of best practices through cooperation with the authorities involved in the AI regulatory sandbox;<br>(c) foster innovation and competitiveness and facilitate the development of an AI ecosystem;<br>(d) contribute to evidence-based regulatory learning;<br>(e) facilitate and accelerate access to the Union market for AI systems, in particular when provided by small and medium-sized enterprises (SMEs), including start-ups
Europe	2024			Artificial Intelligence Act	Article 54a Testing of high-risk AI systems in real world conditions outside AI regulatory sandboxes<br>1. Testing of AI systems in real world conditions outside AI regulatory sandboxes may be conducted by providers or prospective providers of high-risk AI systems listed in Annex III, in accordance with the provisions of this Article and the real-world testing plan referred to in this Article, without prejudice to the prohibitions under Article 5. The detailed elements of the real world testing plan shall be specified in implementing acts adopted by the Commission in accordance with the examination procedure referred to in Article 74(2). This provision shall be without prejudice to Union or national law for the testing in real world conditions of high-risk AI systems related to products covered by legislation listed in Annex II.
Europe	2024			Artificial Intelligence Act	Article 56 Establishment and structure of the European Artificial intelligence board<br>1. A ‘European Artificial Intelligence Board’ (the ‘Board’) is established.<br>2. The Board shall be composed of one representative per Member State. The European Data Protection Supervisor shall participate as observer. The AI Office shall also attend the Board’s meetings without taking part in the votes. Other national and Union authorities, bodies or experts may be invited to the meetings by the Board on a case by case basis, where the issues discussed are of relevance for them.<br>2a. Each representative shall be designated by their Member State for a period of 3 years, renewable once.<br>2b. Member States shall ensure that their representatives in the Board:<br>(a) have the relevant competences and powers in their Member State so as to contribute actively to the achievement of the Board’s tasks referred to in Article 58;<br>(b) are designated as a single contact point vis-à-vis the Board and, where appropriate, taking into account Member States’ needs, as a single contact point for stakeholders;<br>(c) are empowered to facilitate consistency and coordination between national competent authorities in their Member State as regards the implementation of this Regulation, including through the collection of relevant data and information for the purpose of fulfilling their tasks on the Board.<br><br>Article 58b Scientific panel of independent experts<br>1. The Commission shall, by means of an implementing act, make provisions on the establishment of a scientific panel of independent experts (the ‘scientific panel’) intended to support the enforcement activities under this Regulation. Those implementing acts shall be adopted in accordance with the examination procedure referred to in Article 74(2).<br>Article 59 Designation of national competent authorities and single point of contact<br>2. Each Member State shall establish or designate at least one notifying authority and at least one market surveillance authority for the purpose of this Regulation as national competent authorities. These national

					competent authorities shall exercise their powers independently, impartially and without bias so as to safeguard the principles of objectivity of their<br>activities and tasks and to ensure the application and implementation of this Regulation.<br>The members of these authorities shall refrain from any action incompatible with their duties. Provided that those principles are respected, such activities and tasks may be performed by one or several designated authorities, in accordance with the organisational needs of the Member State.<br><br>Article 60 EU database for high-risk AI systems listed in Annex III<br>1. The Commission shall, in collaboration with the Member States, set up and maintain a EU database containing information referred to in paragraphs 2 and 2a concerning high-risk AI systems referred to in Article 6(2) which are registered in accordance with Articles 51 and<br>54a. When setting the functional specifications of such database, the Commission shall consult the relevant experts, and when updating the functional specifications of such database, the Commission shall consult the AI Board
Europe	2024			Artificial Intelligence Act	Article 61 Post-market monitoring by providers and post-market monitoring plan for high-risk AI systems 1. Providers shall establish and document a post-market monitoring system in a manner that is proportionate to the nature of the artificial intelligence technologies and the risks of the high-risk AI system.
Europe	2024			REGULATION OF THE EUROPEAN PARLIAMENT AND OF THE<br>COUNCIL laying down measures for a high level of public sector interoperability across the Union (Interoperable Europe Act)	Art 1(1): This Regulation lays down measures to promote the cross-border interoperability of network and information systems which are used to provide or manage public services in the Union by establishing common rules and a framework for coordination on public sector interoperability.<br><br>Art 1(2): This Regulation applies to public sector bodies of Member States and institutions, bodies and agencies of the Union that provide or manage network or information systems that enable public services to be delivered or managed electronically.
Europe	2024			REGULATION OF THE EUROPEAN PARLIAMENT AND OF THE<br>COUNCIL laying down measures for a high level of public sector	Art 2(3): interoperability solution’ means a technical specification, including a standard, or another solution, including conceptual frameworks, guidelines and applications, describing legal, organisational, semantic or technical requirements to be fulfilled by a network and information system in order to enhance cross-border interoperability;

				interoperability across the Union (Interoperable Europe Act)	Art 2(4): public sector bodies means the State, regional or local authorities, bodies governed by public law or associations formed by one or more such authorities or one or more such bodies governed by public law.
Europe	2024			REGULATION OF THE EUROPEAN PARLIAMENT AND OF THE COUNCIL laying down measures for a high level of public sector interoperability across the Union (Interoperable Europe Act)	Art 3(1): Where a public sector body or an institution, an agency or body of the Union intends to set up a new or significantly modify an existing network and information system that enables public services to be delivered or managed electronically, it shall carry out an assessment of the impacts of the planned action on cross-border interoperability ('interoperability assessment') in the following cases:<br>(a) where the intended set-up or modification affects one or more network and information systems used for the provision of cross-border services across several sectors or administrations;<br>(b) where the intended set-up or modification will most likely result in procurements for network and information systems used for the provision of cross-border services above the threshold set out in Article 4of Directive 2014/24/EU;<br>(c) where the intended set-up or modification concerns a network and information system used for the provision of cross-border services and funded through Union programmes.<br><br>Art 3(2): The interoperability assessment shall be carried out before taking decisions on the legal, organisational, semantic or technical requirements for the new or modified network and information system in a binding manner. A single interoperability assessment may be carried out to address a set of requirements and several network and information systems.<br>The public sector body or the institution, body or agency of the Union concerned shall publish a report presenting the outcome of the interoperability assessment on its website.<br><br>Art 3(4): The interoperability assessment shall contain at least:<br>(a) a description of the intended operation and its impacts on the cross-border interoperability of one or several network and information systems concerned, including the estimated costs for the adaptation of the network and information systems concerned;<br>(b) a description of the level of alignment of the network and information systems concerned with the European Interoperability Framework, and with the Interoperable Europe solutions, after the operation and where it has improved compared to the level of alignment before the operation;<br>(c) a description of the Application Programming Interfaces that enable machine- to-machine interaction with the data considered relevant for cross-border exchange with other network and information systems.<br><br>Art 3(5): The public sector body, or institution, body or agency of the Union concerned shall consult recipients of the services affected or their representatives on the intended operation if it directly affects the recipients. This consultation is without prejudice to the protection of commercial or public interests or the security of such systems.<br><br>Art 3(6): The Interoperable Europe Board shall adopt guidelines on the content of the interoperability assessment by ... at the latest [one year after the entry into force of this Regulation], including practical check lists.

Europe	2024			REGULATION OF THE EUROPEAN PARLIAMENT AND OF THE COUNCIL laying down measures for a high level of public sector interoperability across the Union (Interoperable Europe Act)	Art 4(1): A public sector body or an institution, body or agency of the Union shall make available to any other such entity that requests it, interoperability solutions that support the public services that it delivers or manages electronically. The shared content shall include the technical documentation and, where applicable, the documented source code. This obligation to share shall not apply to any of the following interoperability solutions:<br>(a) that support processes which fall outside the scope of the public task of the public sector bodies or institutions, bodies, or agencies of the Union concerned as defined by law or by other binding rules, or, in the absence of such rules, as defined in accordance with common administrative practice in the Member State or Union administrations in question, provided that the scope of the public tasks is transparent and subject to review;<br>(b) for which third parties hold intellectual property rights and do not allow sharing;<br>EN 23 EN<br>(c) access to which is excluded or restricted on grounds of:<br>(i) sensitive critical infrastructure protection related information as defined in Article 2, point (d) of Council Directive 2008/114/EC41;<br>(ii) the protection of defence interests, or public security.<br><br>Art 4(2): To enable the reusing entity to manage the interoperability solution autonomously, the sharing entity shall specify the guarantees that will be provided to the reusing entity in terms of cooperation, support and maintenance. Before adopting the interoperability solution, the reusing entity shall provide to the sharing entity an assessment of the solution covering its ability to manage autonomously the cybersecurity and the evolution of the reused interoperability solution.
Europe	2024			REGULATION OF THE EUROPEAN PARLIAMENT AND OF THE COUNCIL laying down measures for a high level of public sector interoperability across the Union (Interoperable Europe Act)	Art 5(1): The Commission shall publish Interoperable Europe solutions and the European Interoperability Framework on the Interoperable Europe portal , by electronic means, in formats that are open, machine-readable, accessible, findable and re-usable, if applicable, together with their metadata.
Europe	2024			REGULATION OF THE EUROPEAN PARLIAMENT AND OF THE COUNCIL laying down measures for a high level of public sector interoperability across the Union (Interoperable Europe Act)	Art 6(1): The Interoperable Europe Board shall develop a European Interoperability Framework (EIF) and propose to the Commission to adopt it. The Commission may adopt the EIF. The Commission shall publish the EIF in the Official Journal of the European Union.<br><br>Art 6(2): The EIF shall provide a model and a set of recommendations on legal, organisational, semantic and technical interoperability, addressed to all entities falling within the scope of this Regulation for interacting with each other through their network and information systems. The EIF shall be taken into account in the interoperability assessment in accordance with Article 3(4), point (b) and Article 3(6).

					Art 6(3): The Commission, after consulting the Interoperable Europe Board, may adopt other interoperability frameworks (‘specialised interoperability frameworks’) targeting the needs of specific sectors or administrative levels. The specialised interoperability frameworks shall be based on the EIF. The Interoperable Europe Board shall assess the alignment of the specialised interoperability frameworks with the EIF. The Commission shall publish the specialised interoperability frameworks on the Interoperable Europe portal.<br>Art 6(4): Where a Member State develops a national interoperability framework and other relevant national policies, strategies or guidelines, it shall take into account the EIF.<br>Art 7(1): The Interoperable Europe Board shall recommend interoperability solutions for the cross- border interoperability of network and information systems which are used to provide or manage public services to be delivered or managed electronically in the Union. When an interoperability solution is recommended by the Interoperable Europe Board, it shall carry the label ‘Interoperable Europe solution’ and shall be published on the Interoperable Europe portal.
Europe	2024			REGULATION OF THE EUROPEAN PARLIAMENT AND OF THE COUNCIL laying down measures for a high level of public sector interoperability across the Union (Interoperable Europe Act)	Art 7(1): The Interoperable Europe Board shall recommend interoperability solutions for the cross- border interoperability of network and information systems which are used to provide or manage public services to be delivered or managed electronically in the Union. When an interoperability solution is recommended by the Interoperable Europe Board, it shall carry the label ‘Interoperable Europe solution’ and shall be published on the Interoperable Europe portal.
Europe	2024			REGULATION OF THE EUROPEAN PARLIAMENT AND OF THE COUNCIL laying down measures for a high level of public sector interoperability across the Union (Interoperable Europe Act)	Art 8(1): The Commission shall provide a portal (‘the Interoperable Europe portal’) as a single point of entry for information related to cross-border interoperability of network and information systems which are used to provide or manage public services to be delivered or managed electronically in the Union. The portal shall be electronically accessible and free of charge. The portal shall have at least the following functions:<br>(a) access to Interoperable Europe solutions;<br>(b) access to other interoperability solutions not bearing the label ‘Interoperable Europe solution’ and provided for by other Union policies or fulfilling the requirements set out in Paragraph 2;<br>(c) access to ICT technical specifications eligible for referencing in accordance with Article 13 of Regulation (EU) No 1025/2012;<br>(d) access to information on processing of personal data in the context of regulatory sandboxes referred to in Articles 11 and 12, if any high risks to the rights and freedoms of the data subjects, as referred to in Article 35(1) of Regulation (EU) 2016/679 and in Article 39 of Regulation (EU) 2018/1725, has been identified, as well as access to information on response mechanisms to promptly mitigate those risks. The published information may include a disclosure of the data protection impact assessment;<br>(e) fostering knowledge exchange between members of the Interoperable Europe Community, as set out in Article 16, such as providing a feedback system to express their views on measures proposed by the Interoperable Europe Board or express their interest to participate to actions related to the implementation of this Regulation;<br>(f) access to interoperability-related monitoring data referred to in Article 20;

					(g) allowing citizens and civil society organisations to provide feedback on the published content.<br><br>Art 8(2): The Interoperable Europe Board may propose to the Commission to publish on the portal other interoperability solutions or to have them referred to on the portal. Such solutions shall:<br>(a) not be subject to third party rights or contain personal data or confidential information;<br>(b) have a high-level of alignment with the Interoperable Europe solutions which may be proven by publishing the outcome of the interoperability assessment referred to in Article 3;<br>(c) use a licence that allows at least for the reuse by other public sector bodies or institutions, bodies or agencies of the Union or be issued as open source. An open source licence means a licence whereby the reuse of the software is permitted for all specified uses in a unilateral declaration by the right holder, and where the source codes of the software are made available for users;<br>(d) be regularly maintained under the responsibility of the owner of the interoperability solution.<br><br>Art 8(3): When a public sector body or an institution, body or agency of the Union provides a portal, catalogue or repository with similar functions, it shall take the necessary measures to ensure interoperability with the Interoperable Europe portal. Where such portals collect open source solutions, they shall allow for the use of the European Union Public Licence.
Europe	2024			REGULATION OF THE EUROPEAN PARLIAMENT AND OF THE COUNCIL laying down measures for a high level of public sector interoperability across the Union (Interoperable Europe Act)	Art 11(1): Regulatory sandboxes shall provide a controlled environment for the development, testing and validation of innovative interoperability solutions supporting the cross- border interoperability of network and information systems which are used to provide or manage public services to be delivered or managed electronically for a limited period of time before putting them into service.<br><br>Art 11(2): Regulatory sandboxes shall be operated under the responsibility of the participating public sector bodies and, where the sandbox entails the processing of personal data by public sector bodies, under the supervision of other relevant national authorities, or where the sandbox entails the processing of personal data by institutions, bodies, and agencies of the Union, under the responsibility of the European Data Protection Supervisor.<br><br>Art 11(3): The establishment of a regulatory sandbox as set out in paragraph 1 shall aim to contribute to the following objectives:<br>(a) foster innovation and facilitate the development and roll-out of innovative digital interoperability solutions for public services;<br>(b) facilitate cross-border cooperation between national competent authorities and synergies in public service delivery;<br>(c) facilitate the development of an open European GovTech ecosystem, including cooperation with small and medium enterprises and start-ups;<br>(d) enhance authorities' understanding of the opportunities or barriers to cross- border interoperability of innovative interoperability solutions, including legal barriers;<br>(e) contribute to the development or update of Interoperable Europe solutions.

Europe	2024			REGULATION OF THE EUROPEAN PARLIAMENT AND OF THE COUNCIL laying down measures for a high level of public sector interoperability across the Union (Interoperable Europe Act)	Art 12(1): The participating public sector bodies shall ensure that, to the extent the innovative interoperability solution involves the processing of personal data or otherwise falls under the supervisory remit of other national authorities providing or supporting access to data, the national data protection authorities and those other national authorities are associated to the operation of the regulatory sandbox. As appropriate, the participating public sector bodies may allow for the involvement in the regulatory sandbox of other actors within the GovTech ecosystem such as national or European standardisation organisations, notified bodies, research and experimentation labs, innovation hubs, and companies wishing to test innovative interoperability solutions. Cooperation may also be envisaged with third countries establishing mechanisms to support innovative interoperability solutions for the public sector.<br><br>Art 12(2): Participation in the regulatory sandbox shall be limited to a period that is appropriate to the complexity and scale of the project, and in any case not longer than 2 years from the establishment of the regulatory sandbox. The participation may be extended for up to one more year if necessary to achieve the purpose of the processing.<br><br>Art 12(3): Participation in the regulatory sandbox shall be based on a specific plan elaborated by the participants taking into account the advice of other national competent authorities or the European Data Protection Supervisor, as applicable. The plan shall contain as a minimum the following:<br>(a) description of the participants involved and their roles, the envisaged innovative interoperability solution and its intended purpose, and relevant development, testing and validation process;<br>(b) the specific regulatory issues at stake and the guidance that is expected from the authorities supervising the regulatory sandbox;<br>(c) the specific modalities of the collaboration between the participants and the authorities, as well as any other actor involved in the regulatory sandbox;<br>(d) a risk management and monitoring mechanism to identify, prevent and mitigate any risk;<br>(e) the key milestones to be completed by the participants for the interoperability solution to be considered ready to be put into service;<br>(f) evaluation and reporting requirements and possible follow-up;<br>(g) where personal data are processed, an indication of the categories of personal data concerned, the purposes of the processing for which the personal data are intended and the actors involved in the processing and their role.<br><br>Art 12(6): Personal data may be processed in the regulatory sandbox subject to the following cumulative conditions:<br>(a) the innovative interoperability solution is developed for safeguarding public interests in the area of a high level of efficiency and quality of public administration and public services;<br>(b) the data processed is limited to what is necessary for the functioning of the interoperability solution to be developed or tested in the sandbox, and the functioning cannot be effectively achieved by processing anonymised, synthetic or other non-personal data;<br>(c) there are effective monitoring mechanisms to identify if any high risks to the rights and freedoms of the data subjects, as referred to in Article 35(1) of Regulation (EU) 2016/679 and in Article 39 of Regulation

					(EU) 2018/1725, may arise during the operation of the sandbox, as well as a response mechanism to promptly mitigate those risks and, where necessary, stop the processing;<br>(d) any personal data to be processed are in a functionally separate, isolated and protected data processing environment under the control of the participants and only authorised persons have access to that data;<br>(e) any personal data processed are not to be transmitted, transferred or otherwise accessed by other parties that are not participants in the sandbox nor transferred to parties other than the participants of the sandbox;<br>(f) any processing of personal data does not affect the application of the rights of the data subjects as provided for under Union law on the protection of personal data, in particular in Article 22 of Regulation (EU) 2016/679 and Article 24 of Regulation (EU) 2018/1725;<br>(g) any personal data processed are protected by means of appropriate technical and organisational measures and deleted once the participation in the sandbox has terminated or the personal data has reached the end of its retention period;<br>(h) the logs of the processing of personal data are kept for the duration of the participation in the sandbox and for a limited period after its termination solely for the purpose of and only as long as necessary for fulfilling accountability and documentation obligations under Union or Member States legislation;<br>(i) a complete and detailed description of the process and rationale behind the training, testing and validation of the interoperability solution is kept together with the testing results as part of the technical documentation and transmitted to the Interoperable Europe Board;<br>(j) a short summary of the interoperability solution developed in the sandbox, its objectives and expected results are made available on the Interoperable Europe portal.<br><br>Art 12(9): The Commission is empowered to adopt implementing acts to set out the detailed rules and the conditions for the establishment and the operation of the regulatory sandboxes, including the eligibility criteria and the procedure for the application for, selection of, participation in and exiting from the sandbox, and the rights and obligations of the participants.<br><br>Art 12(10): Where a regulatory sandbox involves the use of artificial intelligence, the rules set out under Article 53 and 54 of the [proposal for a] Regulation of the European Parliament and of the Council laying down harmonised rules on artificial intelligence (Artificial Intelligence Act) and amending certain Union legislative acts shall prevail in case of conflict with the rules set out by the Regulation.

France			LOI n° 2018-493 du 20 juin 2018 relative à la protection des données personnelles (1)	LAW no. 2018-493 of June 20, 2018 relating to the protection of personal data (1)	Article 32<br>I.-Under the conditions provided for in Article 38 of the Constitution and in compliance with the provisions provided for in Titles I to III of this Law and in this Title, the Government is authorized to take by way of ordinance the measures relating of the field of law necessary:<br>1° To the rewriting of the entire law n° 78-17 of January 6, 1978 relating to computing, files and freedoms in order to make the formal corrections and the necessary adaptations the simplification and consistency as well as the simplicity of implementation by the persons concerned of the provisions which bring national law into conformity with Regulation (EU) 2016/679 of the European Parliament and of the Council of April 27, 2016 on the protection of natural persons with regard to the processing of personal data and on the free movement of such data, and repealing Directive 95/46/EC and transposing Directive (EU) 2016/680 of the European Parliament and of the Council of 27 April 2016 on the protection of natural persons with regard to the processing of personal data by competent authorities for the purposes of prevention, detection, investigation and prosecution of criminal offenses or execution of criminal sanctions, and thefree movement of this data, and repealing Council framework decision 2008/977/JHA, as resulting from this law;<br>2° To bring all of the legislation applicable to the protection of personal data into line with these changes, make any modifications that may be necessary to ensure compliance with the hierarchy of standards and the editorial consistency of the texts, harmonize the the state of the law, remedy any errors and omissions resulting from this law and repeal provisions that have become irrelevant;

France			Loi n° 78-17 du 6 janvier 1978 relative à l'informatique, aux fichiers et aux libertés	Law No. 78-17 of January 6, 1978 relating to data processing, files and freedoms	Article 1 Modified by Order no. 2018-1125 of December 12, 2018 - art. 1<br>It must be at the service of every citizen. Its development must take place within the framework of international cooperation. It must not infringe upon human identity, human rights, private life, or individual or public freedoms. The rights of individuals to decide and control the uses that are made of personal data concerning them and the obligations incumbent on persons who process this data are exercised within the framework of Regulation (EU) 2016/679 of the European Parliament and the Council of April 27, 2016, Directive (EU) 2016/680 of the European Parliament and of the Council of April 27, 2016 and this law<br><br>Article 2 Modified by Order no. 2018-1125 of December 12, 2018 - art. 1<br>This law applies to automated processing of all or part of personal data, as well as to non automated processing of personal data contained or intended to appear in files, when the person responsible meets the conditions provided for in this law. article 3 of this law, with the exception of processing carried out by natural persons for the exercise of strictly personal or domestic<br>activities.<br>A personal data file constitutes any structured set of personal data accessible according to determined criteria, whether this set is centralized, decentralized or distributed functionally or<br>geographically.<br>Unless otherwise provided, within the framework of this law, the definitions of Article 4 of Regulation (EU) 2016/679 of April 27, 2016 apply.<br><br>Article 3<br>Modified by Order no. 2018-1125 of December 12, 2018 - art. 1<br>I.-Without prejudice, with regard to processing operations falling within the scope of<br>Regulation (EU) 2016/679 of April 27, 2016, to the criteria provided for in Article 3 of this<br>regulation, all of the provisions of this law apply to the processing of personal data carried<br>out as part of the activities of an establishment of a data controller or a subcontractor on French<br>territory, whether or not the processing takes place in France.<br>II.-The national rules taken on the basis of the provisions of the same regulation referring to<br>national law the task of adapting or supplementing the rights and obligations provided for by<br>this regulation apply as long as the person concerned resides in France, including when the data<br>controller is not established in France<br><br>Article 4<br>Modified by Order no. 2018-1125 of December 12, 2018 - art. 1<br>Personal data must be:<br>1° Processed in a lawful, fair and, for processing under Title II, transparent manner with regard<br>to the person concerned;<br>2° Collected for specific, explicit and legitimate purposes, and not subsequently<br>processed in a manner incompatible with these purposes. However, further processing of data<br>for archival purposes in the public interest, for scientific or historical research purposes,<br>or for statistical purposes is considered compatible with the original purposes of the data<br>collection, if carried out in compliance with the provisions of Regulation (EU) 2016/679 of April 27,

					2016 and this law, applicable to such processing and if it is not used to make decisions with regard to the persons concerned;<br>3° Adequate, relevant and, with regard to the purposes for which they are processed, limited to what is necessary or, for processing falling under Titles III and IV, not excessive;<br>4° Accurate and, if necessary, kept up to date. All reasonable measures must be taken to ensure that personal data which are inaccurate, having regard to the purposes for which they are processed, are erased or rectified without delay;<br>5° Kept in a form allowing the identification of the persons concerned for a period not exceeding that necessary for the purposes for which they are processed.<br>However, personal data may be retained beyond this period to the extent that they are processed exclusively for archival purposes in the public interest, for scientific or historical research purposes, or for statistical purposes. The choice of data kept at<br>Machine Translated by Google<br>archival purposes in the public interest is operated under the conditions provided for in Article L. 212-3 of the Heritage Code;<br>6° Processed in such a way as to ensure appropriate security of personal data, including protection against unauthorized or unlawful processing and against accidental loss, destruction or damage, or access by unauthorized persons. authorized, using appropriate technical or organizational measures.<br><br>Article 5<br>Modified by Order no. 2018-1125 of December 12, 2018 - art. 1<br>Processing of personal data is only lawful if, and to the extent that, it meets at least one of the following conditions:<br>1° The processing, when it falls under Title II, has received the consent of the data subject, under the conditions mentioned in 11 of Article 4 and Article 7 of Regulation (EU) 2016/679 of April 27 2016 previously mentioned;<br>2° The processing is necessary for the performance of a contract to which the data subject is a party or for the execution of pre-contractual measures taken at the request of the data subject;<br>3° The processing is necessary for compliance with a legal obligation to which the data controller is subject;<br>4° The processing is necessary to safeguard the vital interests of the data subject or another natural person;<br>5° The processing is necessary for the execution of a mission of public interest or relating to the exercise of public authority vested in the controller;<br>6° Except for processing carried out by public authorities in the execution of their missions, processing is necessary for the purposes of the legitimate interests pursued by the controller or by a third party, unless the interests or freedoms and fundamental rights of the data subject which require protection of personal data, in particular when the data subject is a child.<br><br>Article 6<br>Modified by Order no. 2018-1125 of December 12, 2018 - art. 1<br>I.-It is prohibited to process personal data which reveal the alleged racial origin or ethnic origin, political

					opinions, religious or philosophical beliefs or trade union membership of a natural person or to process genetic data , biometric data for the purposes of uniquely identifying a natural person, data concerning health or data concerning the sex life or sexual orientation of a natural person.<br><br>Article 31<br>Modified by Order no. 2018-1125 of December 12, 2018 - art. 1<br>I.-The processing of personal data implemented on behalf of the State is authorized by order of the competent minister(s), taken after reasoned and published opinion of the National Commission for Information Technology and Freedoms, and:<br>1° Which concern state security, defense or public security;<br>2° Or whose purpose is the prevention, investigation, detection or prosecution of criminal offenses or the execution of criminal convictions or security measures.<br><br>Article 44<br>Modified by Order no. 2018-1125 of December 12, 2018 - art. 1<br>Article 6 does not apply if one of the conditions provided for in 2 of Article 9 of Regulation (EU) 2016/679 of April 27, 2016 is met, as well as for:<br>1° Treatments necessary for the purposes of preventive medicine, medical diagnoses, the administration of care or treatment, or the management of health services and implemented by a member of a health profession, or by another person on whom, because of his or her functions, the obligation of professional secrecy is imposed, the violation of which is punishable by article 226-13 of the penal code;<br>2° Statistical processing carried out by the National Institute of Statistics and Economic Studies or one of the ministerial statistical services in compliance with Law No. 51-711 of June 7, 1951 on obligation, coordination and secrecy in matters of statistics, after advice from the National Council for Statistical Information;<br>3° Processing operations involving data concerning health justified by the public interest and in accordance with the provisions of Section 3 of Chapter III of this Title;<br>4° Processing in accordance with the standard regulations mentioned in c of 2° of I of Article 8 implemented by employers or administrations which relate to biometric data strictly necessary for the control of access to workplaces as well as 'devices and applications used as part of the missions entrusted to employees, agents, interns or service providers;<br>5° Processing operations relating to the reuse of public information appearing in the decisions mentioned in article L. 10 of the code of administrative justice and in article L. 111-13 of the code of judicial organization, provided that these processing operations have neither the purpose nor the effect of allowing the reidentification of the persons concerned;<br>6° Processing operations necessary for public research within the meaning of Article L. 112-1 of the Research Code, provided that reasons of significant public interest make them necessary, under the conditions provided for by g of 2 of Article 9 of Regulation (EU) 2016/679 of April 27, 2016, after a reasoned and published opinion from the National Commission for Information Technology and Liberties delivered in accordance with the procedures provided for in Article 34 of this law.

					Article 64<br>Modified by Order no. 2018-1125 of December 12, 2018 - art. 1<br>When the exercise of the right of access applies to personal health data, these may be communicated to the data subject, according to their choice, directly or through a doctor designated by them. for this purpose, in compliance with the provisions of article L. 1111-7 of the public health code.

France			Loi n° 78-17 du 6 janvier 1978 relative à l'informatique, aux fichiers et aux libertés	Law No. 78-17 of January 6, 1978 relating to data processing, files and freedoms	Chapter IV: Rights and obligations specific to processing in the electronic communications sector<br><br>Article 81<br>Modified by Order no. 2018-1125 of December 12, 2018 - art. 1<br>The rights and obligations mentioned in Chapters II and III apply subject to the specific provisions of this chapter.<br><br>Article 82<br>Modified by Order no. 2018-1125 of December 12, 2018 - art. 1<br>Any subscriber or user of an electronic communications service must be informed clearly and completely, unless previously informed by the data controller or his representative:<br>1° The purpose of any action tending to access, by electronic transmission, information already stored in its electronic communications terminal equipment, or to enter information in this equipment;<br>2° The means available to him to oppose it.<br>These accesses or registrations can only take place on condition that the subscriber or user has expressed, after having received this information, their consent which may result from appropriate parameters of their connection device or any other device placed under their control. control. These provisions are not applicable if access to information stored in the user's terminal equipment or the recording of information in the user's terminal equipment:<br>1° Either, has the exclusive purpose of enabling or facilitating communication by electronic means;<br>2° Either, is strictly necessary for the provision of an online communication service at the express request of the user.<br><br>Article 83<br>Modified by Order no. 2018-1125 of December 12, 2018 - art. 1<br>I.-This article applies to the processing of personal data implemented in the context of the provision to the public of electronic communications services on electronic communications networks open to the public, including those supporting devices data collection and identification. For the purposes of this article, a personal data breach means any security breach resulting in accidental or unlawful destruction, loss, alteration, disclosure or unauthorized access to personal data. personal nature subject to processing in the context of the provision of electronic communications services to the public.<br>II.-In the event of a personal data breach, the provider of electronic communications services accessible to the public notifies, without delay, the National Commission for Information<br>Technology and Liberties. When this violation may harm the personal data or privacy of a subscriber or another natural person, the provider shall also notify the interested party without delay.<br>Notification of a personal data breach to the data subject is, however, not necessary if the National Commission for Information Technology and Liberties has established that appropriate protective measures have been implemented by the provider in order to render the data incomprehensible to any person not authorized to have access to it and were applied to the data affected by said violation.<br>Failing this, the National Commission for Information Technology and Liberties may, after examining the seriousness of the violation, give formal notice to the supplier to also inform the interested parties.

					III.-Each electronic communications service provider maintains an inventory of personal data violations, including their modalities, their effect and the measures taken to remedy them, and<br>keeps it available to the commission
France	2018		Décret n° 2018-1144 du 12 décembre 2018 modifiant le décret n° 2017-693 du 3 mai 2017 créant un conseil scientifique sur les processus de radicalisation	Decree No. 2018-1144 of December 12, 2018 amending Decree No. 2017-693 of May 3, 2017 creating a scientific council on radicalization processes	Article 1<br>Version in force since December 15, 2018<br>Modified by Decree No. 2018-1144 of December 12, 2018 - art. 2<br><br>The scientific council on radicalization processes is chaired by the Prime Minister or his representative.<br>The vice-president is designated by order of the minister responsible for research published in the Official Journal of the French Republic from among the members mentioned in c, d and f of article 2.<br>In order to promote prevention and the fight against the processes of radicalization, and without prejudice to the respective responsibilities of ministers in this area, the scientific council on radicalization processes is responsible for facilitating interactions between public administrations and researchers in the human and social sciences, for proposing priority areas of research on radicalization questions of radicalization, to encourage the organization of general reviews of research on radicalization and to disseminate good practices, to encourage reflection on access to data of a sensitive nature in matters of radicalization and to contribute to the valorization of research results in the human and social sciences and their reuse for the benefit of public policies for prevention and the fight against radicalization.

					The council can propose to the Prime Minister any measure aimed at improving the policy of prevention and fight against radicalization.

France			Code de la propriété intellectuelle	Intellectual Property Code	Article R331-44<br><br>Transferred by Decree n°2021-1853 of December 27, 2021 - art. 1 Created by Decree No. 2010-872 of July 26, 2010 - art. 1<br>The public prosecutor informs the rights protection commission of the follow-up given to the procedure transmitted.<br><br>Article R331-45<br>Transferred by Decree n°2021-1853 of December 27, 2021 - art. 1 Created by Decree No. 2010-872 of July 26, 2010 - art. 1<br>The rights protection commission is the recipient of enforceable decisions containing a penalty of suspension of access to an online communication service pronounced in application of articles L. 335-7, L. 335-7-1 and R. 335 -5 .<br><br>Article R331-46<br>Transferred by Decree n°2021-1853 of December 27, 2021 - art. 1 Created by Decree No. 2010-872 of July 26, 2010 - art. 1<br>The rights protection commission informs by letter delivered against signature the person whose activity is to offer access to communication services to the public online of the suspension sentence pronounced against his subscriber.<br>Pursuant to Article L. 331-28 , the person whose activity is to provide access to communication services to the public online informs, by letter delivered against signature, the rights protection commission of the date at which the suspension period began. The rights protection commission informs the automated criminal record of the execution of the measure.<br>If the person whose activity is to offer access to communication services to the public online fails to implement the suspension penalty which has been notified to him, the commission for the protection of rights deliberates, under the conditions of majority defined in article R. 331-42 , for the purposes of informing the public prosecutor of the facts likely to constitute the offense referred to in the sixth paragraph of article L. 335-7 .<br><br>Article R331-35<br>Modified by Decree No. 2010-872 of July 26, 2010 - art. 1<br>To be admissible, referrals addressed to the rights protection commission of the High Authority by regularly constituted professional defense organizations, rights collection and distribution societies and the National Center for Cinema and Animated Images in the conditions provided for in article L. 331-24 must include:<br><br>1° Personal data and information mentioned in 1° of the annex to Decree No. 2010-236 of March 5, 2010 relating to the automated processing of personal data authorized by article L. 331-29 of the code of intellectual property called “System for managing measures for the protection of works on the internet”;<br><br>2° A declaration on honor according to which the author of the referral has the capacity to act on behalf of the holder of rights to the work or protected object concerned by the facts.

					Upon receipt of the referral, the rights protection commission acknowledges receipt electronically.<br><br>Article R331-36<br>Modified by Decree No. 2010-872 of July 26, 2010 - art. 1<br>The reports drawn up by the sworn and approved agents mentioned in article L. 331-24 may be drawn up in electronic form. In this case, use is made of a secure electronic signature under the conditions provided for by article 1316-4 of the civil code and decree no. 2001-272 of March 30, 2001 taken for the application of article 1316 -4 of the civil code and relating to electronic signature.<br><br>Article R331-37<br>Modified by Decree No. 2010-872 of July 26, 2010 - art. 1<br>The electronic communications operators mentioned in Article L. 34-1 of the Postal and Electronic Communications Code and the service providers mentioned in 1 and 2 of I of Article 6 of Law No. 2004-575 of June 21, 2004 for confidence in the digital economy are required to communicate the personal data and information mentioned in 2° of the annex to Decree No. 2010-236 of March 5, 2010 within eight days following transmission by the commission for the protection of the rights of technical data necessary for the identification of the subscriber whose access to online public communication services has been used for the purposes of reproduction, representation, making available or communication to the public of protected works or objects without the authorization of the holders of the rights provided for in Books I and II when it is required.<br><br>These operators and service providers are also required to provide the documents and copies of the documents mentioned in the third and fourth paragraphs of Article L. 331-21 within fifteen days following the request made to them by the protection commission. Rights.<br><br>Article R331-38<br>Transferred by Decree n°2021-1853 of December 27, 2021 - art. 1 Created by Decree No. 2010-872 of July 26, 2010 - art. 1<br>Violation of the provisions of article R. 331-37 is punishable by the fine provided for fifth class contraventions .<br>Repeat offenses provided for in this article are punishable in accordance with articles 132-11 and 132-15 of the penal code.<br><br><br>Article R331-40<br>Transferred by Decree n°2021-1853 of December 27, 2021 - art. 1 Created by Decree No. 2010-872 of July 26, 2010 - art. 1<br>When, within one year following the presentation of the recommendation mentioned in the first paragraph of Article L. 335-7-1 , the commission for the protection of rights is informed of new facts likely to constitute gross negligence defined in Article R. 335-5, it informs the subscriber, by letter delivered against signature, that these facts are subject to prosecution. This letter invites the interested party to present their

					observations within fifteen days. It specifies that he may, within the same period, request a hearing pursuant to article L. 331-21-1 and that he has the right to be assisted by counsel. She also invites him to specify his family responsibilities and his resources.<br><br>The commission may, on its own initiative, summon the person concerned for a hearing. The summons letter specifies that he has the right to be assisted by counsel.<br><br>Article R331-41<br>Transferred by Decree n°2021-1853 of December 27, 2021 - art. 1 Created by Decree No. 2010-872 of July 26, 2010 - art. 1<br>A report of the hearing of the interested party is drawn up by a member of the commission for the protection of rights or by an authorized and sworn agent in application of article R. 331-16.<br><br>The report is signed by the person concerned and by his or her counsel, by the person carrying out the hearing as well as by the person who wrote it. If the person interviewed or his or her counsel does not want to sign the report, this will be noted in it.<br>A copy of the report is given to the interested party.<br><br>Article R331-42<br>Created by Decree No. 2010-872 of July 26, 2010 - art. 1<br>The rights protection commission notes by a deliberation taken by a majority of at least two votes that the facts are likely to constitute the offense provided for in article R. 335-5 or the offenses provided for in articles L. 335- 2, L. 335-3 and L. 335-4.<br><br>However, when only two members of the commission are present and in the event of a tie of votes, the examination of the procedure is postponed to the first plenary session of the commission.<br><br>Article R331-43<br>Created by Decree No. 2010-872 of July 26, 2010 - art. 1<br>The deliberation of the commission noting that the facts are likely to constitute an offense, to which are attached, depending on the case, a summary report of all the facts and procedure as well as all useful documents, is transmitted to the prosecutor of the Republic before the competent high court.<br>The rights protection commission notifies the authors of the referrals sent to it under the conditions provided for in article L. 331-24 of the transmission of the procedure to the public prosecutor.

France	2023		Arrêté du 14 août 2023 portant création d'un service à compétence nationale dénommé « Agence du numérique des forces de sécurité intérieure »	Order of August 14, 2023 creating a service with national competence called "Digital Agency of the Forces ofsecurityinterior »	Article 1<br>A service with national competence called the "Digital Agency of the Internal Security Forces" is created, reporting jointly to the Director General of the National Gendarmerie and the Director General of the National Police.<br><br>Article 2<br>This agency is responsible for the development, implementation and security of information systems, digital equipment and applications for the benefit of internal security forces.<br>It is competent in the construction and management of infrastructure, terminals and peripheral equipment for services and units, national gendarmerie personnel and national police officers.<br>In collaboration with operational departments and services and for their benefit, it designs and leads projects relating to information, communication and command systems, as well as in the field of related technologies. Without prejudice to shared or interministerial projects carried out by other digital players, it ensures project management of the operational systems of the national police and the national gendarmerie. Depending on the projects, it can also have the role of project management. It ensures and organizes the convergence of the information and communication systems as well as the digital tools of the two forces when relevant and relies as far as possible on shared ministerial and interministerial solutions.<br>Its action falls within the framework of the governance of the Ministry of the Interior and Overseas Territories in terms of information and communication systems defined by the Deputy Secretary General in charge of digital technology.<br>It leads the technological innovation policy of the Ministry of the Interior and Overseas Territories with regard to internal security missions.

France		2024	Code des postes et des communications électroniques	Postal and Electronic Communications Code	Article L34-1<br>Modified by LAW n°2021-998 of July 30, 2021 - art. 17<br><br>I. – This article applies to the processing of personal data in the context of the provision of electronic communications services to the public; it applies in particular to networks that support data collection and identification devices.<br><br>II. – Electronic communications operators, and in particular persons whose activity is to offer access to communication services to the public online, erase or make anonymous, subject to II bis to VI, data relating to electronic communications .<br>Persons who provide electronic communications services to the public shall establish, in compliance with the provisions of the preceding paragraph, internal procedures enabling them to respond to requests from the competent authorities.<br>Persons who, as a main or secondary professional activity, offer the public a connection allowing online communication via network access, including free of charge, are subject to compliance with the provisions applicable to electronic communications operators under this article.<br><br>II bis.-Electronic communications operators are required to keep:<br><br>1° For the purposes of criminal proceedings, the prevention of threats against public security and the safeguarding of national security, information relating to the civil identity of the user, until the expiry of a period of five years from the end of the validity of their contract;<br>2° For the same purposes as those set out in 1° of this II bis, other information provided by the user when subscribing to a contract or creating an account as well as information relating to payment, up to 'at the end of a period of one year from the end of the validity of his contract or the closure of his account;<br>3° For the purposes of combating crime and serious delinquency, preventing serious threats to public security and safeguarding national security, technical data making it possible to identify the source of the connection or those relating to the terminal equipment used, until the expiry of a period of one year from the connection or use of the terminal equipment.<br><br>III.-For reasons relating to the protection of national security, when a serious threat, current or foreseeable, against the latter is noted, the Prime Minister may order by decree electronic communications operators to keep, for a period of one year, certain categories of traffic data, in addition to those mentioned in 3° of II bis, and location data specified by decree of the Council of State.<br><br>The Prime Minister's injunction, the duration of which cannot exceed one year, may be renewed if the conditions provided for its issuance continue to be met. Its expiration has no impact on the retention period of the data mentioned in the first paragraph of this III.<br><br>III bis.-The data retained by operators in application of this article may be the subject of a rapid conservation order by the authorities having, in application of the law, access to data relating to electronic communications to for the purposes of preventing and suppressing crime, serious delinquency and other

					serious breaches of the rules for which they are responsible for ensuring compliance, in order to access this data.<br><br>IV. – For the purposes of invoicing and payment of electronic communications services, operators may, until the end of the period during which the invoice can be legally contested or proceedings initiated to obtain payment, use, retain and, where applicable, transmit to third parties directly concerned with invoicing or collection the categories of technical data which are determined, within the limits set by the VI, according to the activity of the operators and the nature of the communication, by decree in the Council of State taken after advice from the National Commission for Information Technology and Liberties.<br><br>Operators may also process traffic data with a view to marketing their own electronic communications services or providing value-added services, if subscribers expressly consent to this and for a specific period. This duration cannot, under any circumstances, be greater than the period necessary for the provision or marketing of these services. They may also retain certain data to ensure the security of their networks.<br><br>V. – Without prejudice to the provisions of III and IV, the data making it possible to locate the user's terminal equipment cannot be used during the communication for purposes other than its routing, nor be retained and processed after the communication. completion of the communication only with the consent of the subscriber, duly informed of the categories of data in question, the duration of the processing, its purposes and whether or not these data will be transmitted to third party service providers. The subscriber can withdraw his consent at any time and free of charge, apart from the costs linked to the transmission of the withdrawal. The user can suspend the consent given, by a simple and free means, apart from the costs linked to the transmission of this suspension. Any call intended for an emergency service constitutes the user's consent until the completion of the emergency operation it triggers and only to enable it to be carried out.<br><br>VI. – The data stored and processed under the conditions defined in II bis to V relate exclusively to the identification of people using the services provided by operators, to the technical characteristics of the communications provided by the latter and to the location of terminal equipment.<br>They cannot under any circumstances relate to the content of correspondence exchanged or information consulted, in any form whatsoever, in the context of these communications.<br>A decree in the Council of State, taken after advice from the National Commission for Information Technology and Liberties and the Regulatory Authority for Electronic Communications, Posts and Press Distribution, determines, depending on the activity of the operators and the nature of the communications, the information and categories of data retained in application of II bis and III as well as the terms of compensation for identifiable and specific additional costs of the services provided in this respect, at the request of the State, by the operators .<br>The storage and processing of this data is carried out in compliance with the provisions of Law No. 78-17 of January 6, 1978 relating to data processing, files and freedoms.<br>The operators take all measures to prevent the use of this data for purposes other than those provided for in this article.

France			Décret n° 2022-367 du 15 mars 2022 autorisant la mise en œuvre de traitements automatisés de données à caractère personnel dénommés « Base ministérielle PPST »	Decree No. 2022-367 of March 15, 2022 authorizing the implementation of automated processing of personal data called “PPST ministerial base”	Article 1<br><br>The minister responsible for energy, the minister responsible for transport, the minister responsible for the environment, the minister responsible for the sea, the minister responsible for health, the minister responsible for the economy, the minister responsible for higher education and research, and the minister responsible for agriculture are each authorized to implement automated processing of personal data called the “PPST ministerial base”.<br>The purpose of these processing operations is to centralize the investigation by ministers of the requests for opinions submitted to them, in application of articles 413-7 and R. 413-5-1 of the penal code, by the heads of department, establishment or company called upon to rule on a request for authorization of access to a zone with restrictive regimes in order to ensure that essential elements of the scientific or technical potential of the nation are not subject to capture likely to weaken its means of defense, compromise its security or harm its other fundamental interests, or be diverted for the purposes of terrorism, proliferation of weapons of mass destruction and their vectors or contribution to increase in military arsenals.
France			Arrêté du 31 mars 2021 portant création d'un traitement automatisé de données à caractère personnel dénommé « SI Honorabilité »	Order of March 31, 2021 creating automated processing of personal data called “SI Honorability”	Article 1<br><br>Modified by Order of January 18, 2022 - art. 1<br><br>I. - A processing of personal data is created, called "SI Honorability", the responsibility of which is ensured jointly by the sports department, the youth department, popular education and associative life and the general secretariat of the ministries responsible for social affairs.<br><br>The purpose of this processing is to enable the authorized persons mentioned in Article 4 to carry out a check of the good repute of persons subject to the provisions of Article L. 133-6 of the Code of Social Action and Families and of articles L. 212-9 and L. 322-1 of the sports code in order to ensure that their continued activity does not present risks or dangers for the health and physical or moral safety of minors or practitioners in application of articles L. 227-10 and L. 227-11 of the social action and families code and article L. 212-13 of the sports code .<br><br>It also aims to allow the authorized persons mentioned in Article 4 to carry out a check of the registration in the automated national judicial file of perpetrators of sexual or violent offenses (FIJAISV) of persons intervening with minors in the context of activities falling under the control of regional health agencies in application of 2° of article L. 1431-2 of the public health code.<br><br>II. - The processing is made up of a “SI Deposit” portal and a “SI Return” portal.<br><br>The “SI Dépose” portal collects the information necessary to query the national criminal record and the automated national judicial file of perpetrators of sexual or violent offenses (FIJAISV). In addition, this portal makes it possible, where applicable, to collect the information necessary to query the database of prohibited executives which lists the people subject to a suspension measure, an injunction to cease

					practicing or prohibited from exercising their activity in application of articles L. 227-10, L. 227-11 of the social action and families code and article L. 212-13 of the sports code.<br><br>The “SI Retour” portal allows you to receive information from the files queried by the “SI Dépose” in order to verify the good repute of persons as provided for in I. In addition, this portal allows you to manage and, if necessary, consult the database of prohibited executives which lists the persons subject to a suspension measure, an injunction to cease practicing or a ban on exercising their activity in application of articles L. 227-10, L. 227 -11 of the social action and families code and article L. 212-13 of the sports code.

France			Arrêté du 21 septembre 2022 relatif au traitement automatisé de données à caractères personnel dénommé « Champollion »	Order of September 21, 2022 relating to the automated processing of personal data called “Chamollion”	Article 1<br><br>A processing of personal data called “Chamollion” is created for which the ministerial administrator of data, algorithms and source codes mentioned in the aforementioned decree of August 17, 2021 is responsible for processing. This processing is implemented for the execution of a mission of public interest, in accordance with e of 1 of article 6 of Regulation (EU) 2016/679 of April 27, 2016 mentioned above.<br><br>Article 2<br><br>The purpose of the “Chamollion” processing is to structure and make available to administrations in the field of work, employment and integration, data for the accomplishment of their missions.<br><br>The processing makes it possible to improve the circulation and use of the aforementioned data with a view to:<br><br>1° Simplify administrative procedures, create teleservices and devices to inform people of their right to possible benefit from a service or advantage and to possibly grant them said services or advantages;<br>2° Promote the use of artificial intelligence and its development, in particular by developing tools, benchmarks and methodologies constituting predictive models in the implementation of public policies;<br>3° Participate in experiments relating to the use of data to strengthen the effectiveness of public policies, contribute to the good management of public funds, improve the quality of services provided to users, contribute to the transparency of the ministry's public policies , and stimulate research and innovation.<br><br>Article 3<br>The categories of personal data that can be recorded in automated processing come from the nominative social declaration mentioned in Article L. 133-5-3 of the Social Security Code , processing falling within the scope of 2° of Article 6 of the aforementioned Decree No. 2013-727 , and processing operations for which the administrations in the field of work, employment and integration are responsible for processing or recipients of the data.<br>These categories of data are:<br>1° Relating to civil status;<br>2° Relating to family situation;<br>3° Relating to professional and extra-professional life;<br>4° Economic, financial and social.<br>The data is collected directly or indirectly by the employment and vocational training delegation, the general labor directorate, the animation, research and statistics directorate, and the digital directorate of the ministries responsible for social affairs.

France	2010	2022	Décret n° 2010-236 du 5 mars 2010 relatif au traitement automatisé de données à caractère personnel autorisé par l'article L. 331-23 du code de la propriété intellectuelle dénommé " Système de gestion des mesures pour la protection des œuvres sur internet	Decree No. 2010-236 of March 5, 2010 relating to the automated processing of personal data authorized by Article L. 331-23 of the Code ofproperty intellectualcalled “System for managing measures for the protection of works on the internet”	Article 1<br>Modified by Decree n°2021-1823 of December 24, 2021 - art. 3<br><br>The processing of personal data called “System for managing measures for the protection of works on the internet” has the purpose of implementation, by the member of the Audiovisual and Digital Communication Regulatory Authority designated in application of IV of article 4 of law no. 86-1067 of September 30, 1986 relating to freedom of communication:<br><br>1° The measures provided for in Book III of the legislative part of the intellectual property code (title III, chapter I, section 3, subsection 3, paragraph 1) and book III of the regulatory part of the same code (title III, chapter I, section 2, subsection 2);<br><br>2° Referrals to the public prosecutor of facts likely to constitute offenses provided for in articles L. 335-2, L. 335-3, L. 335-4 and R. 335-5 of the same code as well as the informing professional defense organizations and collective management organizations of these referrals;<br><br>The purpose of this processing is also the implementation by the Audiovisual and Digital Communication Regulatory Authority of the measures for notification of penalties provided for in Articles L. 335-7 and L. 335-7-1 of the same code.<br><br>Annex:<br>The personal data and information recorded in the processing called System for managing measures for the protection of works on the internet are as follows:<br><br>1° Personal data and information from regularly constituted professional defense organizations, collective management organizations, the National Center for Cinema and Animated Images, bailiff's reports established at the request of a beneficiary as well as than those coming from the public prosecutor: ----<br><br>2° Personal data and information relating to the subscriber collected from electronic communications operators in application of article L. 34-1 of the postal and electronic communications code : ----<br><br>3° Recommendations by electronic means and recommendations by letter delivered against signature or by any other means suitable for establishing proof of the date of presentation provided for in Article L. 331-20 of the Intellectual Property Code as well as letters and observations from subscribers receiving recommendations;<br><br>4° Referrals to the public prosecutor relating to facts likely to constitute offenses provided for in articles L. 335-2 , L. 335-3 , L. 335-4 , L. 335-7 , R. 331-8 , R 331-10 and R. 335-5 of the Intellectual Property Code as well as information letters from professional defense organizations, collective management organizations and rights holders;

					5° Enforceable court decisions comprising additional penalties for suspension of access to an online communication service and their notification to electronic communications operators, pursuant to Article R. 331-17 of the Intellectual Property Code .
France			Arrêté du 29 juin 2021 portant création du comité stratégique des données de santé	Order of June 29, 2021 creating the strategic data committee of health	Article 1<br>A strategic committee is established under the Minister responsible for Health. This committee provides the Minister with guidance and decision-making elements relating to the implementation and development of the national health data system.<br>Provided for by article R. 1461-10 of the public health code , the strategic committee is notably responsible for:<br><br>- propose guidelines on the development of the national health data system, and its legislative and regulatory developments;<br>- identify the existing databases which are intended to be registered in the order provided for in I of article R. 1461-2 of the public health code , recommend their registration and, if necessary, issue an opinion on the order of priority of their registration;<br>- identify the categories of missing data and make recommendations regarding the collection or production of this data to data producers;<br>- issue recommendations to encourage the sharing of data relating to the national health data system, taking into account in particular its financial and legal aspects;<br>- conduct and disseminate prospective reflection on the legal framework relating to the collection and sharing of data relating to the national health data system.

France			Décret n° 2021-848 du 29 juin 2021 relatif au traitement de données à caractère personnel dénommé « système national des données de santé »	Decree No. 2021-848 of June 29, 2021 relating to the processing of personal data called “national data system of health»	Article R1461-1<br>Version in force since July 1, 2021 Modified by Decree n°2021-848 of June 29, 2021 - art. 1<br>The processing of personal data called the national health data system (SNDS), established in article L. 1461-1 , is implemented by the Health Data Platform, mentioned in article L. 1462- 1, and the National Health Insurance Fund under the conditions established by this chapter.<br><br>Its purpose, in application of the provisions of Article L. 1461-1, is to enable the data it collects to be made available under the conditions defined in Articles L. 1461-2 to L. 1461-6 in order to contribute :<br>1° To information on health as well as on the provision of care, medico-social care and their quality, the orientation of users in the health system, by allowing the comparison of care practices, equipment and prices of establishments and health professionals;<br>2° The definition, implementation and evaluation of health and social protection policies, by promoting the identification of patients' care pathways, the monitoring and evaluation of their state of health and their consumption of care and social support services, analysis of patients' social coverage, monitoring of care consumption based on public health indicators or health risks;<br>3° Knowledge of health expenditure, health insurance expenditure and medico-social expenditure, by making it possible to analyze the expenditure of health insurance schemes by geographical area, by nature of expenditure, by category of professionals or prescribers and by professional or establishment, health insurance expenditure with regard to the sectoral expenditure objectives set, within the framework of the national health insurance expenditure objective, by social security financing laws, quantitative analysis of the determinants of healthcare provision and the measurement of their impacts on the evolution of health insurance expenditure;<br>4° To inform health professionals, health or medico-social structures and establishments about their activity, by allowing the transmission to healthcare providers of relevant information relating to their activity, their revenues and, if where applicable, to their prescriptions and the provision to their representatives of data which does not reveal the identity of the health professionals;<br>5° Health surveillance, monitoring and security, by developing observation of the state of health of populations, evaluation and production of indicators relating to the state of health of the population and analysis of their variation in time and space, the detection of unusual health events that may represent a threat to public health and the evaluation of their possible links with exposure factors and the evaluation of public health actions;<br>6° Research, studies, evaluation and innovation in the fields of health and medico-social care.<br><br>Data from the National Health Data System are hosted within the European Union. No transfer of personal data may be carried out outside the European Union, except in the case of occasional access to data by persons located outside the European Union, for a purpose falling under 1° of I of article L. 1461-3.<br><br>Article R1461-2<br>Modified by Decree n°2021-848 of June 29, 2021 - art. 1<br><br>I.-The national health data system includes a main base covering the entire population and a set of databases not covering the entire population called “catalogue”.

					The main database brings together the data mentioned in 1° to 4° of I of article L. 1461-1 , gradually supplemented by data mentioned in 5° to 11° of I of this same article. The catalog databases include data mentioned in 1° to 11° of I of the aforementioned article. The data from surveys in the field of health referred to in 8° of I of the aforementioned article are those which are governed by the provisions of Law No. 51-711 of June 7, 1951 on the obligation, the coordination and secrecy in statistical matters.<br>An order from the Minister responsible for health, taken after consulting the National Commission for Information Technology and Liberties, lists the data falling under 1° to 11° of I of Article L. 1461-1 which feeds the main database , sets the conditions for supplying this base and designates the databases appearing in the catalog. It is updated according to data availability.<br>II.- May be constituted by the Health Data Platform or the National Health Insurance Fund from the databases of the national health data system:<br>1° Anonymous datasets made available to the public in the conditions provided for in the first paragraph of article L. 1461-2 ;<br>2° Aggregated and semi-aggregated data sets adapted to different types of research, studies or evaluation. Semi-aggregated data is individualized for healthcare professionals or establishments and aggregated for care recipients;<br>3° Samples, including all or part of the data relating to the people from whom they come.<br>The list of these datasets and the characteristics of the samples are published on the Health Data Platform website when they are part of the scope of data made available by the Health Data Platform. Their updating is the subject of information from the National Commission for Information Technology and Liberties.<br><br>III.-The Health Data Platform and the National Health Insurance Fund make data available to the organizations responsible for the processing provided for in 1° and 2° of I of Article L. 1461-3 within a reasonable period of time .<br><br>IV.-Without prejudice to the provisions of I, the organizations responsible for the data supplying the national health data system may make them available to other data controllers under the conditions provided for in articles L. 1461-1 to L. 1461 -6.<br><br>Article R1461-3 Modified by Decree n°2021-848 of June 29, 2021 - art. 1<br>I.-The Health Data Platform and the National Health Insurance Fund are the joint managers of the national health data system.<br>II.-The National Health Insurance Fund is responsible for:<br>1° For gathering the data mentioned in 1° to 4° of I of Article L. 1461-1 for the constitution of the main database;<br>2° The storage and provision of data from the main database;<br>3° Pseudonymization operations in the context of:<br>a) The constitution of the main database and the catalog provided for in I of article R. 1461-2;<br>b) The exercise of the rights provided for by article R. 1461-9 ;<br>c) Matching databases with the national health data system.

					III.-The Health Data Platform is responsible for:<br>1° Enriching the main database with data mentioned in 5° to 11° of I of Article L. 1461-1 and matching the databases catalog data with the main database;<br>2° The storage and provision of data from the main database and all of the catalog databases;<br>3° Operations of pseudonymization of the data which it ensures is made available.<br>It can also contribute to the creation of catalog databases.<br>To carry out its missions, the Platform holds a copy of the main database held by the National Health Insurance Fund, as well as a copy of the databases listed in the catalog.<br>It cannot, for the enrichment of the main database and the constitution of the catalog, have the names and first names of people, their registration number in the National Identification Directory of natural persons, nor their address.<br>IV.-The National Institute of Statistics and Economic Studies and the National Old Age Insurance Fund may, in accordance with Article 1 of Decree No. 2018-390 of May 24, 2018 relating to the processing of personal data called “national identifier management system” and article 7 of decree no. 82-103 of January 22, 1982 relating to the national directory for the identification of natural persons, contribute as a subcontractor to the necessary matching operations the creation of bases as part of the implementation of the national health data system.<br>In this context, they do not have access to any information other than that relating to the surnames, first names, sex, date and place of birth of the individual whose registration number in the National Identification Directory is to be reconstructed. natural persons.<br>V.-Persons authorized to access personal data from the national health data system are responsible for the processing carried out using this data.<br><br>Article R1461-4<br>Modified by Decree n°2021-848 of June 29, 2021 - art. 1<br><br>I.-The categories of data collected within the national health data system are as follows:<br>1° Information relating to beneficiaries of care and medico-social services:<br>a) The pseudonym, consisting of a non-significant code obtained by an irreversible cryptographic process from the registration number in the National Identification Directory of natural persons;<br>b) Sex, month and year of birth, order of birth, municipality of residence and its code as well as sub-municipal location data, excluding any address;<br>c) Medical-administrative information, in particular, if applicable, that linked to long-term illnesses appearing on the list mentioned in Article D. 160-4 of the Social Security Code , and to occupational diseases;<br>d) If applicable, information relating to the death:<br>i) The date of death;<br>ii) The municipality and place of death;<br>iii) The causes and circumstances of death;<br>iv) Family situation and occupation at the date of death;

					2° Information relating to compulsory health insurance organizations and, where applicable, to supplementary health insurance organizations involved in the financial support of the beneficiary of care and services:<br>a) Identification of organisms;<br>b) The characteristics of coverage by compulsory health insurance and, where applicable, supplementary health insurance organizations;<br>3° Information relating to health, medico-social and financial care associated with each beneficiary:<br>a) Information relating to services provided for outpatient care: nature of the procedures, goods and services, codes of medical procedures, medical devices, pharmacy, biology and medical transport procedures, date of care, date of pregnancy;<br>b) Information relating to services and stays provided in a health establishment or medico-social establishment and service, including external care and emergency reception, as well as medical diagnoses associated with the description of the care;<br>c) The amount of the act or service, its rating or coefficient, the price applied and the part covered by compulsory health insurance, where applicable the modulation or exemption of the co-payment and its reason, as well as the date of reimbursement or payment;<br>d) Accounting data relating to services covered by compulsory health insurance;<br>e) The type of contract, the nature of the risks covered and information relating to the coverage provided by supplementary health insurance if applicable;<br>4° Information relating to health professionals and services involved in the care of the beneficiaries mentioned in I:<br>a) The identification number of the professional and, where applicable, of the establishment;<br>b) Gender, date of birth;<br>c) The profession and, if applicable, the specialty, the mode of practice, the conventional status, the affiliation fund;<br>d) The place where the procedure was carried out by the healthcare professional;<br>5° Medical and social information relating to the situation of people with disabilities transmitted to the National Solidarity Fund for Autonomy as part of the information system mentioned in Article L. 247-2 of the Code of social action and families :<br>a) Data relating to the disability and care of the persons concerned;<br>b) Data concerning the decisions mentioned in Article L. 241-6 of the Social Action and Families Code ;<br>c) Data relating to the follow-up given to the guidelines issued by the Commission on the Rights for the Autonomy of Persons with Disabilities, in particular with establishments and services likely to accommodate or support the persons concerned;<br>6° Information relating to work stoppages and cash benefits: data relating to work stoppages, the payment of daily allowances for illness, maternity, paternity, work accidents and occupational illnesses and the payment of pensions disability, annuities following a work accident or occupational illness or death benefits.<br>7° Information relating to the health, social and environmental conditions, lifestyle habits and economic context of the persons concerned, collected during prevention, diagnosis, care or medical or medico-monitoring activities. social or a survey in the field of health.

					II.-On expiry of the period provided for in 4° of IV of Article L. 1461-1 , this data is archived in accordance with Articles L. 212-2 and L. 212-3 of the Heritage Code.<br><br>Article R1461-5<br>Modified by Decree n°2021-848 of June 29, 2021 - art. 1<br><br>I.-Within the limits of their respective responsibilities and their need to know, only have access to the data mentioned in article R. 1461-4 :<br>1° The personnel or service providers of the Health Data Platform acting as data controller, authorized and named by its director due to their missions;<br>2° The staff or service providers of the National Health Insurance Fund acting as data controller, authorized and named by its general director because of their missions.<br>II.-The heads of State services, establishments or organizations mentioned in Article R. 1461-12 authorize, under their responsibility within the structure they manage, or, where applicable within the framework of a subcontracting contract, persons specifically designated to access data from the national health data system.<br>Their number is limited to what is strictly necessary to ensure the execution of authorized data processing. These people must have been specially trained, within the framework of their functions, to carry out their missions.<br>III.-Persons authorized to access data from the national health data system within the framework of the provisions provided for in section 3 of chapter III of title II of law no. 78-17 of January 6, 1978, in application of the provisions of II of article L. 1461-3 , are specifically designated and authorized by the data controller.<br><br>Article R1461-6<br>Modified by Decree n°2021-848 of June 29, 2021 - art. 1<br><br>Each of the State services, public establishments or organizations responsible for a public service mission mentioned in Article R. 1461-12 maintains the list of competent persons within it to issue authorization to access to data from the national health data system, the list of people authorized to access this data, their respective access profiles and the terms of allocation, management and control of authorizations, in accordance with a model established by order of the minister responsible for health.<br><br>These documents are communicated to the National Commission for Information Technology and Liberties, upon its request.<br><br>Article R1461-7<br>Modified by Decree n°2021-848 of June 29, 2021 - art. 1<br><br>I.-To guarantee compliance with the provisions of Article L. 1461-4 , the data present in the national health data system and those made available by the joint managers are linked to each person concerned by a pseudonym. This pseudonym is produced according to the procedures provided for in II of this article.

					II.-The rules for the secure management of the national health data system, defined in a security framework adopted by the ministers responsible for health, social security and digital technology after advice from the National Commission for Informatics and freedoms are established in compliance with the following principles:<br>1° Pseudonymization:<br>a) The databases under the national health data system do not contain any directly identifying data: neither the name nor the first name nor the address nor the registration number in the National Directory for the Identification of Individuals. A pseudonym, consisting of a non-significant code obtained by an irreversible cryptographic process from the registration number in the National Identification Directory of Natural Persons, is associated with the data relating to each person. The provision is made using a different pseudonym for each of the databases made accessible to each of the data controllers;<br>b) The irreversible cryptographic process mentioned above is used to constitute the databases of the national health data system and to match the data extracted from the national health data system and data relating to insurance beneficiaries disease appearing in other systems. This procedure is organized so that no one can have both the identity of the persons, in particular their registration number in the National Identification Directory of Natural Persons, on the one hand, and the pseudonym mentioned in I of this article, on the other hand. The people involved in this procedure are bound by professional secrecy;<br>2° Traceability:<br>The terms of storage and use of data make it possible to control its use and provide proof in the event of unauthorized use.<br><br>Article R1461-8 Modified by Decree n°2021-848 of June 29, 2021 - art. 1<br><br>The identification numbers of health professionals are kept and managed by the Health Data Platform and the National Health Insurance Fund, within the national health data system in tables separate from those in which the others appear. data from the national health data system, in accordance with the provisions of article L. 1461-4 .<br><br>Article R1461-9<br>Modified by Decree n°2021-848 of June 29, 2021 - art. 1<br><br>I.-The Health Data Platform, in accordance with 2° of Article L. 1462-1 , makes the following information available on its website:<br>1° The identity and contact details of the data controllers of the national system health data as well as information relating to the databases that feed it;<br><br>2° The terms and conditions for exercising the rights provided for in II and III of this article;<br><br>3° The list and characteristics of projects relating to data from the national health data system, in particular those mentioned in article R. 1461-17 ;<br><br>4° The information provided for in II of article L. 1461-3 .

					The National Health Insurance Fund produces individual information, by paper or electronic means, relating to the implementation of the national health data system making it possible to bring the main characteristics of this system directly to the attention of people. It makes this information available on its site which links to that of the Health Data Platform.<br><br>The persons concerned are informed by the organizations responsible for the databases that their data feeds into the national health data system.<br><br>II.-The rights of access and rectification are exercised under the conditions defined in articles 15 and 16 of regulation (EU) 2016/679 of April 27, 2016.<br><br>The right of opposition, provided for in article 21 of the regulation (EU) 2016/679 of April 27, 2016 and article 74 of law no. 78-17 of January 6, 1978, concerns the processing data mentioned in 1° of I of article L. 1461-3. It does not apply:<br><br>1° As part of the creation of the national health data system, to the data from the main database mentioned in I of Article R. 1461-2;<br><br>2° As part of the provision of data, to processing implemented by State services, establishments and organizations listed in Article R. 1461-12 in the context of their access permanent.<br><br>When their data relates to databases registered in the catalogue, the persons concerned may exercise a right of erasure, under the conditions provided for in Article 17 of Regulation (EU) 2016/679 of April 27, 2016.<br><br>III.-For the exercise of the rights mentioned in II, the person concerned sends their request, providing proof of their identity by any means, to the director of the Platform or to the director of the compulsory health insurance management body to which they belong.<br><br>IV.-The joint data controllers ensure that they respond to requests made by data subjects who exercise their rights, in accordance with II, in a consistent manner for all data in the national health data system, regardless of who is responsible. spouse who makes them available.<br><br>They set up a circuit in accordance with the provisions of article R. 1461-7 and according to a procedure guaranteeing that no one can have both the identity of the persons, in particular their registration number in the National Directory of identification of natural persons and the pseudonym provided for in I of the same article.<br>Strictly within the limits of what is necessary for the exercise of the rights mentioned in II and according to appropriate technical and organizational protection measures, the Health Data Platform or its subcontractor is authorized to process appropriate personal data. and relevant, including the registration number of individuals in the National Identification Directory of Natural Persons. These provisions do not prejudice the

					powers of the National Health Insurance Fund to ensure the pseudonymization of the data of the person concerned.<br>Releases<br>Related links<br><br>Article R1461-10 Modified by Decree n°2021-848 of June 29, 2021 - art. 1<br><br>The Minister responsible for Health sets the general guidelines for the development of the national health data system. To this end, it brings together a strategic committee bringing together the Health Data Platform and the National Health Insurance Fund.<br><br>An order from the Minister responsible for health establishes the composition, missions and operating procedures of this committee.

France			Arrêté du 30 août 2018 portant organisation de l'agence de l'innovation de défense	Order of August 30, 2018 organizing the agency innovation defense	Article 1<br>Modified by Order of December 30, 2019 - art. 30<br>The defense innovation agency is headed by a director, appointed on the recommendation of the general delegate for armaments.<br>The director may represent the Minister of Defense in international and European cooperation bodies in matters of defense research and technology.<br><br>Article 3<br>Modified by Order of December 30, 2019 - art. 30<br>In terms of defense strategy and technologies, the Defense Innovation Agency is responsible for:<br><br>1° To guide the work carried out in the field of innovation within the ministry. It coordinates the action of staffs, directorates and services. It ensures the overall consistency of the innovation systems implemented and their compliance with the objectives defined by the Minister of Defense. In this context, it contributes to the expertise of scientific and technical projects carried out within the ministry. It proposes the establishment of the necessary partnerships with research organizations and other innovation stakeholders outside the ministry;<br><br>2° To develop synergies and propose the establishment of cooperation with foreign and international public and private organizations working in the field of innovation;<br><br>3° To prepare the acts relating to the supervision of the national office for aerospace studies and research, the Franco-German research institute of Saint-Louis and the national center for space studies. It monitors the public service missions carried out by these establishments and the Atomic Energy and Alternative Energies Commission. It verifies the adequacy of the use of allocated resources with regard to these missions and the strategic orientations of the ministry. It ensures that ministerial objectives are taken into account in research activities carried out in schools and public scientific, cultural and professional establishments reporting to the Minister of Defense.
France			Code de la consommation	Consumer Code	Article L411-1<br><br>Creation Ordinance n°2016-301 of March 14, 2016 - art.<br><br>From the first time they are placed on the market, products and services must meet current requirements relating to personal safety and health, fair commercial transactions and consumer protection.<br>The person responsible for the first placing on the market of a product or service verifies that it complies with the regulations in force.<br>At the request of authorized agents, it justifies the verifications and controls carried out.

France	2023		Décret n° 2023-1083 du 23 novembre 2023 portant création de l'office anti-cybercriminalité	Decree No. 2023-1083 of November 23, 2023 creating the anti-cybercrime office	Article 1<br>An anti-cybercrime office (OFAC) is created, attached to the general directorate of the national police (national directorate of the judicial police) under the Ministry of the Interior.<br>The general directorate of the national gendarmerie, the general directorate of internal security, the general directorate of customs and indirect rights, the general directorate of competition, consumption and the repression of fraud and the ministry of justice are associated with the activities of this office.<br><br>Article 3<br>The office is responsible for:<br>1° Leading and coordinating, at the national level, and at the operational level, the fight against the perpetrators and accomplices of offenses specific to crime linked to information and communication technologies in its scope of jurisdiction;<br>2° To carry out judicial investigations into cybercrime under the authority of the public prosecutor or the investigating judge;<br>3° To carry out, at the request of the judicial authority, all acts of investigation and technical work of digital investigations in assistance to the services responsible for judicial police investigations into offenses whose commission is facilitated by or linked to the use of information and communication technologies, without prejudice to the competence of other central judicial police offices and State services responsible for providing technical assistance to judicial activity;<br>4° To provide assistance to the services of the national police, the national gendarmerie, the general directorate of customs and indirect duties, the general directorate of competition, consumption and the repression of fraud and any other service , in the event of offenses referred to in the second and third paragraphs of Article 2 of this decree, when they request it. This assistance does not take over from the requesting services;<br>5° To intervene on its own initiative, with the agreement of the judicial authority, whenever circumstances require it, to obtain information on the spot about the facts relating to the investigations carried out;<br>6° To participate, in their area of expertise, in training activities;<br>7° To collect and analyze criminal intelligence in its area of competence and to contribute to the production of threat reports induced by cybercrime.
France			INSTRUCTION N° 1221/DEF/EMA/CPI relative aux missions, à l'organisation et au fonctionnement du commandement des programmes interarmées et de la cyberprotection.	INSTRUCTION No. 1221/DEF/EMA/CPI relating to the missions, organization and operation of the command of joint programs and cyber protection.	The purpose of this instruction is to specify the missions of the Joint Programs and Cyber Protection Command (CPIC) as well as its organization and operation.<br>1. CREATION.<br>The CPIC was created as a joint agency (OIA) by decision reference g).<br>2. MISSIONS.<br>The CPIC is responsible for assisting the Chief of Staff of the Armed Forces (CEMA) in the implementation of joint capabilities, approval of joint and multinational information systems, cryptology, and operational interoperability of security systems. operational information and communication (SIOC)

France			Décret n° 2017-428 du 28 mars 2017 relatif à la confidentialité des correspondances électroniques privées	Decree No. 2017-428 of March 28, 2017 relating to the confidentiality private electronic correspondence	change to post and electronic communications code<br>Article L32-3<br>Version in force since March 31, 2017<br><br>I. - Operators, as well as members of their staff, are required to respect the secrecy of correspondence. Secrecy covers the content of the correspondence, the identity of the correspondents as well as, where applicable, the title of the message and the documents attached to the correspondence.<br><br>II. - Providers of online public communication services allowing their users to exchange correspondence, as well as members of their staff, respect their confidentiality. Secrecy covers the content of the correspondence, the identity of the correspondents as well as, where applicable, the title of the message and the documents attached to the correspondence.<br><br>III. - I and II of this article do not prevent automated analysis processing, for the purposes of display, sorting or routing of correspondence, or detection of unsolicited content or malicious computer programs, of the content online correspondence, the identity of the correspondents as well as, where applicable, the title or attached documents mentioned in the same I and II.<br><br>IV. - Automated analysis processing, for advertising, statistical purposes or to improve the service provided to the user, of the content of online correspondence, the identity of the correspondents as well as, where applicable, the title or attached documents mentioned in said I and II is prohibited, unless the express consent of the user is obtained at a frequency fixed by regulation, which cannot be more than one year. Consent is specific to each treatment.<br><br>V. - The operators and persons mentioned in I and II are required to bring to the attention of their staff the obligations resulting from this article.<br><br>VI. - This article is applicable in the Wallis and Futuna Islands.
France			LOI n° 2023-451 du 9 juin 2023 visant à encadrer l'influence commerciale et à lutter contre les dérives des influenceurs sur les réseaux sociaux (1)	LAW no. 2023-451 of June 9, 2023 aimed at regulating commercial influence and combating the excesses of influencers on social networks	Article 5<br>I. - The promotion of goods, services or any cause carried out by the persons mentioned in article 1 must be explicitly indicated by the mention “Advertising” or the mention “Commercial collaboration”. This notice is clear,<br>readable and identifiable on the image or video, in all formats, during the entire promotion. The absence of indication of the true commercial intention of a communication, carried out under the conditions provided for in the first paragraph of this I by the persons mentioned in Article 1 of this law, constitutes a misleading commercial practice by omission within the meaning of article L. 121-3 of the Consumer Code. Violation of the provisions set out in this I is punishable by two years' imprisonment and a fine of 300,000 euros, under the conditions provided for in articles L. 132-1 to L. 132-9 of the Consumer Code.

France	2018		Décret n° 2018-856 du 8 octobre 2018 portant création de l'Agence du numérique de la sécurité civile	Decree No. 2018-856 of October 8, 2018 creating the Digital Agency of thesecuritycivil	Section 2<br><br>The digital civil security agency, as it is created in article R. 732-11-1 of the internal security code, replaces the State in its rights and obligations with regard to matters relating to the accomplishment of the missions provided for in 1°, 2° and 5° of article R. 732-11-2 of the same code undertaken by the ministry in charge of civil security, in particular in respect of affected goods, property intellectual property of current productions and contracts.<br>The list of these goods, contracts and productions is established by joint order of the minister in charge of civil security and the minister in charge of the budget.
France		2018	LOI n° 2018-1202 du 22 décembre 2018 relative à la lutte contre la manipulation de l'information (1)	LAW no. 2018-1202 of December 22, 2018 relating to the fight against the manipulation of information (1)	Title III: DUTY OF COOPERATION OF ONLINE PLATFORM OPERATORS IN THE FIGHT AGAINST THE DISTRIBUTION OF FALSE INFORMATION (Articles 11 to 15)<br>Section 11<br>Modified by LAW n°2021-1382 of October 25, 2021 - art. 33<br>I. - The online platform operators mentioned in the first paragraph of Article L. 163-1 of the electoral code implement measures to combat the dissemination of false information likely to disturb public order or alter the sincerity of one of the votes mentioned in the first paragraph of article 33-1-1 of law no. 86-1067 of September 30, 1986 relating to freedom of communication.<br>They set up an easily accessible and visible system allowing their users to report such information, particularly when it comes from content promoted on behalf of a third party.<br>They also implement additional measures which may include:<br>1° The transparency of their algorithms;<br>2° The promotion of content from companies and press agencies and audiovisual communication services;<br>3° The fight against accounts massively spreading false information;<br>4° Information for users on the identity of the natural person or the company name, the registered office and the corporate purpose of the legal entities paying them remuneration in return for the promotion of information content relating to a debate of general interest;<br>5° Information for users on the nature, origin and methods of dissemination of content;<br>6° Media and information education.<br>These measures, as well as the resources they devote to them, are made public. Each operator sends a declaration each year to the Audiovisual and Digital Communication Regulatory Authority specifying the terms of implementation of said measures.

France			Décret n° 2019-206 du 20 mars 2019 relatif à la gouvernance de la politique de sécurité économique	Decree No. 2019-206 of March 20, 2019 relating to the governance of economic security policy	Article 1<br>Modified by Decree No. 2020-1270 of October 19, 2020 - art. 1<br>I. - The economic security policy aims to ensure the defense and promotion of the economic, industrial and scientific interests of the Nation, consisting in particular of strategic material and intangible assets for the French economy. It includes the defense of digital sovereignty.<br><br>Article 3<br>I. - The nationally competent service called “strategic information and economic security service” is attached to the general director of companies. It is notably responsible, in consultation with the ministries concerned:<br>1° As part of its strategic information missions:<br>- to identify the sectors, technologies and entities relating to the economic, industrial and scientific interests of the Nation, to gather strategic information concerning them with the assistance of the ministries concerned, to ensure their synthesis and to promote their capitalization and sharing for the benefit of these same ministries;<br>- to inform State authorities about persons, entities, compliance standards, and any regulations, including those of extraterritorial scope, as well as business practices, representing a threat to the aforementioned interests and to propose , if necessary, the measures to remedy it;<br><br>2° As part of its missions in terms of economic security:<br>- to raise awareness among economic actors of the issues of economic security;<br>- to contribute to the detection and identification of foreign investment operations likely to fall under the prior authorization procedure defined in I of Article L. 151-3 of the Monetary and Financial Code , before their implementation, or when these operations were carried out without having been previously authorized by the minister responsible for the economy;<br>- to coordinate the monitoring by the ministerial departments concerned or entities attached to them of the commitments made by companies as part of the authorization procedure mentioned in the previous paragraph;<br>- to ensure the application of the provisions of Law No. 68-678 of July 26, 1968 mentioned above , by the persons subject to it, subject to the powers attributed by law in this matter to another authority and, where appropriate where applicable, in connection with it;<br>- to formulate any proposals intended to strengthen the effectiveness of public policy tools contributing to the defense of the economic, industrial and scientific interests of the Nation;<br><br>3° For the promotion of the economic, industrial and scientific interests of the Nation:<br>- to identify actions likely to contribute to the promotion of the economic, industrial and scientific interests of the Nation;<br>- to support, as necessary, the State services responsible for implementing these actions;<br>- to contribute to the dissemination to economic players of strategic information useful in the context of their international development, in conjunction with the ministries concerned.

					II. - The service leads a network of regional delegates for strategic information and economic security responsible for coordinating the implementation, under the authority of regional prefects, of the economic security policy, to which all of the administrations concerned.
Germany			Vertrauensdienstegesetz (VDG)	Trust Services Act (VDG)	§ 2 Supervisory body; responsible body for information security<br><br>(3) The Federal Office for Information Security is the national body responsible for information security within the meaning of Article 19 paragraph 2 of Regulation (EU) No. 910/2014..

Germany	2024		Gesetz zur Beschleunigung der Digitalisierung des Gesundheitswesens	Law to accelerate the digitalization of the healthcare system	Change to SGB V<br>After Section 318, the following Sections 318a and 318b are inserted:<br>“§ 318a Digital Advisory Board of the Society for Telematics<br>(1) The Telematics Society must set up a digital advisory board by ... [insert: date of the last day of the third calendar month following the announcement]. The Digital Advisory Board includes the Federal Office for Information Security and the Federal Commissioner for Data Protection and Freedom of Information. The shareholders' meeting of the Telematics Society can appoint additional members. When appointing the digital advisory board, medical and ethical perspectives must be taken into account in particular.<br>(2) The Digital Advisory Board has its own rules of procedure, which require approval by the shareholders' meeting of the Telematics Society.<br>(3) The Digital Advisory Board continuously advises the Telematics Society on matters of data protection and data security as well as the user-friendliness of the telematics infrastructure and its applications. It is before the resolution of the shareholders' meeting of the company for telematics on the matter.<br>to hear the statements according to sentence 1. Section 318 paragraph 2 sentence 2 and paragraphs 3, 5 and 6 apply accordingly.<br><br>“§ 337 Right of the insured to process data and to grant or deny access rights to data”.<br><br>Section 342 is amended as follows:<br>a) Paragraph 1 is worded as follows: (1) “The health insurance companies are obliged until January 14, 2025 to provide every insured person, upon request and with their consent, with an electronic patient file approved by the Gesellschaft für Telematik in accordance with Section 325 paragraph 1 which meets the requirements of paragraph 2 number 1 letters a to f and n to r. From January 15, 2025, the health insurance companies are obliged to provide every insured person who, after prior information in accordance with Section 343, has not objected to the health insurance company setting up an electronic patient file within a period of six weeks, a certificate from the Gesellschaft für Telematik in accordance with Section 325 paragraph 1 to provide approved electronic patient files that meet the requirements in accordance with paragraph 2 number 1 letters a, b, g to r, numbers 3, 6 and 7 as well as paragraph 2a in a timely manner.<br><br>“§ 344 Objection by the insured and admissibility of data processing by the health insurance companies and the providers of electronic patient files”.<br><br>“§ 351 Transfer of data from applications according to § 33a into the electronic patient file; Provision of electronic patient file data in cross-border exchange”.<br>b) Paragraphs 1 and 2 are replaced by the following paragraph 1:<br>(1) “The health insurance company must ensure within the period to be determined by way of the statutory ordinance in accordance with Section 342 paragraph 2b that<br>1. data of the insured person in digital health applications in accordance with Section 33a can, with the consent of the insured person, be transmitted by the manufacturer of a digital health application in accordance with Section 33a via the provider of the electronic patient file into the electronic patient file of the insured person in accordance with Section 341 paragraph 2 number 9 and stored there,

					2. Data from the electronic patient file can be processed in digital health applications by the manufacturer of a digital health application with the consent of the insured person and<br>3. Data from the electronic patient record in accordance with Section 341 paragraph 2 number 1 letter c with the consent of the insured person to support specific treatment of the insured person in another member state of the European Union by the respective national eHealth contact point in accordance with Section 359 paragraph 4 via the electronic provider Patient files can be processed.<br><br>“§ 355 Specifications for the semantic and syntactic interoperability of data in the electronic patient file”.<br>b) Paragraphs 1 and 2 are worded as follows:<br>(1) “The National Association of Statutory Health Insurance Physicians shall, in agreement with the Competence Center for Interoperability, make the necessary specifications and specifications for the content and updating of the content of the electronic patient file for the use and use of the content in order to ensure their semantic and syntactic interoperability in health care and in behavior with
Germany	2024		Gesetzes zur verbesserten Nutzung von Gesundheitsdaten (Gesundheitsdatennutzungsgesetz – GDNG)	Act for the Improved Use of Health Data (Health Data Usage Act – GDNG)	§ 1 Purpose of the law; scope of application<br>(1) This law serves to regulate the use of health data for public interest-oriented research purposes and for the data-based further development of the healthcare system as a learning system. The aim of using health data is to ensure safer, better and quality-assured healthcare and care, to promote research and innovation and to further develop the digitized healthcare system based on a solid database.<br>(2) This law applies to the processing of health data for research purposes, to improve health care and nursing care and for other purposes of public interest.<br>(3) The provisions of this Act take precedence over those of the Fifth and Eleventh Books of the Social Code to the extent that health data is processed for scientific research purposes and for other purposes in the public interest mentioned in this Act.

Germany	1995	2021	Gesetz über Urheberrecht und verwandte Schutzrechte (Urheberrechtsgesetz)	Copyright and Related Rights Act (Copyright Act)	§ 2 Protected works<br>(1) Protected works of literature, science and art include in particular:<br>1. Language works, such as written works, speeches and computer programs;<br>2. works of music;<br>3. pantomime works including works of dance art;<br>4. Works of the visual arts, including works of architecture and applied art, and designs for such works;<br>5.Photographic works, including works created similarly to photographic works;<br>6.Cinematographic works, including works created similar to cinematographic works;<br>7.Representations of a scientific or technical nature, such as drawings, plans, maps, sketches, tables and plastic representations.<br>(2) Works within the meaning of this law are only personal intellectual creations.<br><br>§ 44b Text and data mining<br>(1) Text and data mining is the automated analysis of individual or multiple digital or digitized works in order to obtain<br>information, particularly about patterns, trends and correlations.<br>(2) Reproductions of legally accessible works for text and data mining are permitted. The reproductions must be deleted when they are no longer required for text and data mining.<br>(3) Uses in accordance with paragraph 2 sentence 1 are only permitted if the right holder has not reserved the right to do so. A reservation of use for works accessible online is only effective if it is in machine-readable form.

Germany	2017		Gesetz zur Anpassung des Datenschutzrechts an die Verordnung (EU) 2016/679 und zur Umsetzung der Richtlinie (EU) 2016/680 (Datenschutz-Anpassungs- und -Umsetzungsgesetz EU – DSAnpUG-EU)	Law to adapt data protection law to Regulation (EU) 2016/679 and to implement Directive (EU) 2016/680 (Data Protection Adaptation and Implementation Act EU – DSAnpUG-EU)	Imlementation of the Federal Data Protection Act (Bundesdatenschutzgesetz)<br>§ 1 Scope of the law<br>(1) This law applies to the processing of personal data by<br>1. federal public bodies,<br>2. public bodies of the federal states, insofar as data protection is not regulated by state law and insofar as they<br>a) Execute federal law or<br>b) act as organs of the administration of justice and do not involve administrative matters.<br>For non-public bodies, this law applies to the fully or partially automated processing of personal data as well as the non-automated processing of personal data contained in a<br>file system are stored or are to be stored, unless the processing is carried out by natural persons for the exercise of exclusively personal or family activities.<br>(2) Other federal legal provisions on data protection take precedence over the provisions of this Act. If they do not regulate a matter to which this law applies, or do not regulate it exhaustively, the provisions of this law apply. The obligation to maintain legal confidentiality obligations or professional or special official secrets that are not based on legal regulations remains unaffected<br><br>Chapter 2<br>Legal basis for processing personal data<br>§ 48 Processing of special categories of personal data<br>(1) The processing of special categories of personal data is only permitted if it is absolutely necessary to fulfill the task.<br>(2) If special categories of personal data are processed, appropriate safeguards must be provided for the legal interests of the data subjects.<br>Suitable guarantees can be in particular<br>1. specific requirements for data security or data protection control,<br>2. the determination of special separation test periods,<br>3. raising awareness among those involved in processing operations,<br>4. restricting access to personal data within the responsible body,<br>5. processing separate from other data,<br>6. the pseudonymization of personal data,<br>7. the encryption of personal data or<br>8. specific procedural rules that ensure the lawfulness of the processing in the case of transfer or processing for other purposes.<br>The information provided to the data subject in accordance with Article 14 paragraph 1, 2 and 4 of Regulation (EU) 2016/679 is in addition to that in Article 14 paragraph 5 of Regulation (EU) 2016/679 and that in Section 29 paragraph 1 sentence 1 mentioned exception not if the provision of the information<br>1. in the case of a public body<br>a) would jeopardize the proper performance of the tasks within the responsibility of the controller within the meaning of Article 23 paragraph 1 letters a to e of Regulation (EU) 2016/679 or

					b) would endanger public safety or order or would otherwise cause disadvantages to the welfare of the federal government or a state and therefore the interest of the person concerned in providing information must be withdrawn,<br>2. in the case of a non-public body<br>a) would impair the assertion, exercise or defense of civil law claims or the processing involves data from civil law contracts and serves to prevent damage caused by criminal offenses, unless the legitimate interest of the data subject in providing the information outweighs it, or<br>b) the responsible public body has determined to the person responsible that disclosure of the data would endanger public safety or order or would otherwise harm the welfare of the federal government or a state; In the case of data processing for criminal prosecution purposes, no statement according to the first half of the sentence is required.<br>2) If the data subject is not informed in accordance with paragraph 1, the controller shall take appropriate measures to protect the legitimate interests of the data subject, including providing the information referred to in Article 14(1).<br>and 2 of Regulation (EU) 2016/679 to the public in a precise, transparent, understandable and easily accessible form in clear and simple language. The person responsible records in writing the reasons for which he or she is informed<br>information apart.<br>(3) Does the provision of information refer to the transmission of personal data by public bodies to the constitutional protection authorities, the Federal Intelligence Service, the Military Counterintelligence Service and, to the extent that federal security is affected<br>other authorities of the Federal Ministry of Defense, it is only permitted with the consent of these authorities<br><br>§ 35 Right to deletion<br>(1) If deletion in the case of non-automated data processing is not possible or only possible with disproportionate effort due to the special type of storage and the interest of the data subject in deletion is considered to be low, then there is<br>the data subject's right to and the controller's obligation to delete personal data in accordance with Article 17(1) of the Regulation<br>(EU) 2016/679 in addition to the exceptions mentioned in Article 17(3) of Regulation (EU) 2016/679. In this case, deletion is replaced by restriction of processing in accordance with Article 18<br>of Regulation (EU) 2016/679. Sentences 1 and 2 do not apply if the personal data was processed unlawfully.<br>(2) In addition to Article 18 paragraph 1 letters b and c of Regulation (EU) 2016/679, paragraph 1 sentences 1 and 2 apply accordingly in the case of Article 17 paragraph 1 letters a and d of Regulation (EU) 2016/679, as long as and to the extent that the person responsible has reason to do so<br>It is assumed that deletion would impair the data subject's legitimate interests. The controller informs the data subject of the restriction of processing, unless informing them proves impossible or would require disproportionate effort.<br>(3) In addition to Article 17 paragraph 3 letter b of Regulation (EU) 2016/679, paragraph 1 applies accordingly in the case of Article 17 paragraph 1 letter a of Regulation (EU) 2016/679 if deletion is subject to statutory or contractual retention periods oppose

					Chapter 3<br>Obligations of those responsible and processors<br>§ 38 Data protection officers of non-public bodies<br>(1) In addition to Article 37 paragraph 1 letters b and c of Regulation (EU) 2016/679, the controller and the processor appoint a data protection officer, provided that they usually have at least ten people<br>constantly engage in the automated processing of personal data. If the controller or processor carries out processing operations that are subject to a data protection impact assessment in accordance with Article 35 of Regulation (EU) 2016/679,<br>or if they process personal data for commercial purposes for the purpose of transmission, anonymized transmission or for market or opinion research purposes, they have a data protection officer, regardless of the number of people involved in the processing<br>n if the processing is necessary for these purposes and the interests of the controller in the processing significantly outweigh the interests of the data subject in excluding the processing. The person responsible shall provide appropriate and specific measures to safeguard the interests of the data subject in accordance with Section 22 Paragraph 2 Sentence 2.<br>(2) The rights of the data subject provided for in Articles 15, 16, 18 and 21 of Regulation (EU) 2016/679 are limited to the extent that these rights are likely to make the achievement of the research or statistical purposes impossible or serious<br>and the restriction is necessary to fulfill the research or statistical purposes.<br>Furthermore, the right to information pursuant to Article 15 of Regulation (EU) 2016/679 does not exist if the data is necessary for the purposes of scientific research and providing the information would require disproportionate effort.<br>(3) In addition to the measures mentioned in Section 22 paragraph 2, special categories of personal data processed for scientific or historical research purposes or for statistical purposes within the meaning of Article 9 paragraph 1 of Regulation (EU) 2016/679 must be anonymized as soon as this is done after Research or statistical purposes are possible unless the legitimate interests of the data subject conflict with this. Until then, the characteristics must be stored separately with which individual information about personal or factual circumstances can be assigned to a specific or identifiable person. They may only be combined with the individual information if the research or statistical purpose requires this.<br>(4) The person responsible may only publish personal data if the person concerned has consented or if this is essential for the presentation of research results about events in contemporary history.<br><br>Chapter 2<br>Rights of the data subject<br>§ 32 Obligation to provide information when collecting personal data from the data subject<br>(1) The obligation to inform the data subject in accordance with Article 13 paragraph 3 of Regulation (EU) 2016/679 does not apply in addition to the exception mentioned in Article 13 paragraph 4 of Regulation (EU) 2016/679 if the provision of the information<br>about the intended further processing

					1. concerns further processing of analogously stored data, in which the person responsible directly addresses the data subject through further processing, the purpose being the same as the original purpose of collection in accordance with the Regulation (EU)<br>2016/679 is compatible, communication with the data subject does not take place in digital form and the data subject's interest in providing information is considered to be low depending on the circumstances of the individual case, in particular with regard to the context in which the data was collected ,<br>2. in the case of a public body, the proper performance of the tasks within the responsibility of the controller within the meaning of Article 23 paragraph 1 letters a to e of Regulation (EU) 2016/679 would be jeopardized and the interests of the controller in not providing the information interests of the person concerned outweigh<br>3. would endanger public safety or order or otherwise harm the welfare of the federal government or a state and the interests of the person responsible in not providing the information outweigh the interests of the person concerned,<br>4. would impair the assertion, exercise or defense of legal claims and the interests of the person responsible in not providing the information outweigh the interests of the data subject or<br>5. would endanger the confidential transmission of data to public authorities.<br>(2) If the data subject is not informed in accordance with paragraph 1, the controller shall take appropriate measures to protect the legitimate interests of the data subject, including providing the information referred to in Article 13 paragraph 1<br>and 2 of Regulation (EU) 2016/679 to the public in a precise, transparent, understandable and easily accessible form in clear and simple language. The person responsible records in writing the reasons for which he or she is informed<br>information apart. Sentences 1 and 2 do not apply in the cases of paragraph 1 numbers 4 and 5.<br>(3) If notification is not provided in the cases referred to in paragraph 1 due to a temporary impediment, the person responsible for the obligation to provide information shall comply with the information obligation within a reasonable time, taking into account the specific circumstances of the processing.<br>This deadline after the impediment no longer applies, but at the latest within two weeks.<br><br>§ 33 Obligation to provide information if the personal data was not collected from the data subject<br>(1) The obligation e.g<br>(3) The provisions of this Act take precedence over those of the Administrative Procedure Act to the extent that personal data is processed when determining the facts of the case.<br>(4) This law applies to public bodies. It applies to non-public bodies, provided that:<br>1. the controller or processor processes personal data domestically,<br>2. the processing of personal data takes place within the scope of the activities of a domestic branch of the controller or processor or<br>3. the controller or processor does not have an establishment in a Member State<br>European Union or in another contracting state to the Agreement on the European Economic Area, but it falls within the scope of application of Regulation (EU) 2016/679 of the Eurozone<br>European Parliament and of the Council of 27 April 2016 on the protection of natural persons with regard to the processing of personal data, on the free movement of such data and repealing the Directive<br>95/46/EC (General Data Protection Regulation) (OJ L 119, 4.5.2016, p. 1; L 314, 22.11.2016, p. 72).

					If this law does not apply in accordance with sentence 2, the responsible person or contracting authority shall apply<br>Processors only §§ 8 to 21, 39 to 44.<br>(5) The provisions of this law do not apply to the extent that the law of the European Union, in particular Regulation (EU) 2016/679 in the currently applicable version, applies directly.<br>(6) For processing purposes pursuant to Article 2 of Regulation (EU) 2016/679, the contracting states to the Agreement on the European Economic Area and Switzerland are equivalent to the member states of the European Union. In this respect, other states are considered third countries.<br>(7) When processing for the purposes referred to in Article 1(1) of Directive (EU) 2016/680 of the European Parliament and of the Council of 27 April 2016 on the protection of natural persons with regard to the processing of personal data by competent authorities for the purposes of prevention, investigation , detection or prosecution of criminal offenses or the execution of sentences, as well as the free movement of data and the repeal of Council Framework Decision 2008/977/JHA (OJ L 119, 4.5.2016, p. 89).<br>States associated with the implementation, application and development of the Schengen acquis are equal to the Member States of the European Union. In this respect, other states are considered third countries.<br>(8) For processing of personal data by public bodies in the context of activities not falling within the scope of Regulation (EU) 2016/679 and Directive (EU) 2016/680<br>Regulation (EU) 2016/679 and parts 1 and 2 of this law apply accordingly, unless otherwise provided for in this law or another law.<br><br>Chapter 3<br>Data protection officers of public bodies<br>§ 5 Designation<br>(1) Public bodies appoint a data protection officer or a data protection officer. This also applies to public bodies in accordance with Section 2 Paragraph 5 that take part in the competition.<br><br>Chapter 4<br>The Federal Commissioner for Data Protection and Freedom of Information<br>§ 8 Construction<br>(1) The Federal Commissioner for Data Protection and Freedom of Information (Federal Commissioner) is a supreme federal authority. The office is in Bonn.<br><br>Part 1<br>PROCESSING SPECIFIC TO THE CATEGORIES IN REFERENCE TO THE DATA AND PROCESSING TO THE OTHERS e c k e n<br>§ 22<br>Processing of special categories of personal data<br>(1) By way of derogation from Article 9(1) of Regulation (EU) 2016/679, the processing of special categories of personal data is within the meaning<br>ne of Article 9(1) of Regulation (EU) 2016/679<br>1. by public and non-public bodies, if they

					a) is necessary to exercise the rights arising from social security and social protection law and to fulfill the obligations in this regard,<br>b) for the purpose of health care, for assessing the employee's ability to work, for medical diagnostics, care or treatment in the health or social sector or for the administration of systems and<br>health and social services or due to a contract between the data subject and a health professional and these data are processed by medical staff or by other persons who are subject to an appropriate obligation of confidentiality or under their responsibility, or<br>c) is necessary for reasons of public interest in the field of public health, such as protection against serious cross-border threats to health or to ensure high standards of quality and safety in healthcare and in medicines and medical devices; In addition to the measures mentioned in paragraph 2, the professional and criminal law requirements for maintaining professional secrecy must be observed,<br>2. by public bodies, if they<br>a) is absolutely necessary for reasons of significant public interest,<br>b) is necessary to avert a significant threat to public safety,<br>c) is absolutely necessary to prevent significant disadvantages for the common good or to protect significant interests of the common good or<br>d) is necessary for compelling reasons of defense or the fulfillment of supra- or intergovernmental obligations of a federal public body in the field of crisis management or conflict prevention or for humanitarian measures<br>and to the extent that the interests of the controller in data processing in the cases of number 2 outweigh the interests of the data subject.<br>(2) In the cases referred to in paragraph 1, appropriate and specific measures must be taken to protect the interests of the data subject. Taking into account the state of the art, the implementation costs and the nature, scope, circumstances<br>and the purposes of the processing as well as the different likelihood and severity of the risks to the rights and freedoms of natural persons associated with the processing may include in particular:<br>1. technical organizational measures to ensure that processing takes place in accordance with Regulation (EU) 2016/679,<br>2. Measures that ensure that it can be subsequently checked and determined whether and by whom personal data has been entered, changed or removed,<br>3. Raising awareness of those involved in processing operations,<br>4. Appointment of a data protection officer,<br>5. Restriction of access to personal data within the responsible body and by processors,<br>6. Pseudonymization of personal data,<br>7. Encryption of personal data,<br>8. Ensuring the ability, confidentiality, integrity, availability and resilience of systems and services related to the processing of personal data, including the ability to quickly restore availability and access in the event of a physical or technical incident,<br>9. to ensure the security of processing, the establishment of a procedure for regular review, assessment and evaluation of the effectiveness of the technical and organizational measures or<br>10. specific procedural regulations that ensure compliance with the requirements of this law and Regulation (EU) 2016/679 in the event of transfer or processing for other purposes.

					§ 24 Processing for other purposes by non-public bodies<br>(1) The processing of personal data by non-public bodies for a purpose other than that for which the data was collected is permitted if<br>1. it is necessary to avert threats to state or public security or to prosecute criminal offenses or<br>2. it is necessary to assert, exercise or defend civil law claims unless the interests of the data subject in excluding processing outweigh them.<br><br>§ 27 Data processing for scientific or historical research purposes and for statistical purposes<br>(1) By way of derogation from Article 9 paragraph 1 of Regulation (EU) 2016/679, the processing of special categories of personal data within the meaning of Article 9 paragraph 1 of Regulation (EU) 2016/679 is also permitted without consent for scientific or historical purposes.<br>technical research purposes or for statistical purposes, we

Germany	2021		Gesetz zur Anpassung des Urheberrechts an die Erfordernisse des digitalen Binnenmarktes	Law to adapt copyright to the requirements of the digital internal market	§3 Uncovered services<br>In particular, this law does not apply to:<br>4. Providers of electronic communications services within the meaning of Article 2 number 4 of Directive (EU) 218/1972 of the European Parliament (CHECK!)<br><br>§4 Obligation to acquire contractual rights of use; Direct remuneration claim of the author<br>(1) A service provider is obliged to make the best possible efforts to acquire the contractual rights of use for the public reproduction of works protected by copyright.

Germany	2019		Gesetz für eine bessere Versorgung durch Digitalisierung und Innovation (Digitale-Versorgung-Gesetz – DVG)	Law for better care through digitalization and innovation (Digital Supply Act – DVG)	article 1<br>Amendment to the Fifth Book of the Social Code The Fifth Book of the Social Code - Statutory Health Insurance - (Article 1 of the law of December 20, 1988, BGBl. I p. 2477, 2482), which was last amended by Article 5 of the law of December 12, 2019 (BGBl. I P. 2522) has been amended, is amended as follows: 1a. § 20h is amended as follows:<br>b) After paragraph 1, the following paragraph 2 is inserted: "(2) The health insurance companies and their associations also take into account, within the framework of the funding in accordance with paragraph 1 sentence 1, those digital applications that meet the requirements for data protection and data security according to the state of the art guarantee."<br><br>"§ 20k Promoting digital health literacy<br>(1) In its statutes, the health insurance company provides services to promote the self-determined, health-oriented use of digital or telemedical applications and procedures by the insured. The services are intended to impart the skills required for the use of digital or telemedical applications and procedures. The health insurance company bases this on the specifications of the central association of health insurance companies in accordance with paragraph 2.<br><br>§ 33a Digital health applications<br>(1) Insured persons are entitled to be supplied with low-risk medical devices whose main function is essentially based on digital technologies and which are intended to detect, monitor, treat or alleviate illnesses or to detect, treat, or treat illnesses in the insured persons or in the care provided by service providers , to support the relief or compensation of injuries or disabilities (digital health applications). The claim only covers those digital health applications that 1. have been included by the Federal Institute for Drugs and Medical Devices in the directory for digital health applications in accordance with Section 139e and 2. are used either according to the prescription of the treating doctor or the treating psychotherapist or with the approval of the health insurance company. For approval in accordance with sentence 2 number 2, proof must be provided of the medical indication for which the digital health application is intended. Select insured medical devices, their functions or areas of application via the digital directory<br>health applications included in Section 139e or whose costs exceed the reimbursement amounts in accordance with Section 134, you must bear the additional costs yourself.<br>(2) Medical devices with a low risk class according to paragraph 1 sentence 1 are those that are in risk class I or IIa according to Article 51 in conjunction with Annex VIII of Regulation (EU) 2017/745 of the European Parliament and of the Council of April 5, 2017 on medical devices , amending Directive 2001/83/EC, Regulation (EC) No 178/2002 and Regulation (EC) No 1223/2009 and repealing Council Directives 90/385/EEC and 93/42/EEC (OJ L 117 of 5.5.2017, p. 1; L 117 of 3.5.2019, p. 9) and have already been placed on the market as such, as a medical device in risk class IIa due to the transitional provisions in Article 120 Paragraph 3 or paragraph 4 of Regulation (EU) 2017/745 were placed on the market or remain marketable and are in circulation as a medical device in risk class I due to Union law regulations.<br>(3) Manufacturers make digital health applications available to insured persons by electronic transmission via publicly accessible networks or on machine-readable data carriers. If a transfer or delivery in accordance with sentence 1 is not possible, digital health applications can also be made available via publicly accessible digital distribution platforms; In these cases, the health insurance company reimburses the insured person for the actual costs up to the amount of the reimbursement amounts in accordance with Section 134.<br>(4) Claims for benefits under other provisions of this book remain unaffected. The entitlement to benefits pursuant to paragraph 1 exists regardless of whether the digital health application is a new examination or treatment method; There is no need for a guideline in accordance with Section 135 Paragraph 1 Sentence 1. A claim to benefits in accordance with Paragraph 1 to digital health applications that contain services [illegible]

German y	2021		Zweites Gesetz zur Erhöhung der Sicherheit informationstechnischer Systeme	Second law to increase the security of information technology systems	Article 1 Amendment to the BSI Act<br>The BSI law of August 14, 2009 (BGBl. IS. 2821), which was last amended by Article 73 of the ordinance of June 19, 2020 (BGBl. I p. 1328), is amended as follows:<br>1. § 1 is worded as follows:<br>“§ 1 Federal Office for Information Security<br>The Federal Office for Information Security (Federal Office) is a higher federal authority within the scope of the Federal Ministry of the Interior, Building and Community. It is the central office for information security at the national level. The Federal Office carries out tasks vis-à-vis the federal ministries on the basis of scientific and technical findings.”<br><br>§ 2 is amended as follows:<br>a) Paragraph 2 is amended as follows: aa) The following sentences are added in front: “Information and information-processing systems, components and processes are particularly worthy of protection. These may only be accessed by authorized persons or programs. Security in information technology and the associated protection of information and information-processing systems from attacks and unauthorized access within the meaning of this law requires compliance with certain security standards to ensure basic information technology values and protection goals.<br><br>After paragraph 9, the following paragraphs 9a and 9b are inserted: “(9a) IT products within the meaning of this law are software, hardware and all individual or interconnected components that process information using information technology. (9b) Attack detection systems within the meaning of this law are processes for detecting attacks on information technology systems supported by technical tools and organizational integration. Attack detection is carried out by comparing the data processed in an information technology system with information and technical patterns that indicate attacks." e) In paragraph 10 sentence 1 number 1, the word "as well as" is replaced by a comma and is used after the word " Insurance” the words “and municipal waste disposal” were added. f) The following paragraphs 13 and 14 are added:<br><br>“(13) Critical components within the meaning of this law are IT products 1. that are used in critical infrastructures, 2. in which disruptions in the availability, integrity, authenticity and confidentiality lead to a failure or a significant impairment of the functionality of critical infrastructures or can lead to threats to public safety and 3. which are a) determined as a critical component on the basis of a law with reference to this regulation or b) implement a function that is determined to be critical on the basis of a law.<br>(14) Companies in the special public interest are companies that are not operators of critical infrastructures in accordance with paragraph 10 and 1. which produce or develop goods in accordance with Section 60 paragraph 1 numbers 1 and 3 of the Foreign Trade Ordinance in the currently applicable version, 2. which in accordance with their are among the largest companies in Germany in terms of domestic value creation and are therefore of considerable economic importance for the Federal Republic of Germany or are of essential importance to such companies as suppliers because of their unique selling points or3. are the operators of an upper class operating area within the meaning of the Major Incident Ordinance in the currently valid version or are treated the same as these in accordance with Section 1 Paragraph 2 of the Major Incident Ordinance. The companies in the special public interest according to sentence 1 number 2 are determined by the legal regulation according to § 10 paragraph 5, which determines which economic

					indicators are decisive for a company being one of the largest companies in Germany within the meaning of number 2 and which unique selling points are key to making suppliers essential for such companies.”<br><br>§ 3 paragraph 1 is amended as follows: a) Sentence 1 is worded as follows: “The Federal Office promotes security in information technology with the aim of ensuring the availability, integrity and confidentiality of information and its processing.”<br><br>§ 4b General Reporting Office for Security in Information Technology (1) In order to carry out the tasks according to § 3, the Federal Office, as the central office for reports from third parties, receives information about security risks in information technology and evaluates this information.<br><br>“§ 7a Investigation of security in information technology<br>(1) In order to fulfill its tasks in accordance with Section 3 Paragraph 1 Sentence 2 Number 1 14, 14a, 17 or 18, the Federal Office may examine information technology products and systems made available on the market or intended to be made available on the market. It can use the support of third parties insofar as the manufacturer's legitimate interests are justified The products and systems affected do not conflict with this.<br>(2) If necessary, the Federal Office of Investigation may request all necessary information from manufacturers of information technology products and systems, in particular regarding technical details, in accordance with paragraph 1. In the request for information, the Federal Office states the legal basis, the purpose of the request for information and the information required and sets a reasonable deadline for the transmission of the information. The request for information also contains a reference to the sanctions provided for in Section 14.<br>(3) The Federal Office shall immediately pass on information and the findings obtained from the investigations to the responsible federal supervisory authorities or, if there is no supervisory authority, to the relevant department if there are indications that they need it to fulfill their tasks.<br>(4) The information and the knowledge gained from the investigations may only be used to fulfill the tasks in accordance with Section 3 Paragraph 1 Sentence 2 Numbers 1, 14, 14a, 17 and 18. The Federal Office may pass on and publish its findings to the extent that this is necessary to fulfill the tasks pursuant to Section 3 Paragraph 1 Sentence 2 Numbers 1, 14, 14a, 17 and 18. The manufacturer of the affected products and systems must first be given the opportunity to comment within a reasonable period of time.<br>(5) If a manufacturer does not comply with the Federal Office's request pursuant to paragraph 2 sentence 1 or only does so inadequately, the Federal Office may inform the public about this. It can state the name of the manufacturer and the name of the product or system concerned and explain to what extent the manufacturer has not fulfilled its obligation to provide information. The manufacturer must first be given the opportunity to comment within a reasonable period of time. Section 7 paragraph 2 sentence 2 applies accordingly.<br><br>“§ 7b Detection of security risks for network and IT security and attack methods<br>(1) As part of its tasks pursuant to Section 3 Paragraph 1 Sentence 2 Numbers 1, 2, 14 or 17, the Federal Office may take measures to detect security gaps and other security risks at federal facilities or the companies mentioned in Section 2 Paragraphs 10, 11 and 14 at the interfaces of publicly accessible

					information technology systems to public telecommunications networks (port scans) if facts justify the assumption that these may be unprotected within the meaning of paragraph 2 and that their security or functionality may therefore be at risk. The measures must be limited to a predetermined range of Internet protocol addresses that are regularly assigned to the information technology systems 1. of the federal government or 2. of critical infrastructures, digital services and companies in the special public interest (white list). The white list must be continually adjusted through appropriate checks to take into account changes in the allocation of Internet protocol addresses to the locations specified in numbers 1 and 2. If the Federal Office receives information that is protected by Article 10 of the Basic Law, it may only process it for the purpose of transmission in accordance with Section 5 Paragraphs 5 and 6. If the requirements of Section 5 Paragraphs 5 and 6 are not met, information that is protected under Article 10 of the Basic Law must be deleted immediately. Measures in accordance with sentence 1 may only be ordered by an employee of the Federal Office who is qualified to hold judicial office. (2) An information technology system is unprotected within the meaning of paragraph 1 if there are publicly known security gaps in it or if the system can be accessed by third parties without authorization due to other obviously inadequate security precautions.<br>(3) If a security gap or other security risk in an information technology system is identified through measures in accordance with paragraph 1, those responsible for the information technology system must be informed immediately. The Federal Office should point out existing remedial options. If the Federal Office does not know who is responsible or if their identification is only possible with disproportionate effort or via an inventory data query in accordance with Section 5c, the operating service provider of the respective network or system must be informed immediately if overriding security interests do not conflict with this. The Federal Office shall inform the Federal Commissioner for Data Protection and Freedom of Information by June 30 of the following year of the number of measures taken in accordance with paragraph 1. The Federal Office submits the white list in accordance with paragraph 1 sentence 3 to the Federal Commissioner or the Federal Commissioner for Data Protection and Freedom of Information for inspection on a quarterly basis.<br>(4) The Federal Office may, in order to fulfill its task Use systems and procedures that simulate a successful attack to an attacker in order to collect and evaluate the use of malware or other attack methods. The Federal Office may process the data necessary to evaluate how the malware and attack methods work.<br><br>“§ 9a National Authority for Cybersecurity Certification<br>(1) The Federal Office is the national authority for cybersecurity certification within the meaning of Article 58 paragraph 1 of Regulation (EU) 2019/881.<br><br>§ 9b Prohibition of the use of critical components<br>(1) The operator of a critical infrastructure must notify the Federal Ministry of the Interior, Building and Community before its use of the planned first-time use of a critical component in accordance with Section 2 Paragraph 13. The notification must indicate the critical component and the planned method of its use. Sentence 1 does not apply to an operator of a critical infrastructure if he has already reported the use of another critical component of the same type for the same type of use in accordance with Sentence 1 and has not been prohibited from doing so.

					(2) The Federal Ministry of the Interior, Building and Community may postpone the planned initial use of a critical component to the operator of the critical infrastructure in consultation with the affected departments listed in Section 10 paragraph 1 and the Federal Foreign Office up to a period of two months Prohibit the receipt of the report pursuant to paragraph 1 or issue orders if the operation is likely to affect public order or security in the Federal Republic of Germany. When examining a likely impairment of public order or security, particular consideration may be given to whether 1. the manufacturer is directly or indirectly controlled by the government, including other government agencies or armed forces, of a third country, 2. the manufacturer has already been involved in activities or which had adverse effects on the public order or security of the Federal Republic of Germany or another member state of the European Union, the European Free Trade Association or the North Atlantic Treaty or on their facilities, or 3. the use of the critical component in accordance with the security policy objectives of the Federal Republic Germany, the European Union or the North Atlantic Treaty.<br><br>§ 9c Voluntary IT security label<br>(1) The Federal Office is introducing a uniform IT security label to inform consumers about the IT security of products in certain product categories defined by the Federal Office. The IT security label makes no statement about the data protection properties of a product.<br>(2) The IT security mark consists of 1. an assurance from the manufacturer or service provider that the product meets certain IT security requirements for a specified period of time (manufacturer's declaration), and 2. information from the Federal Office about security-relevant IT properties of the product (security information).

Germany	2017		Gesetz zur Umsetzung der Richtlinie (EU) 2016/1148 des Europäischen Parlaments und des Rates vom 6. Juli 2016 über Maßnahmen zur Gewährleistung eines hohen gemeinsamen Sicherheitsniveaus von Netz- und Informationssystemen in der Union	Law implementing Directive (EU) 2016/1148 of the European Parliament and of the Council of July 6, 2016 on measures to ensure a high common level of security of network and information systems in the Union	Article 1 Amendment to the BSI Act<br>The BSI law of August 14, 2009 (BGBl. I p. 2821), which was last amended by Article 3 paragraph 6 of the law of July 18, 2016 (BGBl. I p. 1666), is amended as follows: 1 § 2 is amended as follows:<br>a) The following paragraph 11 is inserted after paragraph 10:<br>“(11) Digital services within the meaning of this law are services within the meaning of Article 1 paragraph 1 letter b of Directive (EU) 2015/1535 of the European Parliament and of the Council of September 9, 2015 on an information procedure in the field of technical regulations and the rules on information society services (OJ L 241, 17.9.2015, p. 1), and the<br>1. consumers or traders within the meaning of Article 4 paragraph 1 letter a or letter b of Directive 2013/11/EU of the European Parliament and of the Council of 21 May 2013 on alternative resolution of consumer disputes and amending Regulation (EC) No. 2006/2004 and Directive 2009/22/EC (Directive on alternative dispute resolution in consumer matters) (OJ L 165, 18.6.2013, p. 63) allow sales contracts or service contracts with traders to be concluded either on the website of these services or on an entrepreneur's website that uses computing services provided by these services (online marketplaces);<br>2. Enable users to conduct searches on all websites or on websites in a specific language based on a query on any topic in the form of a keyword, phrase or other input, which then displays links to retrieve content corresponding to the query can be (online search engines);<br>3. provide access to a scalable and elastic pool of shared computing resources (cloud computing services) and are not established or used to protect essential government functions.”<br><br>§ 8c Special requirements for providers of digital services<br>(1) Digital service providers shall take appropriate and proportionate technical and organizational measures to manage risks to the security of the network and information systems they use to provide the digital services within the European Union. They must take measures to prevent or minimize the impact of security incidents on digital services provided within the European Union.<br>(2) Measures to deal with risks to the security of the network and information systems in accordance with paragraph 1 sentence 1 must, taking into account the state of the art, ensure a level of security for the network and information systems that is appropriate to the existing risk. The following aspects must be taken into account:<br>1. the security of the systems and facilities,<br>2. the detection, analysis and containment of security incidents,<br>3. business continuity management,<br>4. monitoring, checking and testing,<br>5. compliance with international standards<br>The necessary measures will be further specified by Commission implementing acts in accordance with Article 16(8) of Directive (EU) 2016/1148.<br>(3) Providers of digital services must immediately report to the Federal Office any security incident that has a significant impact on the provision of a digital service they provide within the European Union. The conditions under which the effects of a security incident are significant are determined in more detail by implementing acts of the Commission in accordance with Article 16(8) of Directive (EU) 2016/1148, taking into account in particular the following parameters: 1. the number of users affected by the security incident, in particular the users who require the service to provide their own services, 2. the duration of the security

					incident, 3. the geographical area affected by the security incident, 4. the extent of the interruption in the provision of the service, 5. the extent of the economic impact and social activities. The obligation to report a security incident does not apply if the provider does not have sufficient access to the information required to assess the impact of a security incident measured against the parameters according to sentence 2. Section 8b paragraph 3 applies accordingly to the content of the reports, unless implementing acts of the Commission pursuant to Article 16 paragraph 9 of Directive (EU) 2016/1148 stipulate otherwise. The Federal Office must inform the competent authority of this member state about security incidents reported in accordance with sentence 1 that have an impact in another member state of the European Union.<br>(4) If there are indications that a provider of digital services meets the requirements of paragraph 1 in conjunction with the implementing regulations If the Commission's acts in accordance with Article 16 paragraph 8 of Directive (EU) 2016/1148 and paragraph 2 in conjunction with the Commission's implementing acts in accordance with Article 16 paragraph 9 of Directive (EU) 2016/1148 are not complied with, the Federal Office may demand that the provider digitally Services require the following measures: 1. the transmission of the information necessary to assess the security of its network and information systems, including evidence of security measures taken, 2. the elimination of deficiencies in the fulfillment of the requirements set out in paragraphs 1 and 2. The evidence may also arise from findings submitted to the Federal Office by the competent authorities of another member state of the European Union. (5) If a provider of digital services has its headquarters, a representative or network and information systems in another member state of the European Union, the Federal Office shall cooperate with the competent authority of this member state in fulfilling the tasks pursuant to paragraph 4. This cooperation may include the request to take the measures in paragraph 4 sentence 1 numbers 1 and 2."<br><br>Article 4<br>Amendment to the Fifth Book of the Social Security Code<br>The Fifth Book of the Social Code - Statutory Health Insurance - (Article 1 of the law of December 20, 1988, BGBl. I p. 2477, 2482), which was last amended by Article 7 of the law of May 23, 2017 (BGBl. I p. 1228). has been amended as follows: 1. The following paragraph 8 is added to Section 291b:<br>“(8) The Telematics Society shall, upon request, submit the following documents and information to the Federal Office for Information Security: 1. the approvals and confirmations in accordance with paragraphs 1a to 1c and 1e, including the underlying documentation, 2. a list of the measures taken in accordance with paragraphs 6 and 7, including the identified security deficiencies and results of the measures, and 3. other information necessary to assess the security of the telematics infrastructure and the approved services and confirmed applications. If the assessment of the information mentioned in sentence 1 reveals security deficiencies, the Federal Office for Information Security can issue binding instructions to the Gesellschaft für Telematik to eliminate the identified security deficiencies. The Telematics Society is authorized to issue binding instructions to operators of approved services and confirmed applications in accordance with paragraphs 1a to 1c and 1e to eliminate identified security deficiencies. The costs of the review are borne by 1. the Telematics Society if the Federal Office for Information Security acted on the basis of evidence that gave rise to legitimate doubts about the security of the telematics infrastructure, 2. the operator of approved services and confirmed applications in accordance with Paragraphs 1a to 1c and 1e, provided that the Federal Office for Information Security acted on the basis of evidence that gave rise to legitimate doubts about the security of the approved services and confirmed applications."

Germany	2021		Gesetz zur Änderung des E-Government-Gesetzes und zur Einführung des Gesetzes für die Nutzung von Daten des öffentlichen Sektors	Act amending the E-Government Act and introducing the Act for the Use of Public Sector Data	Article 2<br>Law governing the use of public sector data<br>(Data Usage Act – DNG)<br>§ 1 Principle of open data<br>(1) Data that falls within the scope of this law should, as far as possible, be created according to the principle of “conceptually and by default open”.<br>(2) This law does not establish an obligation to provide data or a right to access to data<br><br>§ 2 Scope<br>(1) This law applies to data from data providers in accordance with paragraph 2<br>1. are provided based on a legal right to access,<br>2. are provided due to a legal obligation to provide or<br>3. is otherwise made available to the public or for exclusive use.<br>(2) Data providers within the meaning of this law are:<br>1. public bodies;<br>2. Public service companies that are subject to the regulations on the award of public contracts and concessions or that operate public passenger transport services;<br>3. in relation to research data that is publicly funded and has already been made publicly available via an institutional or thematic repository:<br>a) universities, research institutions and research funding institutions,<br>b) Researchers, if the research data has not already been provided by other data providers obliged by this law;<br>4. This does not apply to legitimate business interests, knowledge transfer activities or existing ones intellectual property rights of third parties stand.<br>(3) This law does not apply to 1. data that is not accessible or only accessible to a limited extent, although a restriction also applies if access only exists with proof of a legal or legitimate interest; Data is not accessible or only accessible to a limited extent, in particular, aa) to the extent that this conflicts with the protection of personal data,<br>bb) to the extent that this conflicts with the protection of business secrets,<br>cc) to the extent that the protection of national security, defense or public safety conflicts,<br>dd) insofar as the status as confidential information about the protection of critical infrastructure conflicts or<br>ee) to the extent that statistical confidentiality conflicts,<br>b) which concern the intellectual property of third parties,<br>c) which is accessible in accordance with federal or state regulations regarding public access to environmental information and is unrestricted, free of charge, machine-readable and usable via an application programming interface or<br>d) the provision of which does not fall within the public mandate of the public body established by law;<br>2. Data from public service companies that were created outside of the activities in accordance with Section 3 Number 2;<br>3. Logos, coats of arms and insignia;<br>4. Data from public broadcasters or their agents that serve to carry out the public program or broadcast order;

					5. Data from cultural institutions, except libraries, museums and archives; Paragraph 2 number 3 does not apply to libraries, museums and archives;<br>6. Data from educational institutions at secondary level and below; For all other educational institutions, this law does not apply to data that is not research data.<br>(4) The provisions on the protection of personal data and further requirements for the provision and use of data from data providers from other legal regulations remain unaffected.<br>(5) Public bodies within the scope of this law do not rely on the rights of the database manufacturer in accordance with Section 87b of the Copyright Act.<br><br>§ 4 Principle of unrestricted data use; Admissibility of Licenses<br>(1) Data may be used for any commercial or non-commercial purpose.<br>(2) For data to which libraries, including university libraries, museums and archives, are entitled to copyright or related rights or industrial property rights, and for data of public companies, paragraph 1 only applies to the extent that the institution or public company has permitted its use.<br>(3) Terms of use (licenses) are permitted provided they are objective, proportionate, non-discriminatory and justified by an objective in the general interest. The license must not lead to distortion of competition and must not unnecessarily limit the possibilities of use. Public bodies should use open licenses wherever possible.<br><br>§ 5 Non-discrimination<br>(1) The conditions for data use must be non-discriminatory.<br>(2) If data is used by a public body as raw material for its own business activities that do not fall within the public mandate of the public body, the same fees and other conditions apply to the provision of the data for the business activity as for other users.<br><br>§ 7 Available formats, metadata<br>(1) The data provider must enable the use of the data in all requested and existing formats and languages.<br>(2) As far as possible and sensible, data must be provided electronically and in open, machine-readable, accessible, discoverable and interoperable formats together with the associated metadata in accordance with the recognized rules of technology. Both the formats and the metadata comply with formal open standards wherever possible.<br>(3) Paragraphs 1 and 2 do not oblige public bodies and public companies to create or adapt data and metadata or to make parts of data sets available if this would involve disproportionate effort beyond simple editing. Public bodies and public companies are also not obliged to continue the creation and storage of certain types of data with a view to their use by a private or public sector organization.<br>(4) The metadata for machine-readable data must, where possible and sensible, be made available via the national metadata portal GovData.

Germany	2021		Gesetz zur Regelung des Datenschutzes und des Schutzes der Privatsphäre in der Telekommunikation und bei Telemedien	Law regulating data protection and the protection of privacy in telecommunications and telemedia (Telecommunications Telemedia Data Protection Act - TTDSG)	§1 Scope of the law<br>(1) This law regulates<br>2. special regulations for the protection of personal data when using telecommunications services and telemedia,<br><br>§2 Definitions<br>(2) Within the meaning of this law is or are<br>2. “Inventory data” within the meaning of Part 3 of this law means the personal data whose processing is necessary for the purpose of establishing, structuring the content or changing a contractual relationship between the provider of telemedia and the user regarding the use of telemedia,<br><br>§ 20 Processing of personal data of minors<br>If a telemedia provider has collected personal data from minors to ensure the protection of minors, for example through age verification or other technical measures, or otherwise obtained it, it may not process this data for commercial purposes.<br><br>§ 21 Inventory data<br>(1) Upon order of the responsible authorities, providers of telemedia may provide information about inventory data in individual cases to the extent that this is necessary to enforce intellectual property rights.<br>(2) The provider of telemedia may, in individual cases, also provide information about existing data, insofar as this is necessary for the enforcement of civil law claims due to the violation of absolutely protected rights due to illegal content, which is covered by Section 10a Paragraph 1 of the Telemedia Act or Section 1 Paragraph 3 of the Network Enforcement Act is required. To this extent, he is obliged to provide information to the injured party.<br>(3) In order to provide the information pursuant to paragraph 2, a prior court order on the admissibility of providing the information is required, which must be applied for by the injured party. At the same time, the court decides on the obligation to provide information, unless the application is expressly limited to ordering the admissibility of providing information. The regional court is responsible for issuing this order regardless of the amount in dispute. The court in whose district the injured party has his or her place of residence, headquarters or branch office has local jurisdiction. The decision is made by the civil chamber. The provisions of the Act on Proceedings in Family Matters and in Matters of Voluntary Jurisdiction apply accordingly to the procedure. The injured party bears the costs of the court order. The appeal against the decision of the regional court is admissible.

Germany	2020		Gesetz zum Schutz elektronischer Patientendaten in der Telematikinfrastruktur (Patientendaten-Schutz-Gesetz – PDSG)	Act to protect electronic patient data in the telematics infrastructure (Patient Data Protection Act – PDSG)	Article 1 Amendment to the fifth SGB<br>"§ 68c Promotion of digital innovations by the associations of statutory health insurance physicians and the federal associations of statutory health insurance physicians"<br><br>“§ 75c IT security in hospitals (1) From January 1, 2022, hospitals are obliged to take appropriate organizational and technical precautions based on the state of the art to avoid disruptions to the Availability, integrity and confidentiality as well as the other security objectives of their information technology systems, components or processes that are crucial for the functionality of the respective hospital and the security of the patient information processed. Organizational and technical Precautions are appropriate if the effort required is not disproportionate to the consequences of a failure or impairment of the hospital or the security of the patient information being processed. The information technology systems must be adapted to the current state of the art every two years at the latest.<br>(2) Hospitals can fulfill the obligations under paragraph 1 in particular by applying an industry-specific security standard for the information technology security of health care in hospitals in the currently valid version, the suitability of which has been approved by the Federal Office for Security in the Information technology was determined in accordance with Section 8a Paragraph 2 of the BSI Act.<br>(3) The obligation under paragraph 1 applies to all hospitals, unless they already have to take appropriate technical precautions as operators of critical infrastructures in accordance with Section 8a of the BSI Act."<br><br>"§ 291 Electronic health card<br>(1) The health insurance company issues an electronic health card for every insured person.<br>(2) The electronic health card must be technically suitable to 1. enable barrier-free authentication, encryption and electronic signature, 2. support the applications of the telematics infrastructure in accordance with Section 334 paragraph 1 and<br>3. to enable the storage of data in accordance with Sections 291a and 334 Paragraph 1 Sentence 2 Number 5 in conjunction with Section 358 Paragraph 4.<br>(3) Electronic health cards issued by health insurance companies after November 30, 2019 must be equipped with a contactless card<br>interface. Health insurance companies are obliged to immediately provide insured persons with an electronic health card with a contactless interface upon their request.<br>(6) When issuing the electronic health card, the health insurance company must implement the measures and specifications provided for in the directive in accordance with Section 217f paragraph 4b to protect the social data of the insured against unauthorized access. This includes in particular the exclusion of substitute delivery and deposit to be included in the directive<br>when using the postal delivery order with postal delivery certificate. For the purpose of comparing the insured person's address with the data from the population register, which is provided for in the directive as of January 1, 2021, the health insurance company can post the data before sending the electronic health card and its personal identification number (PIN) to the insured person<br>§ 34 Paragraph 1 Sentence 1 Numbers 1 to 6 and 10 of the Federal Registration Act can be retrieved from the population register.<br><br>§ 291a Electronic health card as proof of insurance and means of billing

					(1) The electronic health card, with the information specified in paragraphs 2 to 5, serves as proof of authorization to use services within the scope of statutory medical care (proof of insurance) and for billing with the service providers. When seeking medical treatment, the insured person confirms membership in the health insurance company by signing the doctor's billing slip.<br>(2) The following data must be stored on the electronic health card:<br>1. the name of the issuing hospital<br>cash register, including a license plate for<br>the Association of Statutory Health Insurance Physicians, in whose<br>the insured person's place of residence,<br>2. the last name and first name of the insured person,<br>3. the date of birth of the insured person,<br>4. the gender of the insured person,<br>5. the address of the insured person,<br>6. the health insurance number of the insured person,<br>7. the insured status, for the groups of people in accordance with Section 264 paragraph 2, the status of mandated care,<br>8. the co-payment status of the insured person,<br>9. the day the insurance coverage begins,<br>10. if the electronic health card is valid for a limited period, the date on which the deadline expires,<br>11. in the case of agreements in accordance with Section 264 Paragraph 1 Sentence 3, second half, the statement that the recipient is a recipient of health services in accordance with Sections 4 and 6 of the Asylum Seekers Benefits Act.<br>(3) In addition to the data referred to in paragraph 2, the electronic health card can also contain the following data:<br>1. Information n to optional tariffs according to Section 53,<br>2. Information on additional contractual relationships,<br>3. in the cases of Section 16 Paragraph 1 Sentence 1 Numbers 2 to 4 and Paragraph 3a, information on the suspension of the entitlement to benefits,<br>4. further information to the extent that the processing of this data is necessary to fulfill tasks that are legally assigned to the health insurance companies and<br>5. Information to prove entitlement to receive services in another member state of the European Union, another contracting state to the Agreement on the European Economic Area or in Switzerland.<br>(4) The information in accordance with paragraphs 2 and 3 numbers 1 to 4 must be stored on the electronic health card in a form that is suitable for automatic transfer to the billing documents and forms intended for statutory medical care in accordance with Section 295 paragraph 3 number 1 and 2.<br><br>“Eleventh chapter Telematics infrastructure<br>First section requirements for the telematics infrastructure<br>§ 306 Telematics infrastructure<br>(1) The Federal Republic of Germany, represented by the Federal Ministry of Health, the National Association of Health Insurance Funds, the National Association of Statutory Health Insurance Physicians, the National Association of Statutory Health Insurance Dentists, the Federal Medical Association, the

					Federal Dental Association, the German Hospital Association and those responsible for the protection of economic interests<br>The leading umbrella organization of pharmacists at the federal level creates the telematics infrastructure. The telematics infrastructure is the interoperable and compatible information, communication and security infrastructure,<br>which serves to network service providers, payers, insured persons and other players in the healthcare system as well as rehabilitation and care and in particular 1. is necessary for the use of the electronic health card and the applications of the telematics infrastructure, 2. is suitable a) for the use of other applications the telematics infrastructure without using the electronic health card in accordance with Section 327 and b) for use for health and nursing research purposes. The Federal Republic of Germany, represented by the Federal Ministry of Health, and the leading organizations named in sentence 1 carry out the task according to sentence 1 in accordance with Section 310 through a telematics company.<br>(2) The telematics infrastructure includes 1. a decentralized infrastructure consisting of components for authentication and the secure transmission of data to the central infrastructure, 2. a central infrastructure consisting of<br>a) secure access services as an interface to the decentralized infrastructure and b) a secure network including<br>services necessary for operation and 3. an application infrastructure consisting of services for the applications according to this chapter.<br>(3) For the processing of personal data belonging to the special categories within the meaning of Article 9 of Regulation (EU) 2016/679 in the telematics infrastructure, a high level of protection corresponding to the special protection needs applies, which is ensured by appropriate technical and organizational measures within the meaning of Article 32 of Regulation (EU) 2016/679<br>must be taken into account.<br>(4) Applications within the meaning of this chapter are user-related functionalities based on services and components approved in accordance with Section 325 for processing health data in the telematics infrastructure as well as other user-related functionalities in accordance with Section 327. Services within the meaning of sentence 1 are centrally provided and in the telematics infrastructure operated technical systems that implement individual functionalities of the telematics infrastructure. Components are decentralized technical systems or their components.<br><br>§ 307 Data protection responsibilities<br>(1) The processing of personal data using the components of the decentralized infrastructure in accordance with Section 306 Paragraph 2 Number 1 is the responsibility of those who use these components for the purposes of authentication and for the secure processing of data via the central infrastructure, insofar as they have the Decide on the means of data processing. This applies to the proper commissioning, maintenance and use of the components.<br><br>Eighth title<br>Availability of data from telematics infrastructure applications for research purposes<br>§ 363 Processing of data from the electronic patient record for research purposes

					(1) Insured persons can voluntarily release the data in their electronic patient files for the research purposes listed in Section 303e paragraph 2 numbers 2, 4, 5 and 7.<br>(2) The released data in accordance with paragraph 1 is transmitted to the research data center in accordance with Section 303d and requires the insured person's informed consent as a processing condition. The insured person declares their consent via the user interface of a suitable device. The scope of data release can en insured persons can choose freely and on specific terms<br>Categories or to groups of documents and records or to specific documents and records. The release is documented in the electronic patient file.<br>(3) Those responsible for data processing in the electronic patient file in accordance with Section 341 Paragraph 4 pseudonymise and encrypt the data released with the informed consent in accordance with Paragraphs 1 and 2, provide them with a work number and transmit them<br>1. to the research data center the pseudonymized and encrypted data including the work number,<br>2. to the trust offices in accordance with Section 303c the delivery pseudonym for the released data and the corresponding work number.<br>The trust converts the delivery pseudonyms into cross-period pseudonyms and sends the research data center a list of the cross-period pseudonyms with the associated work numbers. With<br>Using the cross-period pseudonym and the work number that has already been sent, the research data center links the released data with the data from previous transmissions available in the research data center.<br>(4) The data released to the research data center may be processed by it in order to fulfill its tasks and, upon request, made available to those authorized to use it in accordance with Section 303e paragraph 1 numbers 6, 7, 8, 10, 13, 14, 15 and 16.<br>be provided. § 303a paragraph 3, § 303c paragraph 1 and 2, §§ 303d, 303e paragraph 3 to 6 as well as §§ 303f and 397 paragraph 1 numbers 2 and 3 apply accordingly.<br>(5) Before giving informed consent, the insured person is fully informed in accordance with Section 343 Paragraph 1 Sentence 1 about the voluntary nature of data release, the pseudonymized data transfer to the research data center, the possible authorized users, the purposes, the tasks of the research data center, and the types of data provision on groove<br>to inform those authorized to do so about the ban on re-identification of insured persons and service providers as well as the options for revocation. According to Section 343 Paragraph 1 Sentence 3 Number 16, this information is part of the appropriate<br>Information material from health insurance companies.<br>(6) If the informed consent in accordance with paragraph 2 is revoked, the corresponding data that has already been transmitted to the research data center will be deleted in the research data center. The deletion procedure is analogous to<br>Data transfer and linking in paragraph 3. The data transmitted until the consent was revoked in accordance with paragraph 2 and already used for specific research projects may continue to be processed for these research projects.<br>The rights of the data subject under Articles 17, 18 and 21 of Regulation (EU) 2016/679 are excluded for these research projects. Consent can be revoked and given via the user interface of a suitable device.

Germany	2023		Gesetz über die Arbeitsweise der Bundesagentur für Sprunginnovationen und zur Flexibilisierung ihrer rechtlichen und finanziellen Rahmenbedingungen (SPRIND-Freiheitsgesetz — SPRINDFG)	Law on the functioning of the Federal Agency for Leap Innovations and on making its legal and financial framework more flexible (SPRIND Freedom Act — SPRINDFG)	§ 1<br>Funding tasks, lending<br>(1) The Federal Agency for Jump Innovations SPRIND GmbH, based in Leipzig under the Commercial register entry HRB 36977 (SPRIND) is provided with promotional tasks in the field of entrusted with breakthrough innovations. Funding tasks within the meaning of this law are identification, validation and<br>public funding for projects that have the potential for breakthrough innovation. SPRIND acts<br>with the aim of creating new added value through new, highly innovative products, processes or services, particularly in Germany and Europe. This is done with the intention of securing the intellectual property supported by SPRIND.<br>(2) Leap innovations within the meaning of this law are innovations that result from novel solutions fundamentally change or replace existing products, technologies or business models in markets and thereby open up new markets and great value creation potential or solve a significant technological, social or ecological problem
Italy	2023		DECRETO 19 ottobre 2023: Riparto delle risorse del «Fondo per l'innovazione tecnologica e la digitalizzazione», per l'anno 2023. (23A06326)	LEGISLATIVE DECREE 19 October 2023: Distribution of resources of the "Fund for technological innovation and digitalization", for the year 2023. (23A06326)	Art 1(1). B) euro 26,045,792.50 are intended to cover expenses for interventions and purchases of goods and services, support measures and projects aimed at encouraging the country's technological innovation, including the implementation of the Italian strategy for ultra-broadband and the Italian participation to projects and initiatives promoted by organizations at European and international level, as well as from international organizations and multilateral fora for the definition of digital policies, the digitalisation of businesses, the development and diffusion of digital services and technologies among citizens, businesses and public administrations, including the diffusion of skills, education and digital culture, the development of emerging technologies, with particular attention to AI, also in implementation of the Italian strategy for AI
Italy	2018	-	DECRETO LEGISLATIVO 2 gennaio 2018, n. 1 (Raccolta 2018) (1): Codice della protezione civile. (18G00011)	LEGISLATIVE DECREE 2 January 2018, n. 1 (Collection 2018) (1): Civil Protection Code. (18G00011)	Art 1(1). The National Civil Protection Service, hereinafter National Service, defined as being of a public utility, is the system that exercises the civil protection function consisting of the set of skills and activities aimed at protecting life, physical integrity, goods, settlements, animals and the environment from damage or the danger of damage resulting from calamitous events of natural origin or resulting from human activity.
Italy	2018		Legge 30 dicembre 2018, n. 145: Bilancio di previsione dello Stato per l'anno finanziario 2019 e bilancio pluriennale per il triennio 2019-2021 (18G00172)	LAW 30 December 2018, n.145: State budget forecast for the 2019 financial year and a multi-year budget for the three-year period 2019-2021 (18G00172)	Art 1(226). To pursue the objectives of economic and industrial policy, also related to the Industry program 4.0, as well as to increase the competitiveness and productivity of the economic system, in the Ministry's forecast of economic development a fund was established for interventions aimed at encouraging the development of AI technologies and applications, blockchain and internet of things, with a budget of 15 million euros for each of the years 2019, 2020 and 2021.The Fund is intended to finance: a) research and innovation projects carried out in Italy by public and private entities, including foreign countries, in strategic areas for the development of AI, blockchain and the internet of things, functional to the country's competitiveness; b) competitive initiatives for achievement of specific technology and application objectives; c) operational and administrative support for the implementation of what is mentioned in letters a) and b), in order to enhance the results and encourage their transfer towards the economy production system, with particular attention to SMEs.

Italy	2018		DECRETO LEGISLATIVO 2 gennaio 2018, n. 1 (Raccolta 2018) (1): Codice della protezione civile. (18G00011)	LEGISLATIVE DECREE 2 January 2018, n. 1 (Collection 2018) (1): Civil Protection Code. (18G00011)	Art 19(1). The scientific community participates in the National Service through integration into the civil protection activities, referred to in article 2, of knowledge and products deriving from research and innovation activities, including those already available, which have reached a level of development and consensus recognized by the scientific community according to current practices, also the result of initiatives promoted by the European Union and International organizations addressing natural disasters.
Italy	2017	-	DECRETO 12 settembre 2017 , n. 214: Regolamento sulle modalità di costituzione e sulle forme di finanziamento di centri di competenza ad alta specializzazione, nel quadro degli interventi connessi al Piano nazionale industria 4.0, in attuazione dell'articolo 1, comma 115, della legge 11 dicembre 2016, n. 232 (legge di bilancio 2017). (17G00223)	DECREE 12 September 2017, n. 214: Regulation on the methods of establishment and forms of financing of highly specialized competence centers, in the framework of the interventions connected to the National Industry Plan 4.0, in implementation of article 1, paragraph 115, of law 11 December 2016, n. 232 (2017 budget law). (17G00223)	Art 2(1). This regulation regulates […] the methods of establishment and forms of financing, within the limit of 20 million euros for 2017 and 10 million euros for 2018, of highly specialized competence centers, with the purpose of promoting and carrying out research projects, technology transfer and training on advanced technologies, within the framework of the interventions related to the National Industry Plan 4.0.
Italy	2022	-	LEGGE 15 luglio 2022 , n. 99: Istituzione del Sistema terziario di istruzione tecnologica superiore. (22G00108)	LAW 15 July 2022, n. 99: Establishment of the tertiary system of higher technological education. (22G00108)	Art 2(1). Within the framework of the overall tertiary system of higher technological education referred to in Article 1, the ITS Academies have the priority task of strengthening and expanding the professional training of higher technicians with high technological and technical-professional skills, with the aim of contributing in systematic way to support measures for the economic development and competitiveness of the production system, progressively bridging the mismatch between job demand and supply, which conditions the development of businesses, especially SMEs small and medium-sized ones, and to ensure, with continuity, the supply of superior higher technicians at post-secondary level in relation to the technological areas considered strategic in the context of industrial and technological development and ecological reconversion policies. In addition to what is foreseen in the first period, the ITS Academies have the task of supporting the diffusion of scientific and technological culture, the permanent continuing orientation of young people towards technical professions and the providing information of to their families, updating and training in service of teachers of scientific, technological and technical-professional disciplines in schools and professional training, active employment policies, especially with regard to the transition of young people into the world of work, the continuous training of highly specialized technical workers, within the framework of lifelong learning, and technology transfer, especially towards SMEs small and medium-sized enterprises.
Italy	2022	-	LEGGE 15 luglio 2022 , n. 99: Istituzione del Sistema terziario di istruzione tecnologica superiore. (22G00108)	LAW 15 July 2022, n. 99: Establishment of the tertiary system of higher technological education. (22G00108)	Art 2(2). A strategic priorities of the ITS Academy is the professional training of superior higher technicians to satisfy training needs in relation to the digital transition, also for the purposes of expanding digital services in the fields of identity, authentication, healthcare and justice, constitutes a strategic priority of the ITS Academy, innovation, competitiveness and culture, the green revolution and the ecological transition, as well as infrastructure for sustainable mobility

Italy	2017	-	DECRETO 12 settembre 2017 , n. 214: Regolamento sulle modalità di costituzione e sulle forme di finanziamento di centri di competenza ad alta specializzazione, nel quadro degli interventi connessi al Piano nazionale industria 4.0, in attuazione dell'articolo 1, comma 115, della legge 11 dicembre 2016, n. 232 (legge di bilancio 2017). (17G00223)	DECREE 12 September 2017, n. 214: Regulation on the methods of establishment and forms of financing of highly specialized competence centers, in the framework of the interventions connected to the National Industry Plan 4.0, in implementation of article 1, paragraph 115, of law 11 December 2016, n. 232 (2017 budget law). (17G00223)	Art 2(2). The highly specialized competence centers implement a structured program of activities - including the services referred to in Article 5.
Italy	2017	-	DECRETO 12 settembre 2017 , n. 214: Regolamento sulle modalità di costituzione e sulle forme di finanziamento di centri di competenza ad alta specializzazione, nel quadro degli interventi connessi al Piano nazionale industria 4.0, in attuazione dell'articolo 1, comma 115, della legge 11 dicembre 2016, n. 232 (legge di bilancio 2017). (17G00223)	DECREE 12 September 2017, n. 214: Regulation on the methods of establishment and forms of financing of highly specialized competence centers, in the framework of the interventions connected to the National Industry Plan 4.0, in implementation of article 1, paragraph 115, of law 11 December 2016, n. 232 (2017 budget law). (17G00223)	Art 5. The activity program is aimed at providing a service of:<br>a) orientation towards businesses, in particular SMEs, through the preparation of a series of tools aimed at supporting businesses in assessing their level of digital and technological maturity;<br>b) training for companies, in order to promote and disseminate skills in the Industry 4.0 field through training activities in the classroom and on the production line and on real applications, using, for example, demonstration production lines and development of use cases, at the aim of supporting the understanding by the benefiting companies of the concrete benefits in terms of reducing operating costs and increasing the competitiveness of the offer;<br>c) implementation of innovation, industrial research and experimental development projects proposed by companies, including those of a collaborative nature between them, and provision of technology transfer services in the Industry 4.0 context, also through actions to stimulate demand for business innovation, by of businesses, especially by SMEs.
Italy	2022	-	LEGGE 15 luglio 2022 , n. 99: Istituzione del Sistema terziario di istruzione tecnologica superiore. (22G00108)	LAW 15 July 2022, n. 99: Establishment of the tertiary system of higher technological education. (22G00108)	Art 5(1). The ITS Academy training courses are divided into semesters and are structured as follows:<br>a) fifth level EQF training courses, which last four semesters, with at least 1,800 hours of training, corresponding to the fifth level of the European Qualifications Framework for lifelong learning, referred to in recommendation 2017/C 189/03 of Council, of 22 May 2017;<br>b) EQF sixth level training courses, which last six semesters, with at least 3,000 hours of training, corresponding to the sixth level of the aforementioned European Qualifications Framework for lifelong learning. The new sixth level EQF training courses can be activated exclusively for professional figures who require a high number of hours of internship, incompatible with the two-year structure of the training course, and who present specific needs, to be identified by decree of the President of the Council of ministers, upon proposal of the Minister of Education and the Minister of Universities and Research, subject to agreement within the Permanent Conference for relations between the State, the regions and the autonomous provinces of Trento and Bolzano.

Italy	2017	-	DECRETO 12 settembre 2017 , n. 214: Regolamento sulle modalità di costituzione e sulle forme di finanziamento di centri di competenza ad alta specializzazione, nel quadro degli interventi connessi al Piano nazionale industria 4.0, in attuazione dell'articolo 1, comma 115, della legge 11 dicembre 2016, n. 232 (legge di bilancio 2017). (17G00223)	DECREE 12 September 2017, n. 214: Regulation on the methods of establishment and forms of financing of highly specialized competence centers, in the framework of the interventions connected to the National Industry Plan 4.0, in implementation of article 1, paragraph 115, of law 11 December 2016, n. 232 (2017 budget law). (17G00223)	Art 8. Innovation, industrial research and experimental development projects, presented by companies, eligible for the benefits provided to the highly specialized competence centre, must include:<br>a) a concrete intervention plan, detailed in terms of investments, costs and times;<br>b) the estimate of the economic benefits for the company in terms of reduction of inefficiencies, waste and costs, also in terms of improvement of the quality of processes and products;<br>c) drafting of a financial plan to cover the costs of the project.
Malta	2018		eli/sl/586.10	Processing of Data concerning Health for Insurance Purposes Regulations	4.(1) The processing of data concerning health shallbe deemed to be in the substantial public interest when such processing is necessary for the purpose of the business of insurance or insurance distribution activities
Malta	2018	2023	eli/cap/586	Data Protection Act	7.A controller shall consult with, and obtain priorauthorisation from, the Commissioner where the controller intends toprocess in the public interest:(a) genetic data, biometric data or data concerninghealth for statistical or research purposes; or(b) special categories of data in relation to themanagement of social care services and systems, including forthe purposes of quality control, management information andthe general national supervision and monitoring of such servicesand systems:Provided that, where genetic data, biometric data or dataconcerning health are required to be processed for research purposes,the Commissioner shall consult a research ethics committee or of aninstitution recognised by the Commissioner for the purposes of thisarticle.
Malta	2018	2023	eli/cap/586	Data Protection Act	6.(1) Subject to the provisions of sub-article (4), controllers and processors may derogate from the provisions of Articles 15, 16, 18 and 21 of the Regulation for the processing of personal data for scientific or historical research purposes or official statistics in so far as the exercise of the rights set out in those Articles:(a) is likely to render impossible or seriously impair the achievement of those purposes; and(b) the data controller reasonably believes that such derogations are necessary for the fulfilment of those purposes.(2) Subject to the provisions of sub-article (4), controllers and processors may derogate from the provisions of Articles 15, 16, 18, 19, 20 and 21 of the Regulation for the processing of personal data for archiving purposes in the public interest in so far as the exercise of the rights set out in those Articles: (a) is likely to render impossible or seriously impair the achievement of those purposes; and(b) the controller reasonably believes that such derogations are necessary for the fulfilment of those purposes.(3) Where data processing referred to in sub-articles (1) and (2) serves at the same time another purpose, the derogations shall apply only to processing for the purposes referred to in those sub-articles.(4) Processing for the purposes referred to in sub-articles (1) and (2) shall be subject to appropriate safeguards for the rights and freedoms of the data subject, including pseudonymisation and other technical and organisational measures to ensure respect for theprinciple of data minimisation:Provided that, where such purposes can

					be fulfilled by processing which does not permit, or no longer permits, the identification of data subjects, those purposes shall be fulfilled in that manner.
Malta	2021	2021		Re-Use of Public Sector Information (Amendment) Act, 2021	(e) immediately after sub-article (7) thereof there shall beadded the following new sub-article:Amendment of article 17 of the principal Act.12.Sub-article (1) of article 17 of the principal Act shall besubstituted by the following: The total income of those bodies from supplying andallowing re-use of documents over the appropriateaccounting period shall not exceed the cost of collection,production, reproduction and dissemination and datastorage, together with a reasonable return on investment,and, where applicable, the anonymisation of personal dataand measures taken to protect commercially confidentialinformation.Charges shall be calculated in line with the accountingprinciples applicable.";"(8) The re-use of the following shall be free ofcharge for the user:(a) subject to the Schedule, the high-valuedatasets, as listed in accordance with item 1 of thatSchedule; (b) research data referred to in article 8A."."(1) The re-use of documents shall not be subject toconditions, unless such conditions are objective,proportionate, non-discriminatory and justified ongrounds of a public interest objective.When re-use is subject to conditions, those conditionsshall not unnecessarily restrict possibilities for re-useand shall not be used to restrict competition.VERŻJONI ELETTRONIKA
Malta	2019		eli/act/2019/3	III of 2019 – Public Administration Act, 2019	Department for Health Regulation<br>Superintendent of Public HealthTo safeguard public health and to license, monitor and inspect the provision of healthcare services<br><br>Department for Healthcare Services<br>Director General (Healthcare Services)To ensure the effective and efficient operation and delivery of healthcare services<br><br>Department for Policy in Health<br>Chief Medical OfficerTo advise the Minister for Health on all matters relating to health policy
Malta	2019		eli/act/2019/3	III of 2019 – Public Administration Act, 2019	Department of InformationDirector (Information)Press RegistrarTo provide the public with up to date, comprehensive and meaningful information on Government policies, services and activities, and on matters which may be of public interest

Malta	2019		eli/ln/2019/263	Processing of Personal Data (Secondary Processing) (Health Sector) Regulations, 2019	3. Secondary processing of personal data in the health sectorshall be permitted where such secondary processing is related to<br>(a) the processing and analysis of records kept by all entities falling within the ambit of the health sector, and the administration of the systems and services by entities, which entities are licensed to deliver any kind of service to patients or individuals, for the purpose of managing and enhancing the health service;<br>(b) the analysis of health records supplied to the Ministry for Health in accordance with licensing legislation, contractual obligations, compliance with EU regulations on public health statistics and to safeguard other public health interests, to produce the indicators required for monitoring, to ensure the quality and cost-effectiveness of the health services at national level;<br>(c) the monitoring of contractual obligations, including the purposes of quality control, management information and monitoring of such services and systems, arising from the public-private partnerships and partnerships with non-governmental organisations which the Ministry for health has entered into with third parties, to ensure that the aforementioned partners are adhering to their contractual obligations to deliver a safe and accessible service;<br>(f) the investigation and monitoring of health threats, which typically requires the processing of health record data forthe protection of public health; and<br>(g) access to health records, for the purpose of research activities
Malta	2018		eli/act/2018/31	XXXI of 2018 – Malta Digital Innovation Authority Act, 2018	4. (3) The Authority shall encourage the development ofinnovative technology in as wide a manner and for as many uses aspossible so as to achieve its benefits in as many economic and socialsectors as possible, including, but not limited to, in financial services,health and education, voluntary organisations, public administrationand transport.
Malta	2018		eli/act/2018/31	XXXI of 2018 – Malta Digital Innovation Authority Act, 2018	3.The Government shall, through the establishment of theAuthority in Malta, seek the development of the innovative technologysector in Malta through proper recognition and regulation of relevantinnovative technology arrangements and related services.This shall be done:(a) in full respect of the importance of not hinderinginnovation and the efforts and potential of the start-up sector inthis area of activity;VERŻJONI ELETTRONIKA<br><br>A 1357(b) considering that the approach to recognition andregulation will be moderated by the pace of change anddevelopment taking place in this sector; and(c) in a manner ensuring that there are standards inplace for the protection of consumers and investors, theintegrity of the market and the public interest in general to beprotected against abuse and non-compliance with mandatorylaws intended for such purposes.
Malta	2018		eli/act/2018/31	XXXI of 2018 – Malta Digital Innovation Authority Act, 2018	8.(1) Except as expressly provided for in other provisionsof this Act, the Authority shall act independently and shall not seek ortake instructions from any other body or person.(2) (a) The Minister may, in relation to matters that appearto him to affect the public interest, from time to time give to theAuthority policy directions in writing of a general character, notinconsistent with the provisions of this Act, on the policies to befollowed in the carrying out of the functions vested in the Authorityby or under this Act.
Malta	2018		eli/act/2018/31	XXXI of 2018 – Malta Digital Innovation Authority Act, 2018	29.The Authority, acting in line with the governing principlesand in furtherance of the regulatory objectives established by this Act,or any other special law which the Authority is entitled to administeror enforce, may additionally refuse to grant an authorisation in any ofthe following instances:(a) if the Authority believes that

					the innovativetechnology service or innovative technology arrangement beingproposed is not compliant with the regulatory instruments inforce; or(b) if the Authority believes that granting anauthorisation to the applicant may pose a risk to the reputationof Malta or be otherwise not in the public interest or contrary toregulatory objectives established by the Ac
Malta	2020		eli/cap/592	Innovative Technology Arrangements and Services Act	3 Principles of recognition<br>(2) This Act provides for various methods of recognition which the Authority may extend to varied types of innovative technology arrangements and innovative technology services as are referred to in the Schedules and this in accordance with the provisions of this Act<br><br>Certification of Innovative Technology Arrangements<br>7 Principles relating to certification.<br>(1) The Authority may certify different innovative technology arrangements for one or more specified purposes and with reference to:(a) qualities;(b) features;(c) attributes; (d) behaviours; or(e) aspects,as may be determined by the Authority, and which shall be stated in the certification<br><br>The following shall be considered to be innovative technology arrangements for the purposes of this Act: 1. software and architectures which are used in designing and delivering DLT which ordinarily, but not necessarily:(a) uses a distributed, decentralized, shared and, or replicated ledger;(b) may be public or private or hybrids thereof;(c) is permissioned or permissionless or hybrids thereof;(d) is secure to a high levelagainst retrospective tampering, such that the history of transactions cannot be replaced;(e) is protected with cryptography; and(f) is auditable 2. Software and other architectures, not necessarily used in the context of DLT, smart contracts and related applications as well as other similar arrangements, but which are used or meant to be used, as a stand-alone or as part of a solution in sectors and areas which are deemed to be of a risky or critical nature, where their failure or misuse could amongst other things result in loss of life, grave prejudice to the well-being and rights of natural persons, significant asset loss or damage and significant damage to the environment.
Malta	2018		eli/act/2018/16	XVI of 2018 – Gaming Act, 2018	17.The Minister shall, by regulations, establish the overallparameters, criteria and conditions for protecting vulnerable persons in order to minimise potential risk to their health associated with participation in games; provided that the Minister may authorise the Authority to devise all reasonable parameters, criteria, conditions andstandards by way of directives or other binding instruments to beissued by the Authority.
Malta	2020		eli/sl/458.59	MEDICAL DEVICES AND IN-VITRO DIAGNOSTIC MEDICAL DEVICES PROVISION ON THE MALTESE MARKET REGULATIONS	"national medical device database" means thecollection of data or other materials in relation tomedical devices which are arranged in a systematic ormethodical order and which are individually accessibleby electronic or other means;

Malta	2020		eli/sl/458.59	MEDICAL DEVICES AND IN-VITRO DIAGNOSTIC MEDICAL DEVICES PROVISION ON THE MALTESE MARKET REGULATIONS	5.(1) No person shall carry out importation, wholesaling,manufacturing or re-purposing in Malta of any medical device which isintended for trade within the European Union and, or the local marketprior to obtaining all the necessary approvals, authorisations, licences,permits and, or any notification/s as required by or under theseregulations or any other law.(2) Any person intending to carry out any of the activitiesidentified in sub-regulation (1) shall:(a) comply with the provisions of these regulations;(b) comply with all national and European Unionregulations, including international obligations resulting fromany treaty to which Malta may from time to time be a part of, asmay be applicable;(c) obtain the necessary authorisation from thecompetent authority;(d) possess the necessary qualifications and haveundergone any necessary training as may be required by the<br><br>MEDICAL DEVICES AND IN-VITRO DIAGNOSTIC MEDICAL DEVICES PROVISION ON THE MALTESE MARKET [S.L. 458.593competent authority;(e) comply with any other applicable law;(f) have a medical device registered person in Malta;and(g) insert details of all medical devices which hemarkets locally in the national medical device database kept bythe competent authority
Malta	2020		eli/sl/458.59	MEDICAL DEVICES AND IN-VITRO DIAGNOSTIC MEDICAL DEVICES PROVISION ON THE MALTESE MARKET REGULATIONS	10.(1) Any economic operator that has receivedcomplaints or reports from healthcare professionals, patients or usersabout incidents related to a medical device which they have madeavailable on the local market, shall immediately forward this informationto the competent authority.(2) Every economic operator shall keep a register containingdetails of complaints, of non-conforming medical devices and of recallsand withdrawals of medical devices, and shall keep the competentauthority informed of such monitoring.(3) The competent authority may request any otherinformation from any person or entity as it may deem necessary for thecarrying out of its functions pursuant to these regulations.
Poland	2019		Ustawa z dnia 21 lutego 2019 r. o zmianie niektórych ustaw w związku z zapewnieniem stosowania rozporządzenia Parlamentu Europejskiego i Rady (UE) 2016/679 z dnia 27 kwietnia 2016 r. w sprawie ochrony osób fizycznych w związku z przetwarzaniem danych osobowych i w sprawie swobodnego przepływu takich danych oraz uchylenia dyrektywy 95/46/WE (ogólne rozporządzenie o ochronie danych)	Act of 21 February 2019 amending certain acts in connection with ensuring the application of Regulation (EU) 2016/679 of the European Parliament and of the Council of 27 April 2016 on the protection of natural persons with regard to the processing of personal data and on the free movement of such data, and repealing Directive 95/46/EC (General Data Protection Regulation)	Art. 157. In the Act of 27 October 2017 on primary health care (Journal of Laws, item 2217)<br><br>in art. 10 section 5 is replaced by the following:<br><br>“5. Completed declarations of election referred to in section 1 point 1, the healthcare provider keeps in his/her<br><br>headquarters or place where primary health care services are provided, ensuring their availability<br><br>to the beneficiaries who submitted them, in compliance with the requirements arising from the provisions on personal data protection

Poland	2019		Ustawa z dnia 21 lutego 2019 r. o zmianie niektórych ustaw w związku z zapewnieniem stosowania rozporządzenia Parlamentu Europejskiego i Rady (UE) 2016/679 z dnia 27 kwietnia 2016 r. w sprawie ochrony osób fizycznych w związku z przetwarzaniem danych osobowych i w sprawie swobodnego przepływu takich danych oraz uchylenia dyrektywy 95/46/WE (ogólne rozporządzenie o ochronie danych)	Act of 21 February 2019 amending certain acts in connection with ensuring the application of Regulation (EU) 2016/679 of the European Parliament and of the Council of 27 April 2016 on the protection of natural persons with regard to the processing of personal data and on the free movement of such data, and repealing Directive 95/46/EC (General Data Protection Regulation)	Art. 107. 1. Who processes personal data even though their processing is not permitted or is not intended to be processed?<br><br>is entitled,<br><br>shall be subject to a fine, restriction of liberty or imprisonment for up to two years.<br><br>2. If the act specified in section 1 applies to data revealing racial or ethnic origin, political opinions,<br><br>religious or philosophical beliefs, trade union membership, genetic data, biometric data processed for the purpose of uniquely identifying a natural person, data regarding health, sexuality or sexual orientation,<br><br>shall be subject to a fine, restriction of liberty or imprisonment for up to three years
Poland	2023		Rozporządzenie Rady Ministrów z dnia 12 lipca 2023 r. w sprawie zakresu danych i wykazu rejestrów publicznych oraz systemów teleinformatycznych podmiotów publicznych, z których użytkownik aplikacji mObywatel może pobrać dane	Regulation of the Council of Ministers of 12 July 2023 on the scope of data and the list of public registers and ICT systems of public entities from which the user of the mObywatel application can download data	"Art. 76. 1. The minister responsible for informatization in consultation with the minister responsible for health matters<br>shall announce in the Official Journal of the Republic of Poland "Monitor Polski" a notice specifying the date of implementation of technical solutions enabling the provision of services specified in:<br>1) Article 7c paragraph 2a-2c of the Act amended in Art. 29,<br>2) Article 49 paragraph 5 item 46 of the Act amended in Article 36,<br>3) Article 41 (1) second sentence and (1d) of the Law amended by Article 42,<br>4) Article 24 (1c) and (1d) of the Law amended by Article 45,<br>5) Article 17 (3) (2), (3b) and (3c) of the Law amended by Article 50,<br>6) Article 113d of the Law amended by Article 52,<br>7) Article 71 (5a) and (5b) of the Law amended by Article 55,<br>8) Article 28 (2) and (3) of the law amended in Article 59<br>- as amended by this Law.<br>(2) The communication shall be announced at least 14 days before the date of implementation of technical solutions specified<br>in this communication"
Portugal	2021	NA	Lei n.º 27/2021: Carta Portuguesa de Direitos Humanos na Era Digital	Law n.º 27/2021: the Portuguese Charter of Human Rights in the Digital Age	Art 9(1). The use of artificial intelligence should be guided by respect for fundamental rights, ensuring a fair balance between the principles of explainability, security, transparency and responsibility, which meets the circumstances of each specific case and establishes processes aimed at avoiding any prejudice and forms of discrimination.
Portugal	2021	NA	Lei n.º 27/2021: Carta Portuguesa de Direitos Humanos na Era Digital	Law n.º 27/2021: the Portuguese Charter of Human Rights in the Digital Age	Art 9(2). Decisions with a significant impact on the sphere of recipients that are taken through the use of algorithms must be communicated to the interested parties, being subject to appeal and auditable, under the terms provided for by law.

Portuga l	2021	NA	Lei n.º 27/2021: Carta Portuguesa de Direitos Humanos na Era Digital	Law n.º 27/2021: the Portuguese Charter of Human Rights in the Digital Age	Art 16(1). Everyone has the right to free intellectual, artistic, scientific and technical creation, as well as to benefit, in the digital environment, from the protection legally conferred on works, performances, productions and other content protected by intellectual property rights.
Portuga l	2023	NA	Portaria n.º 360/2023, de 14 de novembro	Ordinance No.º 360/2023, of November 14	Art 2. This ordinance aims to establish the standards for the creation of Innovation and Incubation Centers, with the purpose of promoting the development of entrepreneurship and other economic activities, at local and regional level, supporting the creation and growth of companies and other entities, regardless of the legal nature, with a view to the creation of jobs.
Portuga l	2023	NA	Portaria n.º 360/2023, de 14 de novembro	Ordinance No.º 360/2023, of November 14	Art 5. The creation of Innovation and Incubation Centers depends on the existence of facilities available and appropriate to its installation, and must also comply with the following assumptions:<br>a) Locate itself in geographical areas affected by processes of restructuring sectors of activity, in interior territories or even in places where there is a need to fill gaps in terms of offering support to entrepreneurship;<br>b) Promote dynamics of innovation and diversification and modernization of productive and business activity;<br>c) Satisfy regional or local needs, through the development of projects aimed at the access of populations to new goods and services;<br>d) Encourage activities of high added value that use new technologies and use highly specialized human resources;<br>e) Involve local economic agents that contribute to the creation of an ecosystem favorable to the development of economic activity.
Portuga l	2023	NA	Portaria n.º 360/2023, de 14 de novembro	Ordinance No.º 360/2023, of November 14	Art 7. The Innovation and Incubation Centers have the following attributions:<br>a) Support the development of companies and other non-profit entities, contributing to the success of their promoters and to the creation of jobs, including the job itself;<br>b) Promote the innovation and competitiveness of the supported entities, through the provision of resources and services, which contribute to the development of new products and services, as well as to the improvement of efficiency and productivity;<br>c) Develop, by itself or in cooperation with other entities, initiatives that prove appropriate to the promotion of entrepreneurship and the attraction, appreciation and fixation of talent;<br>d) Contribute to the sustainable development of the site and region of implementation of the CII, through the promotion of socially and environmentally responsible business models, boosting the respective economy and contributing to the diversification of the business fabric and the creation of quality jobs.
Portuga l	2021	NA	Decreto-Lei n.º 67/2021, de 30 de julho	Decree-Law No. º 67/2021, of July 30	Chapter 1; Art 1. This decree-law establishes the regime and defines the governance model for the promotion of technology-based innovation through the creation of technological free zones (ZLT).
Portuga l	2021	NA	Decreto-Lei n.º 67/2021, de 30 de julho	Decree-Law No. º 67/2021, of July 30	Chapter 1; Art 4(1). The ZLT do not imply the derogation from the existing legal framework must respect the regime provided for in this decree-law and in the applicable sectoral legislation and are created by ordinance of the members of the Government responsible for the areas of economy, science and the area that guards the sector of activity in which the ZLT is inserted.
Portuga l	2021	NA	Decreto-Lei n.º 67/2021, de 30 de julho	Decree-Law No. º 67/2021, of July 30	Chapter 1; Art 4(2). The special ZLTs, which imply the derogation from the existing legal framework, are created by legislative act, as provided for in Article 6(6), preceded, where applicable, by prior hearing of the

					competent regulatory authority by reason of the matter, the regime provided for in this decree-law is applied subsidiarily.
Portugal	2021	NA	Decreto-Lei n.º 67/2021, de 30 de julho	Decree-Law No. º 67/2021, of July 30	Chapter 1; Art 6(1). The constitutive acts of the ZLT must identify:<br>a) The delimitation of areas, sectors of activity or priority technologies for testing, including air, land and maritime space, always safeguarding the possibility of testing technologies, products, services and processes that cross several areas or sectors;<br>b) The geographical scope of the ZLT;<br>c) The objectives of dynamizing the business fabric in the geographical delimitation selected for the installation of the ZLT;<br>d) The availability of resources, including human, material and infrastructure, to the promoters to carry out the tests, with an indication of the following:<br>i) ZLT's own resources and ZLT's partner resources, if existing;<br>ii) The conditions for the availability of resources to the promoters of the tests;<br>iii) The conditions for the inclusion or removal of resources from ZLT;<br>e) The identification of the managing entity responsible for the management, operation and maintenance of the ZLT, and may alternatively indicate the process for the selection of the managing entity, and must in any case define its tasks and competences, revenues, if applicable, and coordination with other competent entities, in particular with regard to the monitoring of tests;<br>f) The conditions for access to the ZLT by the promoters, as well as for the performance of the tests, and for the cessation and suspension of them.
Portugal	2021	NA	Decreto-Lei n.º 67/2021, de 30 de julho	Decree-Law No. º 67/2021, of July 30	Chapter 1; Art 6(3). Each ZLT has an internal regulation, drawn up by the respective managing entity, subject to the opinion of the competent regulatory authority and the approval of the Test Authority
Portugal	2021	NA	Decreto-Lei n.º 67/2021, de 30 de julho	Decree-Law No. º 67/2021, of July 30	Chapter 1; Art 6(4). Any other conditions that are added by the constitutive act or by the regulation of each ZLT shall not call into question the final objective of promoting innovation and experimentation and testing activities.
Portugal	2021	NA	Decreto-Lei n.º 67/2021, de 30 de julho	Decree-Law No. º 67/2021, of July 30	Chapter 1; Art 6(6). The ZLT may provide for the creation of specific instruments of experimentation whenever the experimentation tests to be carried out, by their nature and specificity, so require, and may assume the modality of programs for innovation and, cumulatively or alternatively, and whenever the legal framework justifies it, integrate into the special ZLT model.
Portugal	2021	NA	Decreto-Lei n.º 67/2021, de 30 de julho	Decree-Law No. º 67/2021, of July 30	Chapter 1; Art 7. The conditions for access to the ZLT by the promoters, as well as for the performance of the tests, and for the cessation and suspension of them, contained in the constitutive act in accordance with paragraph 1(f) of the previous article, shall provide for:<br>a) the requirements that promoters must meet in order to access the ZLT, in particular with regard to establishment or representative in Portugal; technical, economic and financial capacity for the tests; compliance with tax and social security obligations; obtaining licenses and approvals that are applicable; and subscription of insurance contracts or the provision of guarantees required under the legislation applicable to the activity to be carried out;<br>b) The requirements that the tests must meet to access the ZLT, which, at least, are as follows:<br>i) The technology, product, service or process being tested must be innovative;

					ii) The tests must not call into question the safety of people, animals and property, and must properly safeguard health and environmental risks in compliance with applicable legislation;<br>iii) The technology, product, service or process must demonstrate potential for technical, economic or commercial feasibility, or interest in the pursuit of objectives of general interest or for the enrichment of technical or scientific knowledge;<br>c) the conditions for access to ZLT, either permanently or through specific programs for innovation;
Portuga l	2021	NA	Decreto-Lei n.º 67/2021, de 30 de julho	Decree-Law No. º 67/2021, of July 30	d) The conditions for submission, evaluation and selection of the tests to be carried out at the ZLT, which must include:<br>i) The information to be included in the application for access to the ZLT, which must include at least the identification of the promoter and the tests it intends to carry out, the area intended for the tests within the ZLT, the resources of the ZLT that the promoter requires for the tests, and the own resources that he allocates to them;<br>ii) The evaluation and selection criteria, which must include, at least, compliance with the requirements applicable in the ZLT and the conditions of refusal, such as lack of space, interference with other tests, non-compliance with the applicable requirements; obtaining, if necessary, opinions or authorizations from competent entities;<br>iii) The evaluation and selection process, with an indication of the applicable deadlines for this purpose;<br>e) The conclusion of a test protocol with indication of the conditions for its realization to be concluded between the managing entity of the ZLT and the promoter, which must indicate, at least, the parameters and objectives of the tests; start and duration; participants in the tests and potential impacts on third parties; conditions of review, renewal and cessation; conditions for the availability of the resources of the ZLT; and conditions for the use of the promoter's own resources;<br>f) The financial conditions for access to the ZLT, and fees may be provided for access to the ZLT, as well as financial counterparts for the availability of the own resources of the ZLT or partners;
Portuga l	2021	NA	Decreto-Lei n.º 67/2021, de 30 de julho	Decree-Law No. º 67/2021, of July 30	g) The conditions for carrying out the tests, which must necessarily include:<br>i) Compliance with the test protocol and applicable legislation;<br>ii) The monitoring and supervision by the managing entity;<br>iii) Monitoring and monitoring by the testing authority and supervision by regulatory authorities;<br>iv) The preparation of test reports by the promoter with information to be defined including the result of the tests, constraints identified and proposals to overcome or mitigate them; the possibility of sharing information (to the general public or with the managing entity and regulatory entities) with the safeguard of intellectual property, business secret and personal data, as well as the security of classified information, of any brand and degree, which is classified by competent entity and in accordance with the legal or regulatory provisions applicable to it;<br>h) The conditions for the suspension or cessation of the tests, which must necessarily include:<br>i) the course of the test period that has not been renewed;<br>ii) Non-compliance with the test protocol;<br>iii) Existence of safety, health and environmental risks, or other risks related to the sector in question;

					iv) In the case of tests that cross areas or sectors of activity subject to different legal or regulatory frameworks, the cessation or suspension of the tests may occur only in relation to the part of the tests of the technology, product, service or process relating to the area or sector whose legal or regulatory framework has been breached, or that presents risks;<br>v) the conditions for the removal of the resources brought by the promoter in case of cessation or suspension of the tests shall also be provided for in the constitutive act;<br>i) The criteria for the selection, evaluation and monitoring of tests.
Portuga l	2021	NA	Decreto-Lei n.º 67/2021, de 30 de julho	Decree-Law No. º 67/2021, of July 30	Chapter 1; Art 13(1). Without prejudice to other insurances whose contracting is legally mandatory, promoters must have adequate civil liability insurance to cover any damages arising from the performance of tests under this decree-law.
Portuga l	2023	NA	Portaria n.º 165/2023, de 21 de junho	Ordinance No. º 165/2023 of June 21	Art 1(1). The Technological Free Zone "ZLT Matosinhos" is created, proposed by the CEiiA Association - Center for Engineering and Development, with a view to carrying out tests, experimentation and demonstration of innovative technologies, products, services and business models, in a real or almost real environment, in the area of mobility oriented to the carbon neutrality of cities.
Portuga l	2023	NA	Portaria n.º 165/2023, de 21 de junho	Ordinance No. º 165/2023 of June 21	Art 1(2). 2 - The "ZLT Matosinhos" is geographically delimited in the municipality of Matosinhos.
Portuga l	2023	NA	Portaria n.º 165/2023, de 21 de junho	Ordinance No. º 165/2023 of June 21	Annex - Chapter 1; Art 1(1). The Technological Free Zone of Matosinhos, hereinafter referred to as "ZLT de Matosinhos", or simply "ZLT", is a geographically delimited space in the municipality of Matosinhos for the realization of tests, experimentation and demonstration of innovative technologies, products, services and business models, in a real or quasi-real environment, in the area of mobility oriented to the carbon neutrality of cities.
Portuga l	2023	NA	Portaria n.º 165/2023, de 21 de junho	Ordinance No. º 165/2023 of June 21	Annex - Chapter 1; Art 1(2). In a complementary way, ZLT also intends to test new policy concepts, forms of governance, financing systems and social innovations that accelerate the placing of new products and services on the market.
Portuga l	2023	NA	Portaria n.º 165/2023, de 21 de junho	Ordinance No. º 165/2023 of June 21	Annex - Chapter 1; Art 3. The ZLT of Matosinhos has as specific objectives:<br>a) Evaluate the performance of new products and services, with a view to identifying needs for improvements, optimizations and technical and functional changes;<br>b) Analyze the feasibility of implementing new business models, policy concepts, governance models and financing systems related to new products and services;<br>c) Stimulate the appropriation and social adoption of technologies, products and services by users, using co-creation and open innovation methodologies;<br>d) Evaluate issues of security, privacy, data protection, ethics, among others relevant to the testing of innovative products and services;<br>e) Evaluate the impacts of new technologies, products and services on the environment, the economy and society, in particular in terms of carbon emissions.

Portugal	2023	NA	Portaria n.º 165/2023, de 21 de junho	Ordinance No. º 165/2023 of June 21	Annex - Chapter 1; Art 4. The existence of the ZLT of Matosinhos allows access to:<br>a) Physical spaces for testing and experimentation;<br>b) Virtual testing and experimentation environments;<br>c) Specific infrastructures and equipment;<br>d) Data, data science platforms and connectivity;<br>e) Technical and technological support and specialized services;<br>f) Involvement of test groups and communities;<br>g) Possibility of integrating partners with complementary resources and services;<br>h) Regulatory framework adapted with the involvement of the competent regulatory authorities.
Portugal	2023	NA	Portaria n.º 165/2023, de 21 de junho	Ordinance No. º 165/2023 of June 21	Annex - Chapter 1; Art 6(1). The area of intervention of the ZLT of Matosinhos is mobility oriented to carbon neutrality in cities.
Portugal	2023	NA	Portaria n.º 165/2023, de 21 de junho	Ordinance No. º 165/2023 of June 21	Annex - Chapter 1; Art 6(2). In particular, it is intended to test cyberphysical carbon-zero mobility products, designed to be used as services, which integrate three essential dimensions: physical mobility products (and energy systems), infrastructure and connectivity devices and data science platforms.
Portugal	2023	NA	Portaria n.º 165/2023, de 21 de junho	Ordinance No. º 165/2023 of June 21	Annex - Chapter 1; Art 6(3). The technologies associated with these products typically focus on the development of cyberphysical systems, such as data analytics, artificial intelligence, sensing and IoT, charging technologies, short-range vehicular communications (V2V and/or V2I/I2V) (ITS G5) and long-range and state-of-the-art mobile communications (5G and following), blockchain, among others.
Portugal	2018	NA	Lei n.º 46/2018, de 13 de agosto	Law No.º 46/2018 of August 13	Chapter 1; Art 1. This law establishes the legal framework for the security of cyberspace.
Portugal	2018	NA	Lei n.º 46/2018, de 13 de agosto	Law No.º 46/2018 of August 13	Chapter 1; Art 2(3). This law applies to digital service providers who have their main establishment in national territory or, without having it, appoint a representative established in national territory, provided that they provide digital services there.
Portugal	2018	NA	Lei n.º 46/2018, de 13 de agosto	Law No.º 46/2018 of August 13	Chapter 2; Art 11. Digital service providers provide the following services:<br>a) Online market service;<br>b) Online search engine service;<br>c) Cloud computing service.
Portugal	2018	NA	Lei n.º 46/2018, de 13 de agosto	Law No.º 46/2018 of August 13	Chapter 3; Art 18(1). Digital service providers identify and take the appropriate and proportionate technical and organizational measures to manage the risks posed to the security of the networks and information systems they use in the context of the offer of digital services.
Portugal	2018	NA	Lei n.º 46/2018, de 13 de agosto	Law No.º 46/2018 of August 13	Chapter 3; Art 18(2). The measures referred to in the previous paragraph shall ensure a level of security of networks and information systems appropriate to the risk in question, taking into account the latest technical progress, and shall take into account the following factors:<br>a) The safety of systems and installations;<br>b) The treatment of incidents;<br>c) The management of the continuity of activities;<br>d) The monitoring, audit and tests carried out;<br>e) Compliance with international standards.

Portugal	2018	NA	Lei n.º 46/2018, de 13 de agosto	Law No.º 46/2018 of August 13	Chapter 3; Art 18(3). Digital service providers take measures to avoid incidents that affect the security of their networks and information systems and to minimize their impact on digital services, in order to ensure the continuity of these services.
Sweden	2020		Lag (2020:421) om Rättsmedicinalverkets behandling av personuppgifter	Law (2020: 421) on the Swedish Medicines Agency's processing of personal data	Chapter 2; Art 2. The personal data may also be processed for other purposes, provided that the data is not processed in a manner that is incompatible with the purpose for which the data was collected in.
Sweden	2020		Lag (2020:421) om Rättsmedicinalverkets behandling av personuppgifter	Law (2020: 421) on the Swedish Medicines Agency's processing of personal data	Chapter 2; Art 5. Access to personal data shall be limited to that which everyone needs to be able to fulfill theirs tasks.
Sweden	2022		Lag (2022:818) om den offentliga sektorns tillgängliggörande av data	Law (2022: 818) on the public sector's availability of data	Chapter 1; Art 1. This law aims to promote the public sector making data available for re-use, especially in the form of open data, provided that requirements information security and protection of personal data can is maintained and that it does not pose risks to Sweden security.
Sweden	2022		Lag (2022:818) om den offentliga sektorns tillgängliggörande av data	Law (2022: 818) on the public sector's availability of data	Chapter 2; Art 1. Data shall be made available for reuse in accordance with with the requirements set out in this chapter. availability shall be made to the extent that information security requirements and protection of personal data can be maintained and maintained provided that it does not entail risks for Sweden's security.
Sweden	2022		Lag (2022:818) om den offentliga sektorns tillgängliggörande av data	Law (2022: 818) on the public sector's availability of data	Chapter 2; Art 3. A valuable amount of data should be made available in one machine-readable format through a suitable interface or through bulk charge.
Sweden	2022		Lag (2022:818) om den offentliga sektorns tillgängliggörande av data	Law (2022: 818) on the public sector's availability of data	Chapter 2; Art 5. An authority or public company may ask conditions for making data available for re-use only if it is justified by a public interest. A condition shall be objective, proportionate and non-discriminatory for comparable types of re-use. A condition may not restrict competition or unnecessarily restrict opportunities to re-use data.
Sweden	2014		Patientlag	Patient Law (2014:821)	Chapter 3; Art 2(1). The patient should also receive information about the ability to choose treatment options and care providers and providers of publicly funded health care
Sweden	2014		Patientlag	Patient Law (2014:821)	Chapter 3; Art 6. The information must be adapted to the recipient's age, maturity, experience, linguistic background and other individual conditions.
Sweden	2014		Patientlag	Patient Law (2014:821)	Chapter 5; Art 3. A patient's involvement in health care through self-care according to the Act (2022: 1250) on self-care shall be based on the patient's wishes and individual conditions.
Sweden	2022		Lag (2022:1250) om egenvård	Self Care Act (2022:1250)	Art 2. For the purposes of this Act, self-care refers to health and medical measure such as treatment in a situation where a healthcare professional has assessed that a patient can perform himself or with the help of someone else
Sweden	2022		Lag (2022:1250) om egenvård	Self Care Act (2022:1250)	Art 3. For the purposes of this Act, a health care measure means a measure to medically prevent, investigate or treat diseases or damage.
Sweden	2022		Lag (2022:1250) om egenvård	Self Care Act (2022:1250)	Art 4. For the purposes of this Act, health care professionals refer to the one who has a credential for a profession in health care or which, according to special ordinance, has received equivalent permission.

Sweden	2022		Lag (2022:482) om elektronisk kommunikation	Electronic Communications Act (2022: 482)	Chapter 1; Art 7. public electronic communications network: an electronic communication networks used wholly or mainly to provide publicly available electronic communication services and supporting information transfer between network connection points,
Sweden	2022		Lag (2022:482) om elektronisk kommunikation	Electronic Communications Act (2022: 482)	Chapter 8; Art 1. The provider of a general electronic communication network or a publicly available electronic communication service shall take appropriate and proportionate technical and organizational measures to appropriately manage risks that threaten the security of networks and services.<br><br>The measures shall ensure a level of security in networks and services that are appropriate in relation to the risks. measures should be taken specifically to prevent and minimize the impact of security incidents on users and on other networks and services.
Sweden	2022		Lag (2022:482) om elektronisk kommunikation	Electronic Communications Act (2022: 482)	Chapter 9; Art 1. The provider of a general electronic communication network or a publicly available electronic<br>communication service should erase or unidentify traffic data that has been stored or processed on something<br>another way when they are no longer needed to transfer one electronic message. This applies provided that<br>the information refers to users who are natural persons or subscribers.
Sweden	2022		Lag (2022:482) om elektronisk kommunikation	Electronic Communications Act (2022: 482)	Chapter 9; Art 2. The provider of a publicly available electronic communication service may, with the consent of the person the information relates to, process the traffic data referred to in Chapter 9; Art 1 to market electronic communications services or to provide other services where the information is needed. Treatment may only be done to the extent and during that time necessary for marketing or service. One's consent may be revoked at any time. The provider of a publicly available electronic communication service shall inform the person concerned of the task:<br>1. the type of traffic data being processed; and<br>2. how long the data is processed for such purposes as are stated in the first and second paragraphs.
Sweden	2021		Lag (2021:600) med kompletterande bestämmelser till EU's förordningar om medicintekniska produkter	Law (2021: 600) with supplementary provisions to EU regulations on medical devices	Chapter 1; Art 3. In the case of products intended for consumers or which can be assumed to be used by consumers also applies Product Safety Act (2004: 451).
Sweden	2004		Produktsäkerhetslag	Product Safety Act (2004:451)	Art 1. This law aims to ensure that goods and services provided to consumers do not cause harm person.
Sweden	2004		Produktsäkerhetslag	Product Safety Act (2004:451)	Art 2. The law applies to goods and services that provided in business activities and goods provided in public operations. A prerequisite is that the product or the service is intended for consumers or may be assumed to used by consumers.
Sweden	2004		Produktsäkerhetslag	Product Safety Act (2004:451)	Art 8. A product or service is safe, if it is normal or reasonably foreseeable use and service life do not bring any risk to human health and safety or just a low risk. However, this risk must be acceptable considering how the product or service is used and must be compatible with a high level of protection of human health and safety.

Sweden	2004		Produktsäkerhetslag	Product Safety Act (2004:451)	Art 9. In assessing whether a risk of a product should be considered as acceptable and compatible with a high level of protection shall be taken into account especially added:<br>1. the characteristics of a product, such as its composition and packaging and instructions for assembly, installation and maintenance,<br>2. other information provided about the product by labeling, warnings, operating instructions, instructions for its disposal or by any other means,<br>3. the effect of the goods on other goods, if it can be assumed that will be used with such,<br>4. risks that the product may entail for some consumer groups, especially children and the elderly.
Sweden	2004		Produktsäkerhetslag	Product Safety Act (2004:451)	Art 10. In assessing whether a risk from a service should be considered which is acceptable and compatible with a high level of protection shall be taken into account especially added:<br>1. how the service is performed,<br>2. security information provided by the trader,<br>3. the impact of the service on goods, if it can be assumed that the security in goods will be affected by how the service is performed, and<br>4. risks that the service may entail for some consumer groups, especially children and the elderly.
Sweden	2004		Produktsäkerhetslag	Product Safety Act (2004:451)	Art 12. When, in cases other than those referred to in section 11, one must assess one be or service is safe should be taken into account:<br>1. Swedish standards other than those referred to in section 11 §,<br>2. European Commission recommendations with guidelines for the assessment of the security of goods or services,<br>3. good practice for product safety in the industry concerned,<br>4. the current level of scientific and technical knowledge; and<br>5. the level of protection that consumers can reasonably expect.
Sweden	2018		Lag (2018:1174) om informationssäkerhet för samhällsviktiga och digitala tjänster	Information Security Act (2018:1174)	Art 1. The purpose of this law is to achieve a high level of security for networks and information systems pertaining to healthcare, digital infrastructure, and digital services.
Sweden	2018		Lag (2018:1174) om informationssäkerhet för samhällsviktiga och digitala tjänster	Information Security Act (2018:1174)	Art 2(4). "digital service" means any service normally provided for remuneration, at a distance, by electronic means and at the individual request of a recipient of services, and which constitute an internet-based marketplace, internet-based search engine or cloud service.
Sweden	2018		Lag (2018:1174) om informationssäkerhet för samhällsviktiga och digitala tjänster	Information Security Act (2018:1174)	Ar 2(6). Internet-based search engine: a service that enables users to search on virtually all websites or websites in a particular language through a request for any topic in the form of a keyword, phrase, or any other input, and which returns links that contains information about the requested content
Sweden	2018		Lag (2018:1174) om informationssäkerhet för samhällsviktiga och digitala tjänster	Information Security Act (2018:1174)	Art 3(2). This law applies to legal entities providing a digital service and which has its main place of establishment in Sweden or has appointed a representative established here (digital service providers).

Sweden	2018		Lag (2018:1174) om informationssäkerhet för samhällsviktiga och digitala tjänster	Information Security Act (2018:1174)	Art 10. A legal entity offering digital services in Sweden but which does not have its main place of establishment<br>within the European Union, nor has it appointed one representatives established in a Member State where the services offered, shall appoint one such representatives.
Sweden	2018		Lag (2018:1174) om informationssäkerhet för samhällsviktiga och digitala tjänster	Information Security Act (2018:1174)	Art 15. Digital service providers should take the technical and organizational measures they consider appropriate and proportionate to manage risks such as threatening the security of networks and information systems when providing digital services within European Union. The measures shall ensure a level of the security of the networks and the information systems that are appropriate in relation to the risk.
Sweden	2018		Lag (2018:1174) om informationssäkerhet för samhällsviktiga och digitala tjänster	Information Security Act (2018:1174)	Art 16. Digital service providers should take action to prevent and minimize the effects of incidents such as affects networks and information systems they use. The obligation applies only in relation to effects such as such incidents have on digital services as the supplier offers within the European Union. The measures shall aim to to ensure the continuity of services.
Sweden	2017	2019	Förvaltningslagen (2017:900)	The Administrative Procedure Act (2017:900)	§1 This Act applies to the handling of cases by the administrative<br>authorities and the handling of administrative cases by the courts.<br>§28 A decision can be taken by one executive alone or by several jointly or automatically. In the final processing, the presenter and other executives can participate without participating in the decision.
Sweden	2017	2023	Kommunallagen (2017:725)	Municipal Act (2017:725)	§ 37 A committee may delegate decision-making rights to the presidium, a committee, a member, a substitute or an automated decision-making function in a certain case or a certain group of cases. A committee may also assign an employee to decide according to ch. 7. Sections 5-8. Law (2022:638) .
Sweden	2008	2023	Patientdatalagen (2008:355)	Patient Data Law (2008:355)	§ 1 This Act is applied to care providers' processing of personal data in health care. The law also contains provisions on the obligation to keep patient records.<br>The law also applies in applicable parts to information about deceased persons.<br>The law does not apply to such processing of personal data as referred to in the original wording of Article 2.2 di Regulation (EU) 2016/679 of the European Parliament and of the Council of 27 April 2016 on the protection of natural persons with regard to the processing of personal data and on the free flow of such data and on the repeal of Directive 95/46/EC (General Data Protection Regulation), here referred to as the EU Data Protection Regulation. Law (2018:447) .<br><br>§ 2 Information management in health care must be organized so that it caters for patient safety and good quality and promotes cost-effectiveness.<br>Personal data must be designed and otherwise processed so that the privacy of patients and other data subjects is respected.<br>Documented personal data must be handled and stored so that unauthorized persons do not gain access to them.<br><br>§ 4 This law contains provisions that supplement the EU's data protection regulation, in the case of such processing of personal data in healthcare that is fully or partially automated or where the data is part of or is intended to be part of a structured collection of personal data that is available for search or compilation according to specific criteria.

Sweden	2008	2023	Diskrimineringslagen	The Discrimination Act	§ 1 The purpose of this law is to counteract discrimination and in other ways to promote equal rights and opportunities regardless of gender,<br>cross-gender identity or expression, ethnic affiliation, religion or other belief, disability, sexual orientation or age.<br><br>§13 Discrimination is prohibited with regard to 1. health care and other medical activities, 2. activities within the social service, and 3. support in the form of travel service and national travel service and housing adjustment allowance. Law (2012:673) .<br><br>Chapter 6 - the rial<br>§ 3 If the person who considers himself to have been discriminated against or subjected to reprisals shows circumstances that give reason<br>to assume that he or she has been discriminated against or subjected to reprisals, it is the defendant who must show that discrimination or<br>reprisals have not occurred.
Sweden	2010	2023	Patientsäkerhetslagen	The Patient Safety Act (2010:659)	Chapter 3 - the healthcare provider's obligations to carry out systematic patient safety work<br>§ 1 The care provider must plan, lead and control the operation in a way that leads to the requirement for good care in the Health and Medical Care Act (2017:30) and the Dental Care Act (1985:125) being maintained. Law (2017:62) .<br><br>chapter 6 - obligations for health and medical care personell, etc<br>§ 1 The health care staff must perform their work in accordance with science and proven experience. A patient must be given expert and caring healthcare that meets these requirements. As far as possible, care must be designed and implemented in consultation with the patient. The patient must be shown care and respect.<br><br>§3 The person who belongs to the health care staff may delegate a work task to someone else only when it is compatible with the requirement for good and safe care.<br>Whoever delegates a work task to someone else is responsible for ensuring that this person has the conditions to complete the task.
Sweden	2011	2023	SOSFS 2011:9 Socialstyrelsens föreskrifter och allmänna råd om ledningssystem för systematiskt kvalitetsarbete	SOSFS 2011:9 The National Board of Health and Welfare's regulations and general advice on management systems for systematic quality work	Chapter 1 - area of application<br>§ 1 These regulations shall be applied in the work of systematically and continuously developing and securing the quality of such activities<br>covered by 1. 5 ch. § 4 of the Health and Medical Care Act (2017:30), 2. § 16 of the Dental Care Act (1985:125), 3. § 6 of the Act (1993:387) on support and services for certain disabled persons, LSS, and 4. 3 ch. Section 3, fourth paragraph of the Social Services Act (2001:453). (HSLF-FS 2023:27)<br>§ 2 The regulations must also be applied in the systematic patient safety work that healthcare providers must conduct according to ch. 3. the Patient Safety Act (2010:659).<br><br>Chapter 5 - systematic improvement work<br>Risk analysis

					§ 1 The care provider or the person who runs social services or activities according to LSS must continuously assess whether there is a risk that events could occur that could lead to deficiencies in the quality of the activity.<br>For each such event, the care provider or the person who runs social services or activities according to LSS<br>1. estimate the probability of the event occurring, and<br>2. assess the negative consequences that could result from the event.
Sweden	2017	2024	Hälso och sjukvårdslagen (2017:30)	The Health and Medical Care Act	1 content and scope<br>§ 1 This Act contains provisions on how health care activities are to be organized and conducted. The law applies to all healthcare providers as well as regions and municipalities as principals<br><br>3 Generally<br>§ 1 The goal of health care is good health and care on equal terms for the entire population.<br>Care must be given with respect for the equal value of all people and for the dignity of the individual person. Those who have the greatest need for health care must be given priority for care.<br>§ 2 The health care service must work to prevent ill health.<br><br>5 the business<br>§ 1 Health and medical care activities must be conducted in such a way that the requirements for good care are met. This means that the care in particular must<br>1. be of good quality with a good hygienic standard,<br>2. meet the patient's need for security, continuity and safety,<br>3. build on respect for the patient's self-determination and integrity,<br>4. promote good contacts between the patient and health - and the healthcare staff, and<br>5. be easily accessible
Sweden	2018		Lagen om informationssäkerhet (2018:1174)	The Act on information security for socially important and digital services (2018:1174)	1. The purpose of this law is to achieve a high level of security in networks and information systems for 1) socially important services within the sectors - energy, transport, banking, financial market infrastcrure, healthcare, delivery and distribution of drinking water, digital infrastructure, and 2. digital services

Sweden	2008	2019?	SOSFS 2008:1 Socialstyrelsens föreskrifter om användning av medicintekniska produkter i hälso- och sjukvården	SOSFS 2008:1 The National Board of Health and Welfare's regulations on the use of medical devices in healthcare	Chapter 1 - Area of application<br><br>§ 1 These regulations shall be applied in activities covered by the Health and Medical Services Act (2017:30) and the Dental Services Act (1985:125) when use of medical devices on patients or when analyzing samples from patients, prescription and dispensing of medical devices to patients, supply of medical devices to patients, and reporting of negative events and incidents. (HSLF-FS 2017:27)<br><br>§ 2 The scope includes information systems connected to medical devices, special authority to prescribe consumables within the framework of the pharmaceutical benefits, and handling of medical devices, e.g. maintenance and transport.<br><br>Chapter 3 - management systems and routines<br><br>§6 The business manager shall, according to assignment, be responsible for only safe and medically appropriate medical devices and, to these, connected information systems are used on patients,only safe and medically appropriate medical technical products are prescribed, dispensed or supplied to patients,the medical devices and the information systems connected to them are checked and correctly installed before they are used on patients,information from manufacturers and authorities about the medical devices is available to the healthcare staff and other concerned staff,the routines within the framework of the management system are available to the healthcare staff and to other affected staff, andmedical devices that have been prescribed, dispensed or supplied to patients can be traced.<br><br>§ 7 The operations manager must, after assignment, make an assessment of whether the training of the health care staff and other relevant staff is adequate against the background of the competence required to be responsible for education,prescribing and dispensing medical devices to patients,produce written instructions for specially adapted products, andbe responsible for notification, which means making notifications according to ch. 5 regarding negative events and accidents with medical devices.<br>The business manager must also, according to the assignment, appoint and list who or which of the health care staff or other relevant staff will fulfill the above tasks. (HSLF-FS 2021:43)
UK	2019	2020	The Data Protection, Privacy and Electronic Communications (Amendments etc) (EU Exit) Regulations 2019	The Data Protection, Privacy and Electronic Communications (Amendments etc) (EU Exit) Regulations 2019	“the 2018 Act” means the Data Protection Act 2018;<br>“the UK GDPR” means Regulation (EU) 2016/679 of the European Parliament and of the Council of 27th April 2016 on the protection of natural persons with regard to the processing of personal data and on the free movement of such data (General Data Protection Regulation) as it forms part of the law of England and Wales, Scotland and Northern Ireland by virtue of section 3 of the European Union (Withdrawal) Act 2018.<br><br>Amendment of the UK GDPR<br>3. Schedule 1 amends the UK GDPR.<br><br>Amendment of the Data Protection Act 2018<br>4. Schedule 2 amends the 2018 Act.

UK	2023		The Data Protection (Adequacy) (United States of America) Regulations 2023	The Data Protection (Adequacy) (United States of America) Regulations 2023	Explanatory Note<br>(This note is not part of the Regulations)<br>These Regulations specify the United States of America as a country which provides an adequate level of protection of personal data for certain transfers for the purposes of Part 2 of the Data Protection Act 2018 (“the 2018 Act”) and the UK GDPR (defined in section 3 of the 2018 Act). This means that personal data which will be in the scope of the EU-US Data Privacy Framework Principles can be transferred to persons in the United States of America who participate in the UK Extension to the EU-US Data Privacy Framework without the need for any specific authorisation. “Personal data” is defined in Article 4(1) of the UK GDPR and has the same meaning in Part 2 of the 2018 Act by virtue of section 5 of that Act.<br><br>Adequate level of protection<br>3.—(1) For the purposes of Part 2 of the Data Protection Act 2018(1) and the UK GDPR(2), the Secretary of State specifies the United States of America as ensuring an adequate level of protection of personal data(3) for a transfer described in paragraph (2).<br>(2) A transfer described by this paragraph is a transfer of personal data which—<br>(a)is to a person in the United States of America who is indicated on the Data Privacy Framework List as participating in the UK Extension to the EU-US Data Privacy Framework; and<br>(b)will be subject to the EU-US Data Privacy Framework Principles on receipt by that person.
UK	2015		The Re-use of Public Sector Information Regulations 2015	The Re-use of Public Sector Information Regulations 2015 (2015 No. 1415)	re-use of documents<br><br>4.—(1) Subject to paragraph (2), re-use means the use by a person of a document held by a public sector body for a purpose other than the initial purpose within that public sector body's public task for which the document was produced.<br>(2) Re-use shall not include—<br>(a)the transfer for use of a document within a public sector body for the purpose of carrying out its own public task; or<br>(b)the transfer for use of a document from one public sector body to another for the purpose of either public sector body carrying out its public task.

UK	2023	2024	Online Safety Act 2023	Online Safety Act 2023	1 Introduction<br>(1)This Act provides for a new regulatory framework which has the general purpose of making the use of internet services regulated by this Act safer for individuals in the United Kingdom.<br>(2)To achieve that purpose, this Act (among other things)—<br>(a)imposes duties which, in broad terms, require providers of services regulated by this Act to identify, mitigate and manage the risks of harm (including risks which particularly affect individuals with a certain characteristic) from—<br>(i)illegal content and activity, and<br>(ii)content and activity that is harmful to children, and<br>(b)confers new functions and powers on the regulator, OFCOM.<br>(3)Duties imposed on providers by this Act seek to secure (among other things) that services regulated by this Act are—<br>(a)safe by design, and<br>(b)designed and operated in such a way that—<br>(i)a higher standard of protection is provided for children than for adults,<br>(ii)users' rights to freedom of expression and privacy are protected, and<br>(iii)transparency and accountability are provided in relation to those services.<br><br>9 Illegal content risk assessment duties<br>(1) This section sets out the duties about risk assessments which apply in relation to all regulated user-to-user services.<br>(2) A duty to carry out a suitable and sufficient illegal content risk assessment at a time set out in, or as provided by, Schedule 3.<br>(3) A duty to take appropriate steps to keep an illegal content risk assessment up to date, including when OFCOM make any significant change to a risk profile that relates to services of the kind in question.<br><br>22 Duties about freedom of expression and privacy (relates to providers of user-to-user services)<br>(1) This section sets out the duties about freedom of expression and privacy which apply in relation to regulated user-to-user services (as indicated by the headings).<br>All services<br>(2) When deciding on, and implementing, safety measures and policies, a duty to have particular regard to the importance of protecting users' right to freedom of expression within the law.<br>(3) When deciding on, and implementing, safety measures and policies, a duty to have particular regard to the importance of protecting users from a breach of any statutory provision or rule of law concerning privacy that is relevant to the use or operation of a user-to-user service (including, but not limited to, any such provision or rule<br>concerning the processing of personal data).<br><br>33 Duties about freedom of expression and privacy (relates to providers of search services)<br><br>"personal data" has the meaning given by section 3(2) of the Data Protection Act 2018<br>"OFCOM" means the Office of Communications;

					“processing” has the meaning given by section 3(4) of the Data Protection Act 2018

UK	2018	2023	The Network and Information Systems Regulations 2018	The Network and Information Systems Regulations 2018	EXPLANATORY NOTE (This note is not part of the Regulations)<br>These Regulations implement Directive (EU) 2016/1148 of the European Parliament and of the Council concerning measures for a high common level of security of network and information systems across the Union (OJ No L194, 19.7.2016, p1).<br><br>PART 2 The National Framework<br>The NIS national strategy<br>2.—(1) A Minister of the Crown must designate and publish a strategy to provide strategic objectives and priorities on the security of network and information systems in the United Kingdom (“the NIS national strategy”).<br>(2) The strategic objectives and priorities set out in the NIS national strategy must be aimed at achieving and maintaining a high level of security of network and information systems in—<br>(a) the sectors specified in column 1 of the table in Schedule 1 (“the relevant sectors”); and<br>(b) digital services.<br>(3) The NIS national strategy may be published in such form and manner as the Minister considers appropriate.<br>(4) The NIS national strategy may be reviewed by the Minister at any time and, if it is revised following such a review, the Minister must designate and publish a revised NIS national strategy as soon as reasonably practicable following that review.<br>(5) The NIS national strategy must, in particular, address the following matters—<br>(a) the regulatory measures and enforcement framework to secure the objectives and priorities of the strategy;<br>(b) the roles and responsibilities of the key persons responsible for implementing the strategy;<br>(c) the measures relating to preparedness, response and recovery, including cooperation between public and private sectors;<br>(d) education, awareness-raising and training programmes relating to the strategy;<br>(e) research and development plans relating to the strategy;<br>(f) a risk assessment plan identifying any risks; and<br>(g) a list of the persons involved in the implementation of the strategy.<br><br>Designation of national competent authorities<br>3.—(1) The person specified in column 3 of the table in Schedule 1 is designated as the competent authority, for the territorial jurisdiction indicated in that column, and for the subsector specified in column 2 of that table (“the designated competent authorities”).<br>(2) The Information Commissioner is designated as the competent authority for the United Kingdom for RDSPs.<br>(3) In relation to the subsector for which it is designated under paragraph (1), the competent authority must—<br>(a) review the application of these Regulations;<br>(b) prepare and publish guidance;<br>(c) keep a list of all the operators of essential services who are designated, or deemed to be designated, under regulation 8 F12...;

					(d) keep a list of all the revocations made under regulation 9;<br>(e) send a copy of the lists mentioned in sub-paragraphs (c) and (d) to GCHQ, as the<br>SPOC designated under regulation 4, to enable it to prepare the report mentioned in regulation 4(3);<br>(f) consult and co-operate with the Information Commissioner when addressing incidents that result in breaches of personal data; and<br>(g) in order to fulfil the requirements of these Regulations, consult and co-operate with—<br>(i) relevant law-enforcement authorities;<br>F13(ii) . . . .<br>(iii) other competent authorities in the United Kingdom;<br>(iv) the SPOC that is designated under regulation 4; and<br>(v) the CSIRT that is designated under regulation 5.

UK	2018	2024	Data Protection Act 2018	Data Protection Act 2018	2 protection of personal data<br>(1)The [UK GDPR, The Data Protection, Privacy and Electronic Communications (Amendments etc) (EU Exit) Regulations 2019 (S.I. 2019/419)] and this Act protect individuals with regard to the processing of personal data, in particular by—<br>(a)requiring personal data to be processed lawfully and fairly, on the basis of the data subject's consent or another specified basis,<br>(b)conferring rights on the data subject to obtain information about the processing of personal data and to require inaccurate personal data to be rectified, and<br>(c)conferring functions on the Commissioner, giving the holder of that office responsibility for monitoring and enforcing their provisions.<br>(2)When carrying out functions under the [F5UK GDPR] and this Act, the Commissioner must have regard to the importance of securing an appropriate level of protection for personal data, taking account of the interests of data subjects, controllers and others and matters of general public interest.<br><br>8 Lawfulness of processing: public interest etc<br>In Article 6(1) of the [UK GDPR] (lawfulness of processing), the reference in point (e) to processing of personal data that is necessary for the performance of a task carried out in the public interest or in the exercise of the controller's official authority includes processing of personal data that is necessary for—<br>(a)the administration of justice,<br>(b)the exercise of a function of either House of Parliament,<br>(c)the exercise of a function conferred on a person by an enactment or rule of law,<br>(d)the exercise of a function of the Crown, a Minister of the Crown or a government department, or<br>(e)an activity that supports or promotes democratic engagement.<br><br>18 Transfers of personal data to third countries etc<br>(1) The Secretary of State may by regulations specify, for the purposes of Article 49(1)(d) of the [F101UK GDPR]— (a) circumstances in which a transfer of personal data to a third country or international organisation is to be taken to be necessary for important reasons of public interest, and (b) circumstances in which a transfer of personal data to a third country or international organisation which is not required by an enactment is not to be taken to be necessary for important reasons of public interest.<br>(2) The Secretary of State may by regulations restrict the transfer of a category of personal data to a third country or international organisation where— [F102(a) the transfer cannot take place based on adequacy regulations (see section 17A),] and (b) the Secretary of State considers the restriction to be necessary for important reasons of public interest.<br>(3) Regulations under this section— (a) are subject to the made affirmative resolution procedure where the Secretary of State has made an urgency statement in respect of them; (b) are otherwise subject to the affirmative resolution procedure.<br>(4) For the purposes of this section, an urgency statement is a reasoned statement that the Secretary of State considers it desirable for the regulations to come into force without delay<br><br>28 National security and defence: modifications to Articles 9 and 32 of the [F147UKGDPR]

					(1) Article 9(1) of [F148the UK GDPR] (prohibition on processing of special categories of personal data) does not prohibit the processing of personal data to which [F149the UK GDPR] applies to the extent that the processing is carried out—<br>(a) for the purpose of safeguarding national security or for defence purposes, and (b) with appropriate safeguards for the rights and freedoms of data subjects.<br>(2) Article 32 of [F150the UK GDPR] (security of processing) does not apply to a controller or processor to the extent that the controller or the processor (as the case may be) is processing personal data to which [F151the UK GDPR] applies for— (a) the purpose of safeguarding national security, or (b) defence purposes.<br>(3) Where Article 32 of [F152the UK GDPR] does not apply, the controller or the processor must implement security measures appropriate to the risks arising from the processing of the personal data.<br>(4) For the purposes of subsection (3), where the processing of personal data is carried out wholly or partly by automated means, the controller or the processor must, following an evaluation of the risks, implement measures designed to—<br>(a) prevent unauthorised processing or unauthorised interference with the systems used in connection with the processing,<br>(b) ensure that it is possible to establish the precise details of any processing that takes place,<br>(c) ensure that any systems used in connection with the processing function properly and may, in the case of interruption, be restored, and<br>(d) ensure that stored personal data cannot be corrupted if a system used in connection with the processing malfunctions<br><br>49 Right not to be subject to automated decision-making<br>(1) A controller may not take a significant decision based solely on automated processing unless that decision is required or authorised by law.<br>(2) A decision is a “significant decision” for the purpose of this section if, in relation to a data subject, it—(a) produces an adverse legal effect concerning the data subject, or (b) significantly affects the data subject.<br><br>57 Data protection by design and default<br>(1) Each controller must implement appropriate technical and organisational measures which are designed—<br>(a) to implement the data protection principles in an effective manner, and<br>(b) to integrate into the processing itself the safeguards necessary for that purpose.<br>(2) The duty under subsection (1) applies both at the time of the determination of the means of processing the data and at the time of the processing itself.<br>(3) Each controller must implement appropriate technical and organisational measures for ensuring that, by default, only personal data which is necessary for each specific purpose of the processing is processed.<br>(4) The duty under subsection (3) applies to—<br>(a) the amount of personal data collected,<br>(b) the extent of its processing,<br>(c) the period of its storage, and

					(d) its accessibility.<br><br>64 Data protection impact assessment<br>(1) Where a type of processing is likely to result in a high risk to the rights and freedoms of individuals, the controller must, prior to the processing, carry out a data protection impact assessment.<br>(2) A data protection impact assessment is an assessment of the impact of the envisaged processing operations on the protection of personal data.<br><br>66 Security of processing<br>(1) Each controller and each processor must implement appropriate technical and organisational measures to ensure a level of security appropriate to the risks arising from the processing of personal data.<br><br>CHAPTER 5 TRANSFERS OF PERSONAL DATA OUTSIDE THE UNITED KINGDOM<br>109 Transfers of personal data outside the United Kingdom<br>(1) A controller may not transfer personal data to—<br>(a) a country or territory outside the United Kingdom, or<br>(b) an international organisation,unless the transfer falls within subsection (2).<br>(2) A transfer of personal data falls within this subsection if the transfer is a necessary and proportionate measure carried out—<br>(a) for the purposes of the controller's statutory functions, or<br>(b) for other purposes provided for, in relation to the controller, in section 2(2)<br>(a) of the Security Service Act 1989 or section 2(2)(a) or 4(2)(a) of the Intelligence Services Act 1994.<br><br>schedules<br>1 Employment, social security and social protection<br>(1) This condition is met if—(a) the processing is necessary for the purposes of performing or exercising obligations or rights which are imposed or conferred by law on the controller or the data subject in connection with employment, social security or social protection, and (b) when the processing is carried out, the controller has an appropriate policy document in place (see paragraph 39 in part 4 of this schedule)<br><br>2 Health or social care purposes<br>(1) This condition is met if the processing is necessary for health or social care purposes.<br><br>3 Public health<br>This condition is met if the processing—<br>(a) is necessary for reasons of public interest in the area of public health, and<br>(b) is carried out—<br>(i) by or under the responsibility of a health professional, or<br>(ii) by another person who in the circumstances owes a duty of confidentiality under an enactment or rule of law.

					Research<br>This condition is met if the processing—<br>(a) is necessary for archiving purposes, scientific or historical research purposes or statistical purposes,<br>(b) is carried out in accordance with Article 89(1) of the [F302UK GDPR] (as supplemented by section 19), and<br>(c) is in the public interest.
